# Developmental stages and episode-specific regulatory genes in andromonoecious melon flower development

**DOI:** 10.1093/aob/mcad186

**Published:** 2023-12-02

**Authors:** Giuliano S Pechar, M Amelia Sánchez-Pina, Teresa Coronado-Parra, Pau Bretó, Roque Carlos García-Almodóvar, Lifeng Liu, Miguel A Aranda, Livia Donaire

**Affiliations:** Centro de Edafología y Biología Aplicada del Segura (CEBAS)-CSIC, Department of Stress Biology and Plant Pathology, PO Box 164, 30100 Espinardo, Murcia, Spain; Centro de Edafología y Biología Aplicada del Segura (CEBAS)-CSIC, Department of Stress Biology and Plant Pathology, PO Box 164, 30100 Espinardo, Murcia, Spain; Microscopy Core Facility, Área Científica y Técnica de Investigación, Universidad de Murcia, PO Box 164, 30100 Espinardo, Murcia, Spain; Abiopep S.L., R&D Department, Parque Científico de Murcia, Ctra. de Madrid, Km 388, Complejo de Espinardo, Edf. R, 2º, 30100 Espinardo, Murcia, Spain; Abiopep S.L., R&D Department, Parque Científico de Murcia, Ctra. de Madrid, Km 388, Complejo de Espinardo, Edf. R, 2º, 30100 Espinardo, Murcia, Spain; Zhengzhou Fruit Research Institute, Chinese Academy of Agricultural Sciences (CAAS), Zhengzhou 450009, Henan, China; Centro de Edafología y Biología Aplicada del Segura (CEBAS)-CSIC, Department of Stress Biology and Plant Pathology, PO Box 164, 30100 Espinardo, Murcia, Spain; Centro de Edafología y Biología Aplicada del Segura (CEBAS)-CSIC, Department of Stress Biology and Plant Pathology, PO Box 164, 30100 Espinardo, Murcia, Spain; Abiopep S.L., R&D Department, Parque Científico de Murcia, Ctra. de Madrid, Km 388, Complejo de Espinardo, Edf. R, 2º, 30100 Espinardo, Murcia, Spain

**Keywords:** Melon (*Cucumis melo* L.), andromonoecious, flower development, light microscopy, scanning electron microscopy, RNA sequencing, ABCDE model

## Abstract

**Background and Aims:**

Given the lack of specific studies on floral development in melon (*Cucumis melo* L.), we carried out an extensive study involving morphological and transcriptomic analyses to characterize floral development in this species.

**Methods:**

Using an andromonoecious line, we analysed the development of floral buds in male and hermaphrodite flowers with both light microscopy and scanning electron microscopy. Based on flower lengths, we established a correlation between the developmental stages and four main episodes of floral development and conducted an extensive RNA sequencing analysis of these episodes.

**Key Results:**

We identified 12 stages of floral development, from the appearance of the floral meristems to anthesis. The main structural differences between male and hermaphrodite flowers appeared between stages 6 and 7; later stages of development leading to the formation of organs and structures in both types of flowers were also described. We analysed the gene expression patterns of the four episodes in flower development to find the genes that were specific to each given episode. Among others, we identified genes that defined the passage from one episode to the next according to the ABCDE model of floral development.

**Conclusions:**

This work combines a detailed morphological analysis and a comprehensive transcriptomic study to enable characterization of the structural and molecular mechanisms that determine the floral development of an andromonoecious genotype in melon. Taken together, our results provide a first insight into gene regulation networks in melon floral development that are crucial for flowering and pollen formation, highlighting potential targets for genetic manipulation to improve crop yield of melon in the future.

## INTRODUCTION

Sexual dimorphism in plants is often associated with morphological and physiological characteristics that differentiate male and female reproductive organs (Wellmer *et al.*, 2013). Unlike animals, hermaphroditism (plants bearing bisexual flowers with both stamens and carpels) occurs in the vast majority of flowering plants (Ming *et al.*, 2011; Renner, 2014). A small percentage of flowering plants, ~10 %, have unisexual flowers, with both male and female flowers in the same plant (monoecious species) or in different plants [dioecious species, with only male (androecious) or female (gynoecious) flowers] (Renner, 2014). Approximately 2 % of plant species are andromonoecious, having both male and hermaphrodite flowers in the same individual (Miller and Diggle, 2003).

In the family *Cucurbitaceae*, cucumber (*Cucumis sativus* L.) and melon (*Cucumis melo* L.) present a highly polymorphic sexual system and are ideal models for understanding the genetic and epigenetic mechanisms of sex determination. In melon, all possible sex forms have been identified, except androecy, with cultivated melon varieties being predominantly andromonoecious (Papadopoulou *et al.*, 2005). In these two species, floral primordia are originally bisexual, and sex determination depends on the developmental arrest of sex-specific organs during the early stages of flower development. In cucumber, flower development has been divided conventionally into 12 developmental stages, from the floral meristem initiation stage (stage 1) to subsequent broadening and initiation of the sepal, petal, stamen and carpel primordia (stages 2–5, respectively). From stage 6 onwards, flower ontogenesis can take two different paths depending on the developmental fate of the flower. In unisexual flowers, sexual determination depends on the repression or activation of specific genes that lead to the formation of either male or female flowers exclusively. In contrast, in bisexual flowers, both male (stamen) and female (pistil) organs differentiate simultaneously, giving rise to the formation of hermaphrodite flowers (Hao *et al.*, 2003; Bai *et al.*, 2004). These 12 developmental stages have been well characterized in cucumber, through a systematic morphogenetic analysis of male and female flower development; however, in melon, the description is limited to the later stages of pollen development (stages 9–12) (Pechar *et al.*, 2022).

The genetics of sex determination is complex, and in melon, cucumber and other cucurbits, it relies on the interaction of the sex determination *Andromonoecious* (M), *Androecious* (A) and *Gynoecious* (G) loci, which are conserved. These genes have been cloned in melon, revealing that the M and A loci are *CmACS7* and *CmACS11*, respectively, and the G locus is *CmWIP1*. *CmACS7* and *CmACS11* encode two 1-aminocyclopropane-1-carboxylic (ACC) acid synthases (ACS) that are part of the ethylene biosynthetic pathway, while *CmWIP1* encodes a leucine zipper transcription factor (Boualem *et al.*, 2008, 2015, 2016). The characterization of melons harbouring natural mutations in these genes allowed researchers to propose a model in which flower sexuality depends on the differential expression of functional and non-functional proteins encoded by allelic variants of these genes. Hermaphrodite melon flowers result from the simultaneous inactivation of stamen *CmACS7* and carpel *CmWIP1* inhibitors. Male flowers in monoecious and andromonoecious lines result from the lack of expression of *CmACS11*, thus *CmWIP1* is expressed, and it can repress carpel development and *CmACS7* expression. In contrast, female flower development in monoecious melon plants is promoted by the expression of *CmACS11*, which represses *CmWIP1* and allows the expression of *CmACS7*. The andromonoecious phenotype results from the expression of a non-functional *CmACS7* allele, giving rise to hermaphrodite flowers instead of female ones (Boualem *et al.*, 2008, 2009, 2015; Martin *et al.*, 2009).

In addition to sex-determination genes, floral organ identity is controlled by MADS-box transcription factor (TF) genes. The development of sex-specific organs relies on the combinatorial and differential expression of floral ABCDE-class genes over time and space (Guo *et al.*, 2015). According to the proposed ABCDE model, the E-class proteins (SEPALLATA or SEP) form different complexes with the other A-, B-, C- and D-class proteins to specify the floral organ identities. The A- (APETALA1 or AP1) and E-class complex (AP1-SEP) is involved in the determination of sepal development; the A-, B- (AP3-PISTILLATA or AP3-PI) and E-class complex (AP1-SEP-AP3-PI) determines petals; the B-, C- (AGAMOUS or AG) and E-class complex (AG-SEP-AP3-PI) specifies stamens; the C- and E-class complex (AG-SEP) specifies carpels, and the D- (SEEDSTICK or STK) and E-class complex (STK-SEP) specifies ovules (Bowman *et al.*, 2012; Guo *et al.*, 2015). Despite extensive research on MADS-box genes in *Arabidopsis* (Pařenicová *et al.*, 2003) and cucumber (Hu and Liu, 2012), in-depth research on melon MADS-box genes has not been performed, apart from the genome-wide identification of the MADS-box gene family (Hao *et al.*, 2016). A recent study showed that the lack of expression of two melon homologues to *Arabidopsis* LIKE HETEROCHROMATIN PROTEIN1 (HP1), *CmLHP1* A and B, resulted in a pleiotropic phenotype, characterized by a general increase in the ratio of male to female flowers. This was attributable to a general deregulation of some hormonal response genes (*CmGH3* and *CmCKX1*) and a local activation of male-promoting sex determination genes and MADS-box TFs (*CmPI* and *CmSEP2*) (Rodriguez-Granados *et al.*, 2022).

Despite melon having been used extensively in the genetic determination of sex and floral morphogenesis studies, a complete morphological characterization of melon flower development is missing. In this work, we used an andromonoecious melon line to carry out a morphological study of the different stages of flower development through light microscopy and scanning electron microscopy of floral buds from male and hermaphrodite flowers. Moreover, we performed a transcriptomic analysis of four main episodes of flower development: floral structure formation (FS), gamete initiation (GI), gamete maturation (GM) and anthesis (AN). This work represents the first global study of morphological and transcriptomic aspects of flower development in melon.

## MATERIALS AND METHODS

### Plant material and tissue collection

Melon seeds of the M2 accession (cv. ‘Piel de Sapo’) were pre-germinated in Petri dishes at 25 °C for 72 h and sown in substrate (peat, coconut fibre and perlite in a 6:3:1 ratio) covered with vermiculite. Plants were grown in a greenhouse at ~25 °C (day) or 18 °C (night) in 16 h light–8 h dark photoperiod and 50–60 % relative humidity.

For the microscopy analysis, floral buds of male and hermaphrodite flowers from ~20 plants were collected by cutting the shoot tip at various stages of development and pooled according to the length of each individual bud (Table 1). The samples were separated into two equal halves for processing for light microscopy or scanning electron microscopy analysis, respectively. In both cases, flower buds were immersed in 70 % ethanol and stored at 4 °C for 24 h before fixation. For the collection of samples for RNA sequencing (RNA-Seq) and qRT-PCR, flower buds were sampled according to the procedure described above and divided into four floral episodes according to their lengths: floral structure formation episode (FS; buds <2 mm in length), gamete initiation episode (GI; floral buds of 3–5 mm), gamete maturation episode (GM; buds of 8–10 mm) and anthesis episode (AN; buds >2 cm in length) (Table 1). For each episode, ~50 buds per each three biological replicates were pooled. Buds were collected in the morning, dissected by hand and immediately frozen in liquid nitrogen and stored at −80 °C until RNA extraction. For both experiments, flower buds were collected at a time interval of 15–20 days, allowing for the formation of new flower buds between samplings.

**Table 1.** Morphological indications and respective lengths of melon (*Cucumis melo* L.) floral buds at various stages of development. Abbreviations: H, hermaphrodite; M, male.

Stage	Morphological indications	Length of floral bud (mm)^a^	Sample figures	Episode
1	Floral meristem initiation and broadening	<0.2	Fig. 1A, B	FS
2	Sepal primordia initiation	~0.2	Fig. 1C	
3	Petal primordia initiation	~0.3	Fig. 1D	
4	Stamen primordia initiation	~0.4	Fig. 1E	
5	Carpel primordia initiation	~0.55	Fig. 1F	
6	Stamen differentiates anthers and filament	0.6–0.7	Fig. 1G	
7M	Anther expands	0.6–0.7	Fig. 2A	
7H	Carpel primordia differentiates stigmas and ovary, and the space in the potential placenta becomes parallel sided	~0.9	Fig. 2K	
8M	Anther locules differentiate morphologically	~0.8	Fig. 2B, G	
8H	Stigma starts to grow beneath the stamens	1–1.5	Fig. 2L, R, S	
9M	Microsporocyte formation initiates	0.8–1	Fig. 2C, H	
9H	The placenta is clearly distinguishable; the differentiation between the stigma and the style is observed	2.5–3	Fig. 2M, T	GI
10M	Meiosis in anthers; nectary tissues initiate	2–5	Fig. 2D, I	
10H	Ovule primordia is initiated; the stigma differentiates further, and nectary tissue forms a ring	4–5.5	Fig. 2N, U	
11M	Uninuclear pollen appears; nectary tissue forms a ring	6–10	Fig. 2E, J	GM
11H	Female meiosis occurs and embryo sacs are formed	8–10	Fig. 2O, P	
12M	Mature pollen is formed; anthesis occurs	>20	Fig. 2F	AN
12H	Nectary tissues vascularize before anthesis	>20	Fig. 2Q	

^a^Floral length is given based on observations of ≥20 floral buds in the respective stages.

### Paraffin sections, light microscopy and scanning electron microscopy

The protocol of sample processing for both light and scanning electron microscopy was described previously (Pechar *et al.*, 2022). Briefly, for light microscopy, floral buds were fixed in FAA (formaldehyde: acetic acid: ethanol: H_2_O, 10:5:50:35, vol:vol), dehydrated through an ethanol series, and embedded in paraffin. Longitudinal and transverse semithin sections (1 μm) were cut using an ultra-microtome (Leica RM2155) and placed on poly-l-lysine cover slides before staining. Samples were dewaxed in an oven for 2 h at 40 °C, and three steps were carried out in Neo-Clear. Sections were then hydrated, and a first staining was done using Mayer’s Haematoxylin. A second staining was carried out with alcoholic Eosin, followed by dehydration in ethanol. Finally, dealcoholization in Neo-Clear was performed, and a drop of Neo-Mount was applied to the preparations. The sections were analysed under a light microscope (Leica DMRB; Leica Biosystems).

For scanning electron microscopy, samples were fixed overnight in FAA and subsequently subjected to several washes in 3 % glutaraldehyde diluted in 0.1 m cacodylate, then washed in cacodylate buffer plus sucrose overnight. A post-fixation procedure in 1 % tetroxide was followed by a second wash in cacodylate buffer. A serial dehydration was performed in acetone solution at increasing concentrations. Samples were critical point dried using 100 % acetone and liquid CO_2_, mounted on aluminum stubs, and analysed with a field emission scanning electron microscope ApreoS LoVac (Thermo Fisher) as described by Pechar *et al.* (2022).

### RNA isolation, RNA-Seq and data analysis

Pooled flower tissue from each sample was ground with a mortar and pestle in the presence of liquid nitrogen. Total RNA was extracted using Tri-reagent (MRC), purified with a phenol–chloroform extraction, and treated with DNase I (Sigma-Aldrich, St. Louis, MO, USA) following the manufacturer recommendations. The RNA in these preparations was quantified using a NanoDrop One (Thermo Scientific) and normalized to equal amounts for each replicate. The RNA quality of the samples was analysed by agarose gel electrophoresis, with an Agilent 2100 Bioanalyser (Agilent Technologies, USA). The RNA integrity number (RIN) of all samples was above seven. For the quantitative RT-PCR, the RNA preparations were normalized to 10 ng μL^−1^.

RNA-Seq libraries were constructed, sequenced and analysed as described before (Pechar *et al.*, 2022; Méndez‐López *et al.*, 2023). In brief, libraries were constructed using the TrueSeq Stranded mRNA LT kit (Illumina, USA), with ribosomal depletion using a Ribo-Zero plant kit (Illumina, USA), and sequenced using the Illumina NovaSeq 6000 platform (150 PE) (Macrogen Inc., South Korea). Approximately 66 × 10^6^ to 85 × 10^6^ paired reads were generated for each sample. The quality of the raw reads was analysed using FastQC (https://www.bioinformatics.babraham.ac.uk/projects/fastqc/). Low-quality reads were filtered using Trimmomatic (Bolger *et al.*, 2014) and mapped against the melon genome (DHL92/v.3.6.1) using the MEM algorithm of BWA software (Li and Durbin, 2009).

Subsequent analyses were done in R using the following R packages or functions. The *FeatureCounts* function of *Rsubread* was used to count the reads mapping mRNAs (v.4.0 of the gene models). Read counts were normalized to fragments per kilobase per million mapped reads (FPKM) using *DESeq2*. Genes were considered as expressed genes if the FPKM value was higher than one in the three biological replicates of at least one sample. Principal component analysis (PCA) and heatmaps were drawn using *Factoextra* and *Pheatmap*, respectively. Hierarchical clustering dendrograms of samples and genes were constructed using the R function *hClust* using Spearman and Pearson correlations, respectively. The K-mer selection for clustering was performed using the R function *Cutree*. Plots of gene expression patterns were drawn using *ggplot2*. Venn diagrams were made using *VennDiagram*. We used hierarchical clustering to group the genes by co-expression. For that, *hClust* was used to calculate the ‘distance’ between genes as one minus the Pearson correlation of one gene with another. This distance represents how differently one gene behaves in comparison to another and was used to construct dendrograms of gene expression, from which *k* = 16 discrete gene clusters were extracted. Episode-specific genes were defined as the genes for which the FPKM value in one episode was 2-fold the value in the remaining episodes for male and hermaphrodite flowers. Differential expression analyses among the different developmental episodes were performed using *DESeq2*. Gene Ontology (GO) analysis was done with *Goseq*. GO terms with a corrected false discovery rate <0.05 were considered to be significantly enriched.

### Quantitative RT-PCR

Primers were designed using the software Primer3 (https://www.primer3plus.com/) or were described previously (Boualem *et al.*, 2015; Supplementary Data Table S1). PCRs were performed in an optical 96-well plate with a StepOnePlus System thermal cycler (Applied Biosystems), using the One-step NZYSpeedy RT-qPCR Green kit, ROX plus (NZYTech). The cycling conditions were as follows: 50 °C for 20 s; 95 °C for 10 min; and 40 cycles of 95 °C for 15 s and 60 °C for 1 min. The *CmActin2* gene from melon was used as the reference gene, because its expression is stable among episodes (Supplementary Data Table S1). The expression levels relative to the reference were calculated using the 2^−ΔΔ*CT*^ method (Livak and Schmittgen, 2001). For MADS-box and sex-determining genes, FS was used to normalize the results of the other episodes, such that its relative expression was always one. Significance was set at the level of 0.05 by Student’s unpaired *t*-test, and the statistical analysis was performed using R basic functions.

## RESULTS

### Early developmental stages in the morphogenesis of melon flowers

**Fig. 1.**
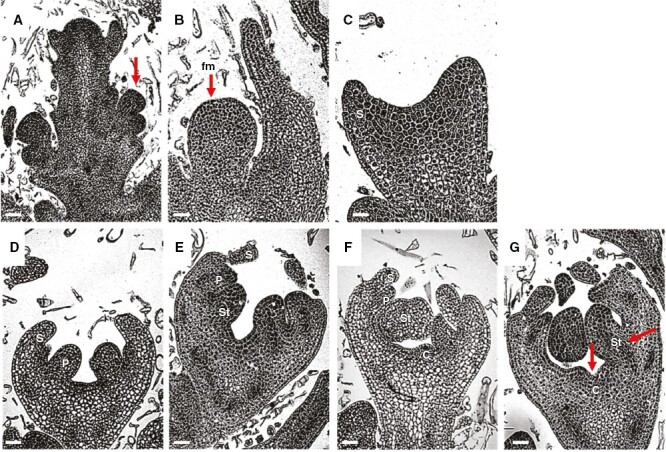
Early development of melon (*Cucumis melo* L.) flowers. Light microscopy images of shoot tip (A), floral meristem at stage 1 (B) and floral buds at stages 2 (C), 3 (D), 4 (E), 5 (F) and 6 (G). The red arrows indicate newly initiated floral buds (A), floral meristem (B) or the anther and carpel primordia (G). Abbreviations: C, carpel; fm, floral meristem; P, petal; S, sepal; St, stamen. Scale bars: 15 µm (A), 20 µm (B–D), 30 µm (E) and 40 µm (F, G).

The M2 accession of the ‘Piel de Sapo’ variety develops both male and hermaphrodite flowers. Longitudinal sections of male or hermaphrodite floral buds with different lengths were analysed by light and scanning electron microscopy. In agreement with observations of other cucurbits, we identified 12 developmental stages, ranging from meristem initiation to anthesis (Table 1) (Hao *et al.*, 2003; Bai *et al.*, 2004). Stage 1 was characterized by the formation of floral meristems in the axils of leaf primordia (red arrows in Fig. 1A, B). In stages 2–5, floral meristems underwent gradual broadening that led to the sequential initiation of sepal, petal, stamen and carpel primordia (Fig. 1C–F). In stage 6, a thickening of the stamen primordia was observed, accompanied by a constriction at the base, indicating the initial differentiation between the stamen and the filament; in contrast, the carpel primordia did not undergo significant changes in structure or size (red arrows in Fig. 1G). Consistent with observations of flower development of melon and other cucurbits, we found no evidence of structural differences between male and hermaphrodite flowers before stage 5, when the initiation of carpel primordia takes place (Fig. 1F) (Bai *et al.*, 2004; Boualem *et al.*, 2008; Martin *et al.*, 2009; Switzenberg *et al.*, 2014); therefore, the morphogenetic process specific of male or hermaphrodite flowers started from stage 6 (Fig. 1G) onwards (Table 1).

### Stages of morphogenesis specific to male melon flowers

**Fig. 2.**
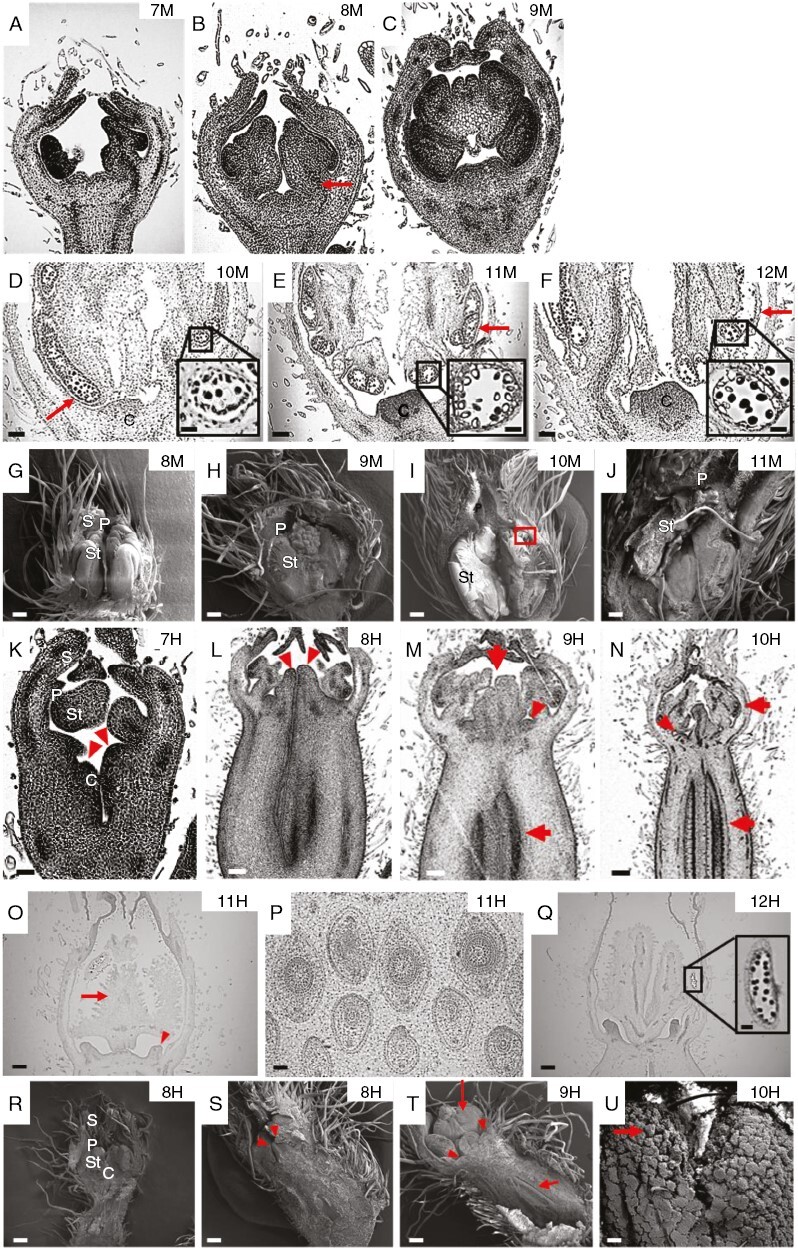
Late development of melon (*Cucumis melo* L.) flowers. Images were observed by light microscopy (A–F, K–Q) or scanning electron microscopy (G–J, R–U). Male floral buds are shown at stages 7 (A), 8 (B, G) and 9 (C, H). The arrow in B indicates vascular bundles. Part of a male floral bud are shown at stages 10 (D, I), 11 (E, J) and 12 (F). The red arrows indicate locules containing microspore mother cells and tetrads in D, uninuclear pollen in E and mature pollen in F. The red box in I indicates locules. Hermaphrodite floral buds are shown at stages 7 (K), 8 (L, R, S), 9 (M, T), 10 (N, U), 11 (O, P) and 12 (Q). The carpel primordia, the newly initiated stigmas and the nectary cells (stages 7, 8, 9 and 11, respectively) are indicated by arrowheads (in K, L, M and O, respectively). The placenta, stigma and the ovule primordia (stages 9 and 10, respectively) are indicated by red arrows (in M and N). The red arrowheads in S indicate stigma. The red arrowheads and red arrows in T indicate nectary cells and ovary, respectively. The microspore formation (stage 10) is indicated by a red arrow in U. Mature pollen in stage 12 is indicated in a black box in Q and at higher magnification in P. Abbreviations: C, carpel; P, petal; S, sepal; St, stamen. Scale bars: 50 µm (A, B, K), 100 µm (C–E, H, P), 200 µm (F, G, R, S), 250 µm (I, T), 400 µm (L), 500 µm (J, M, O, Q, U) and 1 mm (N). Scale bar in the inset in panels D–F: 30 µm; and P: 60 µm.

From stages 7 to 12, male flowers completed their development from anthers and filament differentiation to anthesis (Table 1; Fig. 2). Specifically, in stage 7, the anther primordia began to enlarge (Fig. 2A), while in stage 8, we observed the initiation of locule differentiation, together with the appearance of vascular bundles in the filaments (Fig. 2B, G). Given that there were no further distinguishable morphological changes in the anther, the later developmental stages were defined according to the differentiation of reproductive cells. Therefore, stage 9 was defined by microsporocyte formation (Fig. 2C, H), stage 10 by meiosis (Fig. 2D, I), stage 11 by uninuclear pollen appearance (Fig. 2E, J) and stage 12 by mature pollen formation (Fig. 2F).

### Stages of morphogenesis specific for hermaphrodite melon flowers

In stage 6, the differences between male and hermaphrodite flowers were very subtle, and the simultaneous development of the male and female structures made it difficult to identify the initiation of the stigma clearly at this stage. The first indication of differentiation was the elongation of carpel primordia, which occurred simultaneously with the differentiation of the stamen between anther and filament (Fig. 1G). In stage 7, the elongation of the carpel primordia was accentuated, creating a parallel-sided invagination into the space of the potential placenta, while the developing stigma almost reached the connection between the newly formed filament and the anther (Fig. 2K). From stage 8 onwards, the development of male and female reproductive structures occurred simultaneously. In stage 8 of hermaphrodite flowers, the primordial stigma reached a height approximately equal to that of the developing stamen (Fig. 2L, R, S). The male structures of the hermaphrodite flower at stage 8 were characterized by the enlargement of the anther primordia (corresponding to stage 7 in the development of the male flower; Table 1; Fig. 1A). In stage 9, the placenta was clearly distinguishable, the differentiation between stigma and style was evident, and nectary cells started to initiate at the base of the style (red arrowheads in Fig. 2M, T). In stage 10, the ovule primordia were initiated (red arrow in Fig. 2N), the papillae cells were differentiated on the stigmas (Fig. 2U), and the nectary cells clearly rose as a dome (Fig. 2N). The male structures of the hermaphrodite flower at stage 10 were characterized by microspore formation. During stage 11, the integuments were initiated (not shown), the stigma differentiated further (red arrow in Fig. 2O), and the nectary tissue formed a ring (red arrowhead in Fig. 2O). At stage 11, meiosis occurred, and embryo sacs were formed (Fig. 2P). Male structures at stage 11 were characterized by the presence of uninucleate pollen after meiosis. Finally, in stage 12, nectary tissue vascularized before it broke for anthesis (Fig. 2Q). Mature pollen was observed in anthers at this stage (inset in Fig. 2Q).

### Transcriptome profiling throughout melon flower developmental episodes

The stages defined above involved subtle morphological changes that could not be observed without a proper microscopy study. Therefore, for the transcriptome analysis, flowers were collected and pooled according to their sizes (Table 1), with pools corresponding to four main episodes during floral development. For the FS episode, which is essentially shared between male and hermaphrodite flowers, the buds were <2 mm in length, probably grouping stages 1–8 together with stage 9M (Table 1). For the GI episode, the floral buds were 2–5 mm in size, and we collected flowers probably at stage 10 (Table 1). For the GM episode, buds were 8–10 mm, corresponding to stage 11 (Table 1). Finally, for the AN episode, floral buds were >2 cm. Logically, for the GI, GM and AN episodes, male and hermaphrodite buds were differentiated, giving rise to pools GI-M/H, GM-M/H and AN-M/H, in addition to the FS pool.

**Fig. 3.**
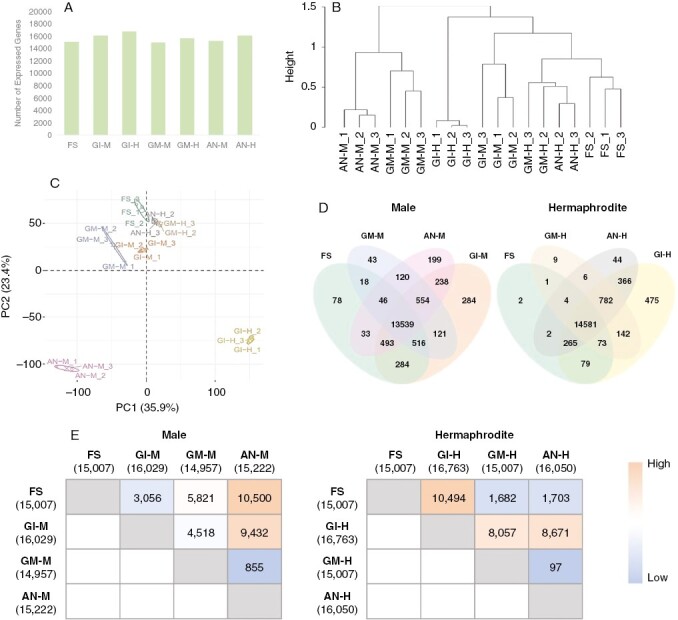
RNA sequencing analysis throughout flower developmental episodes. Four episodes during floral development were defined: formation of floral structures (FS), gamete initiation (GI), gamete maturation (GM) and anthesis (AN). (A) Number of expressed genes per episode. Expressed genes are considered those with an average value of fragments per kilobase per million mapped reads (FPKM) > 1 in at least one episode. (B) Cluster dendrogram of gene expression profiles between biological replicates and among different developmental episodes. The dendrogram shows the hierarchical clustering of the different replicates according to their gene expression profiles. The *y*-axis represents the height of the branches and indicates how similar or different the replicates/samples are from each other using the complete agglomeration method in *hclust* R function. (C) Principal component analysis (PCA) scores plotted for male and hermaphrodite floral development episodes. The PCA was computed using expressed genes. Abbreviations: PC1, principal component 1; PC2, principal component 2. The percentages of variance explained by PC1 and PC2 are 35.9 % and 23.4 %, respectively. Confidence ellipses were plotted around group mean points. (D) Venn diagrams showing the numbers of expressed genes specific or shared among different developmental episodes for male and hermaphrodite flowers. Venn diagrams were drawn using expressed genes. (E) Pairwise comparison matrixes showing the number of differentially expressed genes (DEGs) between the specified melon male and hermaphrodite flower developmental episodes. The numbers within each matrix correspond to the number of DEGs between each of the comparisons. Numbers in parentheses indicate the number of genes expressed in each episode.

Total RNA was subjected to next-generation sequencing using an Illumina platform. The number of genes expressed in each stage, defined here as those genes whose average FPKM value was higher than one in at least one episode, was similar in all episodes, ~15 500 genes (Fig. 3A). A cluster dendrogram analysis of gene expression profiles, together with a PCA, showed that gene expression patterns among biological replicates were highly related except for two replicates, one for GM-H and the other for AN-H (Supplementary Data Fig. S1). When these two anomalous replicates were excluded from the analysis, both the PCA and the hierarchical clustering analysis showed that replicates clustered together (Fig. 3B, C), providing confidence in the use of floral bud size as an indicator of the flower developmental episodes. Therefore, for the analyses described next, all the treatments had three replicates, except for GM-H and AN-H, which had two. In addition, the PCA showed a separation between the clusters of male and hermaphrodite samples, especially noticeable for male flowers at anthesis (AN-M) and hermaphrodite buds at gamete initiation (GI-H), from all the other samples and from each other (Fig. 3C). Regarding the expressed genes, 13 539 (80 %) were expressed in all four episodes in male flowers, whereas 14 581 (86 %) were expressed in hermaphrodite flowers in all the episodes (Fig. 3D). The number of genes expressed in only one episode in male flowers was 78, 284, 43 and 199 for FS, GI, GM and AN, respectively (Fig. 3D), suggesting a more complex gene expression landscape during gamete initiation and anthesis in comparison to the other episodes. The number of genes expressed uniquely in the hermaphrodite flowers was 2, 425, 9 and 44 for FS, GI, GM and AN, respectively (Fig. 3D), reflecting the same general trend observed for the male flowers.

### Differentially expressed genes in male and hermaphrodite melon flowers

To identify differentially expressed genes (DEGs), pairwise comparisons were performed between all possible pair combinations of our datasets across the four developmental episodes (Fig. 3E). For male flowers, the number of DEGs was correlated with the developmental distance between episode pairs, i.e. the largest number of DEGs was found between FS and AN-M (10 500 genes), whereas the contiguous episodes GM-M and AN-M had the smallest number of DEGs (855 genes) (Fig. 3E). In contrast, for hermaphrodite flowers, this trend was not clear; the largest number of DEGs occurred between FS and GI-H (10 494 genes), whereas FS and AN-H had a moderate number of DEGs (1703 genes) (Fig. 3E). In total, the pairwise comparisons identified 12 469 and 11 574 DEGs in male and hermaphrodite flowers, respectively. These genes were considered in the later analyses.

**Fig. 4.**
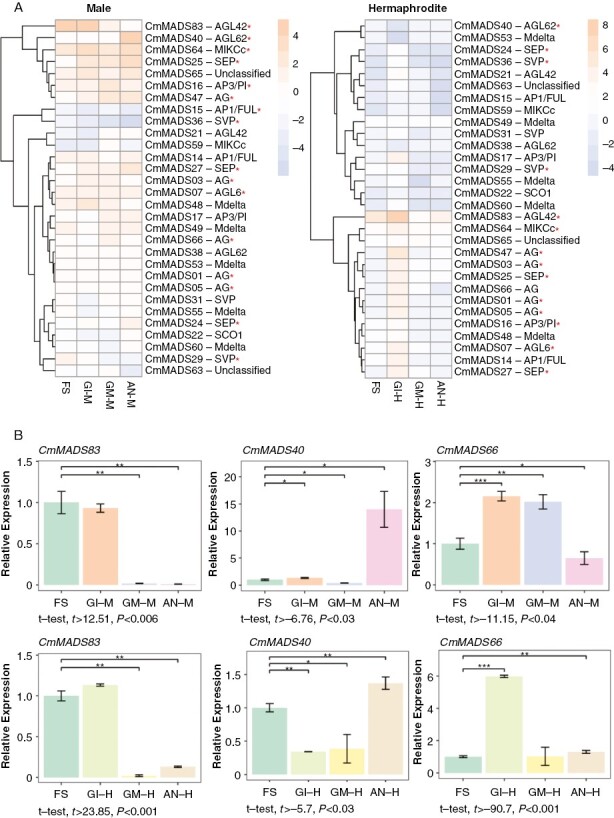
Differential expression of MADS-box transcription factors during floral development in melon. (A) Clustered heatmap showing expression of melon MADS-box genes in both male (left) and hermaphrodite (right) flower developmental episodes. Genes are named and classified according to their distribution in subfamilies based on the phylogenetic relationships with their homologues in *Arabidopsis* and cucumber (Hao *et al.*, 2016; Supplementary Data Table S2). Gene expression was represented as scaled fragments per kilobase per million mapped reads (FPKM) values for male or hermaphrodite episodes separately. The genes discussed in the text are marked with a red asterisk. (B) Quantitative RT-PCR validation of MADS-box transcription factors expression during male and hermaphrodite floral development. The *Actin2* RNA was used as an endogenous reference to normalize the qPCR results. The RNA corresponding to each episode was extracted from a pool of three biological replicates. Each bar represents the average of three technical replicates. Error bars indicate the s.d. Asterisks represent statistically significant differences among pairwise comparisons (FS vs. the other three episodes). Significance was set at the level of 0.05 by Student’s unpaired *t*-test, with *P > 0.01, **P > 0.001 and ***P *≈* 0. The statistical analysis was performed with R software. In the case of significant differences, the lower *t* and the higher *P*-values of all pairwise comparisons are shown below each plot.

Initially, we searched for MADS-box TF genes in the list of all DEGs to study their levels of expression throughout the floral developmental episodes. For this, we constructed a heatmap using their scaled FPKM values in each of the four episodes and we used it to find the MADS-box TF genes that had distinctive patterns of expression and a described function in floral development (Fig. 4A; Supplementary Data Table S2). The results showed that the melon homologue to the *Arabidopsis* MADS-box gene encoding the protein AGL42 (*CmMADS83*) had elevated expression levels at the FS and GI episodes for both male and hermaphrodite flowers, and its expression levels declined throughout the flowering process (Fig. 4A). This result was confirmed by qRT-PCR using an RNA pool of the three biological replicates per episode of the sequenced samples (Fig. 4B). The melon homologue of the MADS-box gene encoding the protein SOC1 (*CmMADS64*) had sustained expression with slight fluctuations in both hermaphroditic and male flowes (Fig. 4A). *CmMADS25* and *CmMADS27*, belonging to the SEP subfamily, showed an increasing expression pattern throughout male flower development, whereas in the hermaphrodite flower, expression was concentrated in the GI episode. In contrast, *CmMADS36* and *CmMADS15*, from the SVP and AP1 subfamilies, respectively, were downregulated throughout floral development for both sexual types (Fig. 4A). The melon homologue of AGL62 (*CmMADS40*) was downregulated throughout development in hermaphrodite flowers and highly expressed in male flowers only in the AN episode, and this result was verified by qRT-PCR (Fig. 4A, B). Finally, we also confirmed the RNA-Seq data on *CmMADS66*, an AGAMOUS MADS-box TF, by qRT-PCR, for which the expression was higher in the intermediate episodes GI and GM in male and hermaphrodite flowers, respectively (Fig. 4A, B).

A more general approach was taken next. A clustering analysis was performed on the total number of DEGs for male and hermaphrodite flowers, with *k* (number of clusters) set to 16 (Supplementary Data Fig. S2A). Different clusters grouped genes with similar expression patterns that were likely to be associated with their related functions throughout melon floral development. In the case of male flowers (Supplementary Data Fig. S2B), 3205 genes were found in clusters 2, 3 and 15, showing the highest expression values during FS, and these genes were downregulated at later flower developmental episodes. The 749 genes in clusters 4 and 11 showed a GI-specific pattern, while only cluster 5, with 745 genes, had the highest expression values in the GM episode. Finally, the 1777 genes in clusters 7 and 14 had expression patterns showing a peak in AN (Supplementary Data Fig. S2B). For hermaphrodite flowers, all 497 genes in clusters 5, 12 and 15 had a peak of expression in the FS episode, and these were downregulated over the course of floral development. In addition, 5865 genes in clusters 1, 8, 9, 10 and 11 had a specific GI pattern, while only 92 genes in cluster 14 had elevated GM expression. Finally, 1002 genes in clusters 7 and 16 had an expression peak in AN-H (Supplementary Data Fig. S2C).

As a general conclusion, although clustering showed that genes can be deregulated in more than one episode during floral development, some clusters showed a specific upregulation in only one episode, suggesting that the genes in these clusters could be the main drivers of floral transitions during development.

### Episode-specific genes during melon floral development

**Fig. 5.**
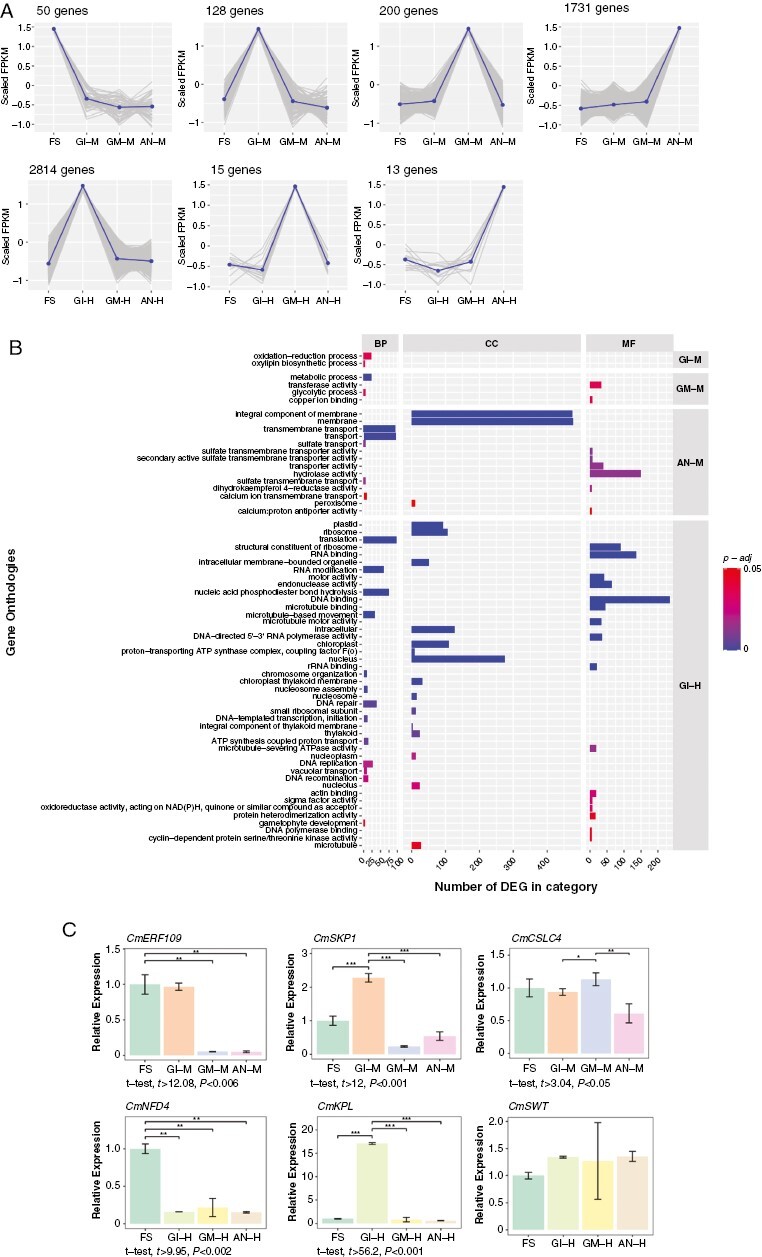
Episode-specific genes and enriched Gene Ontology (GO) categories during melon floral development. (A) Expression patterns of episode-specific genes in FS, GI-M, GM-M, AN-M, GI-H, GM-H and AN-H, respectively. The *y*-axis represents scaled fragments per kilobase per million mapped reads (FPKM) of genes across the four developmental episodes, where the grey lines represent the values for each gene in a cluster, and the blue line represents the core (*k*-medoids) of each cluster. (B) Enriched GO terms of episode-specific genes for GI-M, GI-H, GM-H and AN-H episodes in each GO category: BP, biological process; CC, cellular component; and MF, molecular function. The *y*-axis indicates the enriched categories, and the colour scale indicates the *P*-adjusted value. The *x*-axis indicates the number of episode-specific genes found in each category. (C) Quantitative RT-PCR expression of episode-specific genes during male and hermaphrodite floral development. The *Actin2* gene was used as an endogenous reference to normalize the qPCR results. The RNA corresponding to each episode was extracted from a pool of three biological replicates. Each bar represents the average of three technical replicates. Error bars indicate the s.d. Asterisks represent statistically significant differences among pairwise comparisons (FS vs. the other three episodes). Significance was set at the level of 0.05 with Student’s unpaired *t*-test, with *P > 0.01, **P > 0.001 and ***P *≈* 0. The statistical analysis was performed with R software. In the case of significant differences, the lower *t* and the higher *P*-values of all pairwise comparisons are shown below each plot.

In order to identify genes that were expressed differently in only one of the flower development episodes, we compared the expression of DEGs among episodes. We considered as episode-specific genes those DEGs that had a level of expression 2-fold or more in one episode above that of the remaining episodes, for both flower types separately. Based on this, we identified 50 FS episode-specific genes (Fig. 5A). A GO analysis did not detect any enriched category for this episode. Among the FS-specific genes, we found homologues of the protein FANTASTIC FOUR 2, and floricaula/leafy homologue, which are involved in anther development in *Arabidopsis* (Wahl *et al.*, 2010; Siriwardana and Lamb, 2012), in addition to the cytochrome P450 78A5 and growth-regulating factor 4-like, which have been associated with floral organ development (Zondlo and Irish, 1999; Kim and Lee, 2006). Characteristic hormone-related genes in this episode were those involved in ethylene signalling (Supplementary Data Table S3). A total of 128 genes were classified as GI-M specific (Fig. 5A). The GO enrichment analysis showed that this episode was enriched in ‘oxide-reduction’ and ‘oxylipin biosynthetic’ biological processes (Fig. 5B). Among the genes specifically expressed during this episode, we found those encoding SKP1-like protein 12, ECERIFERUM 1-like protein and WAT1-related protein. The *Arabidopsis* homologue of the former is involved in early flower reproductive development, whereas for the other two, specific roles in exine biosynthesis and pollen development have been described (Bourdenx *et al.*, 2011; Dezfulian *et al.*, 2012; Supplementary Data Table S3).

We then examined the 200 genes that were expressed specifically in GM-M (Fig. 5A). A GO analysis showed that the most enriched category was ‘metabolic processes’ (Fig. 5B). Among the GM-M-specific genes, we found those encoding xyloglucan glycosyltransferase 4 and tubulin beta chain, among others. The *Arabidopsis* homologue of the former seemed to be involved in gamete formation and plant reproduction, whereas for the latter, a role in the mitotic cycle has been described (Ferreira *et al.*, 2018; Kim *et al.*, 2020; Supplementary Data Table S3).

Substantially more genes (1731) were expressed specifically in AN-M (Fig. 5A). These genes were enriched in numerous GO terms related to membranes within the cellular component category and to biological processes related to transport (Fig. 5B). When checking the genes that were expressed specifically in AN-M, we found *Arabidopsis* homologue genes involved in sporopollenin biosynthesis, such as protein ECERIFERUM 3; genes responsible for the programmed cell death process, such as those encoding accelerated cell death 11; genes related to germination and maturation of pollen, such as pollen receptor-like kinase 1; and genes controlling photoperiodism and flowering, such as zinc finger protein CONSTANS-LIKE 2 and protein GIGANTEA-like (Supplementary Data Table S3).

A total of 2814 genes related to hermaphrodite gamete initiation were classified as GI-H specific (Fig. 5A). A GO enrichment analysis showed that this episode was enriched in ‘plastid’ and ‘ribosome’ within the cellular component category; ‘translation’ and ‘RNA modification’ biological processes and molecular functions related to ‘ribosome’ and ‘RNA binding’, among others (Fig. 5B). In this episode, we found the largest number of specific genes, many of which were *Arabidopsis* homologues related to general floral development, such as those encoding the PHOTOPERIOD-INDEPENDENT EARLY FLOWERING 1 family protein or the zinc finger protein CONSTANS. We also found genes involved in female gamete development, such as those encoding the DYAD protein (female meiosis), E3 ubiquitin-protein ligase Arkadia (embryonic development) or protein YABBY 4-like (ovule development). Specific male gamete development genes were also identified in this episode, such as the genes encoding Apoptosis inhibitor 5-like protein API5 (involved in programmed cell death during anther development) and pollen Ole e 1 family protein. A gene of particular relevance to hermaphrodite flowers was *Kokopelli*, which plays a role in double fertilization (Agashe *et al.*, 2002; Ron *et al.*, 2010). We also detected several members of MADS-box T (as described above) and Myb TFs (Supplementary Data Table S2).

The GM-H and AN-H episodes showed only 15 and 13 specific genes, respectively (Fig. 5A). Although the enrichment analysis did not detect any GO category that was significantly overrepresented for them, among the GM-H-specific genes, we found *Arabidopsis* homologues that play key roles in floral development, such as the gene encoding PHD finger protein MALE STERILITY 1, which is a transcriptional activator required for anther and post-meiotic pollen development and maturation (Wilson *et al.*, 2001). Finally, among the AN-H specific genes, we found homologues of the genes encoding protein nuclear fusion defective 4 and WAT-1 related protein, which have been associated with the process of karyogamy during female gametophyte development and flower development (Portereiko *et al.*, 2006; Supplementary Data Table S3).

To confirm that these genes were episode-specific genes, we quantified the expression of six of them by qRT-PCR (Fig. 5C). The selected genes were specific for FS (*CmERF109* and *CmNFD4*), GI-M (*CmSKP1*), GI-H (*CmKPL*), GM-M (*CmCSLC4*) and GM-H (*CmSWT*) (Supplementary Data Table S3). The expression pattern of all of them was similar to the transcriptome sequencing data; however, the qRT-PCR expression pattern of *CmSWT* was not consistent with the sequencing data (Fig. 5C).

### Expression of sex determination genes during melon floral development

**Fig. 6.**
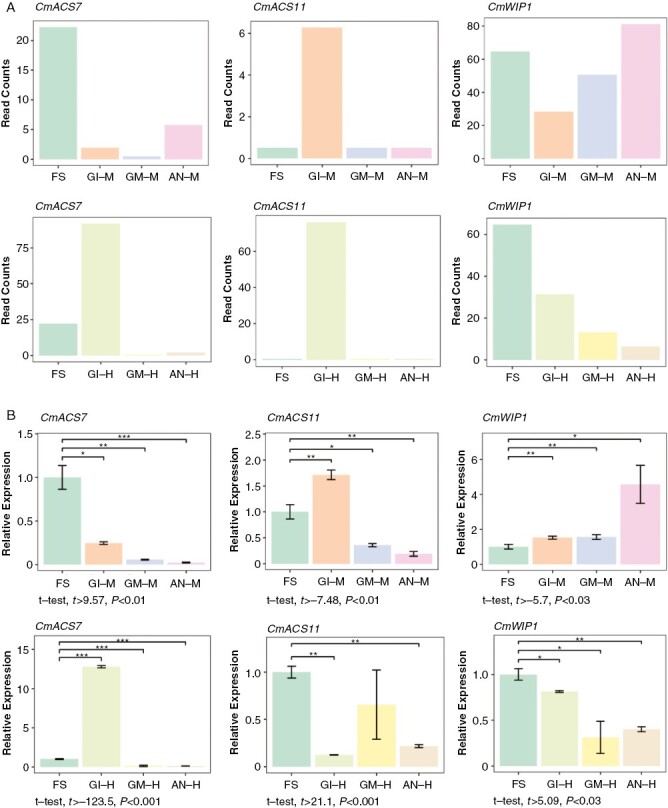
Expression patterns of sex-determining genes across floral developmental episodes in male and hermaphrodite flowers. (A) Gene expression of *ACS11*, *ACS7* and *WIP1* measured in fragments per kilobase per million mapped reads (FPKM) by RNA-Seq analysis. (B) Gene expression of *ACS11*, *ACS7* and *WIP1* by quantitative RT-PCR. The *Actin2* gene was used as an endogenous reference to normalize the qPCR results. The RNA corresponding to each episode was extracted from a pool of three biological replicates. Each bar represents the average of three technical replicates. Error bars indicate the s.d. Asterisks represent statistically significant differences among pairwise comparisons (FS vs. the other three episodes). Significance was set at the level of 0.05 with Student’s unpaired *t*-test, with *P > 0.01, **P > 0.001 and ***P *≈* 0. The statistical analysis was performed with R software. In the case of significant differences, the lower *t* and the higher *P*-values of all pairwise comparisons are shown below each plot.

Finally, we investigated the expression patterns of the sex determination genes *CmACS11*, *CmACS7* and *CmWIP1*, using both RNA-Seq and qRT-PCR, obtaining similar results. *CmACS11* showed a pattern of expression specific to the GI episode, in both male and hermaphrodite flowers, although the expression was higher in GI-H (Fig. 6). *CmACS7* showed a decreasing expression throughout flower development in male flowers, with a small increase corresponding to the AN-M episode, while the *CmACS7* profile in the hermaphrodite flower was characterized by a peak corresponding to the GI-H episode (Fig. 6). *CmWIP1* was characterized by having opposite expression patterns throughout flower development in male and hermaphrodite flowers; in male flowers its expression increased, whereas in hermaphrodite flowers the expression of *CmWIP1* decreased with development (Fig. 6). These results are in agreement with previous data for other cucurbits (Boualem *et al.*, 2008, 2009, 2015; Martin *et al.*, 2009).

## DISCUSSION

Most studies of floral development in the *Cucurbitaceae* family have been carried out on monoecious accessions. In contrast, only a few studies have focused on the characterization of andromonoecious varieties (Boualem *et al.*, 2008; Ge *et al.*, 2020). Here, we decided to use an andromonoecious accession, because andromonoecious melons constitute a significant portion of the currently cultivated varieties. As in sex-separated accessions, stages 1–5 were shared by male and hermaphrodite flowers, and the first structural differentiations between both types of flowers appeared between stages 6 and 7. The developmental patterns observed here were conserved between melon and cucumber and did not appear to be affected greatly by environmental conditions, because different samplings were carried out at different times of the year.

By establishing a correlation between developmental stages and episodes, our aim was to try to identify the patterns of gene expression that were specific to a given episode of floral development and to identify genes that determined the passage from one episode to the next. The hierarchical clustering analysis, together with the PCA, showed that gene expression patterns among biological replicates were highly related, providing confidence in the use of floral bud size as an indicator of the flower development episodes. In addition, the PCA showed clear separation of male flowers at anthesis and hermaphrodite buds at gamete initiation. A certain tendency to group together could be detected for the remaining episodes, especially for the consecutive GM and AN episodes in hermaphrodite flowers (Fig. 3B, C).

The number of genes identified as expressed genes was within the expected range for a transcriptome such as that of melon. Of these, 80 % were expressed in all four episodes in male flowers and 86 % in hermaphrodite flowers during all episodes. An analysis of the number of genes expressed in only one episode suggested a more complex gene expression landscape during the gamete initiation and anthesis episodes in comparison to the other ones for both male and hermaphrodite flowers (Fig. 3D). This could be attributable to a shift in transcriptomic activity during these stages of flower development. Plants, in fact, do not have a proper germ line, but are characterized by alternating sporophytic and haploid gametophytic stages that differ in their development and role in reproduction, meaning that the function and evolution of genes expressed in sporophytes and gametophytes are likely to diverge. During plant gametogenesis and anthesis, the transcription profile changes dramatically, and genes involved in reproduction are enriched in these phases (Schmid *et al.*, 2005; Gossmann *et al.*, 2016).

In general, for male flowers, numbers of DEGs were correlated with the developmental distance between episode pairs. In contrast, for hermaphrodite flowers, this trend did not emerge so clearly, because the largest number of DEGs was detected between FS and GI-H, whereas FS and AN-H had a smaller number of DEGs (Fig. 3E). This could appear to be somehow counter-intuitive but agrees well with the PCA and clustering analyses, where episodes FS, AN-H and GM-H appeared close (Fig. 3B, C). This difference in trend can be explained by the differences in terms of development observed in the analysis carried out by microscopy. In the male flower, the development of the reproductive organs followed a linear progression across the episodes, whereas in the hermaphrodite flower, the male reproductive organs started to develop earlier than the female ones. It was only at stage 7 when the carpel primordia began to differentiate into stigma and ovary, and it was at stage 8 when the stigma could be observed growing below an already partly developed anther. Thus, it was in the GI-H episode that the process of initiation of the gametes of the male and female structures within the same flower truly came together. In the hermaphrodite flower, the developmental processes of male and female gametes within the same flower could depend on the expression of specific and distinct genes between male and female gametes, resulting in a cumulative effect on the number of DEGs coinciding with the GI-H episode, and this would be mitigated by progressing towards a stage where the development of floral structures predominates over gamete developmental processes.

Among the MADS-box TF genes that might play a role in the floral development in melon, the melon homologue to the *Arabidopsis* MADS-box protein AGL42 (*CmMADS83*)-encoding gene is a strong candidate for this function; *Arabidopsis* AGL42 has been reported to play a role in promotion of flowering at the shoot apical and axillary meristems (Dorca-Fornell *et al.*, 2011). This gene showed elevated expression levels in the FS and GI episodes for both male and hermaphrodite flowers, and its expression levels declined, as expected, throughout the flowering process. Homologues of the MADS-box gene encoding protein SOC1 (*CmMADS64*), which is a transcription activator active in flowering time control (Immink *et al.*, 2012), were highly expressed in both hermaphrodite and male flowers, which was maintained, with some fluctuations, throughout the entire process of flower development. Moreover, *CmMADS36* and *CmMADS15*, of the AP1 and SVP subfamilies, respectively, involved in the determination of floral meristem and sepal identity in many plant species, including *Arabidopsis* (Gregis *et al.*, 2008, 2009), were downregulated throughout floral development for both sexual types. The analysis of MADS-box TF expression patterns coincided, in part, with the ABCDE model of floral development in *Arabidopsis* (Bowman *et al.*, 2012; Guo *et al.*, 2015). It has been described that the complex AG-SEP-AP3 specifies stamens. In our study, some MADS-box genes belonging to these subfamilies (*CmMADS03*, *CmMADS07*, *CmMADS16* and *CmMADS25*) had elevated expression patterns in the male (and hermaphrodite) flower, especially in the mid floral developmental episodes and, in general, after the FS episode. Genes encoding the AG-SEP complex (AG subfamily: *CmMADS01*, *CmMADS03*, *CmMADS05*, *CmMADS47* and *CmMADS66*; and SEP subfamily: *CmMADS25* and *CmMADS27*), which specifies carpels, showed elevated expression patterns in correspondence to the GI-H episode. Genes encoding the STK-SEP complex (*CmMADS24*, *CmMADS25* and *CmMADS27*), which specifies ovules, had a peak of expression in GI-H, and were maintained at a certain basal level throughout development in the hermaphrodite flower. Finally, the SVP-like MADS-box gene *CmMADS29* was expressed mostly during the FS episode and was downregulated over the course of floral development. These conserved expression patterns might suggest that major function of this key gene is conserved across the plant kingdom (Gramzow and Theißen, 2015; Schilling *et al.*, 2018).

To our knowledge, very little is known about the expression and function of MADS-box TF genes in melon. Most studies have been carried out in cucumber, and all correlated the expression of these genes with different reproductive developmental functions and fruit maturation (Cheng *et al.*, 2019, 2020). The transition of the melon inflorescence from meristematic status to floral organ differentiation is accompanied by the downregulation of meristem activity genes and a significant upregulation of a large number of genes associated with organ development. As in other plants, melon homologues of various MADS-box genes might play central roles during this process.

The overall number of episode-specific genes was different between male and hermaphrodite flowers. This was consistent with the trend observed for the number of DEGs between pairs of episodes described above. The genes found to be overexpressed specifically during each episode provided further evidence of the stage–episode correspondence. In the first episode, many genes related to floral organ growth and development of anther structures were detected, which correspond to the processes characterizing the different stages observed through microscopy that were grouped together to identify FS. Likewise, the GI episode was characterized by the presence of genes related to early flower reproductive and pollen development in male flowers and genes related to female gamete development in hermaphrodite flowers. In the FM episode, genes involved in gamete formation, mitotic cycle and pollen tube initiation were observed for male flowers, whereas in the hermaphrodite, post-meiotic pollen development and maturation-related genes were detected. Finally, in the AN episode, genes related to photoperiodism and flowering were observed. Among the genes overexpressed throughout flower development, we also found several genes involved in ethylene signalling, many homologues of the cytochrome p450 family, in addition to genes of the ABC transporter family (A, B, C, D, F and I). This was in agreement with what was described in melon (Dai *et al.*, 2019). In general, the analysis of the enriched terms also gave us a broader view of the set of processes that take place during flower development, and the results seemed to indicate that metabolic and nuclear processes prevail in the early stages of development, whereas membrane-associated processes predominate in the later stages. This is consistent with what has been observed for other species, such as wheat (Feng *et al.*, 2017) or *Arabidopsis* (Honys and Twell, 2003).

The determination of differences in gene expression between the consecutive episodes GI and GM in hermaphrodite flowers via qRT-PCR was challenging; however, in general, both the RNA-Seq and qRT-PCR data showed congruent expression patterns of the sex determination genes, in agreement with the model described previously (Boualem *et al.*, 2015); such that the male flower in our andromonoecious accession was characterized by the lack of *CmACS11* expression and the consequent expression of *CmWIP1* throughout floral development, which, in turn, represses *CmACS7* expression and carpel development. In contrast, hermaphrodite flowers were characterized by the expression of *CmACS11*, which represses *CmWIP1*, and by the expression of a probably non-functional version of the *CmACS7* allele, which is not capable of suppressing stamen development, giving rise to hermaphrodite flowers instead of female ones (Fig. 6A). Recently, a new study was published that described *CRABS CLAW* (*CRC*), a transcription factor of the YABBY gene family controlling carpel and nectary development, in addition to flower meristem termination, as a new player in the sex determination model in melon (Zhang *et al.*, 2022). Interestingly, in our RNA-Seq data this gene was overexpressed during the GI-H episode.

### Conclusions

To our knowledge, this is the only work combining a detailed morphological analysis and a comprehensive transcriptomic study for characterization of the structural and molecular mechanisms that determine the floral development of an andromonoecious genotype in melon. Taken together, our results provide new insights into gene regulatory networks in melon floral development that might be crucial for floral and pollen development, highlighting potential targets for genetic manipulation to improve melon yield in the future. In this sense, the CRISPR/Cas technology constitutes a valuable tool for the identification of gene functions and the study of the mechanisms underlying floral development in cucurbits for fundamental research (Pechar *et al.*, 2022) or, once identified, to take advantage of potential biotechnological applications through genome editing, as in the case of the breeding of non-transgenic gynoecious cucumber for hybrid production (Hu *et al.*, 2017).

## SUPPLEMENTARY DATA

Supplementary data are available at *Annals of Botany* online and consist of the following.

Table S1: primers for qRT-PCR used in this study. Table S2: scaled FPKM values of MADS-box transcription factor genes. Table S3: differentially expressed gene (DEG) results of RNA-Seq data. Fig. S1: quality control of RNA-Seq samples. Fig. S2: gene clustering analysis of differentially expressed genes.

mcad186_suppl_Supplementary_Figures_S1-S2

mcad186_suppl_Supplementary_Table_S1

mcad186_suppl_Supplementary_Table_S2

mcad186_suppl_Supplementary_Table_S3
