# Supplementary material for: Developmental stages and episode-specific regulatory genes in andromonoecious melon flower development
Source: Ann Bot. 2023 Dec 2;133(2):305–20. doi: 10.1093/aob/mcad186 (PMC11005788; doi:10.1093/aob/mcad186)
Supplement: mcad186_suppl_Supplementary_Figures_S1-S2 [file mcad186_suppl_supplementary_figures_s1-s2.pptx]

## Slide 1
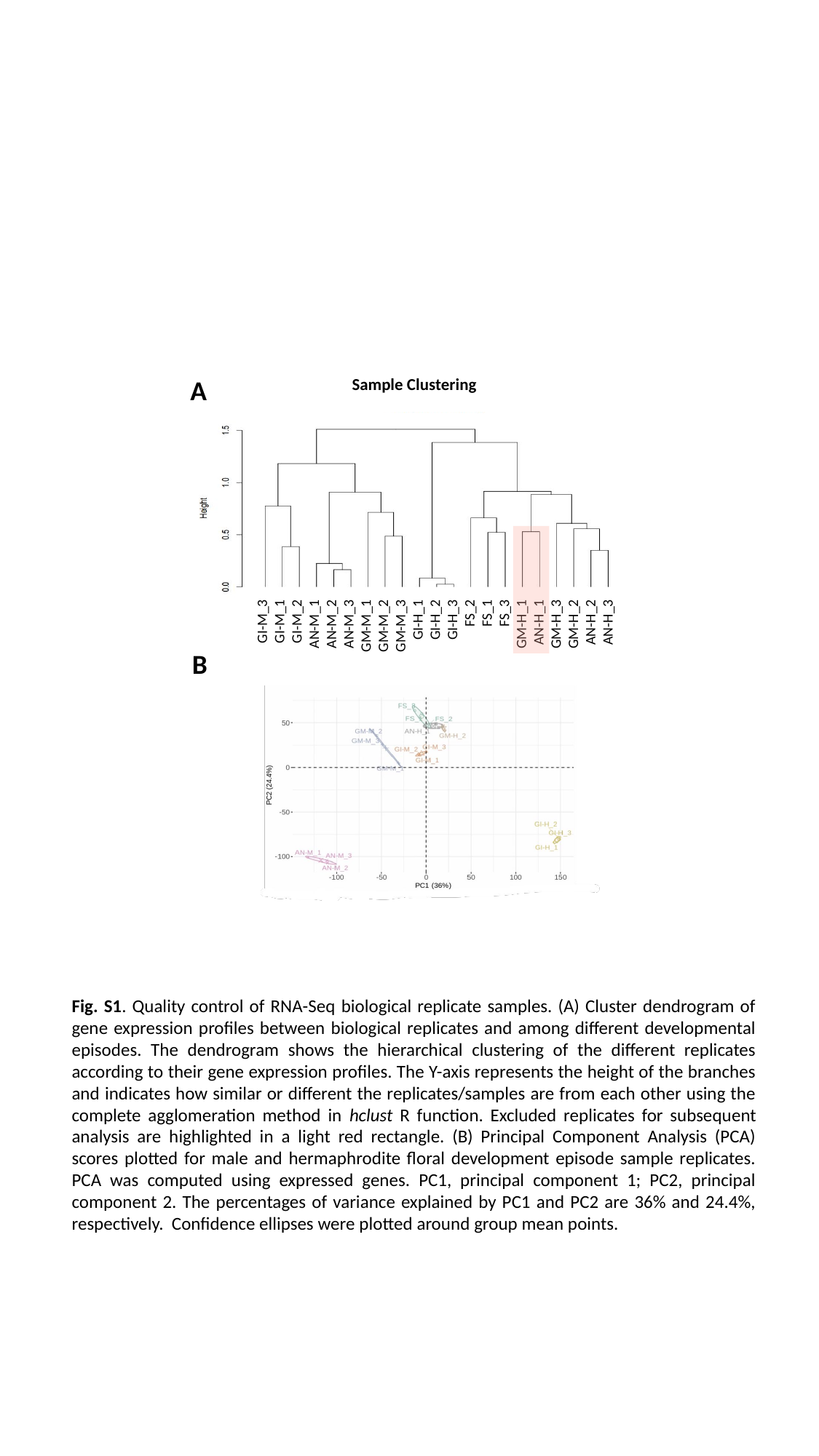

A
Sample Clustering
GI-M_3
GI-M_1
GI-M_2
AN-M_1
AN-M_2
AN-M_3
GM-M_1
GM-M_2
GM-M_3
GI-H_1
GI-H_2
GI-H_3
FS_2
FS_1
FS_3
GM-H_1
AN-H_1
GM-H_3
GM-H_2
AN-H_2
AN-H_3
B
Fig. S1. Quality control of RNA-Seq biological replicate samples. (A) Cluster dendrogram of gene expression profiles between biological replicates and among different developmental episodes. The dendrogram shows the hierarchical clustering of the different replicates according to their gene expression profiles. The Y-axis represents the height of the branches and indicates how similar or different the replicates/samples are from each other using the complete agglomeration method in hclust R function. Excluded replicates for subsequent analysis are highlighted in a light red rectangle. (B) Principal Component Analysis (PCA) scores plotted for male and hermaphrodite floral development episode sample replicates. PCA was computed using expressed genes. PC1, principal component 1; PC2, principal component 2. The percentages of variance explained by PC1 and PC2 are 36% and 24.4%, respectively. Confidence ellipses were plotted around group mean points.

## Slide 2
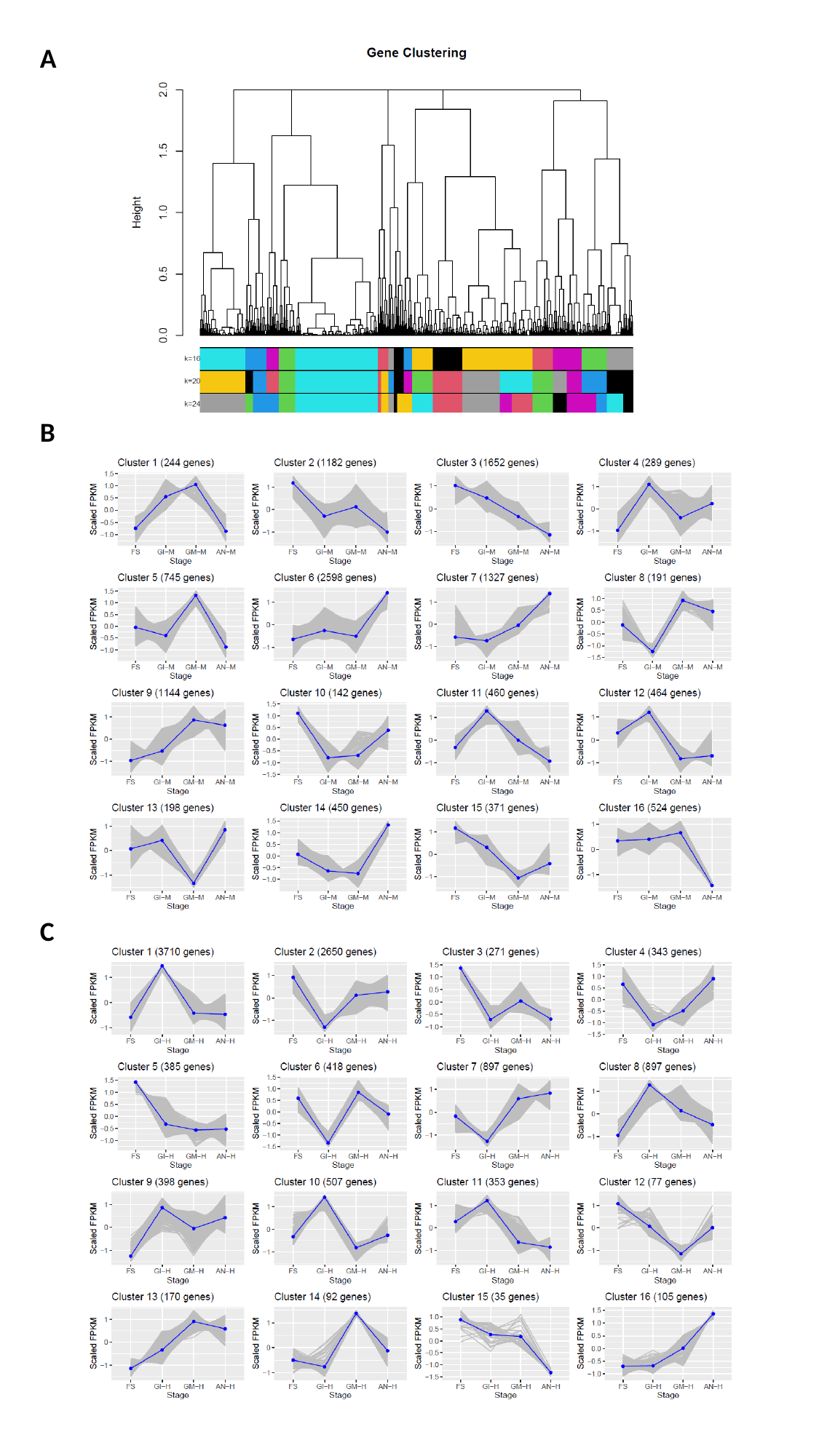

A
B
C

## Slide 3
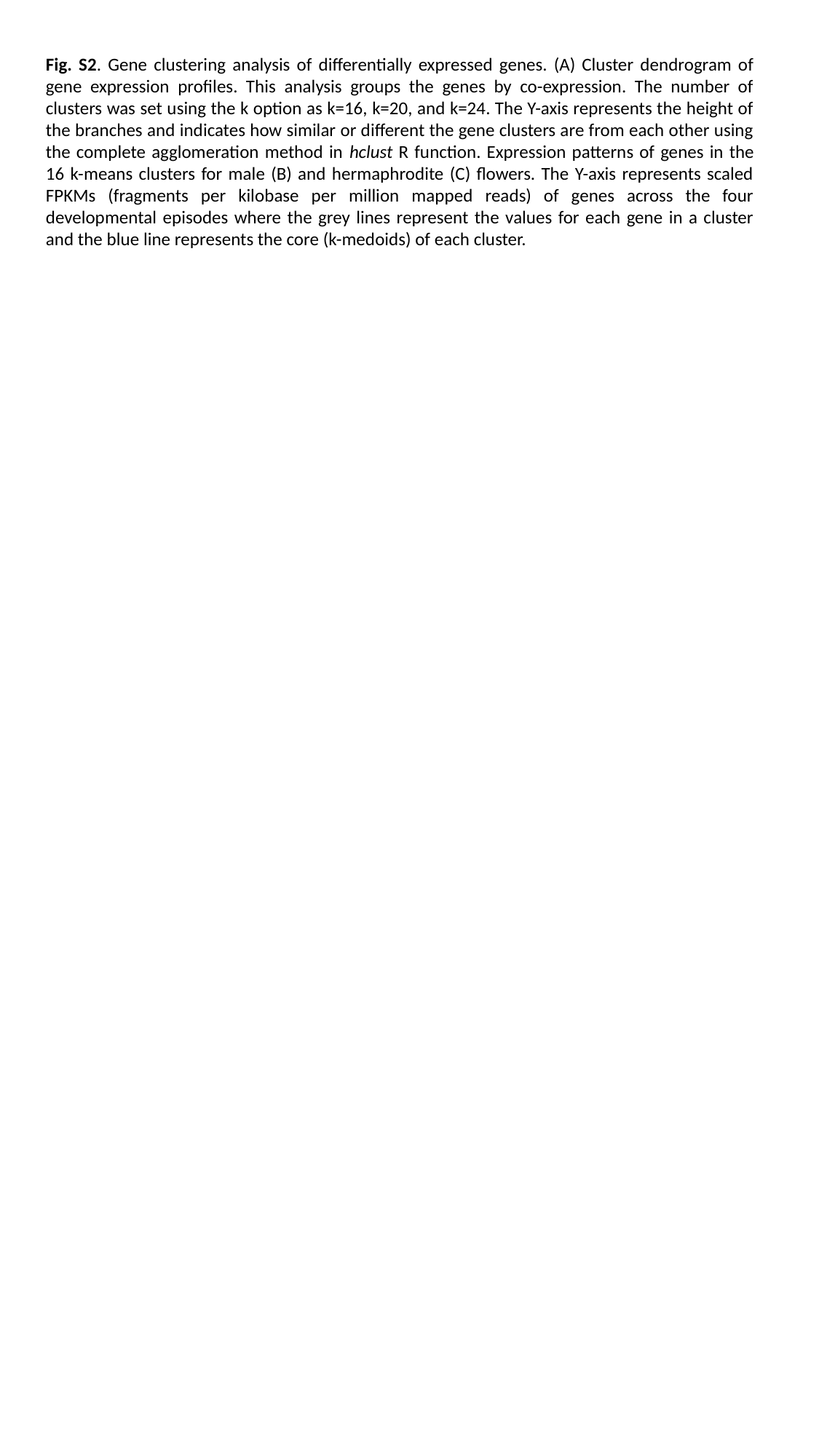

Fig. S2. Gene clustering analysis of differentially expressed genes. (A) Cluster dendrogram of gene expression profiles. This analysis groups the genes by co-expression. The number of clusters was set using the k option as k=16, k=20, and k=24. The Y-axis represents the height of the branches and indicates how similar or different the gene clusters are from each other using the complete agglomeration method in hclust R function. Expression patterns of genes in the 16 k-means clusters for male (B) and hermaphrodite (C) flowers. The Y-axis represents scaled FPKMs (fragments per kilobase per million mapped reads) of genes across the four developmental episodes where the grey lines represent the values for each gene in a cluster and the blue line represents the core (k-medoids) of each cluster.
