# Supplementary material for: Developmental stages and episode-specific regulatory genes in andromonoecious melon flower development
Source: Ann Bot. 2023 Dec 2;133(2):305–20. doi: 10.1093/aob/mcad186 (PMC11005788; doi:10.1093/aob/mcad186)
Supplement: mcad186_suppl_Supplementary_Table_S1 [file mcad186_suppl_supplementary_table_s1.pdf]

Table S1. qRT-PCR primers used in this study

Experiment	Primer ID	Primer sequence (5'-3’)
**MADS-box genes** (Fig. 4B)	*CmMADS40_F_qPCR*	CACACGAAATCCTCCACCCA
	CmMADS40_R_qPCR	TTTGTTCAACTCTTCGCCGC
	CmMADS66_F_qPCR	GAGAGCGTTGGAGGGCAATA
	CmMADS66_R _qPCR	TCTGAATCCATAAGCGCCCC
	CmMADS83_F_qPCR	CAGTTGGAAGAAGGCAGTTTCA
	CmMADS83_R_qPCR	GCGGTCGGGAAATCTTTGC
**Episode-specific genes** (Fig. 5C)	*CmERF109_F_qPCR*	GTTCTTCCAGTACCGACGCA
	CmERF109_R_qPCR	TCTTCTCGACGTTGACCTGC
	CmSKP1_F_qPCR	CAAGGTGAGTGAGGAGGTCG
	CmSKP1_R_qPCR	CTCGATCACCATTCCCAGCA
	CmCSLC4_F_qPCR	TGTTCGTACCAGAAGCCGAG
	CmCSLC4_R_qPCR	GGTGACGGACATGGTGTTCT
	CmNFD4_F_qPCR	CCTACGACCAAACCACCCTC
	CmNFD4_R_qPCR	AGGTTCATGACGACGCCAAT
	CmKPL_F_qPCR	CAGTAGGATCCAAGGCCACC
	CmKPL_R_qPCR	GCCCCATTACCATGTGACCA
	CmSWT_F_qPCR	TGCCAGAAAGCCATTGTTGC
	CmSWT_R_qPCR	AGGGGTTGGGTTTGGGAATC
**Sex-determining genes** (Fig. 6B)	*CmAcs7_F_qPCR*	TCCAACGCTCCACAATCGAA
	CmAcs7_R_qPCR	TGCGGGATTCCAAAACCTCA
	CmAcs11_F_qPCR	AACTGTCATGGACCCGAACC
	CmAcs11_R_qPCR	TTTTGGTAGCCGCATCCACT
	CmWip1_F_qPCR	TTCGTCTCCTCCTCTCCGAG
	CmWip1_R_qPCR	TCACTGACTCCTCCTCCTCG
**Referece gene**	*CmActin2_F_qPCR*	ATTCTTGCATCTCTAAGTACCTTCC
	CmActin2_R _qPCR	CCAACTAAAGGGAAATAACTCACC
