# Supplementary material for: Developmental stages and episode-specific regulatory genes in andromonoecious melon flower development
Source: Ann Bot. 2023 Dec 2;133(2):305–20. doi: 10.1093/aob/mcad186 (PMC11005788; doi:10.1093/aob/mcad186)
Supplement: mcad186_suppl_Supplementary_Table_S2 [file mcad186_suppl_supplementary_table_s2.pdf]

Table S2. FPKM values of MADS-box transcription factor genes

FPKM							ID	Gene	Gene_description	Subfamily
FS	GI-M	GM-M	AN-M	GI-H	GM-H	AN-H				
NA	2.971	5.015	3.656	31.705	NA	1.716	MELO3C000260.2	*CmMADS47*	MADS box transcription factor AGAMOUS	AG
2.119	3.543	1.924	1.369	5.940	NA	1.393	MELO3C001111.2	*CmMADS48*	MADS-box protein	Mdelta
0.180	0.089	0.049	0.016	2.130	0.123	0.049	MELO3C018601.2	*CmMADS36*	MADS box protein	SVP
NA	0.171	0.716	NA	1.559	0.077	NA	MELO3C011410.2	*CmMADS55*	MADS-box transcription factor	Mdelta
0.275	0.085	0.286	0.087	1.092	NA	0.039	MELO3C011409.2	*CmMADS15*	MADS box transcription factor	AP1/FUL
2.544	3.965	2.680	3.641	6.451	4.670	2.708	MELO3C011103.2	*CmMADS65*	MADS box interactor-like	Unclassified
0.756	0.232	NA	NA	1.585	0.401	0.769	MELO3C011093.2	*CmMADS31*	MADS box protein	SVP
1.860	2.139	4.918	3.240	7.213	1.174	0.837	MELO3C003778.2	*CmMADS16*	MADS-box transcription factor	AP3/PI
NA	0.757	1.205	NA	0.472	0.629	1.368	MELO3C003801.2	*CmMADS38*	agamous-like MADS-box protein AGL62	AGL62
28.824	34.910	3.129	NA	346.757	4.051	11.578	MELO3C022991.2	*CmMADS83*	MADS-box protein AGL42-like isoform X1	AGL42
0.492	0.304	0.260	NA	1.461	0.262	0.323	MELO3C006159.2	*CmMADS22*	MADS-box protein	SCO1
4.735	8.978	6.248	13.503	12.572	5.325	4.109	MELO3C006940.2	*CmMADS64*	MADS-box protein SOC1	$MIKC^c$
0.880	0.718	2.720	1.765	10.659	0.387	0.432	MELO3C010515.2	*CmMADS17*	MADS-box transcription factor	AP3/PI
1.406	1.860	2.064	3.713	12.939	1.984	1.380	MELO3C026300.2	*CmMADS27*	MADS-box transcription factor	SEP
0.533	0.451	0.336	NA	1.874	0.176	0.189	MELO3C026299.2	*CmMADS60*	MADS-box transcription factor	Mdelta
NA	NA	NA	30.167	0.104	NA	NA	MELO3C018030.2	*CmMADS40*	agamous-like MADS-box protein AGL62	AGL62
NA	NA	NA	1.288	0.048	NA	NA	MELO3C007017.2	*CmMADS53*	Transcription factor, MADS-box	Mdelta
0.847	3.070	2.259	1.350	13.494	2.800	2.101	MELO3C007181.2	*CmMADS03*	MADS box transcription factor AGAMOUS	AG
2.038	0.766	0.208	0.213	2.586	0.482	0.292	MELO3C007716.2	*CmMADS29*	MADS-box protein SVP-like	SVP
NA	NA	NA	NA	12.688	0.961	0.845	MELO3C033521.2	*CmMADS05*	AGAMOUS MADS box factor transcription factor	AG
0.162	0.079	NA	NA	4.712	NA	0.144	MELO3C005393.2	*CmMADS21*	MADS-box protein AGL42-like	AGL42
NA	0.507	1.853	0.780	13.878	2.144	0.431	MELO3C019694.2	*CmMADS66*	AGAMOUS MADS box factor transcription factor	AG
0.826	3.724	4.948	8.857	12.006	2.207	2.913	MELO3C022316.2	*CmMADS25*	MADS-box transcription factor	SEP
0.105	0.139	NA	0.242	1.072	NA	0.096	MELO3C022515.2	*CmMADS59*	MADS-box protein SOC1-like	$MIKC^c$
2.046	3.421	3.061	1.854	10.763	1.825	0.850	MELO3C022516.2	*CmMADS07*	MADS-box transcription factor	AGL6
NA	NA	NA	NA	16.454	0.861	0.611	MELO3C002691.2	*CmMADS01*	MADS box transcription factor AGAMOUS	AG
0.351	0.605	0.770	0.143	2.935	NA	0.095	MELO3C002369.2	*CmMADS63*	MADS-box family transcription factor	Unclassified
1.762	2.725	NA	2.365	16.797	1.313	1.096	MELO3C002050.2	*CmMADS14*	MADS box transcription factor	AP1/FUL
0.349	0.807	0.451	1.709	1.619	0.118	0.109	MELO3C002049.2	*CmMADS24*	MADS-box transcription factor	SEP
1.995	0.887	1.810	2.064	2.068	2.226	1.684	MELO3C001991.2	*CmMADS49*	Mads box protein, putative	Mdelta
