# Supplementary material for: Developmental stages and episode-specific regulatory genes in andromonoecious melon flower development
Source: Ann Bot. 2023 Dec 2;133(2):305–20. doi: 10.1093/aob/mcad186 (PMC11005788; doi:10.1093/aob/mcad186)
Supplement: mcad186_suppl_Supplementary_Table_S3 [file mcad186_suppl_supplementary_table_s3.pdf]

Table S3. FPKM values of episode-specific genes

Gene ID	FPKM							Gene Description	Specific in episode
	FS	GI-M	GM-M	AN-M	GI-H	GM-H	AN-H		
MELO3C027448.2	631.733	119.579	15.615	173.696	234.778	197.224	91.659	ORF64c	FS
MELO3C028567.2	21.893	5.142	1.092	8.084	9.850	5.891	5.058	Unknown protein	FS
MELO3C029053.2	76.968	7.498	0.602	3.381	3.867	9.120	0.613	thioredoxin F-type, chloroplastic-like	FS
MELO3C012610.2	4.113	0.741	1.249	0.346	1.891	0.606	1.125	Peroxidase	FS
MELO3C015932.2	52.181	1.589	4.057	1.802	0.580	0.908	0.599	Beta-D-xylosidase family protein	FS
MELO3C015329.2	5.548	2.232	1.777	0.972	2.024	1.174	1.917	amidase 1-like	FS
MELO3C015515.2	23.543	1.472	4.237	8.268	7.867	2.176	3.899	auxin-binding protein ABP19a-like	FS
MELO3C017096.2	49.782	14.497	5.322	9.331	10.720	9.115	8.401	Protein EXECUTER 1, chloroplastic	FS
MELO3C026269.2	10.414	2.323	1.226	2.650	3.393	0.867	1.878	LOB domain-containing protein 25	FS
MELO3C026157.2	10.786	1.204	0.804	0.454	0.739	0.432	0.715	floricaula/leafy homolog	FS
MELO3C029816.2	27.896	5.566	1.154	8.388	10.380	10.428	2.127	ORF64c	FS
MELO3C011550.2	16.241	5.472	3.213	4.203	5.710	0.673	4.999	transcription factor HBP-1b(C38) isoform X2	FS
MELO3C019777.2	1.803	0.846	0.184	0.407	0.698	0.329	0.717	U4/U6 small nuclear ribonucleoprotein PRP4-like protein	FS
MELO3C011413.2	13.777	3.454	4.655	0.401	4.795	0.144	1.431	cytochrome P450 78A5	FS
MELO3C010994.2	6.256	2.130	2.866	2.472	2.826	1.111	2.494	Galactose oxidase, putative	FS
MELO3C003370.2	2.553	0.713	0.764	0.195	1.018	0.037	0.991	dynamin-related protein 1E-like	FS
MELO3C003732.2	36.100	6.374	7.374	2.475	5.378	1.870	4.236	Lactoylglutathione lyase / glyoxalase I family protein	FS
MELO3C014712.2	8.682	1.812	2.723	2.487	3.379	1.085	2.473	Geranylgeranyl reductase	FS
MELO3C014542.2	13.425	3.645	2.824	3.716	3.665	5.164	2.593	ARM repeat superfamily protein	FS
MELO3C004585.2	7.508	0.281	0.236	1.105	0.832	1.646	0.460	RING-H2 finger protein ATL5-like	FS
MELO3C005966.2	8.113	2.927	2.581	0.723	1.487	1.556	1.390	Squamosa promoter-binding-like protein	FS
MELO3C006743.2	17.925	4.874	5.881	3.878	6.805	1.282	6.940	Mitochondrial carrier protein	FS
MELO3C006856.2	1.086	0.371	0.431	0.377	0.342	0.242	0.237	Kinase superfamily protein isoform 1	FS
MELO3C013840.2	17.022	5.879	4.527	5.062	7.429	6.896	7.176	inositol-tetrakisphosphate 1-kinase 1-like	FS
MELO3C016324.2	2.800	1.223	0.910	0.915	1.096	1.122	1.264	Cation/H(+) antiporter	FS
MELO3C016409.2	48.216	23.251	5.999	15.110	6.820	22.637	6.668	Beta-galactosidase	FS
MELO3C017906.2	8.847	1.693	2.124	1.936	3.964	3.461	3.977	acyl-protein thioesterase 2	FS
MELO3C018017.2	2.919	1.236	0.421	0.275	0.401	1.114	0.664	Nucleobase-ascorbate transporter-like protein	FS
MELO3C007251.2	52.778	24.967	9.499	19.135	24.217	7.085	24.143	Auxin response factor	FS
MELO3C005132.2	3.592	1.560	0.518	0.836	0.763	0.347	0.976	cation/H(+) antiporter 4-like	FS

Gene ID	FPKM							Gene Description	Specific in episode
	FS	GI-M	GM-M	AN-M	GI-H	GM-H	AN-H		
MELO3C011994.2	4.235	1.162	1.773	0.577	1.322	0.380	1.731	BHLH transcription factor	FS
MELO3C023854.2	17.557	8.303	1.125	5.838	7.063	1.530	5.612	CASP-like protein	FS
MELO3C034418.2	39.234	9.571	0.443	7.232	5.231	6.296	3.424	Unknown protein	FS
MELO3C022917.2	5.496	2.065	2.227	2.086	2.035	1.150	1.652	Respiratory burst oxidase, putative	FS
MELO3C020975.2	145.067	32.494	17.663	8.079	21.749	3.441	18.562	cytochrome P450 78A7	FS
MELO3C020810.2	237.255	91.079	44.456	112.699	98.194	38.278	91.914	Subtilisin-like protease	FS
MELO3C024739.2	10.730	3.925	3.590	0.744	5.282	0.184	5.182	growth-regulating factor 4-like	FS
MELO3C013473.2	208.823	10.676	10.167	2.890	9.041	6.674	1.664	tetraketide alpha-pyrone reductase 1	FS
MELO3C021259.2	408.888	159.733	38.262	20.643	20.541	0.718	6.458	Peroxidase	FS
MELO3C035217.2	1.378	0.083	0.241	0.639	0.122	0.424	0.290	Unknown protein	FS
MELO3C002567.2	1.407	0.470	0.410	0.676	0.627	0.063	0.664	Unknown protein	FS
MELO3C027553.2	18.090	6.922	3.833	5.613	7.151	4.212	4.844	kinesin-like protein KIN12B	FS
MELO3C028652.2	3.029	1.215	1.288	0.687	0.695	0.271	1.085	Protein DETOXIFICATION	FS
MELO3C015838.2	7.101	2.537	0.944	2.083	1.260	2.269	0.375	Protein FANTASTIC FOUR 2	FS
MELO3C030845.2	1.305	0.634	0.427	0.281	0.298	0.236	0.368	Unknown protein	FS
MELO3C014517.2	3.882	1.635	1.816	1.249	1.839	1.504	0.808	Late embryogenesis abundant protein	FS
MELO3C006152.2	33.524	14.677	13.123	2.437	15.499	0.680	3.945	At4g00770	FS
MELO3C019506.2	104.568	51.638	44.742	11.284	42.527	5.298	10.881	ethylene-responsive transcription factor ERF109	FS
MELO3C019451.2	5.746	2.223	1.243	1.530	2.531	1.283	1.582	Unknown protein	FS
MELO3C027162.2	2.133	0.695	0.095	0.789	0.862	0.791	0.119	Serine/threonine-protein kinase	FS
MELO3C027169.2	0.442	4.080	2.603	1.925	1.699	1.118	1.891	Lipoxygenase	GI-M
MELO3C000768.2	2.164	11.306	6.598	3.070	3.439	3.430	3.899	Unknown protein	GI-M
MELO3C018426.2	6.208	16.349	38.080	3.107	12.108	1.540	16.143	AT3g19030/K13E13_15	GI-M
MELO3C018882.2	6.607	70.957	53.570	2.803	3.350	33.863	2.917	Unknown protein	GI-M
MELO3C023665.2	0.878	2.742	1.554	0.626	1.955	0.275	1.252	leucine-rich repeat receptor-like tyrosine-protein kinase PXC3	GI-M
MELO3C024087.2	0.634	1.858	2.003	0.651	1.330	0.654	0.570	G-box-binding factor 4-like isoform X1	GI-M
MELO3C015699.2	2.353	11.430	0.971	1.369	2.879	0.330	1.955	exopolygalacturonase isoform X1	GI-M
MELO3C015711.2	10.124	124.852	5.015	49.078	14.763	15.422	9.513	Carboxypeptidase	GI-M
MELO3C024423.2	0.618	1.443	2.326	0.495	0.413	0.302	0.567	Receptor-like protein kinase	GI-M
MELO3C015490.2	0.458	1.688	2.580	0.326	0.431	0.293	0.684	Calmodulin-binding family protein	GI-M
MELO3C015540.2	1.572	16.910	0.813	0.859	0.682	0.192	0.253	Beta-galactosidase	GI-M

Gene ID	FPKM							Gene Description	Specific in episode
	FS	GI-M	GM-M	AN-M	GI-H	GM-H	AN-H		
MELO3C015651.2	3.999	14.372	1.340	6.188	6.469	1.374	5.038	Metal tolerance protein	GI-M
MELO3C010172.2	0.371	1.390	1.273	0.405	3.229	0.230	2.609	Glutamate receptor	GI-M
MELO3C010317.2	2.777	9.780	0.899	1.528	1.531	1.400	0.584	Auxin-responsive protein	GI-M
MELO3C025311.2	2.853	11.008	8.888	2.781	34.648	1.630	0.936	omega-hydroxypalmitate O-feruloyl transferase	GI-M
MELO3C024633.2	6.062	12.697	13.236	4.918	17.677	1.305	13.728	Protein ABIL1	GI-M
MELO3C024691.2	38.042	237.928	17.423	77.644	54.769	15.514	32.225	Peroxiredoxin	GI-M
MELO3C017356.2	6.382	19.389	107.835	2.694	265.260	0.365	323.505	Phosphoethanolamine n-methyltransferase, putative	GI-M
MELO3C017297.2	3.762	9.068	1.300	3.270	3.100	2.740	3.194	protein ASPARTIC PROTEASE IN GUARD CELL 1-like	GI-M
MELO3C026248.2	1.114	45.154	18.100	0.775	22.151	1.483	0.973	E3 ubiquitin-protein ligase RGLG2	GI-M
MELO3C030167.2	0.609	38.073	0.416	10.220	3.299	0.307	2.161	Flavonol synthase	GI-M
MELO3C019887.2	1.050	2.426	6.511	1.091	1.948	0.987	1.137	Protein BUD31-like protein 1	GI-M
MELO3C011369.2	1.617	103.141	1.752	10.213	12.570	0.981	7.625	Hexosyltransferase	GI-M
MELO3C011177.2	1.674	5.059	6.684	1.944	2.891	1.282	3.155	Protein disulfide isomerase (PDI)-like protein	GI-M
MELO3C011106.2	0.519	1.715	6.964	0.685	1.589	0.642	1.758	Pentatricopeptide repeat-containing protein	GI-M
MELO3C003592.2	20.214	103.960	8.958	40.016	26.754	9.201	14.220	BURP domain protein RD22	GI-M
MELO3C012936.2	2.656	15.775	1.601	1.109	4.918	1.242	2.013	boron transporter 4-like	GI-M
MELO3C012993.2	1.088	4.378	1.418	1.504	1.163	1.286	0.983	biotin--protein ligase 2-like	GI-M
MELO3C009920.2	0.514	1.418	0.275	0.279	0.693	0.508	0.708	protein NRT1/ PTR FAMILY 5.8-like	GI-M
MELO3C009826.2	0.741	4.517	3.313	0.701	0.696	0.986	0.355	Carboxypeptidase	GI-M
MELO3C009739.2	0.503	5.335	1.010	1.548	0.636	1.968	0.156	WAT1-related protein	GI-M
MELO3C030737.2	3.779	7.738	11.022	3.453	9.246	2.458	4.349	Glutathione S-transferase	GI-M
MELO3C009179.2	0.548	1.643	1.367	0.765	0.806	0.211	0.496	Receptor-kinase, putative	GI-M
MELO3C014656.2	2.158	16.640	35.913	3.790	3.797	6.062	6.120	Peroxidase	GI-M
MELO3C014437.2	5.735	15.325	28.407	3.272	6.601	2.911	4.312	1-aminocyclopropane-1-carboxylate oxidase 1	GI-M
MELO3C031318.2	3.863	22.937	47.525	10.077	4.308	0.937	3.531	Lipoxygenase	GI-M
MELO3C004249.2	0.422	4.148	4.379	1.187	0.762	0.151	0.564	Lipoxygenase	GI-M
MELO3C004449.2	0.692	2.049	6.999	0.435	3.567	0.502	3.072	leucine-rich repeat receptor-like protein kinase TDR	GI-M
MELO3C006027.2	0.235	119.669	1.740	19.615	8.710	0.110	4.662	RING-H2 finger protein ATL3K, putative	GI-M
MELO3C006252.2	6.180	27.905	2.387	8.186	2.823	1.003	2.426	protein ECERIFERUM 1-like	GI-M
MELO3C006934.2	3.032	6.809	2.122	2.498	3.642	1.409	1.912	protein REVEILLE 6-like isoform X2	GI-M
MELO3C016574.2	1.023	2.672	2.564	0.348	1.221	0.511	1.213	Serine/threonine-protein kinase	GI-M

Gene ID	FPKM							Gene Description	Specific in episode
	FS	GI-M	GM-M	AN-M	GI-H	GM-H	AN-H		
MELO3C014128.2	0.387	1.539	5.145	0.544	0.505	0.594	0.306	Calcium-dependent protein kinase	GI-M
MELO3C032305.2	2.720	6.822	0.964	2.468	2.394	1.546	1.021	Unknown protein	GI-M
MELO3C016856.2	0.639	1.468	4.948	0.711	2.250	0.694	1.317	homeobox protein 4-like	GI-M
MELO3C016854.2	1.116	5.683	1.356	2.188	1.443	0.811	0.624	Core-2/I-branching beta-1,6-N-acetylglucosaminyltransferase family protein	GI-M
MELO3C016761.2	0.259	1.780	1.142	0.808	1.006	0.219	0.452	Lipase	GI-M
MELO3C016719.2	6.997	14.788	5.364	5.712	8.767	5.908	8.395	Cytochrome P450	GI-M
MELO3C016667.2	0.630	2.507	2.776	1.243	1.187	0.667	0.908	Random slug protein 5	GI-M
MELO3C026378.2	2.107	11.322	1.707	3.654	2.595	5.536	4.603	zinc transporter 5-like	GI-M
MELO3C010532.2	5.783	15.017	3.055	1.729	2.083	7.153	3.504	Structural constituent of ribosome, putative	GI-M
MELO3C016135.2	25.726	69.411	53.024	6.209	10.331	13.378	12.348	Serine/threonine-protein kinase	GI-M
MELO3C017754.2	5.529	17.420	24.387	7.008	4.156	6.893	4.426	NAC domain-containing protein	GI-M
MELO3C017811.2	2.923	16.791	1.585	3.665	1.349	4.907	2.755	phenylalanine ammonia-lyase-like	GI-M
MELO3C018051.2	2.701	13.497	1.022	3.592	2.262	2.636	2.231	Gamma-glutamyl peptidase 5	GI-M
MELO3C007150.2	4.030	74.909	3.757	4.607	7.751	1.849	4.949	Beta-D-xylosidase	GI-M
MELO3C007173.2	0.331	1.383	0.494	0.472	0.091	0.670	0.070	ABC transporter B family protein	GI-M
MELO3C007399.2	0.585	1.752	4.411	0.370	2.034	0.324	1.587	Rhamnogalacturonate lyase	GI-M
MELO3C007613.2	0.331	2.992	1.997	1.483	0.818	1.235	1.057	acyl-CoA-binding domain-containing protein 3	GI-M
MELO3C007919.2	2.607	12.142	4.985	2.916	7.975	0.553	7.320	alpha-mannosidase	GI-M
MELO3C008988.2	0.759	8.890	16.910	0.208	5.033	0.369	0.118	Fatty acyl-CoA reductase	GI-M
MELO3C014223.2	1.647	6.497	1.026	1.192	1.299	1.233	1.005	phenylalanine ammonia-lyase-like	GI-M
MELO3C003186.2	1.205	5.057	2.993	1.924	0.839	2.293	0.564	Proteasome inhibitor-related	GI-M
MELO3C022107.2	0.332	4.864	0.225	1.540	1.011	0.454	0.324	Methylesterase 17	GI-M
MELO3C022098.2	0.512	1.827	0.252	0.707	0.829	0.313	0.383	Cytochrome P450, putative	GI-M
MELO3C021550.2	5.929	14.847	3.810	2.100	4.988	4.587	3.942	cinnamoyl-CoA reductase 1-like	GI-M
MELO3C021552.2	2.435	5.480	9.932	2.428	4.501	1.288	3.525	universal stress protein A-like protein	GI-M
MELO3C005209.2	1.214	3.402	4.085	0.641	1.634	0.504	1.227	Phytosulfokine receptor, putative	GI-M
MELO3C005631.2	0.163	22.870	0.113	0.721	1.145	0.107	0.188	sugar carrier protein C-like	GI-M
MELO3C012407.2	2.089	8.243	7.395	2.359	4.772	2.389	2.002	SKP1-like protein 12	GI-M
MELO3C012027.2	2.897	6.408	1.609	3.165	1.676	0.488	1.727	IAA-amino acid hydrolase ILR1, putative	GI-M
MELO3C011885.2	15.642	31.520	9.841	12.878	11.667	4.218	8.335	Lipoxygenase	GI-M

Gene ID	FPKM							Gene Description	Specific in episode
	FS	GI-M	GM-M	AN-M	GI-H	GM-H	AN-H		
MELO3C026824.2	13.879	36.939	17.068	15.442	3.362	3.342	9.665	cytochrome P450 714A1-like	GI-M
MELO3C019206.2	12.585	25.977	4.589	11.297	7.623	8.876	9.079	cytochrome P450 81E8-like	GI-M
MELO3C019363.2	19.828	107.797	4.719	51.473	11.105	11.216	11.258	patellin-1-like	GI-M
MELO3C013505.2	0.139	2.530	0.064	1.029	0.288	0.746	0.066	isocitrate dehydrogenase [NAD] regulatory subunit 1, mitochondrial-like	GI-M
MELO3C013538.2	11.839	29.187	4.305	3.682	37.663	1.785	37.245	AT4g28240/F26K10_120	GI-M
MELO3C035066.2	0.280	1.535	1.220	0.517	0.969	0.580	0.293	5'-nucleotidase domain-containing protein	GI-M
MELO3C025880.2	1.378	12.527	2.705	4.494	3.416	2.780	2.356	Neutral ceramidase	GI-M
MELO3C025712.2	0.225	1.353	2.570	0.337	0.275	0.167	0.389	Respiratory burst oxidase-like protein	GI-M
MELO3C025780.2	4.958	16.730	7.169	5.428	7.345	0.978	5.428	Cytokinin riboside 5'-monophosphate phosphoribohydrolase	GI-M
MELO3C021379.2	2.663	5.830	10.078	0.557	4.191	0.527	2.867	Histidine-containing phosphotransfer protein, putative	GI-M
MELO3C021256.2	1.263	24.213	31.509	1.061	7.996	1.556	0.836	Desiccation-related protein PCC13-62	GI-M
MELO3C022430.2	2.340	7.974	8.508	1.155	2.785	2.993	2.310	Amaranthin-like lectin	GI-M
MELO3C002553.2	10.530	26.766	3.028	9.155	10.210	3.154	8.820	sugar carrier protein C-like	GI-M
MELO3C002508.2	11.981	27.315	10.933	2.769	9.717	8.532	5.321	thioredoxin-like protein CXXS1	GI-M
MELO3C002350.2	3.397	8.955	2.596	0.794	0.771	2.902	1.098	Malate synthase	GI-M
MELO3C002272.2	4.614	39.110	6.264	5.707	9.719	1.956	8.441	Transmembrane protein, putative	GI-M
MELO3C002212.2	0.261	4.188	0.500	0.988	0.772	0.201	0.432	protein NRT1/ PTR FAMILY 2.8	GI-M
MELO3C011535.2	4.756	9.544	1.752	0.755	4.168	3.476	2.984	Transmembrane protein, putative	GI-M
MELO3C011263.2	1.400	3.242	4.554	0.454	1.365	0.665	0.448	EEIG1/EHBP1 protein amino-terminal domain protein	GI-M
MELO3C009530.2	6.255	12.808	2.842	0.882	NA	5.681	0.405	YLS9	GI-M
MELO3C014803.2	0.985	2.067	1.321	0.264	1.105	0.294	1.157	Phosphate carrier, mitochondrial	GI-M
MELO3C010520.2	2.620	8.051	35.469	0.141	13.187	0.987	0.239	Chalcone synthase	GI-M
MELO3C026951.2	1.634	4.418	10.208	0.615	8.990	1.019	5.408	RING finger and CHY zinc finger protein	GI-M
MELO3C020535.2	5.271	15.121	3.001	1.335	2.353	5.889	0.779	mitogen-activated protein kinase kinase kinase NPK1	GI-M
MELO3C000773.2	2.568	8.317	4.824	3.666	7.639	0.452	2.379	(S)-ureidoglycine aminohydrolase	GI-M
MELO3C011671.2	0.933	1.962	0.267	0.582	0.965	0.255	0.514	ethylene-responsive transcription factor CRF6-like	GI-M
MELO3C004202.2	3.554	7.113	5.903	1.277	3.035	0.949	2.207	protein SAR DEFICIENT 1-like isoform X1	GI-M
MELO3C004335.2	1.468	2.971	3.495	1.469	2.135	0.367	1.272	50S ribosomal protein L18	GI-M
MELO3C006028.2	0.953	2.237	5.788	0.681	2.579	0.057	2.794	cyclic nucleotide-gated ion channel 1	GI-M
MELO3C026404.2	4.455	9.161	6.277	2.626	9.400	0.191	13.579	gamma aminobutyrate transaminase 1, mitochondrial	GI-M

Gene ID	FPKM							Gene Description	Specific in episode
	FS	GI-M	GM-M	AN-M	GI-H	GM-H	AN-H		
MELO3C032563.2	3.061	7.054	18.033	2.196	2.242	0.908	1.439	Glutathione S-transferase	GI-M
MELO3C003015.2	4.251	10.320	7.184	4.506	2.053	1.186	1.538	Allene oxide cyclase	GI-M
MELO3C018475.2	4.060	10.614	7.012	2.157	1.960	3.200	1.790	Elicitor-responsive protein 3	GI-M
MELO3C018777.2	1.501	3.280	7.239	1.102	3.186	0.881	1.875	Serine/threonine-protein kinase	GI-M
MELO3C026238.2	1.469	4.686	1.551	1.063	0.444	1.012	0.942	peptide methionine sulfoxide reductase-like	GI-M
MELO3C009681.2	8.461	32.442	9.428	2.043	3.278	2.270	1.428	At3g57450	GI-M
MELO3C009172.2	2.809	6.474	8.989	1.889	3.113	2.413	2.268	RING finger protein	GI-M
MELO3C004609.2	1.259	2.850	1.070	1.057	1.448	1.254	1.322	inositol oxygenase 2	GI-M
MELO3C007317.2	13.048	32.309	25.049	5.853	7.268	8.377	4.337	Transmembrane protein, putative	GI-M
MELO3C011818.2	2.809	5.960	8.428	1.556	2.800	2.645	2.112	C2 domain-containing family protein	GI-M
MELO3C023228.2	0.528	1.539	2.908	0.357	1.030	0.480	1.024	U2 small nuclear ribonucleoprotein auxiliary factor-like protein	GI-M
MELO3C019590.2	1.760	4.328	1.385	1.656	0.943	2.155	2.059	ethylene-responsive transcription factor ERF069-like	GI-M
MELO3C021412.2	0.760	1.739	1.113	0.497	0.764	0.418	1.120	Glucose-1-phosphate adenylyltransferase	GI-M
MELO3C024895.2	0.808	1.718	1.869	0.647	0.395	0.672	0.833	Cytokinin riboside 5'-monophosphate phosphoribohydrolase LOG	GI-M
MELO3C015497.2	0.582	1.224	13.922	0.581	1.994	0.514	1.557	LOW QUALITY PROTEIN: mitogen-activated protein kinase kinase 2-like	GI-M
MELO3C009420.2	2.709	6.278	2.360	3.021	8.459	2.007	6.815	Homeobox leucine zipper protein	GI-M
MELO3C009165.2	0.768	2.466	2.351	0.866	1.813	0.626	1.945	dnaJ homolog subfamily B member 4	GI-M
MELO3C006818.2	2.178	4.834	8.722	2.395	6.201	2.000	3.369	protein jagunal homolog 1-like	GI-M
MELO3C019002.2	0.765	1.987	11.458	0.860	3.415	0.621	2.083	Annexin	GI-M
MELO3C017945.2	1.304	2.918	0.870	1.432	0.725	0.675	0.597	bifunctional epoxide hydrolase 2-like	GI-M
MELO3C022161.2	0.461	1.006	3.522	0.480	0.975	0.362	0.894	pentatricopeptide repeat-containing protein At3g13880	GI-M
MELO3C005321.2	5.420	18.159	6.186	7.231	1.755	4.472	0.374	DnaJ-like protein	GI-M
MELO3C012279.2	1.774	5.533	1.488	2.225	2.813	1.670	1.528	Lipid phosphate phosphatase 2	GI-M
MELO3C018366.2	1.608	4.171	0.614	1.653	1.099	0.781	0.679	RING-H2 finger protein ATL66	GI-M
MELO3C018356.2	1.074	3.214	9.270	1.323	0.859	0.691	0.905	Protein WVD2-like 7	GI-M
MELO3C021262.2	1.411	4.857	2.244	1.499	NA	0.907	0.942	Unknown protein	GI-M
MELO3C027134.2	4.907	13.857	0.944	35.499	1.820	5.263	2.125	Sucrose transporter	GM-M
MELO3C018590.2	0.693	2.137	1.665	7.685	1.402	3.477	1.206	root phototropism protein 3	GM-M
MELO3C018672.2	0.281	1.149	0.086	4.762	0.070	0.866	0.092	Peroxidase	GM-M
MELO3C018888.2	9.140	4.608	3.593	23.772	10.488	4.074	6.118	Fasciclin-like arabinogalactan protein 7	GM-M

Gene ID	FPKM							Gene Description	Specific in episode
	FS	GI-M	GM-M	AN-M	GI-H	GM-H	AN-H		
MELO3C024190.2	37.612	71.128	10.511	152.978	53.567	31.465	39.882	Acetyl-CoA acetyltransferase	GM-M
MELO3C015831.2	13.305	6.810	3.190	33.861	15.729	1.516	18.823	Patatin	GM-M
MELO3C015962.2	3.387	10.133	1.051	31.817	5.849	7.492	3.355	Pectinesterase	GM-M
MELO3C015963.2	8.896	4.107	3.717	29.158	7.765	3.724	7.729	pectinesterase-like	GM-M
MELO3C029317.2	17.714	29.676	18.964	70.730	23.294	3.988	17.111	L-ascorbate oxidase	GM-M
MELO3C029352.2	8.172	17.975	1.196	49.752	5.118	4.494	4.159	non-specific phospholipase C2	GM-M
MELO3C015098.2	31.832	20.514	7.586	124.683	33.666	20.063	26.841	Tubulin beta chain	GM-M
MELO3C010623.2	2.967	8.231	0.917	22.718	3.041	4.211	3.330	IAA-amino acid hydrolase ILR1, putative	GM-M
MELO3C026503.2	1.344	2.531	4.005	19.632	1.153	0.453	2.137	transcription factor bHLH79	GM-M
MELO3C026554.2	20.575	36.328	9.042	135.056	22.424	33.972	26.520	alcohol dehydrogenase-like 7	GM-M
MELO3C010662.2	37.613	66.914	17.166	186.771	36.596	68.293	41.056	Polygalacturonase-1 non-catalytic subunit beta	GM-M
MELO3C013026.2	8.699	14.451	3.282	39.473	9.198	16.111	8.130	IAA-amino acid hydrolase ILR1, putative	GM-M
MELO3C022681.2	1.030	3.563	1.338	8.555	2.741	2.658	2.293	Peroxisomal (S)-2-hydroxy-acid oxidase	GM-M
MELO3C023926.2	0.401	1.028	0.112	2.590	0.503	1.285	0.490	DUF946 family protein (DUF946)	GM-M
MELO3C009924.2	9.880	5.799	7.074	24.111	10.285	8.279	11.560	peroxidase 31	GM-M
MELO3C009872.2	504.589	268.491	229.163	1582.147	514.458	366.212	481.359	Gibberellin-regulated family protein	GM-M
MELO3C009782.2	380.744	204.156	62.035	1492.136	335.032	507.897	274.469	5-methyltetrahydropteroyltriglutamate-- homocysteine methyltransferase	GM-M
MELO3C014682.2	0.333	0.810	0.698	3.278	0.640	0.790	0.966	Acetyl-coenzyme A synthetase	GM-M
MELO3C004338.2	1.143	5.595	0.537	23.785	1.146	5.087	1.941	Acyl carrier protein	GM-M
MELO3C006851.2	29.745	14.210	7.600	69.607	28.853	6.393	28.964	Subtilisin-like protease	GM-M
MELO3C014937.2	7.522	12.522	3.137	72.357	8.404	16.458	13.519	acyltransferase-like protein At3g26840, chloroplastic	GM-M
MELO3C014857.2	0.656	3.432	2.307	10.710	0.767	5.313	0.664	Lysine histidine transporter	GM-M
MELO3C025629.2	5.336	3.060	2.518	16.480	4.939	2.192	5.647	transcription factor TCP4	GM-M
MELO3C022970.2	11.811	103.851	5.962	248.796	24.499	44.286	18.738	Aldo/keto reductase family protein	GM-M
MELO3C016297.2	25.283	43.604	8.909	173.180	18.129	75.696	13.556	Nucleoside diphosphate kinase	GM-M
MELO3C016405.2	3.973	0.982	2.233	21.942	0.693	7.561	3.305	Peroxidase	GM-M
MELO3C017566.2	1.055	6.816	0.087	13.740	1.502	1.586	1.461	Glycosyltransferase	GM-M
MELO3C007258.2	0.868	6.765	2.028	106.678	2.453	18.372	1.060	DUF679 domain membrane protein	GM-M
MELO3C007408.2	4.683	14.348	11.312	69.862	3.542	27.461	3.887	Aspartic proteinase	GM-M
MELO3C007508.2	1.380	5.898	0.371	19.308	1.184	2.029	2.937	Cold acclimation protein	GM-M

Gene ID	FPKM							Gene Description	Specific in episode
	FS	GI-M	GM-M	AN-M	GI-H	GM-H	AN-H		
MELO3C007549.2	0.206	0.722	10.820	4.025	1.042	1.331	1.221	Estradiol 17-beta-dehydrogenase 1	GM-M
MELO3C007566.2	0.225	1.188	0.853	2.533	0.671	0.642	0.940	Late embryogenesis abundant protein, LEA-14	GM-M
MELO3C007677.2	1.848	4.262	1.157	10.376	1.741	4.006	1.301	Phosphotransferase	GM-M
MELO3C007799.2	2.730	10.866	3.421	32.898	4.407	10.539	3.445	cytochrome P450 CYP736A12-like	GM-M
MELO3C024539.2	0.294	2.533	0.459	13.135	0.718	1.898	0.355	Purple acid phosphatase	GM-M
MELO3C003107.2	6.826	18.174	7.263	54.559	8.802	11.369	7.957	Soluble inorganic pyrophosphatase-like protein	GM-M
MELO3C002978.2	0.431	1.933	0.335	9.904	1.744	1.901	0.942	2-alkenal reductase (NADP(+)-dependent)-like	GM-M
MELO3C005260.2	11.456	6.939	5.647	24.689	43.130	4.064	42.684	Vacuolar iron transporter 1	GM-M
MELO3C018316.2	1.772	0.787	1.125	5.633	2.246	2.584	2.295	subtilisin-like protease SBT1.1	GM-M
MELO3C023310.2	4.696	11.402	9.903	54.250	7.175	23.945	6.446	Glucose-1-phosphate adenylyltransferase	GM-M
MELO3C020872.2	6.618	10.126	4.715	78.449	7.411	28.453	9.327	Zeaxanthin epoxidase, chloroplastic	GM-M
MELO3C020531.2	0.695	2.750	0.681	6.624	0.484	0.213	0.514	Pyruvate decarboxylase	GM-M
MELO3C002562.2	1.381	27.243	1.787	70.877	6.789	14.050	3.711	endonuclease 1	GM-M
MELO3C002447.2	9.499	3.569	3.804	21.233	7.859	2.027	7.774	L-ascorbate oxidase homolog	GM-M
MELO3C002319.2	35.579	17.305	9.191	164.455	25.537	59.094	29.515	Pectate lyase	GM-M
MELO3C002143.2	5.653	9.131	4.683	30.531	7.017	11.069	6.499	Protein TIFY 6B	GM-M
MELO3C000701.2	3.558	4.580	43.689	9.505	4.957	2.452	4.282	DNA-directed RNA polymerase subunit beta''	GM-M
MELO3C018489.2	0.174	0.393	0.173	1.503	0.435	0.598	0.347	Glycosyltransferase	GM-M
MELO3C018573.2	4.599	5.487	13.260	15.978	9.780	4.196	8.930	Inorganic pyrophosphatase	GM-M
MELO3C018657.2	10.973	7.093	7.844	31.034	8.979	11.477	12.781	non-specific lipid-transfer protein-like protein At5g64080	GM-M
MELO3C018664.2	0.244	0.195	1.141	3.531	0.981	0.376	1.072	7-deoxyloganetin glucosyltransferase-like	GM-M
MELO3C018743.2	5.743	8.159	0.768	30.755	2.808	2.945	3.063	expansin-B3-like	GM-M
MELO3C018814.2	19.061	22.385	4.423	60.027	12.028	16.193	9.667	Protein DETOXIFICATION	GM-M
MELO3C018833.2	3.939	5.040	0.894	17.655	2.993	7.171	2.242	Allene oxide synthase	GM-M
MELO3C013111.2	2.835	2.061	0.839	8.710	4.355	0.992	3.084	acyl-CoA--sterol O-acyltransferase 1	GM-M
MELO3C012714.2	0.972	0.845	1.322	2.082	1.147	0.933	1.055	monocopper oxidase-like protein SKU5 isoform X1	GM-M
MELO3C021057.2	0.524	0.977	1.008	2.009	0.654	0.640	1.049	RPM1-interacting protein 4	GM-M
MELO3C021071.2	4.577	3.869	2.480	12.648	3.174	3.132	4.374	GTP cyclohydrolase 1	GM-M
MELO3C015948.2	2.109	4.331	1.299	8.891	2.647	1.927	2.319	Flagellar biosynthesis protein flhA	GM-M
MELO3C024448.2	5.007	3.515	0.782	15.455	1.834	2.808	2.574	GATA transcription factor	GM-M
MELO3C015448.2	0.993	1.431	9.208	3.433	2.618	0.924	1.986	AT4G29520-like protein	GM-M

Gene ID	FPKM							Gene Description	Specific in episode
	FS	GI-M	GM-M	AN-M	GI-H	GM-H	AN-H		
MELO3C015549.2	2.091	2.589	2.050	14.144	4.820	1.289	7.832	21 kDa protein	GM-M
MELO3C015638.2	1.307	2.607	5.999	10.722	7.864	0.947	6.955	protein YLS3-like	GM-M
MELO3C015643.2	30.675	28.752	20.617	67.690	44.812	2.779	51.962	L-ascorbate oxidase	GM-M
MELO3C010233.2	0.626	1.123	1.171	3.106	1.097	0.578	0.866	reticulon-like protein B21	GM-M
MELO3C010312.2	15.681	10.318	38.001	68.701	23.882	15.827	43.445	36.4 kDa proline-rich protein	GM-M
MELO3C017453.2	0.906	1.089	2.488	4.287	1.800	1.925	1.392	Protein kinase family protein	GM-M
MELO3C017268.2	6.314	6.487	8.543	27.840	5.689	5.978	7.673	Enolase, putative	GM-M
MELO3C017243.2	0.396	0.543	1.242	3.651	0.851	0.338	0.619	COBRA-like protein 4	GM-M
MELO3C017154.2	2.132	1.471	1.358	4.471	1.386	1.550	2.443	protein NRT1/ PTR FAMILY 4.6-like	GM-M
MELO3C026199.2	1.017	1.480	0.566	7.009	NA	2.922	0.999	Transmembrane protein, putative	GM-M
MELO3C008078.2	7.131	5.439	2.522	20.483	6.024	9.041	6.794	glucuronoxylan 4-O-methyltransferase 3-like	GM-M
MELO3C008269.2	6.979	4.442	2.835	15.765	6.466	3.551	8.228	L-type lectin-domain containing receptor kinase S.4-like	GM-M
MELO3C010636.2	0.276	0.128	0.712	2.534	0.160	0.486	0.296	Beta-galactosidase	GM-M
MELO3C030091.2	1.500	2.004	23.444	4.676	2.823	1.055	1.961	Unknown protein	GM-M
MELO3C019951.2	1773.205	2074.207	3206.023	4157.884	2442.864	1943.093	1880.799	ATP synthase subunit alpha	GM-M
MELO3C011484.2	0.168	0.172	0.783	2.517	NA	1.099	0.187	Spermidine synthase	GM-M
MELO3C011439.2	12.221	13.108	9.669	29.531	9.206	10.613	9.983	6,7-dimethyl-8-ribityllumazine synthase	GM-M
MELO3C011338.2	3.481	3.380	0.767	27.865	3.241	7.832	2.525	hippocampus abundant transcript 1 protein-like	GM-M
MELO3C011046.2	18.697	16.075	16.363	45.954	25.225	12.108	25.962	O-fucosyltransferase family protein	GM-M
MELO3C010951.2	0.689	1.234	1.093	7.669	1.266	2.899	0.996	Isoflavone reductase like	GM-M
MELO3C010668.2	17.656	19.047	37.359	56.570	19.219	8.333	22.479	polygalacturonase At1g48100-like	GM-M
MELO3C003616.2	8.565	7.385	3.922	22.144	12.463	7.533	13.297	xyloglucan glycosyltransferase 4	GM-M
MELO3C030695.2	1.378	1.863	8.672	3.902	3.242	0.438	2.882	Plasma membrane ATPase	GM-M
MELO3C018139.2	5.259	6.927	3.911	14.233	6.261	4.106	5.111	Protein kinase family protein	GM-M
MELO3C018082.2	19.929	19.275	7.883	40.654	19.046	15.747	18.864	Tryptophan synthase	GM-M
MELO3C012838.2	10.857	11.296	3.221	42.153	4.964	10.844	5.933	leucine-rich repeat receptor-like protein kinase PXL2	GM-M
MELO3C012851.2	0.654	1.417	0.528	8.534	0.992	1.377	NA	protein NRT1/ PTR FAMILY 5.2-like	GM-M
MELO3C013048.2	0.472	0.511	0.501	2.635	0.742	0.989	0.557	Phospholipase D	GM-M
MELO3C025791.2	13.228	10.051	4.137	31.728	11.591	9.571	14.251	D-3-phosphoglycerate dehydrogenase	GM-M
MELO3C023962.2	54.884	37.520	14.726	409.191	75.000	52.256	69.242	Leucine-rich repeat receptor-like protein kinase family	GM-M
MELO3C030670.2	21.763	33.858	1.892	69.363	9.095	11.892	11.817	Unknown protein	GM-M

Gene ID	FPKM							Gene Description	Specific in episode
	FS	GI-M	GM-M	AN-M	GI-H	GM-H	AN-H		
MELO3C009722.2	16.698	22.438	5.562	62.200	9.269	11.785	9.613	Alpha-L-arabinofuranosidase 1	GM-M
MELO3C009517.2	3.301	2.212	0.568	20.875	0.700	2.421	0.992	HVA22-like protein	GM-M
MELO3C009344.2	14.562	16.722	15.269	39.489	28.344	11.276	23.533	Adenosine kinase, putative	GM-M
MELO3C009182.2	16.899	18.193	6.573	91.768	15.069	43.743	10.322	Glycerol-3-phosphate acyltransferase	GM-M
MELO3C014448.2	13.853	17.935	4.756	56.668	13.505	24.272	11.842	Peroxisomal membrane 22 kDa (Mpv17/PMP22) family protein	GM-M
MELO3C026765.2	3.090	4.391	14.232	23.960	5.520	9.861	6.047	Protein SIEVE ELEMENT OCCLUSION B	GM-M
MELO3C026766.2	0.887	1.709	6.052	3.541	0.649	0.291	0.815	Protein SIEVE ELEMENT OCCLUSION B	GM-M
MELO3C022998.2	433.280	312.151	141.783	942.938	391.441	428.713	401.759	Malate dehydrogenase	GM-M
MELO3C023001.2	13.168	13.595	21.526	28.363	20.751	9.740	21.144	S-adenosyl-L-methionine-dependent methyltransferases superfamily protein	GM-M
MELO3C023043.2	1.307	1.304	1.150	4.906	1.916	1.398	1.709	Cysteine-rich repeat secretory protein	GM-M
MELO3C020429.2	140.487	110.228	35.513	381.392	137.424	116.845	122.129	Serine hydroxymethyltransferase	GM-M
MELO3C008768.2	4.104	3.877	3.182	9.358	5.185	1.722	4.534	Beta-hexosaminidase	GM-M
MELO3C003916.2	1.822	1.436	1.589	20.906	4.049	8.889	4.193	Cellulose synthase	GM-M
MELO3C004352.2	11.492	15.790	8.869	32.154	9.441	8.807	8.648	acyltransferase-like protein At1g54570, chloroplastic	GM-M
MELO3C004424.2	6.391	4.313	4.955	16.155	6.885	7.029	9.614	Biotin carboxyl carrier protein of acetyl-CoA carboxylase	GM-M
MELO3C004519.2	1.751	2.443	10.083	6.277	2.438	1.653	2.681	NADH-cytochrome b5 reductase-like protein	GM-M
MELO3C004576.2	0.632	1.198	0.084	3.445	0.259	1.687	0.188	sugar transport protein 14-like	GM-M
MELO3C006165.2	0.830	1.245	0.308	3.301	0.370	1.245	0.877	glyoxylate/hydroxypyruvate reductase HPR3-like	GM-M
MELO3C006195.2	10.379	10.948	10.951	21.915	17.470	5.462	24.315	Receptor-like kinase	GM-M
MELO3C006198.2	17.269	13.111	8.710	39.684	18.690	13.821	19.189	UDP-glucuronate 4-epimerase 4	GM-M
MELO3C006266.2	0.381	1.078	0.757	25.852	1.127	4.059	0.738	Pectinesterase inhibitor	GM-M
MELO3C006559.2	2.920	6.209	0.797	21.011	1.268	1.997	0.703	Aquaporin NIP1.1, putative	GM-M
MELO3C006576.2	2.593	2.096	2.344	8.551	1.035	3.833	1.493	ARM repeat superfamily protein	GM-M
MELO3C006590.2	0.764	0.697	0.626	28.543	NA	4.155	NA	DUF679 domain membrane protein 2	GM-M
MELO3C006759.2	0.497	0.651	1.087	1.373	0.399	0.408	0.649	Ca2+-activated RelA/SpoT-like protein	GM-M
MELO3C006964.2	8.476	8.442	12.883	30.938	12.369	7.491	13.517	Pyruvate kinase	GM-M
MELO3C006971.2	1.904	1.663	2.379	6.959	2.330	2.817	3.097	Receptor-like kinase	GM-M
MELO3C016421.2	46.256	36.827	12.346	101.570	54.561	49.660	50.426	Endoglucanase	GM-M
MELO3C032238.2	7.808	8.808	0.562	41.428	7.053	6.903	6.134	2S sulfur-rich seed storage protein 2-like	GM-M
MELO3C013751.2	6.252	5.679	15.536	12.918	9.038	1.744	10.493	Protein MID1-COMPLEMENTING ACTIVITY 1	GM-M

Gene ID	FPKM							Gene Description	Specific in episode
	FS	GI-M	GM-M	AN-M	GI-H	GM-H	AN-H		
MELO3C013762.2	3.375	6.534	0.859	29.817	2.038	10.723	3.583	Zeamatin	GM-M
MELO3C031937.2	2.494	3.685	62.976	14.396	6.799	5.419	6.372	acyl-CoA-binding protein	GM-M
MELO3C013908.2	0.355	0.694	0.878	1.489	0.596	0.740	0.497	Transmembrane protein	GM-M
MELO3C013954.2	11.101	12.084	1.715	25.363	4.829	5.956	14.618	14 kDa proline-rich protein dc2.15	GM-M
MELO3C031950.2	1.472	2.583	14.165	5.192	4.207	1.163	6.998	Unknown protein	GM-M
MELO3C014017.2	23.893	23.979	17.174	61.267	27.166	20.959	24.076	hydroxymethylglutaryl-CoA synthase	GM-M
MELO3C014109.2	2.142	3.331	2.183	9.076	5.430	1.808	5.272	Nucleotide/sugar transporter family protein	GM-M
MELO3C017005.2	12.883	8.718	6.756	30.797	16.498	5.881	20.908	L-ascorbate oxidase homolog	GM-M
MELO3C016981.2	2.633	1.459	35.565	10.389	7.619	1.844	10.361	60S ribosomal protein L39	GM-M
MELO3C016944.2	0.438	0.592	1.537	1.538	0.745	0.692	0.768	protein TIC 55, chloroplastic	GM-M
MELO3C025633.2	1.477	1.641	4.305	4.308	2.565	1.398	4.901	Hexosyltransferase	GM-M
MELO3C010479.2	20.723	20.457	10.676	49.633	21.988	16.781	29.987	Long-Chain Acyl-CoA Synthetase	GM-M
MELO3C024857.2	1.242	0.753	0.280	3.487	0.888	0.628	0.627	1-aminocyclopropane-1-carboxylate oxidase	GM-M
MELO3C016185.2	16.696	15.831	4.631	54.588	9.958	12.839	13.397	Phytoene synthase	GM-M
MELO3C016287.2	61.707	45.433	18.250	128.155	41.126	13.980	51.077	Endoglucanase	GM-M
MELO3C032603.2	38.958	26.964	10.372	100.736	35.946	17.734	23.537	GDP-mannose 4,6-dehydratase	GM-M
MELO3C017823.2	3.649	3.547	3.532	8.172	2.175	1.288	3.370	protein NRT1/ PTR FAMILY 6.1	GM-M
MELO3C018056.2	1.297	1.285	7.779	2.936	1.855	0.190	1.480	Carbonic anhydrase	GM-M
MELO3C007022.2	21.288	17.977	5.168	43.433	13.611	21.586	11.807	UDP-N-acetylglucosamine diphosphorylase 2-like	GM-M
MELO3C007257.2	0.145	0.165	0.051	1.104	0.156	0.286	0.154	receptor-like protein kinase HSL1	GM-M
MELO3C007421.2	17.819	17.029	41.459	37.367	24.090	10.926	24.306	pyrophosphate--fructose 6-phosphate 1-phosphotransferase subunit beta	GM-M
MELO3C007494.2	4.034	2.853	1.374	9.138	2.733	2.470	1.730	Hexosyltransferase	GM-M
MELO3C007815.2	0.235	0.566	0.347	1.452	0.362	0.722	0.398	Sugar transporter, putative	GM-M
MELO3C007828.2	1.147	1.543	1.951	3.366	2.139	1.202	1.578	Ribosomal protein L6	GM-M
MELO3C007872.2	35.362	30.397	35.003	99.049	61.795	14.858	66.172	Beta-galactosidase	GM-M
MELO3C024531.2	2.037	3.633	6.575	58.230	4.333	3.944	1.358	GDSL esterase/lipase At5g03610-like	GM-M
MELO3C024519.2	344.946	276.291	85.359	821.485	336.048	373.956	284.234	Fructose-bisphosphate aldolase	GM-M
MELO3C024495.2	3.322	3.753	2.911	41.202	2.874	8.616	4.074	Cytochrome P450	GM-M
MELO3C008796.2	16.628	12.634	3.211	34.470	9.403	7.545	9.765	Short-chain dehydrogenase/reductase family protein	GM-M
MELO3C003228.2	1.071	0.989	0.493	5.692	2.114	1.916	1.605	Laccase	GM-M

Gene ID	FPKM							Gene Description	Specific in episode
	FS	GI-M	GM-M	AN-M	GI-H	GM-H	AN-H		
MELO3C021985.2	6.781	11.495	4.410	32.346	8.837	3.047	9.685	protease Do-like 8, chloroplastic	GM-M
MELO3C025505.2	3.013	2.920	4.711	6.287	2.745	2.520	3.369	IQ-domain 1	GM-M
MELO3C005291.2	15.273	12.314	25.634	31.262	29.159	10.106	30.446	21 kDa protein	GM-M
MELO3C005748.2	5.622	7.426	1.788	53.214	4.328	13.585	4.001	Endoglucanase	GM-M
MELO3C012477.2	15.198	14.615	14.795	38.959	20.340	11.148	19.607	Aspartic proteinase nepenthesin-1	GM-M
MELO3C012454.2	3.146	4.831	11.838	15.917	7.120	2.619	10.611	Annexin	GM-M
MELO3C012440.2	5.680	4.949	3.776	11.816	7.118	2.139	6.880	Carboxypeptidase	GM-M
MELO3C012240.2	0.210	0.978	0.835	2.336	NA	0.640	0.292	protein NIM1-INTERACTING 2	GM-M
MELO3C012108.2	90.911	76.771	22.227	257.377	79.613	113.292	57.091	Expansin	GM-M
MELO3C012100.2	0.350	0.883	6.214	9.784	0.520	2.355	0.323	ribonuclease 3-like	GM-M
MELO3C025183.2	1.274	1.319	0.372	3.473	0.672	1.475	0.864	Cytochrome P450 protein	GM-M
MELO3C018368.2	4.667	3.809	1.203	10.120	3.861	2.768	3.605	omega-hydroxypalmitate O-feruloyl transferase	GM-M
MELO3C020949.2	2.019	1.906	4.041	4.520	3.894	1.922	4.740	4-alpha-glucanotransferase	GM-M
MELO3C020802.2	4.395	5.186	2.104	12.078	4.529	5.657	3.520	Thaumatin-like protein 1	GM-M
MELO3C020780.2	20.205	24.300	11.206	56.946	24.408	26.365	22.830	Triosephosphate isomerase	GM-M
MELO3C019361.2	1.700	2.779	2.016	13.581	1.358	2.888	1.997	fasciclin-like arabinogalactan protein 7	GM-M
MELO3C025848.2	7.667	10.391	24.974	26.863	11.452	8.582	10.743	Peptidyl-prolyl cis-trans isomerase	GM-M
MELO3C026832.2	1.189	1.063	0.800	3.548	1.234	0.777	1.537	zinc transporter 6, chloroplastic	GM-M
MELO3C025685.2	1.647	1.811	1.139	4.173	1.268	1.687	1.128	F-box/LRR-repeat protein 15	GM-M
MELO3C021458.2	18.630	13.894	18.890	37.996	30.702	7.459	36.898	glucomannan 4-beta-mannosyltransferase 9-like	GM-M
MELO3C021447.2	8.321	6.262	6.247	18.244	13.557	9.078	11.310	Stress-response A/B barrel domain-containing protein At5g22580	GM-M
MELO3C021277.2	3.347	4.798	53.608	11.724	6.131	3.479	9.919	60S ribosomal protein L29	GM-M
MELO3C021245.2	41.355	59.767	20.398	146.337	39.750	11.604	34.248	polygalacturonase At1g48100	GM-M
MELO3C022441.2	2.133	1.441	15.214	4.918	2.559	0.594	3.480	60S ribosomal protein L38	GM-M
MELO3C020670.2	0.900	0.949	3.150	2.462	0.514	0.836	0.405	3-ketoacyl-CoA synthase	GM-M
MELO3C026068.2	0.501	0.537	5.504	1.309	0.817	0.409	1.036	Transcriptional adapter ADA2b-like protein	GM-M
MELO3C021682.2	6.633	6.367	2.816	67.538	13.634	6.181	9.699	TNFR/CD27/30/40/95 cysteine-rich region	GM-M
MELO3C002672.2	1.962	1.553	1.533	9.750	2.940	3.586	2.002	Cellulose synthase	GM-M
MELO3C002665.2	0.286	0.378	0.787	1.212	0.190	0.581	0.341	O-fucosyltransferase family protein	GM-M
MELO3C002131.2	11.520	9.683	13.401	35.302	12.304	4.946	6.992	Phosphoethanolamine n-methyltransferase, putative	GM-M

Gene ID	FPKM							Gene Description	Specific in episode
	FS	GI-M	GM-M	AN-M	GI-H	GM-H	AN-H		
MELO3C002114.2	0.997	0.938	0.658	2.987	1.386	0.897	0.402	Transmembrane protein, putative	GM-M
MELO3C002016.2	1.555	1.445	1.580	3.717	1.156	1.184	1.871	zinc finger protein ZAT4-like	GM-M
MELO3C035414.2	0.760	0.434	4.316	1.523	1.136	0.171	0.904	RING/FYVE/PHD zinc finger superfamily protein	GM-M
MELO3C028945.2	3.998	2.315	2.959	8.675	5.194	3.649	4.918	Unknown protein	GM-M
MELO3C015395.2	0.961	0.851	0.732	2.799	0.980	1.165	0.638	late embryogenesis abundant protein D-34-like	GM-M
MELO3C006400.2	3.040	1.103	4.857	7.109	1.448	1.234	2.611	50S ribosomal protein L28, chloroplastic	GM-M
MELO3C023318.2	3.771	3.156	5.834	9.543	4.986	2.333	6.001	bZIP transcription factor 53-like	GM-M
MELO3C001082.2	11.502	10.957	107.650	23.151	9.564	7.722	11.716	Protein TIC 214	GM-M
MELO3C018491.2	0.584	0.635	3.638	1.490	0.588	0.302	1.133	RNA pseudouridine synthase 4, mitochondrial	GM-M
MELO3C026775.2	0.291	0.334	4.173	1.173	0.429	0.223	0.343	Protein SIEVE ELEMENT OCCLUSION B	GM-M
MELO3C020097.2	1.776	1.959	41.148	4.757	1.809	1.496	2.777	Ribosomal protein S7	GM-M
MELO3C020586.2	0.569	0.629	11.451	1.663	0.718	0.413	0.904	Mitochondrial import inner membrane translocase subunit Tim10	GM-M
MELO3C002736.2	1.236	1.198	7.218	2.498	1.819	0.569	2.042	Calcyclin-binding protein	GM-M
MELO3C002070.2	0.514	0.537	1.827	1.272	1.723	0.148	1.692	Peptidyl-prolyl cis-trans isomerase	GM-M
MELO3C027345.2	6.912	11.856	7.665	12.011	4.150	60.321	4.752	Kelch repeat-containing F-box family protein	AN-M
MELO3C000070.2	0.346	18.798	0.873	50.283	0.347	271.096	1.225	sugar transport protein 10-like	AN-M
MELO3C000776.2	4.534	2.076	1.957	5.066	2.478	50.624	3.622	Aquaporin	AN-M
MELO3C028533.2	0.264	1.116	0.517	1.713	NA	57.137	NA	Myb/SANT-like DNA-binding domain protein	AN-M
MELO3C018407.2	4.990	7.229	17.108	9.330	4.569	22.592	3.786	4-hydroxy-3-methylbut-2-enyl diphosphate reductase	AN-M
MELO3C018473.2	30.950	536.250	14.774	1851.161	141.123	3959.458	84.062	Cysteine proteinase	AN-M
MELO3C018801.2	11.553	26.893	4.219	16.536	7.661	62.658	8.182	B-box zinc finger protein 21	AN-M
MELO3C018862.2	2.903	0.789	1.321	4.298	2.762	14.845	2.000	Fasciclin-like arabinogalactan protein	AN-M
MELO3C024162.2	3.736	12.685	1.194	5.214	3.873	33.522	1.795	Oil body-associated protein 1A	AN-M
MELO3C024964.2	5.840	20.466	3.997	7.093	4.770	66.155	6.009	E3 ubiquitin-protein ligase	AN-M
MELO3C024949.2	1.782	5.643	8.501	3.887	4.134	24.631	3.517	RPM1-interacting protein 4	AN-M
MELO3C013228.2	7.980	15.259	6.297	31.587	5.656	177.379	5.874	chaperone protein ClpD, chloroplastic	AN-M
MELO3C013250.2	17.867	31.795	12.458	25.300	21.976	79.117	27.789	Amino acid transporter, putative	AN-M
MELO3C013255.2	2.570	1.220	3.743	2.297	1.903	6.132	1.831	Hexosyltransferase	AN-M
MELO3C013307.2	11.112	7.670	2.733	11.279	7.978	27.235	6.608	L-ascorbate oxidase homolog	AN-M
MELO3C013435.2	8.160	14.601	6.993	14.934	3.668	32.367	3.780	Prostatic spermine-binding-like protein	AN-M

Gene ID	FPKM							Gene Description	Specific in episode
	FS	GI-M	GM-M	AN-M	GI-H	GM-H	AN-H		
MELO3C012526.2	4.618	8.459	6.441	11.163	7.296	46.813	9.117	Adenylyl cyclase-associated protein	AN-M
MELO3C012601.2	0.651	1.479	1.237	3.775	1.116	20.927	1.854	Transglycosylase SLT domain containing protein expressed	AN-M
MELO3C021073.2	11.314	21.322	5.250	18.902	9.476	117.027	11.718	Quinone oxidoreductase	AN-M
MELO3C015691.2	5.200	9.866	1.976	16.085	4.398	147.278	3.361	3-hydroxy-3-methylglutaryl coenzyme A reductase	AN-M
MELO3C015766.2	2.070	8.315	0.980	1.147	1.333	31.298	0.255	phospholipase A1-Igamma2, chloroplastic	AN-M
MELO3C015860.2	0.222	0.639	0.222	0.589	0.376	1.489	0.249	Calcium permeable stress-gated cation channel 1	AN-M
MELO3C015979.2	1.299	20.563	0.187	57.273	NA	171.916	1.129	Haloacid dehalogenase-like hydrolase (HAD) superfamily protein	AN-M
MELO3C015980.2	1.095	11.774	0.135	27.433	0.625	107.634	0.671	Haloacid dehalogenase-like hydrolase (HAD) superfamily protein	AN-M
MELO3C015995.2	194.494	605.835	206.770	370.015	100.846	3259.988	124.270	Late embryogenesis abundant 3 family protein	AN-M
MELO3C016007.2	5.332	19.612	4.244	29.014	3.804	698.352	5.637	universal stress protein A-like protein	AN-M
MELO3C016050.2	20.287	13.058	13.074	36.343	17.923	84.741	19.051	Malate dehydrogenase	AN-M
MELO3C016052.2	0.504	1.909	3.151	0.789	0.987	4.256	0.419	Zinc metalloproteinase aureolysin	AN-M
MELO3C023607.2	5.293	15.289	14.252	7.319	8.833	40.712	7.733	Autophagy-related protein	AN-M
MELO3C023590.2	6.430	18.309	11.941	16.283	11.819	61.831	13.713	Pheophytinase, chloroplastic	AN-M
MELO3C023586.2	0.769	1.975	1.060	2.130	0.830	29.636	1.258	Carboxypeptidase	AN-M
MELO3C023551.2	24.177	10.266	20.339	13.231	28.379	55.341	39.662	Cellulose synthase	AN-M
MELO3C023539.2	2.739	37.783	1.332	33.369	3.575	110.744	1.934	Unknown protein	AN-M
MELO3C023536.2	31.748	61.532	17.294	32.172	19.357	167.223	17.026	zinc finger A20 and AN1 domain-containing stress-associated protein 5	AN-M
MELO3C023484.2	0.802	2.632	0.935	3.125	0.947	7.346	0.188	AP2-like ethylene-responsive transcription factor At1g16060	AN-M
MELO3C023465.2	2.904	5.596	1.056	26.798	2.697	207.187	2.876	Cinnamoyl-CoA reductase	AN-M
MELO3C023452.2	1.735	3.938	1.723	3.330	1.155	22.062	2.649	F-box/LRR-repeat protein 14	AN-M
MELO3C023426.2	2.944	6.725	2.121	8.802	3.525	21.165	2.979	Post-illumination chlorophyll fluorescence increase	AN-M
MELO3C023394.2	4.809	1.304	1.355	2.374	1.393	9.863	1.580	(6-4)DNA photolyase isoform X1	AN-M
MELO3C024226.2	6.293	10.839	3.348	23.195	5.187	92.768	4.505	Sulfate transporter 3.1	AN-M
MELO3C024297.2	1.522	3.121	12.698	4.084	3.445	16.112	2.400	ATP synthase subunit epsilon, mitochondrial	AN-M
MELO3C015280.2	0.709	1.898	2.930	2.474	2.659	4.990	2.346	calmodulin-binding protein 60 C-like isoform X1	AN-M
MELO3C015332.2	0.607	0.977	1.215	1.496	0.912	4.425	0.769	nuclear transcription factor Y subunit C-9	AN-M
MELO3C015375.2	15.392	39.932	11.440	34.689	25.223	114.641	31.982	Sodium/hydrogen exchanger	AN-M
MELO3C015469.2	6.676	12.540	10.688	18.476	13.148	180.578	10.769	beta-galactosidase-like	AN-M

Gene ID	FPKM							Gene Description	Specific in episode
	FS	GI-M	GM-M	AN-M	GI-H	GM-H	AN-H		
MELO3C015470.2	6.888	29.274	37.826	25.703	12.744	166.235	11.403	beta-galactosidase	AN-M
MELO3C015564.2	6.839	10.764	4.335	9.352	7.860	21.827	7.359	Pyruvate dehydrogenase E1 component subunit beta	AN-M
MELO3C010174.2	7.495	15.194	19.367	15.035	10.124	54.324	8.270	protein ECERIFERUM 3	AN-M
MELO3C010188.2	12.665	80.509	28.651	23.134	22.936	174.666	14.745	Cinnamoyl-CoA reductase, putative	AN-M
MELO3C010273.2	5.584	10.090	1.935	12.190	4.544	31.937	3.508	Transmembrane protein	AN-M
MELO3C010308.2	0.392	0.084	0.578	1.087	0.544	4.416	0.832	vesicle-associated membrane protein 711-like	AN-M
MELO3C010349.2	0.180	1.873	0.428	2.391	0.301	5.536	0.184	Metal-nicotianamine transporter	AN-M
MELO3C024696.2	1.003	2.409	7.104	1.802	2.590	9.906	1.879	transcription factor bHLH68 isoform X1	AN-M
MELO3C017438.2	2.175	5.079	7.578	4.362	2.908	11.709	2.723	cadmium/zinc-transporting ATPase HMA3-like	AN-M
MELO3C017200.2	1.014	3.363	2.863	1.293	1.555	9.965	2.186	LRR receptor-like kinase family protein	AN-M
MELO3C017159.2	1.305	2.982	1.673	2.598	1.589	6.640	1.476	ABC transporter A family member 2	AN-M
MELO3C017104.2	9.729	18.202	9.932	9.528	5.963	39.467	7.193	Trehalose-6-phosphate synthase, putative	AN-M
MELO3C026184.2	0.529	5.658	1.822	2.600	2.364	106.603	1.206	Bidirectional sugar transporter SWEET	AN-M
MELO3C008099.2	8.719	6.260	4.679	8.184	4.522	17.883	6.109	eukaryotic translation initiation factor 2D	AN-M
MELO3C008218.2	2.077	4.459	9.552	4.691	3.371	25.287	2.005	Plant UBX domain-containing protein 1	AN-M
MELO3C008250.2	0.595	3.117	0.193	2.162	0.608	7.015	0.296	ras-related protein Rab2BV	AN-M
MELO3C008331.2	0.328	0.913	1.885	5.029	0.268	83.500	0.513	ethylene-responsive transcription factor ERF113-like	AN-M
MELO3C008417.2	5.952	12.765	1.823	11.189	2.954	49.308	2.451	Bidirectional sugar transporter SWEET	AN-M
MELO3C008481.2	30.891	54.227	13.860	55.447	22.778	146.271	23.137	Glutamate synthase 1 [nadh], chloroplastic	AN-M
MELO3C010648.2	6.330	16.380	4.375	13.354	4.399	47.086	4.908	Amino acid permease	AN-M
MELO3C011635.2	5.848	12.078	11.064	6.298	5.710	36.015	5.104	BnaC04g02880D protein	AN-M
MELO3C011629.2	11.552	17.082	2.445	21.988	8.763	45.711	9.781	Protein phosphatase-2c, putative	AN-M
MELO3C011580.2	14.863	24.387	9.603	22.268	14.188	108.567	12.673	Selenoprotein K	AN-M
MELO3C026153.2	1.199	1.905	2.609	2.057	1.577	6.747	1.170	Regulator of Vps4 activity in the MVB pathway protein	AN-M
MELO3C020000.2	1.523	4.033	1.252	12.789	0.757	60.307	0.768	adenine/guanine permease AZG1	AN-M
MELO3C019959.2	2.908	4.786	4.765	4.351	3.693	10.902	4.051	Eukaryotic translation initiation factor 3 subunit C	AN-M
MELO3C019884.2	19.839	8.080	11.233	20.970	4.691	47.102	8.261	cytochrome P450 77A3-like	AN-M
MELO3C019832.2	7.850	15.585	7.217	12.859	6.512	135.888	5.999	p-loop nucleoside triphosphate hydrolase superfamily protein	AN-M
MELO3C019815.2	1.159	3.429	3.398	2.050	2.261	14.673	1.020	Receptor-like protein kinase	AN-M
MELO3C019794.2	3.214	1.530	1.237	3.827	2.376	17.104	2.268	Aquaporin PIP2	AN-M
MELO3C026498.2	1.987	4.646	7.187	5.115	2.145	21.173	1.187	Plant/protein	AN-M

Gene ID	FPKM							Gene Description	Specific in episode
	FS	GI-M	GM-M	AN-M	GI-H	GM-H	AN-H		
MELO3C026550.2	3.447	7.027	8.528	13.718	3.108	36.919	3.188	phosphoprotein ECPP44-like	AN-M
MELO3C011389.2	9.862	27.413	1.479	112.754	6.196	2184.460	3.318	Alpha/beta-Hydrolases superfamily protein	AN-M
MELO3C011232.2	4.468	7.482	1.540	7.972	2.694	31.603	2.331	Boron transporter-like protein	AN-M
MELO3C011192.2	5.198	8.033	1.189	9.667	1.758	29.480	2.269	Plasma membrane ATPase	AN-M
MELO3C011158.2	0.247	4.217	0.361	1.138	0.232	24.872	0.331	Flavin-containing monooxygenase	AN-M
MELO3C011056.2	0.768	1.901	0.886	6.458	1.182	44.424	1.235	purine permease 3-like	AN-M
MELO3C010982.2	40.960	129.693	5.735	111.223	26.175	524.420	26.446	cysteine proteinase RD19a-like	AN-M
MELO3C010920.2	42.311	26.690	12.868	49.091	34.566	146.470	40.547	Leishmanolysin-like peptidase	AN-M
MELO3C010919.2	2.236	55.954	34.860	3.878	39.547	1222.700	7.716	Thaumatin	AN-M
MELO3C010781.2	56.757	35.159	18.658	60.200	33.221	290.357	57.754	squalene monooxygenase-like	AN-M
MELO3C010778.2	3.094	5.875	6.320	2.344	2.799	12.298	3.228	CSC1-like protein RXW8 isoform X1	AN-M
MELO3C010754.2	140.839	97.181	22.548	170.607	92.591	384.396	122.226	glycine dehydrogenase (Decarboxylating), mitochondrial	AN-M
MELO3C010750.2	4.691	1.797	1.464	3.950	1.128	13.954	1.101	ADP,ATP carrier protein, putative	AN-M
MELO3C010748.2	0.116	0.731	0.531	0.493	0.278	2.094	0.600	Heat shock transcription factor	AN-M
MELO3C010725.2	8.714	28.240	12.184	74.664	3.102	253.983	4.941	B12D protein	AN-M
MELO3C003345.2	2.511	5.416	5.144	4.436	3.520	11.232	3.355	Receptor-like protein kinase	AN-M
MELO3C003371.2	0.099	1.535	1.070	0.429	0.149	35.863	0.230	Expansin-like protein	AN-M
MELO3C003382.2	2.242	9.324	1.673	33.196	3.587	288.379	1.877	protein NRT1/ PTR FAMILY 7.3-like	AN-M
MELO3C003494.2	0.675	2.168	0.176	4.484	0.325	13.805	0.538	Phosphoglycerate mutase-like protein 1	AN-M
MELO3C003525.2	2.020	4.069	5.344	3.272	3.700	8.185	4.563	DNAJ heat shock N-terminal domain-containing family protein	AN-M
MELO3C003577.2	70.997	125.640	34.081	319.955	59.870	965.628	52.531	Aspartate aminotransferase	AN-M
MELO3C003662.2	0.066	0.359	0.359	0.312	0.226	1.799	0.269	Arf GTPase activating protein	AN-M
MELO3C003698.2	0.125	0.576	0.449	1.065	0.610	2.774	0.907	Serine/threonine-protein kinase	AN-M
MELO3C003731.2	0.530	1.227	1.443	0.892	0.317	2.693	0.502	Protein disulfide isomerase (PDI)-like protein	AN-M
MELO3C003783.2	10.061	4.930	4.652	10.211	7.049	38.607	10.785	WAT1-related protein	AN-M
MELO3C003791.2	3.534	11.432	3.195	13.734	6.697	47.038	3.848	Thioredoxin	AN-M
MELO3C003793.2	6.102	9.852	5.028	7.141	9.719	28.076	8.280	GTP-binding protein hflx, putative	AN-M
MELO3C003833.2	5.524	8.948	4.136	10.405	4.951	38.634	4.958	DUF21 domain-containing-like protein	AN-M
MELO3C003856.2	0.218	1.029	0.364	1.879	NA	8.970	NA	AT3g19540/T31J18_4	AN-M
MELO3C003898.2	1.086	2.188	2.551	2.616	1.993	7.264	3.854	inactive protein kinase SELMODRAFT_444075-like	AN-M
MELO3C018242.2	11.824	28.303	29.663	11.261	5.894	64.605	7.599	NAC domain-containing protein 72-like	AN-M

Gene ID	FPKM							Gene Description	Specific in episode
	FS	GI-M	GM-M	AN-M	GI-H	GM-H	AN-H		
MELO3C018184.2	1.235	5.455	0.545	14.058	1.641	48.304	1.197	Membrane lipoprotein lipid attachment site-like protein, putative (DUF1223)	AN-M
MELO3C018126.2	9.218	14.450	5.277	16.502	8.679	61.935	9.857	Autophagy-related protein 18f	AN-M
MELO3C018083.2	29.413	46.524	13.108	104.751	25.033	249.618	22.617	glucose-6-phosphate/phosphate translocator 2, chloroplastic-like	AN-M
MELO3C012866.2	2.314	3.796	5.179	3.742	2.103	9.765	3.577	Tetratricopeptide repeat protein 7A	AN-M
MELO3C012904.2	0.971	1.509	1.765	1.204	1.246	3.829	1.124	Autophagy-related protein 9	AN-M
MELO3C012939.2	1.817	4.840	1.272	10.106	1.693	20.916	3.859	Caffeoylshikimate esterase	AN-M
MELO3C013000.2	105.536	73.550	36.981	74.604	61.083	233.541	85.890	Malic enzyme	AN-M
MELO3C022699.2	17.929	25.519	7.678	53.210	20.522	136.189	23.878	Alanine aminotransferase 2	AN-M
MELO3C022734.2	0.957	2.120	1.093	6.569	0.266	65.016	0.585	Glycosyltransferase	AN-M
MELO3C022736.2	0.187	0.663	0.463	5.479	0.268	14.059	0.134	Glycosyltransferase	AN-M
MELO3C026666.2	0.993	2.806	2.033	5.194	1.732	10.733	2.234	Cytochrome b5	AN-M
MELO3C024027.2	3.736	2.049	1.005	2.298	1.443	14.186	1.354	Oligopeptide transporter, putative	AN-M
MELO3C026755.2	157.073	97.499	49.597	212.413	175.561	2306.840	213.099	Xyloglucan endotransglucosylase/hydrolase	AN-M
MELO3C026759.2	1.886	3.490	2.245	3.308	2.463	10.987	2.867	Eukaryotic initiation factor 4F subunit p150 isoform 1	AN-M
MELO3C026760.2	5.257	8.276	5.429	8.088	6.881	19.987	6.218	Arabinogalactan protein	AN-M
MELO3C009973.2	34.522	22.560	13.668	37.081	21.285	75.961	27.177	Dead box ATP-dependent RNA helicase, putative	AN-M
MELO3C009956.2	47.104	76.730	20.098	160.046	44.092	1730.985	50.353	formate dehydrogenase, mitochondrial	AN-M
MELO3C009948.2	0.949	2.087	1.631	1.747	1.358	7.620	2.914	homeobox-leucine zipper protein HAT5	AN-M
MELO3C009894.2	50.907	76.666	26.124	66.114	41.828	155.527	44.411	thioredoxin H2	AN-M
MELO3C009791.2	18.838	34.912	5.825	49.620	12.591	206.707	15.548	Cation calcium exchanger	AN-M
MELO3C009542.2	4.908	7.980	7.412	11.859	4.304	42.012	5.737	BEL1-like homeodomain protein 1	AN-M
MELO3C009540.2	12.488	24.632	7.476	17.211	13.306	84.966	15.000	NC domain-containing protein	AN-M
MELO3C009260.2	298.394	1194.672	175.089	807.225	285.057	3610.191	283.512	metallothionein-like protein type 2	AN-M
MELO3C009210.2	1.643	9.106	5.157	8.618	NA	28.062	2.335	Class I heat shock protein	AN-M
MELO3C014700.2	3.221	4.942	19.295	6.009	5.270	15.514	3.980	Non-specific serine/threonine protein kinase	AN-M
MELO3C014672.2	17.400	13.076	9.201	115.344	24.963	649.447	19.757	Lipid transfer protein	AN-M
MELO3C014658.2	0.651	10.228	5.384	3.166	2.745	52.297	9.195	Peroxidase	AN-M
MELO3C014595.2	5.145	9.039	5.716	17.277	6.246	138.632	5.234	Protein DETOXIFICATION	AN-M
MELO3C014547.2	5.103	8.347	5.359	9.127	5.335	65.836	6.321	hydroxymethylglutaryl-CoA lyase, mitochondrial-like	AN-M
MELO3C014417.2	9.463	13.446	3.238	85.243	8.683	386.613	9.092	sugar transporter ERD6-like 7	AN-M

Gene ID	FPKM							Gene Description	Specific in episode
	FS	GI-M	GM-M	AN-M	GI-H	GM-H	AN-H		
MELO3C014315.2	0.805	1.325	1.538	1.247	0.648	3.773	0.387	Bifunctional DNA-directed RNA polymerase subunit beta-beta	AN-M
MELO3C014294.2	1.174	2.459	0.678	2.996	0.797	52.177	1.183	Homocysteine S-methyltransferase	AN-M
MELO3C023067.2	0.754	2.712	1.188	10.165	0.463	351.756	0.283	Beta-amylase	AN-M
MELO3C020350.2	2.257	4.491	4.226	3.601	2.882	13.672	3.848	Emb|CAB89363.1	AN-M
MELO3C008709.2	21.130	31.605	7.476	43.105	21.124	242.721	23.566	Amino acid transporter, putative	AN-M
MELO3C008741.2	0.306	1.145	1.789	0.601	0.497	5.924	0.423	ABC transporter B family protein	AN-M
MELO3C008751.2	0.782	0.138	0.419	0.110	0.668	1.657	0.381	TPR repeat-containing protein ZIP4	AN-M
MELO3C004130.2	18.892	25.539	21.879	19.622	12.408	79.548	15.479	Signal recognition particle 54 kDa protein 1	AN-M
MELO3C004193.2	2.511	1.078	0.473	1.188	2.080	6.671	1.887	pyrophosphate-energized vacuolar membrane proton pump	AN-M
MELO3C004315.2	3.893	2.042	3.225	2.185	3.075	67.268	3.567	receptor-like cytosolic serine/threonine-protein kinase RBK2	AN-M
MELO3C004372.2	0.793	2.054	3.781	2.397	1.417	7.151	1.502	Peptide-N(4)-(N-acetyl-beta-glucosaminyl)asparagine amidase	AN-M
MELO3C004484.2	1.321	2.778	2.914	2.986	1.004	8.091	1.526	NEDD8 ultimate buster 1	AN-M
MELO3C004550.2	42.108	27.499	9.815	44.691	42.103	109.012	43.470	Leucine-rich repeat extensin-like protein 4	AN-M
MELO3C004596.2	2.289	8.170	1.072	10.004	0.854	24.088	1.099	Profilin	AN-M
MELO3C004638.2	1.978	5.545	3.643	6.488	3.080	30.618	2.455	60S ribosomal protein L18a-like protein	AN-M
MELO3C005947.2	7.232	1.233	1.214	3.656	1.528	20.184	0.103	basic blue protein	AN-M
MELO3C006007.2	3.743	5.876	3.312	7.416	3.328	15.579	2.528	ORMDL family protein	AN-M
MELO3C006019.2	0.407	1.227	2.650	1.143	0.498	4.260	0.265	lysine-specific demethylase JMJ25	AN-M
MELO3C006086.2	1.142	3.292	4.560	9.374	1.927	26.873	1.565	protein REVEILLE 6 isoform X2	AN-M
MELO3C006128.2	3.733	12.679	1.269	13.617	3.572	42.202	3.501	LIM domain-containing protein PLIM2b-like	AN-M
MELO3C006234.2	18.996	28.876	6.059	34.098	17.416	70.743	16.635	3-hydroxyisobutyryl-CoA hydrolase 1-like	AN-M
MELO3C006277.2	0.886	1.845	4.415	1.498	1.278	4.112	0.857	Gb|AAF01580.1	AN-M
MELO3C006316.2	27.758	51.323	28.270	43.179	34.616	139.323	30.606	cytochrome b5	AN-M
MELO3C006334.2	33.406	64.340	6.809	81.597	28.411	343.563	25.368	Peroxisomal 3-ketoacyl-CoA thiolase	AN-M
MELO3C006362.2	18.093	35.743	9.507	60.429	9.660	636.194	9.942	Beta-amylase	AN-M
MELO3C006429.2	1.318	2.756	2.178	2.780	1.842	5.835	2.121	Serine/threonine-protein phosphatase	AN-M
MELO3C006498.2	3.212	6.483	13.168	6.956	5.115	19.617	4.443	transmembrane emp24 domain-containing protein p24beta3	AN-M
MELO3C006502.2	23.264	49.085	29.563	37.638	30.830	131.856	25.053	1,2-dihydroxy-3-keto-5-methylthiopentene dioxygenase 2-like	AN-M
MELO3C006523.2	7.573	5.069	2.123	6.824	4.235	25.771	3.639	Folate-biopterin transporter 1, chloroplastic	AN-M
MELO3C006527.2	2.302	4.732	2.351	3.618	4.299	16.675	3.518	Heat shock protein	AN-M
MELO3C006545.2	3.782	8.411	6.539	7.045	4.933	17.130	5.829	Long-chain acyl-CoA synthetase	AN-M

Gene ID	FPKM							Gene Description	Specific in episode
	FS	GI-M	GM-M	AN-M	GI-H	GM-H	AN-H		
MELO3C006585.2	11.248	19.132	3.735	10.418	8.167	84.609	9.580	Pyruvate, phosphate dikinase	AN-M
MELO3C006684.2	6.376	9.137	6.108	12.086	5.641	25.938	6.309	Acyl-CoA dehydrogenase	AN-M
MELO3C006711.2	10.472	19.605	21.730	17.103	14.752	64.885	11.403	Universal stress protein A	AN-M
MELO3C006721.2	6.138	11.187	3.154	15.475	17.964	70.112	11.452	calmodulin	AN-M
MELO3C006917.2	6.727	3.777	2.676	3.886	3.614	22.438	3.396	Pectin acetylesterase	AN-M
MELO3C026931.2	0.342	1.238	9.335	0.728	1.261	6.599	0.479	RNA polymerase sigma factor	AN-M
MELO3C019497.2	0.154	1.085	0.998	1.326	0.096	3.301	0.186	Tubby-like F-box protein	AN-M
MELO3C019528.2	9.085	16.205	3.261	21.067	7.795	64.213	7.937	Glycosyltransferase	AN-M
MELO3C014957.2	2.693	1.746	1.790	2.430	2.711	7.860	2.541	Rhamnogalacturonate lyase family protein	AN-M
MELO3C014870.2	1.847	4.861	1.285	5.794	1.565	75.360	1.840	DSR6	AN-M
MELO3C014825.2	23.402	15.907	6.137	22.545	16.292	68.151	17.036	Steroid nuclear receptor, ligand-binding	AN-M
MELO3C016552.2	12.877	6.986	9.993	5.895	14.784	52.939	11.622	Fiber expressed protein	AN-M
MELO3C025375.2	3.725	8.950	8.065	5.977	3.327	22.821	2.692	Protein translation factor sui1-like protein	AN-M
MELO3C025405.2	0.211	1.993	2.127	2.465	0.642	5.936	0.595	early nodulin-like protein 3	AN-M
MELO3C025417.2	1.581	5.017	6.755	10.648	2.091	41.402	1.697	Polyubiquitin	AN-M
MELO3C013744.2	11.686	15.724	4.125	41.881	11.365	332.693	12.138	RING-H2 finger protein ATL4M, putative	AN-M
MELO3C013881.2	9.093	12.310	5.723	10.575	6.745	34.285	8.469	Lon protease homolog 2, peroxisomal	AN-M
MELO3C013918.2	3.015	8.710	9.036	2.428	9.149	20.516	6.115	Nudix hydrolase	AN-M
MELO3C014161.2	52.248	95.499	35.347	83.526	51.083	676.132	59.813	aspartic proteinase A1-like	AN-M
MELO3C017060.2	5.549	10.585	8.794	12.979	7.868	27.749	9.533	Auxin efflux carrier family protein, putative	AN-M
MELO3C017024.2	0.543	13.963	0.082	41.701	0.666	92.082	1.207	Catalase	AN-M
MELO3C017021.2	2.520	4.597	6.620	2.036	3.394	11.869	3.783	BTB/POZ domain-containing family protein	AN-M
MELO3C016991.2	0.520	1.257	1.745	0.698	0.475	3.163	0.491	Octicosapeptide/Phox/Bem1p (PB1) domain-containing protein / tetratricopeptide repeat (TPR)-containing protein	AN-M
MELO3C016776.2	1.499	3.213	2.501	1.860	2.019	11.127	1.487	50S ribosomal protein L4	AN-M
MELO3C025595.2	2.958	5.709	3.897	4.569	8.778	15.064	7.983	Hydroxyacylglutathione hydrolase 3, mitochondrial-like protein	AN-M
MELO3C026357.2	0.267	0.644	1.570	0.836	0.290	1.967	0.327	Callose synthase-like protein	AN-M
MELO3C022964.2	0.963	1.805	1.037	1.655	1.062	9.141	0.869	transmembrane protein 56-like	AN-M
MELO3C010560.2	0.311	5.065	3.807	10.802	0.510	30.960	3.330	Pentatricopeptide repeat-containing family protein	AN-M
MELO3C010500.2	11.468	36.284	10.763	47.789	3.864	304.976	6.584	NAC domain-containing protein, putative	AN-M
MELO3C010493.2	3.488	5.164	3.563	5.740	3.825	29.519	5.815	Aldehyde dehydrogenase	AN-M

Gene ID	FPKM							Gene Description	Specific in episode
	FS	GI-M	GM-M	AN-M	GI-H	GM-H	AN-H		
MELO3C032663.2	1.468	3.515	2.884	6.122	1.945	39.743	1.766	Kinesin-like protein	AN-M
MELO3C026298.2	2.843	6.833	5.194	6.195	4.819	23.571	2.990	DSBA oxidoreductase family protein	AN-M
MELO3C018967.2	0.428	2.151	0.426	2.458	0.228	66.182	0.309	Protein DETOXIFICATION	AN-M
MELO3C018990.2	1.502	2.436	3.889	2.847	1.796	9.924	2.380	ABC transporter D family member 1	AN-M
MELO3C024822.2	49.156	34.270	20.981	43.714	39.585	106.673	49.572	Splicing factor 3B subunit 1	AN-M
MELO3C016085.2	1.969	3.015	4.818	2.202	2.062	8.284	2.381	E3 ubiquitin-protein ligase Arkadia	AN-M
MELO3C016089.2	11.029	18.109	4.169	13.668	10.231	42.877	7.854	homogentisate 1,2-dioxygenase	AN-M
MELO3C016174.2	16.071	10.225	5.421	24.300	17.118	67.855	17.243	CASP-like protein	AN-M
MELO3C016203.2	0.456	1.043	17.783	2.092	1.485	7.143	1.376	Myosin-related family protein	AN-M
MELO3C032583.2	10.600	24.298	7.045	29.203	13.865	63.388	14.507	Mitochondrial gamma aminobutyrate transaminase 1	AN-M
MELO3C016259.2	0.283	6.992	0.191	12.936	1.282	36.989	1.594	Bidirectional sugar transporter SWEET	AN-M
MELO3C016271.2	0.731	2.311	0.637	1.101	0.416	5.080	0.596	cellulose synthase-like protein G3	AN-M
MELO3C016284.2	4.948	9.579	4.152	15.517	8.845	56.405	7.191	BnaC06g13730D protein	AN-M
MELO3C016290.2	21.679	38.675	25.946	22.044	27.677	99.893	24.195	Long-Chain Acyl-CoA Synthetase	AN-M
MELO3C016300.2	8.659	16.217	6.808	29.213	8.551	71.207	9.311	UDP-glucose 4-epimerase family protein	AN-M
MELO3C017603.2	2.880	4.571	1.576	4.482	2.503	58.065	2.330	Peroxidase	AN-M
MELO3C017673.2	1.701	10.921	1.145	36.390	3.933	97.087	2.707	Glycosyltransferase	AN-M
MELO3C017756.2	0.617	1.342	0.554	1.369	0.736	3.288	0.512	calcium-dependent protein kinase 24	AN-M
MELO3C017757.2	5.394	7.849	2.382	14.651	4.572	30.306	5.291	Inositol transporter 1	AN-M
MELO3C017833.2	10.671	19.913	6.760	19.561	12.917	46.295	12.835	Golgi SNAP receptor complex member 1-1	AN-M
MELO3C017954.2	0.305	0.949	1.130	1.595	0.625	17.100	0.548	Caffeoylshikimate esterase	AN-M
MELO3C017989.2	52.022	30.646	16.437	60.615	49.107	127.888	31.218	PXMP2/4 family protein 4	AN-M
MELO3C007018.2	3.846	2.729	6.648	2.187	3.158	12.573	3.108	Chromatin remodeling complex subunit	AN-M
MELO3C007151.2	6.561	14.682	25.487	10.176	10.398	34.413	7.363	transcription factor bHLH144	AN-M
MELO3C007179.2	0.973	0.284	0.382	0.351	0.665	5.085	0.668	Subtilisin-like protease	AN-M
MELO3C007213.2	0.206	10.850	0.024	20.894	NA	95.150	0.839	olee1-like protein isoform X1	AN-M
MELO3C007311.2	2.242	3.739	1.782	6.127	1.942	32.068	2.455	sphingosine kinase 1	AN-M
MELO3C007405.2	5.126	14.804	5.440	7.459	9.346	31.903	6.948	peroxisomal membrane protein PMP22	AN-M
MELO3C007456.2	3.589	5.501	2.137	7.113	2.561	15.090	2.426	UDP-galactose transporter 2-like	AN-M
MELO3C007469.2	0.528	1.321	3.025	1.422	1.282	3.004	0.471	iron-sulfur assembly protein IscA, chloroplastic	AN-M
MELO3C007485.2	5.399	2.727	1.516	10.760	2.662	66.702	2.272	shikimate kinase 1, chloroplastic	AN-M

Gene ID	FPKM							Gene Description	Specific in episode
	FS	GI-M	GM-M	AN-M	GI-H	GM-H	AN-H		
MELO3C007568.2	2.489	5.444	2.084	8.220	4.043	18.406	3.745	PI-PLC X domain-containing protein At5g67130	AN-M
MELO3C007597.2	19.538	8.384	5.436	4.745	3.608	103.023	2.201	Indole-3-acetic acid-amido synthetase GH3.3	AN-M
MELO3C007636.2	8.442	11.851	2.249	9.326	3.674	49.438	4.474	Calcium-transporting ATPase	AN-M
MELO3C007739.2	5.923	15.675	7.580	15.911	3.420	64.785	4.281	serine/threonine-protein kinase SAPK2	AN-M
MELO3C007917.2	1.395	4.014	2.313	1.469	3.159	11.969	3.256	thiosulfate sulfurtransferase 18 isoform X1	AN-M
MELO3C008024.2	6.097	10.337	18.419	10.322	8.971	30.471	9.060	Transcription elongation factor (TFIIS) family protein	AN-M
MELO3C008025.2	0.196	0.971	7.797	0.533	0.660	1.948	1.008	WAT1-related protein	AN-M
MELO3C008048.2	6.274	3.954	5.991	3.503	11.344	16.610	9.020	Chromatin assembly factor 1 subunit FAS2	AN-M
MELO3C019109.2	5.166	8.715	3.841	44.247	7.385	184.416	6.048	3-oxoacyl-[acyl-carrier-protein] reductase FabG-like	AN-M
MELO3C019125.2	1.776	4.567	3.015	4.354	2.173	38.924	3.440	WAT1-related protein At5g07050-like	AN-M
MELO3C033295.2	0.464	3.960	2.240	10.853	NA	32.000	3.600	Unknown protein	AN-M
MELO3C023110.2	285.366	456.615	66.395	530.695	333.363	1229.341	379.048	Alkaline alpha galactosidase	AN-M
MELO3C008846.2	0.991	1.713	1.118	1.138	0.837	4.601	1.006	Copper-transporting atpase p-type, putative	AN-M
MELO3C008836.2	3.016	1.570	1.027	2.571	1.652	16.236	1.518	plant cysteine oxidase 2-like	AN-M
MELO3C025205.2	0.497	1.310	0.484	1.810	0.674	11.264	0.698	RING-type E3 ubiquitin transferase	AN-M
MELO3C026054.2	12.988	21.534	14.890	23.624	14.339	77.329	15.211	Saposin B domain protein	AN-M
MELO3C014227.2	2.178	4.692	0.703	36.356	3.562	90.315	3.392	phenylalanine ammonia-lyase	AN-M
MELO3C003219.2	4.792	6.853	3.336	7.044	7.408	22.532	6.872	Chaperone protein dnaJ-like protein	AN-M
MELO3C022176.2	2.871	5.342	1.424	9.987	1.467	42.477	1.747	Non-lysosomal glucosylceramidase	AN-M
MELO3C022138.2	15.328	40.651	11.082	69.286	29.287	164.657	30.836	Protein disulfide isomerase (PDI)-like protein 2	AN-M
MELO3C022102.2	9.488	14.446	4.906	22.008	8.263	54.782	9.131	quinone oxidoreductase-like protein 2 homolog	AN-M
MELO3C022099.2	1.148	2.905	2.353	3.171	2.755	8.346	2.303	Alpha/beta-Hydrolases superfamily protein	AN-M
MELO3C022086.2	5.580	14.030	23.266	11.928	11.684	29.639	11.818	cyclin-B1-2	AN-M
MELO3C022050.2	1.878	3.099	3.167	2.501	2.434	6.711	2.625	E3 ubiquitin-protein ligase	AN-M
MELO3C021992.2	6.627	11.269	3.103	16.520	6.709	33.234	5.299	Ribosomal protein L34Ae	AN-M
MELO3C021510.2	4.938	12.939	14.971	10.260	7.652	93.932	7.140	Ferredoxin-thioredoxin reductase catalytic chain	AN-M
MELO3C021562.2	24.610	62.523	23.299	60.265	25.966	144.540	24.410	Glutaredoxin	AN-M
MELO3C021633.2	1.014	13.213	2.106	29.035	0.257	88.444	0.920	Actin family protein	AN-M
MELO3C021658.2	177.732	106.122	49.292	183.874	111.238	398.040	131.168	Heat shock 70 kDa protein	AN-M
MELO3C025448.2	1.838	6.840	2.729	10.143	2.559	33.258	1.980	enoyl-CoA delta isomerase 1, peroxisomal	AN-M
MELO3C003035.2	12.269	6.790	4.810	13.074	11.790	77.862	12.854	Auxin influx transporter	AN-M

Gene ID	FPKM							Gene Description	Specific in episode
	FS	GI-M	GM-M	AN-M	GI-H	GM-H	AN-H		
MELO3C022802.2	5.249	8.590	1.340	8.711	4.612	23.896	5.172	Sulfate transporter, putative	AN-M
MELO3C025076.2	13.816	21.350	24.302	75.249	17.252	225.891	17.380	Isocitrate dehydrogenase [NADP]	AN-M
MELO3C005084.2	1.118	2.471	1.265	3.370	0.974	11.651	1.329	E3 ubiquitin-protein ligase	AN-M
MELO3C005136.2	1.454	2.955	6.015	2.710	2.130	9.342	2.695	Protein EARLY RESPONSIVE TO DEHYDRATION 15	AN-M
MELO3C005170.2	1.227	3.344	6.035	2.401	3.865	6.747	4.062	Receptor-like kinase	AN-M
MELO3C005221.2	14.738	21.657	7.393	22.539	14.278	56.751	17.470	protein NRT1/ PTR FAMILY 8.3 isoform X1	AN-M
MELO3C005267.2	66.074	39.863	14.817	52.477	48.642	152.157	58.468	receptor protein kinase TMK1	AN-M
MELO3C005466.2	6.652	23.789	7.752	4.418	2.937	55.577	2.413	ethylene-responsive transcription factor ERF106-like	AN-M
MELO3C005473.2	2.528	4.397	2.453	4.186	1.691	17.305	2.018	Cytochrome P450	AN-M
MELO3C005538.2	6.529	13.618	6.853	12.060	9.200	48.397	11.769	Autophagy-related protein	AN-M
MELO3C005570.2	3.330	6.690	2.034	27.868	1.717	238.579	3.421	Cytochrome P450 family protein	AN-M
MELO3C005578.2	0.122	0.531	1.417	0.719	0.381	24.754	0.223	Amine oxidase	AN-M
MELO3C005597.2	4.237	9.326	4.124	12.871	3.985	105.672	4.439	1-aminocyclopropane-1-carboxylate synthase	AN-M
MELO3C005607.2	0.672	1.147	1.177	3.528	0.614	11.999	1.163	Cytochrome P450, putative	AN-M
MELO3C005616.2	4.847	18.698	9.084	15.140	4.050	44.216	4.717	Protein STAY-GREEN, chloroplastic	AN-M
MELO3C005666.2	28.244	50.396	33.993	68.948	39.207	632.846	41.212	Glutathione peroxidase	AN-M
MELO3C005798.2	5.838	14.979	5.652	18.569	10.006	65.636	9.147	StAR-related lipid transfer 7, mitochondrial	AN-M
MELO3C012457.2	41.011	53.280	6.995	76.515	27.427	593.084	30.323	Annexin	AN-M
MELO3C012286.2	0.997	1.738	1.091	1.862	1.115	4.490	1.260	CDP-diacylglycerol--serine O-phosphatidyltransferase 1	AN-M
MELO3C012200.2	0.536	2.726	0.608	2.736	0.473	8.189	0.775	Phosphate transporter PHO1-like protein	AN-M
MELO3C012159.2	19.617	35.975	7.283	43.935	15.800	165.876	14.424	6-phosphogluconolactonase, putative	AN-M
MELO3C012029.2	70.656	99.049	20.655	84.531	61.204	213.584	62.592	Inositol-1,4,5-trisphosphate 5-phosphatase 4 isoform 2	AN-M
MELO3C011974.2	0.771	10.894	0.274	14.508	1.105	40.072	0.654	pectinesterase inhibitor-like	AN-M
MELO3C011973.2	1.568	7.082	0.799	27.887	2.399	65.841	1.339	pectinesterase inhibitor-like	AN-M
MELO3C011955.2	0.989	2.184	1.923	2.642	2.557	7.113	1.701	Tetratricopeptide-like helical	AN-M
MELO3C011945.2	1.304	2.582	8.090	1.409	2.628	9.841	4.090	BTB/POZ domain-containing protein At1g67900	AN-M
MELO3C011809.2	2.583	5.264	2.627	9.301	3.613	20.222	3.507	protein ZINC INDUCED FACILITATOR-LIKE 1-like	AN-M
MELO3C011713.2	1.091	3.095	2.114	5.211	1.518	12.742	2.243	Phosphatase 2C family protein	AN-M
MELO3C020030.2	2.742	6.225	2.468	5.286	2.407	22.974	2.767	Protease	AN-M
MELO3C020131.2	0.735	1.988	1.399	1.444	1.298	31.248	2.476	Potassium transporter	AN-M
MELO3C034424.2	0.273	0.663	0.301	0.753	0.392	2.213	0.561	AP-3 complex subunit sigma-like	AN-M

Gene ID	FPKM							Gene Description	Specific in episode
	FS	GI-M	GM-M	AN-M	GI-H	GM-H	AN-H		
MELO3C026435.2	2.440	4.572	4.356	3.211	3.487	9.639	2.589	Ubiquitin-conjugating enzyme, E2	AN-M
MELO3C026436.2	2.284	11.775	4.219	22.983	1.783	87.895	2.068	1-aminocyclopropane-1-carboxylate oxidase-1-like protein	AN-M
MELO3C023241.2	0.164	0.650	0.407	3.552	0.644	11.431	0.382	callose synthase 5-like	AN-M
MELO3C023266.2	0.227	1.262	1.747	1.027	0.595	3.592	0.625	Trihelix transcription factor GT-2	AN-M
MELO3C023300.2	2.480	5.735	4.310	3.038	3.707	83.944	5.286	Universal stress protein A-like protein	AN-M
MELO3C023316.2	0.524	3.070	0.357	2.827	0.170	13.533	0.164	phosphatidylinositol/phosphatidylcholine transfer protein SFH3-like isoform X1	AN-M
MELO3C023338.2	140.501	242.004	37.014	207.716	100.810	552.407	73.840	cysteine proteinase RD19a-like	AN-M
MELO3C020941.2	12.294	37.634	5.656	43.052	8.706	329.784	7.995	Calcium uniporter, mitochondrial	AN-M
MELO3C020798.2	2.428	5.695	2.486	6.225	7.229	50.121	5.766	1-phosphatidylinositol phosphodiesterase	AN-M
MELO3C021940.2	23.788	38.166	10.446	21.669	15.033	115.073	15.220	Cold acclimation protein	AN-M
MELO3C021893.2	3.688	8.741	5.864	4.089	5.572	22.404	6.003	binding partner of ACD11 1-like isoform X1	AN-M
MELO3C021855.2	0.137	0.637	0.501	1.712	0.458	5.205	0.340	PLATZ transcription factor family protein	AN-M
MELO3C021853.2	17.895	28.012	6.519	58.393	23.441	145.921	20.104	UPF0131 protein At3g02910	AN-M
MELO3C024762.2	1.097	4.809	0.422	4.186	1.274	36.038	1.615	benzyl alcohol O-benzoyltransferase-like	AN-M
MELO3C019290.2	0.264	1.184	1.463	9.616	0.461	31.288	0.418	mitogen-activated protein kinase kinase kinase 2-like	AN-M
MELO3C019310.2	17.394	33.381	8.723	15.094	10.780	306.007	20.149	protein NRT1/ PTR FAMILY 6.4	AN-M
MELO3C025944.2	2.072	4.330	3.916	5.509	1.543	15.678	1.870	ABC1 family protein	AN-M
MELO3C013463.2	6.279	9.906	6.251	7.091	6.678	20.089	5.821	Vacuolar cation/proton exchanger, putative	AN-M
MELO3C013498.2	24.149	41.149	6.365	46.949	17.516	151.813	14.074	Amino acid transporter family protein	AN-M
MELO3C013558.2	0.166	0.591	1.941	0.760	0.231	2.558	0.290	Indole-3-acetic acid-amido synthetase GH3.3	AN-M
MELO3C013563.2	22.125	30.335	15.218	47.669	25.731	104.348	32.073	Metacaspase-5	AN-M
MELO3C025877.2	4.873	3.489	1.344	4.924	2.103	10.060	2.445	Glucose-6-phosphate isomerase	AN-M
MELO3C026873.2	0.804	3.196	2.612	1.979	0.854	10.547	0.589	Non-specific serine/threonine protein kinase	AN-M
MELO3C025730.2	3.107	4.686	4.896	5.550	3.506	11.673	3.334	V-type proton ATPase subunit a	AN-M
MELO3C026955.2	15.903	24.714	8.212	28.318	14.264	67.159	18.264	Superoxide dismutase [Cu-Zn]	AN-M
MELO3C021428.2	5.547	1.934	1.557	4.723	4.459	237.820	2.835	Transmembrane 9 superfamily member	AN-M
MELO3C021314.2	0.162	0.906	0.248	117.979	0.421	348.790	0.327	Protein DETOXIFICATION	AN-M
MELO3C021300.2	0.400	2.569	1.419	1.920	0.840	37.810	0.632	Protein phosphatase 2c, putative	AN-M
MELO3C021265.2	6.513	13.656	4.864	13.130	6.494	39.179	6.064	3-hydroxybutyryl-CoA dehydratase	AN-M
MELO3C021249.2	2.371	8.129	0.864	13.756	1.160	35.681	1.303	Hexosyltransferase	AN-M

Gene ID	FPKM							Gene Description	Specific in episode
	FS	GI-M	GM-M	AN-M	GI-H	GM-H	AN-H		
MELO3C022342.2	4.191	9.110	12.003	7.553	2.349	19.653	4.631	NAC domain-containing protein 83	AN-M
MELO3C022445.2	48.887	32.678	15.018	22.538	22.447	121.523	22.801	Zinc finger, B-box	AN-M
MELO3C022463.2	0.291	1.782	1.753	2.751	0.655	6.702	0.896	UMP-CMP kinase	AN-M
MELO3C022477.2	2.364	1.150	1.175	6.147	0.925	35.509	1.043	Protein nuclear fusion defective 4	AN-M
MELO3C020560.2	9.862	15.527	2.687	15.444	8.553	35.355	9.237	sodium-dependent phosphate transport protein 1, chloroplastic	AN-M
MELO3C020617.2	0.513	6.046	1.173	9.073	1.906	28.505	1.460	Haloacid dehalogenase-like hydrolase	AN-M
MELO3C035466.2	7.001	4.957	2.976	6.544	5.367	29.960	5.362	dolichol kinase EVAN	AN-M
MELO3C025563.2	8.962	15.992	2.794	8.866	5.688	219.300	5.479	tonoplast dicarboxylate transporter	AN-M
MELO3C021730.2	6.720	9.479	6.077	11.990	9.261	31.438	11.936	calcium permeable stress-gated cation channel 1-like	AN-M
MELO3C002682.2	4.922	7.310	3.391	8.338	4.351	22.371	4.852	RING/FYVE/PHD-type zinc finger protein	AN-M
MELO3C002560.2	0.606	2.206	0.595	0.790	2.431	6.511	1.858	Cotton fiber expressed protein	AN-M
MELO3C002550.2	0.235	1.321	0.593	1.591	0.320	12.155	0.633	Flowering locus T/terminal flower 1-like protein	AN-M
MELO3C002521.2	0.716	2.253	3.027	2.095	1.162	8.209	2.123	At1g11440	AN-M
MELO3C002463.2	5.216	8.278	5.889	7.323	6.131	22.520	6.588	UPF0051 protein ABCI8, chloroplastic	AN-M
MELO3C002442.2	72.587	111.618	26.548	210.095	61.974	995.280	57.229	Aspartic proteinase	AN-M
MELO3C002421.2	1.245	4.308	4.141	2.325	6.339	47.760	2.024	Caffeoylshikimate esterase	AN-M
MELO3C002297.2	23.539	33.684	23.268	24.971	21.047	74.761	24.463	protein NBR1 homolog	AN-M
MELO3C002233.2	33.457	55.391	31.224	63.469	28.132	161.778	30.901	B12d-like protein	AN-M
MELO3C002111.2	1.941	4.294	3.318	15.091	3.525	75.260	2.930	Alpha/beta hydrolase family protein	AN-M
MELO3C002105.2	0.459	1.387	1.642	0.670	1.066	3.931	0.969	LEAF RUST 10 DISEASE-RESISTANCE LOCUS RECEPTOR-LIKE PROTEIN KINASE-like 1.2 isoform X2	AN-M
MELO3C002072.2	0.216	0.847	0.805	1.023	0.160	2.478	0.045	Adenine nucleotide alpha hydrolases-like superfamily protein	AN-M
MELO3C002065.2	1.860	3.506	3.342	3.351	1.822	11.027	1.480	phospholipid-transporting ATPase 10-like	AN-M
MELO3C027060.2	5.715	5.300	3.215	15.266	4.475	62.709	8.741	Pectinesterase	AN-M
MELO3C027076.2	5.659	3.955	4.559	12.117	3.204	55.902	4.129	Bidirectional sugar transporter SWEET	AN-M
MELO3C027219.2	60.341	48.808	14.322	92.422	24.208	365.709	22.739	Hydroxycinnamoyl-CoA shikimate/quinate hydroxycinnamoyltransferase	AN-M
MELO3C027813.2	5.247	4.977	1.248	9.179	5.360	48.644	6.032	vacuolar cation/proton exchanger 3	AN-M
MELO3C027375.2	6.338	7.067	5.762	18.946	5.454	77.363	7.499	Pectinesterase	AN-M
MELO3C028357.2	0.620	0.413	2.831	1.719	0.581	15.435	0.514	Serine/threonine-protein phosphatase 7 long form-like protein	AN-M
MELO3C000391.2	0.765	0.805	0.604	2.871	NA	13.269	0.243	zinc finger protein ZAT5-like	AN-M

Gene ID	FPKM							Gene Description	Specific in episode
	FS	GI-M	GM-M	AN-M	GI-H	GM-H	AN-H		
MELO3C000450.2	23.951	33.673	4.373	41.108	14.243	182.274	10.362	IAA-amino acid hydrolase ILR1, putative	AN-M
MELO3C027159.2	0.069	0.116	0.456	0.602	0.178	7.155	0.160	Serine/threonine-protein phosphatase 7 long form-like protein	AN-M
MELO3C001609.2	4.623	10.015	1.140	27.177	1.880	86.301	1.539	Isoaspartyl peptidase/L-asparaginase	AN-M
MELO3C001055.2	26.349	32.564	5.398	47.097	23.853	103.741	25.004	Glyoxylate reductase	AN-M
MELO3C018424.2	0.979	0.631	0.446	0.370	0.278	2.521	0.411	1-aminocyclopropane-1-carboxylate oxidase	AN-M
MELO3C018480.2	2.188	3.133	1.477	6.492	2.761	48.024	4.173	Leucine-rich repeat receptor-like protein kinase family	AN-M
MELO3C018511.2	3.256	4.214	1.462	5.610	2.383	13.480	2.252	Amino acid transporter, putative	AN-M
MELO3C018584.2	0.207	0.217	0.210	1.539	0.233	5.410	0.162	Gamma-glutamyl peptidase 5	AN-M
MELO3C018713.2	62.083	55.908	14.647	84.805	44.912	438.702	53.992	ATP-dependent zinc metalloprotease FTSH 2, chloroplastic	AN-M
MELO3C018859.2	0.216	0.542	0.475	1.492	0.540	3.070	0.789	O-methyltransferase	AN-M
MELO3C018861.2	1.750	1.810	1.771	5.114	1.552	10.580	2.108	translation initiation factor IF-2 isoform X1	AN-M
MELO3C024179.2	6.442	7.126	7.185	11.228	4.564	30.036	5.604	zinc finger protein CONSTANS-LIKE 9-like	AN-M
MELO3C024074.2	1.386	1.248	1.820	2.189	0.905	9.343	1.150	Holocarboxylase synthetase	AN-M
MELO3C024962.2	10.592	12.930	2.730	17.140	9.452	34.496	11.772	Transmembrane protein C9orf5	AN-M
MELO3C024948.2	1.754	1.460	2.328	0.648	1.310	28.035	0.912	Unknown protein	AN-M
MELO3C024886.2	3.429	5.615	4.677	16.876	4.427	93.188	4.286	4-coumarate:CoA ligase	AN-M
MELO3C013215.2	6.353	6.125	2.170	22.184	4.996	52.930	1.883	Chaperone DnaJ domain protein	AN-M
MELO3C013362.2	23.769	30.649	10.834	34.628	20.089	97.518	20.845	Ascorbate peroxidase	AN-M
MELO3C012717.2	2.866	4.709	3.915	5.229	2.816	14.004	2.997	Fanconi anemia group D2 protein	AN-M
MELO3C021008.2	47.368	43.904	22.543	81.594	31.575	186.043	25.335	PPPDE thiol peptidase family protein, putative	AN-M
MELO3C021075.2	9.928	10.532	10.014	6.097	8.061	21.752	9.373	UV-B-induced protein At3g17800, chloroplastic	AN-M
MELO3C021090.2	4.463	6.195	4.759	11.244	5.005	49.700	5.767	serine/threonine-protein kinase OXI1-like	AN-M
MELO3C021130.2	5.703	6.581	3.314	29.768	6.444	59.608	7.192	ABC transporter I family member 19-like	AN-M
MELO3C015706.2	7.620	11.081	3.650	16.737	6.638	62.302	7.540	Glycerol kinase	AN-M
MELO3C028882.2	3.040	2.089	3.079	1.197	1.919	7.831	3.269	Phosphate carrier, mitochondrial	AN-M
MELO3C015762.2	0.601	0.563	6.057	3.229	0.503	9.864	0.504	Auxin-responsive protein	AN-M
MELO3C028893.2	0.580	1.755	1.502	6.056	NA	70.360	0.172	Auxin-responsive protein	AN-M
MELO3C015798.2	41.311	41.311	25.551	25.020	24.980	245.275	36.456	sulfate transporter 3.1-like	AN-M
MELO3C015880.2	27.816	33.261	8.097	15.577	13.280	67.450	14.419	Gibberellin receptor GID1A	AN-M
MELO3C015929.2	12.922	13.466	5.695	18.577	10.659	45.124	12.513	GTP-binding protein SAR1A	AN-M
MELO3C016047.2	5.371	6.502	3.159	8.352	3.758	18.690	5.736	Peroxisome biogenesis 6-like protein	AN-M

Gene ID	FPKM							Gene Description	Specific in episode
	FS	GI-M	GM-M	AN-M	GI-H	GM-H	AN-H		
MELO3C023495.2	0.035	0.060	1.225	0.328	0.111	1.464	0.082	myosin-12	AN-M
MELO3C028965.2	0.281	0.538	0.278	1.144	0.163	616.128	0.430	4-coumarate:CoA ligase	AN-M
MELO3C023493.2	1.859	2.064	1.049	4.757	0.984	65.289	0.684	4-coumarate:CoA ligase	AN-M
MELO3C023477.2	79.585	94.901	16.419	115.618	61.120	337.803	58.725	Serinc-domain containing serine and sphingolipid biosynthesis protein	AN-M
MELO3C023436.2	5.623	9.366	3.375	16.176	2.934	90.605	3.488	VQ motif-containing protein	AN-M
MELO3C023383.2	0.171	0.184	1.538	3.104	0.298	6.641	0.296	Pleiotropic drug resistance ABC transporter	AN-M
MELO3C024346.2	168.386	167.215	26.702	235.654	107.444	727.483	104.532	Subtilisin-like protease	AN-M
MELO3C024410.2	1.768	1.387	4.556	0.499	1.354	8.161	1.170	Protein EARLY FLOWERING 3	AN-M
MELO3C015302.2	2.874	4.579	4.462	7.567	5.537	59.026	2.643	Hypoxia-responsive family protein	AN-M
MELO3C015502.2	0.892	1.551	4.364	1.679	1.499	14.940	1.036	Integral membrane protein	AN-M
MELO3C015505.2	0.512	1.042	9.156	1.253	0.345	2.980	0.403	vacuolar protein sorting-associated protein 2 homolog 1	AN-M
MELO3C015552.2	4.219	5.296	4.276	7.450	6.529	29.914	7.023	sucrose synthase	AN-M
MELO3C015633.2	65.166	78.456	26.936	96.159	49.631	224.657	70.637	protein ETHYLENE INSENSITIVE 3	AN-M
MELO3C015113.2	12.677	15.589	16.019	9.728	14.423	37.014	15.576	Protein SMG7	AN-M
MELO3C010007.2	0.955	1.444	1.616	0.348	1.596	5.299	2.494	aspartic proteinase-like protein 2	AN-M
MELO3C010055.2	0.957	1.430	1.308	2.533	1.567	6.548	1.796	4-hydroxy-4-methyl-2-oxoglutarate aldolase	AN-M
MELO3C010185.2	6.245	7.898	0.720	23.427	NA	150.730	4.252	Glycine-rich protein	AN-M
MELO3C010234.2	15.412	15.208	19.436	21.594	7.683	50.309	11.791	Non-specific serine/threonine protein kinase	AN-M
MELO3C010249.2	0.706	0.683	0.374	2.053	0.778	14.914	0.924	chitotriosidase-1-like	AN-M
MELO3C010251.2	0.976	1.764	0.194	11.741	0.853	43.794	0.715	Plasma membrane ATPase	AN-M
MELO3C010261.2	14.138	17.370	5.555	34.183	8.789	86.149	7.042	Zinc finger, SWIM-type	AN-M
MELO3C010336.2	1.450	1.450	3.406	0.849	1.233	6.713	0.982	Ribosomal protein	AN-M
MELO3C026639.2	10.534	14.798	11.740	20.464	14.776	68.777	16.539	DeSI-like protein At4g17486	AN-M
MELO3C026635.2	0.391	0.531	1.999	1.823	0.565	6.324	0.298	Plant Tudor-like RNA-binding protein	AN-M
MELO3C026629.2	3.103	3.411	7.358	1.192	3.825	7.502	4.230	potassium channel AKT1	AN-M
MELO3C025308.2	2.174	1.995	3.065	0.487	1.470	14.259	1.940	auxin-responsive protein IAA16-like	AN-M
MELO3C025309.2	12.695	9.622	11.278	5.266	8.784	36.331	8.789	Acyl-[acyl-carrier-protein] hydrolase	AN-M
MELO3C025348.2	7.980	5.241	4.133	15.019	7.017	47.601	8.564	proline-rich receptor-like protein kinase PERK1	AN-M
MELO3C024674.2	4.517	6.094	2.446	17.626	3.889	51.364	4.492	Zeta-carotene desaturase	AN-M
MELO3C017501.2	11.521	10.216	5.672	3.859	2.891	29.136	3.316	zinc finger protein CONSTANS-LIKE 5	AN-M

Gene ID	FPKM							Gene Description	Specific in episode
	FS	GI-M	GM-M	AN-M	GI-H	GM-H	AN-H		
MELO3C017415.2	0.289	0.836	0.892	1.655	0.353	9.245	0.502	WRKY transcription factor, putative	AN-M
MELO3C017374.2	2.486	2.802	2.406	1.204	2.584	6.816	1.887	MAR-binding filament-like protein 1-1 isoform 2	AN-M
MELO3C017319.2	0.378	0.574	1.844	1.482	0.329	3.180	0.210	BnaA06g03540D protein	AN-M
MELO3C017305.2	1.091	1.583	0.409	4.012	0.961	20.277	1.168	LOB domain-containing protein 41	AN-M
MELO3C017222.2	1.272	2.018	2.346	2.853	0.552	7.340	0.765	LOW QUALITY PROTEIN: DNAJ protein JJJ1 homolog	AN-M
MELO3C017158.2	3.129	3.227	12.937	1.384	6.536	19.496	5.862	Serine/threonine-protein kinase atr	AN-M
MELO3C017146.2	0.764	1.412	0.629	3.318	0.665	68.738	0.576	protein NUCLEAR FUSION DEFECTIVE 4	AN-M
MELO3C017093.2	4.653	6.193	10.055	9.716	4.916	26.259	7.411	Kinesin light chain	AN-M
MELO3C008234.2	4.910	4.030	1.918	11.883	3.947	31.936	2.924	Transferring glycosyl group transferase	AN-M
MELO3C008328.2	2.940	4.861	1.661	6.091	2.335	15.433	2.101	Receptor serine-threonine protein kinase	AN-M
MELO3C008335.2	19.670	18.495	4.802	28.522	13.507	80.786	13.238	UPF0496 protein 4-like	AN-M
MELO3C008421.2	17.344	27.317	15.186	33.707	23.765	98.053	30.297	thioredoxin-like	AN-M
MELO3C010608.2	98.207	83.003	13.316	155.308	61.815	321.782	72.148	Photosystem II 22 kDa family protein	AN-M
MELO3C011682.2	47.100	48.739	16.097	78.019	44.615	195.534	45.052	Fructose-1,6-bisphosphatase	AN-M
MELO3C024990.2	0.411	1.185	1.083	1.402	0.861	3.852	0.629	Like-COV protein	AN-M
MELO3C030034.2	25.578	37.428	13.874	49.181	22.105	103.201	26.065	protein GIGANTEA-like	AN-M
MELO3C019904.2	1.829	1.531	2.299	0.548	0.229	4.329	0.683	transcription factor DIVARICATA	AN-M
MELO3C019845.2	8.144	10.897	10.414	15.443	6.298	44.579	7.871	NAC domain-containing protein 78-like isoform X2	AN-M
MELO3C026529.2	0.492	0.749	0.242	7.687	0.360	121.443	0.317	glycosyltransferase family 92 protein At1g27200	AN-M
MELO3C026549.2	1.391	1.855	1.393	3.316	2.376	10.056	1.446	NADH-ubiquinone oxidoreductase chain 5	AN-M
MELO3C011471.2	6.344	7.043	3.997	10.625	5.294	21.524	6.348	Protein PLASTID MOVEMENT IMPAIRED 1-RELATED 1	AN-M
MELO3C011435.2	0.875	0.840	0.668	2.204	0.852	16.117	0.966	ABC transporter G family member 29-like	AN-M
MELO3C011401.2	0.341	0.784	6.964	0.904	1.200	2.019	0.710	18S pre-ribosomal assembly protein gar2-like protein	AN-M
MELO3C011340.2	2.239	4.483	7.035	7.143	2.786	22.271	4.664	thioredoxin-like protein CDSP32, chloroplastic	AN-M
MELO3C011320.2	16.704	17.912	7.735	28.009	14.410	84.183	14.836	NAD(P)-binding rossmann-fold protein	AN-M
MELO3C011315.2	9.604	10.352	5.427	14.888	7.499	34.038	6.717	protein ENHANCED DISEASE RESISTANCE 2	AN-M
MELO3C011124.2	1.683	3.804	4.897	4.395	4.474	25.597	2.623	Early nodulin 93	AN-M
MELO3C011078.2	2.354	2.215	1.950	0.718	1.267	4.793	1.698	GATA transcription factor, putative	AN-M
MELO3C011025.2	11.788	10.563	3.818	17.067	9.758	66.108	9.023	Transmembrane protein	AN-M
MELO3C030111.2	1.250	1.890	0.674	3.563	3.679	9.122	3.841	Thaumatin-like protein 1	AN-M
MELO3C030112.2	47.070	47.256	20.719	104.773	51.496	237.134	59.684	V-type proton ATPase proteolipid subunit	AN-M

Gene ID	FPKM							Gene Description	Specific in episode
	FS	GI-M	GM-M	AN-M	GI-H	GM-H	AN-H		
MELO3C010990.2	8.807	6.918	4.610	14.408	11.906	45.617	10.694	1-acylglycerol-3-phosphate O-acyltransferase	AN-M
MELO3C010979.2	1.209	1.771	1.064	6.687	2.027	25.442	4.463	WAT1-related protein	AN-M
MELO3C010946.2	14.899	15.189	3.986	61.050	18.535	376.329	15.999	sugar transporter ERD6-like 6	AN-M
MELO3C010938.2	16.989	16.745	13.487	7.031	10.556	39.586	11.444	Erythronate-4-phosphate dehydrogenase family protein	AN-M
MELO3C010765.2	38.581	24.697	26.476	98.348	55.173	260.534	55.206	Unknown protein	AN-M
MELO3C010763.2	56.567	72.694	12.721	122.268	37.006	423.536	36.021	vacuolar-processing enzyme-like	AN-M
MELO3C010751.2	1.455	1.902	1.623	32.485	1.875	130.956	1.702	LOW QUALITY PROTEIN: beta-fructofuranosidase, insoluble isoenzyme CWINV3-like	AN-M
MELO3C010667.2	5.031	4.557	5.474	3.274	4.002	10.208	4.755	Phosphatidylinositol 3-kinase	AN-M
MELO3C003331.2	2.649	2.048	0.550	0.646	0.996	14.485	0.448	BAX inhibitor-1	AN-M
MELO3C003339.2	1.111	2.540	3.600	3.725	0.929	9.097	0.908	Calcineurin B-like protein	AN-M
MELO3C003439.2	11.313	12.141	4.118	17.769	11.077	106.143	10.369	NAD(P)-binding rossmann-fold protein	AN-M
MELO3C003441.2	2.435	3.091	2.347	5.813	6.126	55.343	4.730	Xyloglucan endotransglucosylase/hydrolase	AN-M
MELO3C003491.2	9.070	12.720	4.036	18.640	4.743	55.384	5.382	Phosphoenolpyruvate carboxykinase	AN-M
MELO3C003538.2	36.490	37.300	7.243	54.917	20.936	132.864	24.911	TRANSPORT INHIBITOR RESPONSE 1 protein, putative	AN-M
MELO3C003571.2	17.479	17.644	14.847	12.661	12.483	37.785	14.982	Protein phosphatase 1 regulatory subunit pprA	AN-M
MELO3C003597.2	1.293	1.887	4.134	2.489	0.662	11.300	1.070	ABC1 family protein	AN-M
MELO3C003701.2	1.267	1.476	2.817	0.246	2.286	6.592	4.013	Auxin-responsive protein	AN-M
MELO3C003714.2	0.159	0.116	0.085	1.627	NA	5.268	NA	ACT domain-containing protein ACR2	AN-M
MELO3C003739.2	11.900	15.448	4.860	18.537	12.213	62.095	14.159	Zinc finger protein	AN-M
MELO3C003846.2	27.769	35.391	14.901	69.424	36.067	155.360	39.587	Glucan endo-1,3-beta-glucosidase, putative	AN-M
MELO3C003878.2	0.858	0.666	1.482	2.185	0.926	80.303	1.334	J-domain protein required for chloroplast accumulation response 1	AN-M
MELO3C012791.2	8.152	5.028	4.023	41.901	3.988	177.314	2.895	Pectate lyase	AN-M
MELO3C012874.2	2.683	1.999	2.635	6.471	3.426	13.202	3.685	Arabinose 5-phosphate isomerase, putative	AN-M
MELO3C012917.2	18.808	16.277	9.154	40.899	32.571	133.232	45.668	Boron transporter, putative	AN-M
MELO3C013007.2	5.215	8.732	5.050	18.876	7.167	113.802	8.443	profilin-like	AN-M
MELO3C022759.2	7.804	7.692	3.070	11.015	8.634	42.000	7.546	MOB kinase activator-like 1A	AN-M
MELO3C026705.2	4.803	5.431	5.154	9.104	4.270	72.878	3.748	Vesicle-associated membrane protein, putative	AN-M
MELO3C024005.2	12.118	8.999	3.782	24.590	12.357	61.552	11.218	Acyl-CoA--sterol O-acyltransferase 1	AN-M
MELO3C023947.2	3.307	4.841	9.822	15.917	4.680	58.708	4.027	O-fucosyltransferase family protein	AN-M
MELO3C026901.2	5.643	8.599	3.747	9.012	6.046	25.641	4.413	HNH endonuclease	AN-M

Gene ID	FPKM							Gene Description	Specific in episode
	FS	GI-M	GM-M	AN-M	GI-H	GM-H	AN-H		
MELO3C026897.2	14.331	15.902	6.661	7.517	8.147	114.207	10.579	thioredoxin-like 1-1, chloroplastic	AN-M
MELO3C026613.2	152.945	171.867	39.380	274.617	141.420	566.682	149.243	Tubulin alpha chain	AN-M
MELO3C026594.2	3.021	6.253	4.280	11.273	1.748	36.160	1.873	E3 ubiquitin-protein ligase RGLG2	AN-M
MELO3C009923.2	1.045	1.437	1.392	3.218	NA	38.420	0.246	zinc finger AN1 domain-containing stress-associated protein 12	AN-M
MELO3C009912.2	22.239	23.450	22.503	33.653	27.894	75.289	37.133	protein transport protein SEC13 homolog B	AN-M
MELO3C009911.2	6.455	6.906	2.976	11.062	4.486	681.097	6.028	Hop-interacting protein THI043	AN-M
MELO3C030704.2	4.015	1.561	0.336	1.255	NA	30.440	0.402	F-box protein CPR30	AN-M
MELO3C009692.2	7.196	4.421	1.824	4.269	1.953	23.567	2.707	protein PHYTOCHROME KINASE SUBSTRATE 4	AN-M
MELO3C009679.2	3.147	3.894	1.098	5.061	2.099	25.418	2.001	Alpha/beta hydrolase-1	AN-M
MELO3C009648.2	4.428	4.478	1.564	9.724	5.270	56.410	5.643	Alpha/beta hydrolase family protein	AN-M
MELO3C009568.2	28.291	33.945	10.649	49.615	25.977	155.804	29.793	E3 ubiquitin-protein ligase RHF2A-like	AN-M
MELO3C009512.2	3.116	1.229	5.919	0.410	3.970	12.499	3.264	Gb|AAF02129.1	AN-M
MELO3C009504.2	5.891	5.665	3.154	9.965	4.955	40.651	2.217	IAA-amino acid hydrolase ILR1-like 8	AN-M
MELO3C009485.2	7.629	5.187	4.152	24.545	8.244	240.803	7.726	GATA zinc finger protein	AN-M
MELO3C009484.2	1.483	2.091	0.898	5.606	0.899	18.153	1.369	GATA zinc finger protein	AN-M
MELO3C009438.2	32.927	36.634	9.554	214.377	41.448	492.565	36.025	Fructose-bisphosphate aldolase	AN-M
MELO3C009405.2	1.257	2.049	0.442	3.422	0.381	22.174	0.778	purine-uracil permease NCS1	AN-M
MELO3C009386.2	4.047	4.765	1.263	18.188	1.856	142.993	2.556	UDP-glycosyltransferase 73C6-like	AN-M
MELO3C009365.2	14.755	13.583	5.092	21.275	10.700	52.879	11.149	FAD synthase	AN-M
MELO3C009363.2	2.725	2.603	1.267	8.782	2.934	35.190	2.136	Unknown protein	AN-M
MELO3C009362.2	0.558	0.942	1.657	2.297	0.525	8.968	0.556	4-coumarate--CoA ligase-like 7	AN-M
MELO3C009355.2	0.619	0.899	5.210	2.100	0.357	5.564	0.733	heavy metal-associated isoprenylated plant protein 3-like	AN-M
MELO3C009322.2	0.197	0.197	0.211	1.471	0.263	5.079	0.198	Glycosyltransferases	AN-M
MELO3C009227.2	14.147	15.167	8.724	21.793	12.583	59.477	13.079	PRA1 family protein	AN-M
MELO3C009205.2	4.410	6.731	2.185	10.938	5.442	43.874	3.381	PAR1 protein	AN-M
MELO3C009199.2	0.432	0.900	2.713	1.980	1.030	28.322	2.289	O-fucosyltransferase family protein	AN-M
MELO3C009190.2	4.957	7.199	1.452	35.333	3.606	105.521	2.194	LOW QUALITY PROTEIN: protein NRT1/ PTR FAMILY 8.1	AN-M
MELO3C009187.2	25.870	26.237	10.767	44.771	11.467	141.600	17.440	Transferase family protein	AN-M
MELO3C009181.2	19.074	17.808	5.920	69.685	9.156	269.452	12.704	Auxin transporter-like protein 2	AN-M
MELO3C009151.2	5.513	8.479	8.206	9.804	12.476	20.169	7.356	phosphoinositide phosphatase SAC8	AN-M
MELO3C009138.2	1.371	1.271	1.889	0.834	1.598	5.553	0.825	Receptor lectin protein kinase-like	AN-M

Gene ID	FPKM							Gene Description	Specific in episode
	FS	GI-M	GM-M	AN-M	GI-H	GM-H	AN-H		
MELO3C014719.2	0.405	0.680	1.875	1.931	0.900	5.941	0.660	Aldehyde oxidase, putative	AN-M
MELO3C014679.2	5.752	5.322	4.059	9.901	6.316	23.541	5.306	Lipid phosphate phosphatase gamma, chloroplastic	AN-M
MELO3C014673.2	3.776	3.988	2.539	6.441	2.678	19.426	2.055	TSA: Wollemia nobilis Ref_Wollemi_Transcript_7484_1220 transcribed RNA sequence	AN-M
MELO3C014556.2	0.087	0.076	0.490	0.695	0.054	2.560	0.178	Calcium dependent protein kinase	AN-M
MELO3C014487.2	5.673	5.887	6.093	10.465	7.889	24.791	8.896	Chloride channel protein	AN-M
MELO3C014481.2	20.585	26.277	18.009	8.074	2.475	203.372	3.086	Haloacid dehalogenase-like hydrolase (HAD) superfamily protein	AN-M
MELO3C014363.2	2.140	2.085	1.339	9.010	1.448	49.168	1.572	F-box protein family	AN-M
MELO3C014361.2	5.516	7.475	5.469	11.630	3.273	25.811	3.675	T-complex protein 11	AN-M
MELO3C014338.2	2.007	2.638	2.413	3.378	2.480	7.084	2.739	E3 ubiquitin protein ligase RIN2	AN-M
MELO3C014329.2	33.262	32.953	21.895	46.463	24.094	148.970	27.262	ETO1-like protein 1	AN-M
MELO3C014269.2	0.817	0.382	0.457	2.520	0.650	23.360	0.772	Non-specific serine/threonine protein kinase	AN-M
MELO3C023004.2	9.448	10.494	9.977	18.905	10.740	73.855	13.994	casein kinase I-like isoform X1	AN-M
MELO3C023032.2	0.344	0.520	4.254	1.072	0.497	2.323	0.415	Cyclopropane-fatty-acyl-phospholipid synthase family protein	AN-M
MELO3C023045.2	0.987	1.334	1.256	2.993	1.621	6.028	1.086	Calcium-dependent lipid-binding (CaLB domain) family protein	AN-M
MELO3C020465.2	12.317	8.946	4.296	23.169	12.072	77.222	15.786	ABC1 family protein	AN-M
MELO3C008659.2	2.478	3.798	0.427	5.829	1.102	21.886	1.840	Ammonium transporter	AN-M
MELO3C008749.2	7.644	8.198	3.638	11.904	7.915	54.207	8.135	CBS domain-containing protein CBSX6	AN-M
MELO3C003976.2	0.353	0.690	0.966	0.797	0.403	6.201	0.461	Clathrin assembly protein, putative	AN-M
MELO3C004110.2	26.688	25.301	12.346	51.314	17.409	104.442	15.046	S-adenosylmethionine decarboxylase proenzyme-like	AN-M
MELO3C004224.2	30.443	35.931	12.181	110.788	40.605	237.622	32.328	Rapid alkalinization factor	AN-M
MELO3C004253.2	0.896	1.109	0.659	4.016	0.959	10.488	1.069	Lipoxygenase	AN-M
MELO3C004383.2	13.442	15.850	4.627	28.836	4.551	238.970	3.873	Aldehyde dehydrogenase family 2 member	AN-M
MELO3C004393.2	5.033	4.291	3.566	9.164	5.732	20.587	6.290	receptor-like protein kinase ANXUR2	AN-M
MELO3C004405.2	4.397	4.740	3.547	6.653	4.492	25.547	4.957	Myosin-binding protein 1	AN-M
MELO3C004435.2	17.006	11.726	6.480	8.978	13.754	39.051	19.783	Transmembrane protein	AN-M
MELO3C004504.2	7.332	8.001	15.986	3.774	3.294	19.081	3.002	SPX domain-containing protein 1	AN-M
MELO3C004595.2	33.083	40.062	67.418	44.937	38.002	105.612	43.267	profilin	AN-M
MELO3C004597.2	1.652	1.725	1.499	3.925	3.091	8.345	4.192	DUF1005 family protein (DUF1005)	AN-M
MELO3C004632.2	5.181	4.638	3.426	11.385	4.734	24.596	5.442	O-acyltransferase	AN-M
MELO3C004667.2	3.701	3.354	0.815	5.498	2.115	29.036	2.115	pollen receptor-like kinase 3	AN-M

Gene ID	FPKM							Gene Description	Specific in episode
	FS	GI-M	GM-M	AN-M	GI-H	GM-H	AN-H		
MELO3C005959.2	4.314	4.246	6.813	10.930	5.642	23.131	4.827	protein transport protein Sec61 subunit beta	AN-M
MELO3C005991.2	1.111	3.246	0.419	7.714	0.275	26.942	0.703	Auxin-responsive family protein	AN-M
MELO3C006000.2	1.846	2.230	1.531	3.694	1.420	24.676	1.310	AAA-ATPase At2g46620	AN-M
MELO3C006004.2	1.485	2.193	2.539	2.951	1.204	8.444	1.423	Kinase, putative	AN-M
MELO3C006060.2	3.954	5.509	2.347	6.411	2.535	37.469	4.245	Mitochondrial carrier protein	AN-M
MELO3C006090.2	8.990	8.151	5.785	24.393	13.132	79.143	13.407	Receptor-like kinase	AN-M
MELO3C006388.2	0.617	1.133	0.854	2.410	0.653	7.826	0.518	F19P19.6 protein	AN-M
MELO3C006427.2	3.693	5.612	0.510	19.092	1.884	94.014	1.074	Phosphate transporter	AN-M
MELO3C006592.2	2.481	3.131	2.526	5.051	4.081	14.780	4.054	Prolyl 4-hydroxylase alpha subunit, putative	AN-M
MELO3C006710.2	33.980	37.881	10.225	51.222	29.356	193.301	28.502	Ubiquitin-conjugating enzyme, E2	AN-M
MELO3C006727.2	24.484	29.969	24.847	5.052	11.164	66.724	12.494	Neutral/alkaline invertase	AN-M
MELO3C006734.2	5.425	5.648	1.961	11.750	5.725	23.532	6.305	Dihydrolipoyl dehydrogenase	AN-M
MELO3C006875.2	1.392	1.963	4.868	3.750	2.395	9.282	0.708	BAG family molecular chaperone regulator 1-like	AN-M
MELO3C006972.2	17.696	19.867	14.255	29.888	15.955	68.662	13.704	Ubiquitin-conjugating enzyme, E2	AN-M
MELO3C019480.2	1.428	1.988	1.593	2.882	2.484	7.269	1.428	Hydroxyproline-rich glycoprotein family protein, putative	AN-M
MELO3C019505.2	2.826	3.378	3.929	7.325	2.742	22.299	4.772	Ferredoxin--nitrite reductase family protein	AN-M
MELO3C019564.2	14.370	10.893	9.796	8.414	9.429	46.420	12.903	High chlorophyll fluorescence phenotype 173	AN-M
MELO3C020241.2	5.484	5.709	2.053	9.674	5.312	25.610	4.636	PC-Esterase	AN-M
MELO3C020230.2	283.770	310.256	83.183	758.104	210.297	1845.145	218.204	Glutamine synthetase	AN-M
MELO3C020229.2	3.136	3.831	1.058	7.969	2.979	26.824	2.942	Receptor-like kinase	AN-M
MELO3C014969.2	6.650	8.811	2.557	27.018	7.906	83.329	8.937	receptor-like cytosolic serine/threonine-protein kinase RBK1	AN-M
MELO3C014824.2	34.556	41.392	14.689	77.077	34.642	343.873	33.781	Succinate dehydrogenase [ubiquinone] iron-sulfur subunit, mitochondrial	AN-M
MELO3C014794.2	10.737	10.432	8.358	6.081	9.130	33.096	7.181	Ras-related protein	AN-M
MELO3C014764.2	1.942	3.329	1.140	4.349	1.195	10.552	1.983	nudix hydrolase 13, mitochondrial	AN-M
MELO3C016623.2	16.066	16.077	7.894	32.706	9.668	67.135	8.904	NAD(P)H dehydrogenase (Quinone)	AN-M
MELO3C016475.2	2.691	3.610	2.153	7.958	2.217	24.027	2.787	Chloride channel protein	AN-M
MELO3C016426.2	2.155	2.844	0.562	4.425	1.759	30.188	2.965	ferric reduction oxidase 7, chloroplastic	AN-M
MELO3C013726.2	0.131	0.277	1.279	0.849	0.140	1.799	0.077	RB1-inducible coiled-coil protein	AN-M
MELO3C013745.2	35.432	28.591	10.510	107.404	28.236	226.691	34.220	Phosphatase 2C family protein	AN-M
MELO3C013876.2	4.654	6.739	3.967	11.117	7.678	24.878	6.833	Vacuolar protein sorting-associated protein 55-like protein	AN-M

Gene ID	FPKM							Gene Description	Specific in episode
	FS	GI-M	GM-M	AN-M	GI-H	GM-H	AN-H		
MELO3C013883.2	0.755	0.774	0.859	3.225	0.138	11.061	0.919	BOI-related E3 ubiquitin-protein ligase 1	AN-M
MELO3C013907.2	25.600	19.326	7.589	81.748	18.632	206.531	18.477	glycine-rich cell wall structural protein 1-like	AN-M
MELO3C013910.2	3.343	3.365	7.186	8.266	3.361	16.829	3.459	Ras-related GTP-binding protein	AN-M
MELO3C013964.2	9.771	13.329	3.416	17.412	10.968	39.784	10.465	Flavin-containing monooxygenase	AN-M
MELO3C013969.2	2.004	2.055	1.627	3.939	1.109	44.009	0.971	Trehalose 6-phosphate phosphatase	AN-M
MELO3C013976.2	14.037	11.925	4.849	23.093	6.622	123.387	7.667	Lipid transfer protein	AN-M
MELO3C014055.2	5.686	6.951	3.111	12.722	6.090	51.472	5.300	F-box protein PP2-A13	AN-M
MELO3C014168.2	2.626	4.089	2.382	5.949	3.091	12.891	3.278	At1g11930/F12F1_20	AN-M
MELO3C014180.2	4.637	5.703	3.901	9.590	4.191	30.277	5.628	EH domain-containing protein 1	AN-M
MELO3C014181.2	0.985	1.498	0.813	2.684	0.313	10.817	1.180	ethylene-responsive transcription factor 3	AN-M
MELO3C017042.2	6.915	7.214	5.781	10.311	5.277	24.908	7.833	Protein phosphatase 2c, putative	AN-M
MELO3C017011.2	34.743	26.122	11.515	54.528	28.090	246.272	30.609	ATP-dependent zinc metalloprotease FTSH, chloroplastic	AN-M
MELO3C016967.2	9.799	10.975	3.858	14.686	13.304	32.337	16.150	Non-specific serine/threonine protein kinase	AN-M
MELO3C016951.2	5.787	8.051	6.907	32.147	6.989	104.589	5.666	Glutathione peroxidase	AN-M
MELO3C016942.2	9.696	14.465	4.870	16.771	11.580	72.572	7.682	aspartyl protease family protein At5g10770-like	AN-M
MELO3C016939.2	18.187	17.505	7.618	27.471	14.768	59.197	17.213	Protein phosphatase 2c, putative	AN-M
MELO3C016930.2	35.220	44.768	9.816	57.832	31.377	122.496	27.331	3-ketoacyl-CoA synthase	AN-M
MELO3C016881.2	5.973	4.985	2.378	15.754	3.514	37.765	4.923	Cytokinin riboside 5'-monophosphate phosphoribohydrolase	AN-M
MELO3C016861.2	25.497	26.582	7.583	46.205	17.731	94.582	19.046	NAD(P)H-quinone oxidoreductase subunit K, chloroplastic	AN-M
MELO3C016781.2	106.208	130.240	33.622	145.849	86.532	702.382	81.513	Cysteine proteinase	AN-M
MELO3C016773.2	2.031	2.244	1.502	4.257	2.937	21.368	4.158	Transferase	AN-M
MELO3C016767.2	0.969	1.293	1.540	0.340	1.133	2.800	1.081	protein CUP-SHAPED COTYLEDON 3-like	AN-M
MELO3C016696.2	7.097	7.806	15.099	9.123	6.751	21.564	5.996	PROTON PUMP INTERACTOR 1 family protein	AN-M
MELO3C016693.2	5.001	5.624	6.936	11.024	4.980	22.107	5.534	2,3-diketo-5-methylthio-1-phosphopentane phosphatase	AN-M
MELO3C026410.2	2.279	2.202	5.229	0.741	1.751	4.873	3.167	transcription factor PIF4-like	AN-M
MELO3C010517.2	13.572	11.117	4.125	22.423	7.137	64.582	8.752	WAT1-related protein	AN-M
MELO3C010508.2	1.056	1.739	1.257	2.598	1.707	27.382	1.751	1-aminocyclopropane-1-carboxylate oxidase	AN-M
MELO3C010506.2	14.246	18.222	6.797	31.162	13.878	89.561	12.998	Amino acid permease	AN-M
MELO3C010463.2	0.648	1.308	0.435	2.046	0.446	4.193	0.879	E3 ubiquitin-protein ligase RING1-like isoform X1	AN-M
MELO3C010412.2	0.128	0.120	0.100	0.512	0.159	7.710	0.156	pollen receptor-like kinase 4	AN-M
MELO3C018969.2	8.504	11.523	2.583	14.086	6.531	30.193	6.789	L-arabinokinase	AN-M

Gene ID	FPKM							Gene Description	Specific in episode
	FS	GI-M	GM-M	AN-M	GI-H	GM-H	AN-H		
MELO3C019004.2	3.022	2.099	0.615	1.170	0.708	7.270	1.307	ENTH/ANTH/VHS superfamily protein, putative	AN-M
MELO3C027020.2	23.684	29.678	8.182	57.546	18.257	608.846	19.669	Ubiquinol oxidase	AN-M
MELO3C016140.2	8.049	6.978	4.497	3.373	6.636	24.161	5.383	Solanesyl diphosphate synthase, putative	AN-M
MELO3C016159.2	2.271	2.892	4.031	4.040	1.774	9.681	1.326	polyadenylate-binding protein-interacting protein 9 isoform X1	AN-M
MELO3C016210.2	15.485	19.325	7.869	134.287	19.683	511.770	21.583	Pectin acetylesterase	AN-M
MELO3C016232.2	17.785	12.548	9.141	11.505	14.802	158.382	18.542	BTB/POZ-like protein	AN-M
MELO3C016298.2	25.762	33.624	39.389	19.086	29.815	89.195	25.131	Ribosome maturation factor	AN-M
MELO3C016370.2	5.761	5.567	3.450	3.307	5.359	21.266	3.529	B-box zinc finger protein 18-like	AN-M
MELO3C016399.2	5.269	4.144	7.100	12.611	3.106	43.120	4.171	Mitogen-activated protein kinase	AN-M
MELO3C017547.2	0.344	0.723	0.211	2.756	0.344	7.118	0.659	GRAS family transcription factor	AN-M
MELO3C017557.2	0.095	0.160	0.028	2.106	0.024	6.326	0.086	proline-rich receptor-like protein kinase PERK4	AN-M
MELO3C017559.2	3.520	3.952	3.310	8.731	3.280	21.305	3.537	cytochrome P450 90B1	AN-M
MELO3C032841.2	158.358	185.627	31.701	373.770	236.719	749.355	198.141	chitinase-like protein 1	AN-M
MELO3C017677.2	121.202	107.735	38.334	211.788	153.878	471.435	129.860	chitinase-like protein 1	AN-M
MELO3C017683.2	6.065	6.128	8.379	8.846	5.780	17.825	6.345	Clathrin interactor EPSIN 2	AN-M
MELO3C017693.2	28.515	25.719	8.544	47.854	22.431	193.872	28.473	Receptor-like kinase	AN-M
MELO3C017722.2	5.081	6.623	2.856	8.876	4.331	22.409	6.399	histidine protein methyltransferase 1 homolog	AN-M
MELO3C017795.2	1.941	2.715	1.821	4.153	2.716	14.974	2.670	Inositol polyphosphate multikinase	AN-M
MELO3C017829.2	2.217	2.544	2.449	4.275	1.964	72.877	2.510	Sterol-8,7-isomerase	AN-M
MELO3C017850.2	23.243	21.087	5.607	40.685	13.471	178.775	15.921	protein NUCLEAR FUSION DEFECTIVE 4-like	AN-M
MELO3C017982.2	5.179	3.699	3.211	3.360	3.454	13.990	5.508	Gibberellin-regulated family protein	AN-M
MELO3C007009.2	10.389	8.460	4.456	7.127	6.744	29.722	7.911	Metal-dependent hydrolase	AN-M
MELO3C007031.2	1.547	1.608	1.805	3.570	2.374	9.983	1.235	Polygalacturonase QRT3	AN-M
MELO3C007171.2	1.852	2.229	1.314	5.704	2.031	11.774	1.238	AWPM-19-like membrane family protein	AN-M
MELO3C007218.2	11.287	10.821	3.293	24.731	8.139	82.095	6.893	50S ribosomal protein L34, chloroplastic	AN-M
MELO3C007264.2	2.833	2.432	2.106	8.139	6.256	353.155	5.524	Transmembrane protein, putative	AN-M
MELO3C032886.2	0.094	0.211	0.133	0.908	NA	56.432	NA	squamosa promoter-binding protein 1	AN-M
MELO3C007281.2	12.878	13.621	5.566	20.978	9.070	52.600	8.989	Transmembrane protein, putative	AN-M
MELO3C007418.2	11.358	13.992	5.288	20.439	7.642	71.088	9.921	LOW QUALITY PROTEIN: trihelix transcription factor ASIL2	AN-M
MELO3C007425.2	1.199	0.638	14.200	3.852	0.585	22.647	0.711	1-aminocyclopropane-1-carboxylate oxidase 1	AN-M
MELO3C007462.2	0.802	1.237	0.899	3.529	NA	7.359	1.199	glucan endo-1,3-beta-glucosidase 8	AN-M

Gene ID	FPKM							Gene Description	Specific in episode
	FS	GI-M	GM-M	AN-M	GI-H	GM-H	AN-H		
MELO3C007480.2	0.646	0.800	5.837	2.133	1.960	5.415	1.107	Cytochrome P450 family protein	AN-M
MELO3C007484.2	5.668	5.002	5.881	3.156	6.383	36.351	4.743	RPM1-interacting protein 4 (RIN4) family protein	AN-M
MELO3C007511.2	5.686	5.759	5.352	3.632	4.882	14.878	4.833	wall-associated receptor kinase-like 14	AN-M
MELO3C007572.2	3.017	2.765	13.704	4.709	5.661	12.140	6.549	AP2-like ethylene-responsive transcription factor TOE3	AN-M
MELO3C007744.2	32.281	28.056	8.572	127.681	28.321	2422.887	24.899	Phospho-2-dehydro-3-deoxyheptonate aldolase	AN-M
MELO3C007806.2	0.405	0.263	0.271	1.409	0.360	10.834	0.826	glycerophosphodiester phosphodiesterase GDPDL6-like	AN-M
MELO3C007908.2	10.763	12.900	7.714	5.040	4.746	34.000	4.444	DnaJ	AN-M
MELO3C024583.2	1.630	1.762	0.868	8.000	NA	19.974	0.480	NDR1/HIN1-like protein 12	AN-M
MELO3C024536.2	14.940	20.004	5.646	31.050	10.055	85.032	7.297	Uridine nucleosidase 1	AN-M
MELO3C024527.2	23.395	29.775	18.469	42.308	25.609	87.425	27.773	Monodehydroascorbate reductase family protein	AN-M
MELO3C024504.2	10.721	9.812	7.606	21.453	12.624	221.030	19.813	Sulfite exporter TauE/SafE family protein	AN-M
MELO3C020750.2	0.442	1.142	1.304	1.210	1.241	2.572	1.241	Ribosome-binding factor A, putative	AN-M
MELO3C020744.2	16.214	15.655	8.781	30.877	9.985	97.343	16.979	Lycopene beta cyclase	AN-M
MELO3C019087.2	1.913	2.521	1.331	3.880	1.630	23.967	2.039	UDP-glycosyltransferase 76E2-like	AN-M
MELO3C019132.2	1.315	1.709	1.196	4.517	0.938	18.493	1.644	phosphatidylinositol 4-kinase gamma 8	AN-M
MELO3C023150.2	9.591	7.950	6.021	14.468	7.855	37.151	7.694	guanylate kinase 2-like	AN-M
MELO3C009010.2	0.627	0.751	0.379	1.363	0.642	13.684	0.572	serine/threonine-protein kinase RHS3-like	AN-M
MELO3C008990.2	8.018	7.828	3.426	13.705	6.819	44.655	6.229	Lipid phosphate phosphatase delta	AN-M
MELO3C008941.2	2.532	2.642	3.435	3.506	2.359	7.013	2.368	Auxilin-related protein 1	AN-M
MELO3C008914.2	3.628	3.096	1.715	5.548	2.270	12.682	2.228	rhomboid-like protein 14, mitochondrial	AN-M
MELO3C008875.2	3.483	4.017	11.126	5.828	3.992	13.152	4.382	Ulp1 protease family C-terminal catalytic domain containing protein expressed	AN-M
MELO3C008791.2	10.112	14.379	7.430	28.077	7.921	179.822	11.003	Hydroxyproline-rich glycoprotein family protein isoform 1	AN-M
MELO3C014229.2	1.833	1.646	0.776	6.610	1.733	155.564	1.778	Phenylalanine ammonia-lyase	AN-M
MELO3C003120.2	17.891	19.592	13.119	24.532	15.061	114.755	17.561	SLT1	AN-M
MELO3C003294.2	133.584	114.544	43.060	209.057	130.483	1049.666	131.009	vacuolar cation/proton exchanger 3	AN-M
MELO3C022213.2	5.061	6.728	4.242	46.851	3.024	142.104	3.789	Myb family transcription factor family protein	AN-M
MELO3C022020.2	22.016	22.035	10.311	51.553	17.044	144.989	19.349	Cyclic nucleotide-gated ion channel, putative	AN-M
MELO3C021972.2	6.779	5.953	1.674	11.896	3.006	98.028	4.277	Plasma membrane ATPase	AN-M
MELO3C021520.2	0.224	0.389	1.216	0.617	0.363	3.722	0.247	WAT1-related protein	AN-M
MELO3C021545.2	13.630	11.827	5.594	7.971	16.214	35.901	18.623	L-aspartate oxidase	AN-M

Gene ID	FPKM							Gene Description	Specific in episode
	FS	GI-M	GM-M	AN-M	GI-H	GM-H	AN-H		
MELO3C021648.2	5.141	9.412	2.308	15.083	1.300	32.138	2.194	Heat shock 70 kDa protein	AN-M
MELO3C025473.2	9.125	8.361	3.915	14.659	7.668	45.213	9.769	sulfate transporter 4.1, chloroplastic-like	AN-M
MELO3C002891.2	6.108	8.627	2.402	9.684	4.818	29.812	5.696	Pseudouridine synthase, RsuA/RluB/C/D/E/F	AN-M
MELO3C002922.2	4.344	4.861	2.480	7.328	3.778	19.093	4.831	Purple acid phosphatase	AN-M
MELO3C002929.2	5.761	8.199	1.911	11.875	8.254	24.294	2.916	RING zinc finger protein-like	AN-M
MELO3C002981.2	3.639	4.423	2.583	7.044	3.942	15.466	3.133	transmembrane emp24 domain-containing protein p24delta9-like	AN-M
MELO3C022820.2	0.459	0.821	0.683	0.855	0.110	2.703	0.203	potassium channel SKOR	AN-M
MELO3C005130.2	0.297	0.753	1.097	0.875	0.408	2.708	0.735	Cytochrome C oxidase subunit	AN-M
MELO3C005243.2	0.259	0.586	0.536	1.289	0.218	6.966	0.347	purple acid phosphatase 4-like	AN-M
MELO3C005247.2	24.089	21.010	8.055	12.638	17.759	66.768	17.987	Protein OBERON 3	AN-M
MELO3C005261.2	0.413	0.713	0.366	1.606	0.535	5.334	0.631	Serine/threonine-protein kinase	AN-M
MELO3C005300.2	103.482	141.991	51.072	228.226	123.411	638.674	99.473	BnaC07g20870D protein	AN-M
MELO3C005301.2	10.933	19.293	12.041	27.512	12.434	62.473	12.419	At1g70780	AN-M
MELO3C005326.2	3.674	5.209	1.184	19.988	2.455	56.781	3.591	Auxin efflux carrier component	AN-M
MELO3C005363.2	3.274	2.347	1.345	36.238	3.234	173.933	3.153	Acid beta-fructofuranosidase	AN-M
MELO3C005383.2	21.800	23.441	16.160	10.883	9.035	75.085	13.940	Glycerol-3-phosphate transporter	AN-M
MELO3C005432.2	10.875	8.974	4.113	19.049	8.746	77.249	10.206	Rubredoxin family protein	AN-M
MELO3C005448.2	5.814	6.256	4.093	8.788	4.939	25.722	6.222	Replicase polyprotein 1ab	AN-M
MELO3C005503.2	7.406	8.829	5.068	11.330	10.420	31.154	8.926	DeSI-like protein At4g17486	AN-M
MELO3C005526.2	30.960	35.653	6.781	48.945	57.936	169.669	85.913	Aquaporin 1	AN-M
MELO3C005571.2	1.558	2.109	0.608	13.259	0.832	96.068	1.433	Cytochrome P450 family protein	AN-M
MELO3C005577.2	1.247	2.302	1.102	14.293	0.613	98.667	1.130	Amine oxidase	AN-M
MELO3C005579.2	6.443	9.984	11.390	13.443	4.918	50.090	5.130	Maspardin	AN-M
MELO3C005595.2	47.618	49.997	13.831	66.099	46.939	199.440	39.442	zinc finger A20 and AN1 domain-containing stress-associated protein 1-like	AN-M
MELO3C005620.2	4.244	5.864	1.855	6.754	2.206	16.490	1.850	Homeobox protein knotted-1, putative	AN-M
MELO3C005686.2	7.458	5.446	2.631	20.841	6.762	44.297	8.845	Cytochrome P450 family protein	AN-M
MELO3C005745.2	16.079	13.164	6.528	10.641	18.801	33.021	16.455	Abhydrolase_5 domain-containing protein	AN-M
MELO3C005763.2	24.327	15.953	3.416	11.736	8.875	158.397	13.984	Glutaredoxin	AN-M
MELO3C005777.2	3.087	4.945	11.472	5.156	4.357	11.758	5.365	serine/threonine-protein kinase WNK8-like isoform X1	AN-M
MELO3C005778.2	4.573	4.017	2.204	9.311	6.373	31.236	5.626	Oligopeptide transporter, putative	AN-M

Gene ID	FPKM							Gene Description	Specific in episode
	FS	GI-M	GM-M	AN-M	GI-H	GM-H	AN-H		
MELO3C005792.2	3.653	4.376	0.741	7.587	3.948	29.178	2.570	alcohol dehydrogenase-like 4	AN-M
MELO3C005834.2	28.668	34.644	6.571	99.536	19.449	639.144	23.761	Scarecrow-like protein 8, putative	AN-M
MELO3C005850.2	1.397	1.223	2.514	11.770	0.813	193.060	0.840	S-type anion channel SLAH2	AN-M
MELO3C005897.2	2.041	0.773	0.763	0.453	0.719	5.937	1.283	AAA-ATPase At2g46620-like	AN-M
MELO3C012448.2	2.355	2.001	1.965	1.259	2.271	12.094	2.713	Aquarius	AN-M
MELO3C012323.2	2.362	2.552	2.586	7.166	2.372	25.335	2.020	3-oxo-5-alpha-steroid 4-dehydrogenase 2-like	AN-M
MELO3C012278.2	16.411	18.809	3.643	25.538	11.139	81.043	14.501	Octicosapeptide/Phox/Bem1p family protein, putative	AN-M
MELO3C012267.2	25.582	30.324	7.725	67.125	20.111	165.771	24.326	Phosphatase 2C family protein	AN-M
MELO3C012145.2	11.280	12.941	8.945	15.535	14.320	48.673	15.605	S-adenosyl-L-methionine-dependent methyltransferases superfamily protein isoform 1	AN-M
MELO3C012071.2	1.901	3.355	3.630	8.457	2.089	33.460	2.509	Alpha-amylase, putative	AN-M
MELO3C012055.2	0.148	0.245	0.231	2.425	0.463	7.344	0.319	NRT1/PTR family protein 2.2	AN-M
MELO3C012021.2	0.946	0.950	0.547	5.943	1.771	30.713	1.131	Serine/threonine-protein kinase TAO3	AN-M
MELO3C012010.2	18.925	20.611	12.281	8.993	8.396	44.744	10.200	Trehalose-6-phosphate synthase	AN-M
MELO3C011949.2	6.751	6.196	6.122	3.038	4.389	14.357	4.484	Octicosapeptide/Phox/Bem1p domain-containing protein kinase	AN-M
MELO3C011905.2	18.949	19.804	14.286	10.651	10.590	41.491	12.849	RING finger protein	AN-M
MELO3C011778.2	18.176	22.616	8.375	26.540	15.585	79.151	18.109	E3 ubiquitin-protein ligase CHFR	AN-M
MELO3C011719.2	4.909	7.223	4.496	12.957	4.091	32.319	6.054	Adenylate kinase	AN-M
MELO3C026572.2	10.986	12.669	2.996	16.732	8.208	39.285	7.230	Proline transporter	AN-M
MELO3C026575.2	0.617	0.832	0.517	2.005	0.751	12.016	0.528	Glycosyltransferase	AN-M
MELO3C025157.2	9.992	14.580	6.657	15.979	11.224	37.533	13.586	peroxisomal nicotinamide adenine dinucleotide carrier isoform X1	AN-M
MELO3C025164.2	628.257	877.297	159.392	1075.118	484.076	2279.204	508.332	Aquaporin	AN-M
MELO3C025189.2	0.454	0.696	0.919	2.136	0.485	9.321	0.480	Alpha/beta hydrolase related protein	AN-M
MELO3C022598.2	4.475	4.879	2.545	6.270	3.208	17.663	4.577	Tobamovirus multiplication 1 homolog	AN-M
MELO3C018331.2	3.134	4.757	6.589	5.221	4.690	15.818	5.434	1-phosphatidylinositol-3-phosphate 5-kinase	AN-M
MELO3C018314.2	0.094	0.080	0.013	0.566	0.058	1.718	NA	subtilisin-like protease SBT4.14	AN-M
MELO3C018312.2	0.058	0.140	0.045	0.564	0.363	1.448	0.245	subtilisin-like protease SBT4.14	AN-M
MELO3C026425.2	4.479	4.789	4.602	5.994	5.727	14.173	5.388	Clathrin assembly family protein	AN-M
MELO3C022882.2	0.279	0.556	0.186	2.138	NA	5.226	0.308	Pectinesterase	AN-M
MELO3C023234.2	0.186	0.335	0.172	0.786	0.613	1.950	0.235	Pectin lyase-like superfamily protein	AN-M

Gene ID	FPKM							Gene Description	Specific in episode
	FS	GI-M	GM-M	AN-M	GI-H	GM-H	AN-H		
MELO3C023270.2	0.626	1.216	0.469	1.620	0.345	23.497	0.655	WAT1-related protein	AN-M
MELO3C023317.2	23.163	23.935	29.495	17.083	25.945	65.351	31.903	phosphatidylinositol/phosphatidylcholine transfer protein SFH8-like	AN-M
MELO3C020958.2	12.007	13.483	5.015	6.447	7.352	165.551	8.456	Shikimate kinase	AN-M
MELO3C020864.2	0.282	0.547	2.611	0.732	0.866	2.097	1.110	homeobox protein 6	AN-M
MELO3C021927.2	0.178	0.457	0.199	0.903	0.364	4.539	NA	CSL zinc finger domain-containing protein	AN-M
MELO3C021821.2	1.858	3.651	0.529	3.614	0.539	7.302	0.695	Cellulose synthase	AN-M
MELO3C024729.2	1.526	2.395	2.823	3.264	1.553	7.196	1.698	NADH dehydrogenase [ubiquinone] iron-sulfur protein 6, mitochondrial	AN-M
MELO3C019234.2	0.444	0.448	0.191	2.049	NA	46.451	1.022	cyclin-U1-1	AN-M
MELO3C019320.2	12.853	13.551	10.691	21.331	8.105	54.848	10.701	Acyl-coenzyme A oxidase	AN-M
MELO3C013625.2	3.591	3.687	2.680	1.665	1.970	10.010	1.901	Protein kinase-like protein	AN-M
MELO3C013676.2	8.163	8.545	5.047	18.404	6.861	115.864	6.093	Very-long-chain (3R)-3-hydroxyacyl-CoA dehydratase	AN-M
MELO3C026841.2	0.215	0.676	1.030	0.878	0.415	2.147	0.400	UDP-glucose 4-epimerase family protein	AN-M
MELO3C026870.2	1.713	3.888	2.214	8.279	1.196	52.274	2.196	Peroxidase	AN-M
MELO3C025784.2	2.244	1.593	0.910	5.884	0.765	26.474	1.612	Zinc finger (C3HC4-type RING finger) family protein	AN-M
MELO3C021413.2	5.804	7.226	18.785	7.997	7.308	16.523	6.806	katanin p60 ATPase-containing subunit A1	AN-M
MELO3C021390.2	95.168	125.966	28.972	220.090	88.939	690.044	85.427	Bi1-like protein	AN-M
MELO3C021362.2	25.524	30.769	27.285	10.083	31.690	98.276	40.593	Beta-amylase	AN-M
MELO3C022302.2	2.361	1.999	7.842	1.008	1.599	6.982	1.922	protein LHY-like isoform X1	AN-M
MELO3C022345.2	6.186	8.636	6.583	24.361	5.511	53.223	4.898	somatic embryogenesis receptor kinase 2-like	AN-M
MELO3C022421.2	4.237	5.996	4.281	8.191	5.074	17.263	4.929	ABC transporter F family-like protein	AN-M
MELO3C022519.2	1.007	1.403	3.773	2.135	1.991	5.037	1.899	Phospholipase D	AN-M
MELO3C020525.2	5.956	5.754	4.933	13.698	6.680	57.846	7.774	farnesol kinase, chloroplastic	AN-M
MELO3C020625.2	9.438	8.578	2.527	27.554	7.020	144.250	6.734	Rhomboid-like protein	AN-M
MELO3C020683.2	25.292	24.576	34.503	38.852	22.799	104.114	22.528	Calcium-transporting ATPase	AN-M
MELO3C020691.2	12.975	20.118	15.299	21.699	14.429	51.234	12.075	Zinc finger, C3HC4 type family protein, putative	AN-M
MELO3C005052.2	0.343	0.628	0.752	2.882	1.639	5.979	0.405	Laccase	AN-M
MELO3C004998.2	1.094	2.998	1.513	7.054	0.627	93.971	0.640	Exostosin family protein	AN-M
MELO3C004957.2	0.436	0.368	0.422	0.796	0.213	2.728	0.229	ARM repeat superfamily protein	AN-M
MELO3C004931.2	0.031	0.009	0.017	0.146	0.022	3.750	0.022	ABC transporter G family member 28	AN-M
MELO3C004889.2	3.074	3.949	1.705	5.043	4.304	12.424	3.301	CASP-like protein	AN-M

Gene ID	FPKM							Gene Description	Specific in episode
	FS	GI-M	GM-M	AN-M	GI-H	GM-H	AN-H		
MELO3C004867.2	16.700	23.299	6.253	28.761	12.154	62.433	11.926	pheophorbide a oxygenase, chloroplastic	AN-M
MELO3C025543.2	2.363	3.275	6.543	6.011	3.495	25.266	3.404	heterogeneous nuclear ribonucleoprotein 1	AN-M
MELO3C025524.2	4.056	5.491	2.789	12.697	5.448	72.439	4.469	ABC transporter I family protein	AN-M
MELO3C026065.2	6.241	7.155	2.738	8.545	5.984	20.977	5.761	Acetyl-CoA acetyltransferase	AN-M
MELO3C021818.2	16.438	14.909	11.619	99.686	12.780	486.510	15.397	Protein LIGHT-DEPENDENT SHORT HYPOCOTYLS 10	AN-M
MELO3C035490.2	0.224	0.365	0.201	0.498	0.256	2.695	0.107	Unknown protein	AN-M
MELO3C002744.2	1.717	2.504	0.710	5.527	4.405	14.342	3.096	9-cis-epoxycarotenoid dioxygenase	AN-M
MELO3C002714.2	3.612	4.622	2.840	6.303	3.475	26.615	3.636	60S ribosomal protein L18a-like protein	AN-M
MELO3C002677.2	12.198	12.827	5.730	16.237	13.374	50.519	13.095	NAD(P)-binding Rossmann-fold superfamily protein	AN-M
MELO3C002605.2	2.234	3.184	4.179	5.826	4.219	18.116	4.208	Inner membrane protein oxaA	AN-M
MELO3C002591.2	3.761	2.170	1.601	11.481	2.628	34.474	2.166	sphingolipid delta(4)-desaturase DES1-like	AN-M
MELO3C002590.2	1.009	2.657	0.846	15.877	1.143	142.681	0.576	CBS domain-containing protein CBSX5	AN-M
MELO3C002555.2	0.547	0.802	1.324	1.602	0.518	5.108	0.563	external alternative NAD(P)H-ubiquinone oxidoreductase B2, mitochondrial	AN-M
MELO3C002484.2	1.725	2.279	2.309	3.542	1.711	15.870	2.229	Pyruvate, phosphate dikinase regulatory protein, putative	AN-M
MELO3C002400.2	0.310	0.644	0.215	2.462	0.268	9.810	0.439	Glycosyltransferase	AN-M
MELO3C035535.2	3.908	3.848	1.893	8.992	5.092	31.090	7.388	4-coumarate-CoA ligase	AN-M
MELO3C002345.2	15.515	15.139	1.636	4.616	3.751	44.296	1.069	Emb|CAB62340.1	AN-M
MELO3C002318.2	44.698	61.387	16.849	21.953	31.102	139.029	34.476	BTB/POZ and TAZ domain-containing protein 1-like	AN-M
MELO3C002269.2	14.953	22.992	11.605	25.713	16.780	98.454	22.340	Boron transporter	AN-M
MELO3C002247.2	1.502	0.955	1.831	20.099	3.335	66.806	4.564	Transcription factor	AN-M
MELO3C002246.2	9.307	12.833	6.790	22.234	7.905	56.892	7.666	RING-H2 finger ATL48-like protein	AN-M
MELO3C002154.2	2.777	4.710	2.150	10.748	1.547	82.499	1.533	Monoglyceride lipase	AN-M
MELO3C002149.2	23.560	30.259	10.265	77.298	23.199	157.917	19.883	Dihydropyrimidine dehydrogenase	AN-M
MELO3C002147.2	10.787	17.323	6.391	21.037	3.507	94.556	6.165	At1g67920	AN-M
MELO3C002086.2	5.469	8.371	29.168	9.424	4.785	21.156	6.761	NADH dehydrogenase [ubiquinone] 1 beta subcomplex subunit 10-B	AN-M
MELO3C002032.2	12.922	14.051	5.192	17.971	13.239	82.166	10.696	Presenilin	AN-M
MELO3C001998.2	89.309	82.891	23.556	144.890	60.903	367.319	56.820	Ribulose-phosphate 3-epimerase-like protein	AN-M
MELO3C027064.2	0.865	0.629	2.343	0.717	1.675	1.944	1.145	Pentatricopeptide repeat-containing protein	AN-M
MELO3C027095.2	0.695	0.921	0.540	1.071	1.348	2.205	1.447	metal-nicotianamine transporter YSL1-like	AN-M
MELO3C027104.2	0.988	1.681	1.843	2.411	NA	5.148	1.457	Protein LIGHT-DEPENDENT SHORT HYPOCOTYLS 10	AN-M

Gene ID	FPKM							Gene Description	Specific in episode
	FS	GI-M	GM-M	AN-M	GI-H	GM-H	AN-H		
MELO3C027188.2	0.471	0.465	1.001	0.224	0.183	3.053	0.381	Rhamnogalacturonate lyase	AN-M
MELO3C027221.2	8.145	6.039	6.410	11.242	7.961	23.813	9.350	RING-type E3 ubiquitin transferase	AN-M
MELO3C027266.2	39.503	36.224	18.896	32.831	24.361	86.936	16.320	Non-specific serine/threonine protein kinase	AN-M
MELO3C027346.2	31.906	42.292	10.408	39.385	7.031	118.515	3.289	Indole-3-acetic acid-amido synthetase GH3.3	AN-M
MELO3C027887.2	2.761	2.155	0.561	2.436	1.126	8.442	1.047	F-box protein At1g67340	AN-M
MELO3C027373.2	1.746	2.555	8.569	2.577	1.789	5.968	0.982	ADP-ribosylation factor GTPase-activating protein AGD12-like	AN-M
MELO3C000032.2	5.411	3.977	8.186	4.827	3.964	21.708	6.011	U3 small nucleolar ribonucleoprotein IMP3	AN-M
MELO3C000393.2	1.127	2.159	NA	2.487	0.226	16.016	1.322	ferric reduction oxidase 7, chloroplastic	AN-M
MELO3C000228.2	1.363	0.709	3.763	0.476	1.659	5.786	0.296	UBN2_3 domain-containing protein	AN-M
MELO3C027678.2	8.583	10.952	6.502	12.242	8.575	56.868	11.410	Aspartic proteinase	AN-M
MELO3C000372.2	9.480	8.912	10.137	6.845	5.476	22.588	6.591	Phospholipid-transporting ATPase	AN-M
MELO3C001234.2	67.785	67.089	23.238	43.752	43.854	310.275	47.828	Sulfate transporter 3.1	AN-M
MELO3C001494.2	28.415	28.697	13.205	34.106	24.782	90.593	33.319	Pectinesterase	AN-M
MELO3C001556.2	23.716	19.712	13.977	19.500	25.750	65.205	27.444	U3 small nucleolar ribonucleoprotein protein IMP3	AN-M
MELO3C001698.2	2.360	3.805	3.194	3.902	2.720	12.258	4.579	Bidirectional sugar transporter SWEET	AN-M
MELO3C018415.2	1.935	3.069	1.402	2.786	2.675	8.228	1.103	Myosin heavy chain kinase B	AN-M
MELO3C018430.2	9.340	9.096	5.292	13.848	3.619	31.183	1.922	Two-component response regulator	AN-M
MELO3C018439.2	4.307	7.670	3.365	9.584	9.196	26.160	9.431	Calmodulin-binding protein	AN-M
MELO3C018442.2	2.852	3.399	1.772	4.437	1.058	17.353	1.000	Cyclin-dependent kinase inhibitor	AN-M
MELO3C018478.2	19.460	13.801	15.353	20.231	12.999	40.546	17.305	kynurenine formamidase	AN-M
MELO3C018482.2	2.916	4.439	2.220	2.845	2.844	16.463	2.387	ADP-ribosylation factor, arf, putative	AN-M
MELO3C018529.2	8.168	8.895	3.089	10.976	7.735	22.177	8.528	Magnesium transporter MRS2-3	AN-M
MELO3C018532.2	16.610	18.774	2.549	25.197	6.568	86.531	7.812	tubulin alpha chain-like	AN-M
MELO3C018569.2	4.820	4.173	2.702	3.020	3.030	17.994	3.764	peptide methionine sulfoxide reductase B5-like	AN-M
MELO3C018570.2	7.167	6.484	3.236	6.680	5.228	21.788	6.099	K(+) efflux antiporter 3, chloroplastic	AN-M
MELO3C018571.2	4.421	4.682	1.641	5.280	3.063	12.574	4.067	Major facilitator superfamily	AN-M
MELO3C018588.2	1.115	0.514	0.997	1.333	0.491	5.449	0.163	E3 ubiquitin-protein ligase RING1-like	AN-M
MELO3C018679.2	2.484	2.727	4.063	2.677	2.557	9.815	2.822	mediator of RNA polymerase II transcription subunit 15a	AN-M
MELO3C018683.2	39.999	45.868	12.580	47.324	35.594	180.178	47.634	Actin	AN-M
MELO3C018724.2	49.555	47.537	36.958	56.394	53.353	159.004	65.468	Phosphoenolpyruvate carboxylase	AN-M
MELO3C018736.2	2.248	2.686	4.289	2.110	2.572	8.792	2.765	Formin-like protein (DUF1421)	AN-M

Gene ID	FPKM							Gene Description	Specific in episode
	FS	GI-M	GM-M	AN-M	GI-H	GM-H	AN-H		
MELO3C018740.2	0.811	0.541	0.568	0.822	0.611	3.692	0.317	E3 ubiquitin-protein ligase RMA1H1	AN-M
MELO3C018778.2	0.941	1.050	1.515	0.718	1.393	3.802	0.411	Protein TIC 20-IV, chloroplastic	AN-M
MELO3C018807.2	57.289	87.400	60.671	72.790	58.898	238.691	40.702	protein translation factor SUI1 homolog	AN-M
MELO3C018834.2	13.726	11.458	6.543	13.612	11.961	43.746	11.496	Ubiquitin fusion degradation 1	AN-M
MELO3C023710.2	3.641	2.856	1.922	2.484	2.031	9.497	3.801	transcription factor VIP1	AN-M
MELO3C023684.2	2.851	4.211	4.828	2.383	1.885	11.670	2.327	Scarecrow-like protein	AN-M
MELO3C023682.2	6.748	4.390	2.837	6.536	4.546	31.826	5.753	Photosystem II reaction center PsbP family protein	AN-M
MELO3C024067.2	3.000	3.036	3.267	2.806	2.571	6.705	3.424	protein GIGANTEA-like	AN-M
MELO3C024080.2	1.664	2.172	0.766	1.863	0.757	11.566	0.422	ABC transporter family protein	AN-M
MELO3C024086.2	1.228	1.240	0.369	1.859	0.366	4.482	0.383	70 kDa heat shock protein	AN-M
MELO3C024122.2	4.918	4.681	3.501	5.184	4.857	10.565	6.374	Calcium-dependent protein kinase, putative	AN-M
MELO3C024969.2	2.748	2.448	5.265	2.037	2.501	5.695	2.246	WW domain-containing protein	AN-M
MELO3C024912.2	0.573	0.662	1.725	0.761	0.694	1.961	0.566	Receptor-like kinase	AN-M
MELO3C024900.2	6.300	9.168	3.503	9.604	3.744	33.428	3.863	Serine acetyltransferase	AN-M
MELO3C013123.2	11.407	15.103	15.546	12.416	10.125	34.018	7.448	Syntaxin-51	AN-M
MELO3C013125.2	0.428	0.865	1.221	0.393	0.484	1.742	0.446	Lipid-binding serum glycoprotein family protein, putative	AN-M
MELO3C013132.2	0.777	0.724	0.228	0.814	0.477	3.359	0.223	Extracellular ligand-gated ion channel protein (DUF3537)	AN-M
MELO3C013163.2	5.919	8.798	24.566	8.732	5.455	20.893	3.604	Protein translation factor sui1-like protein	AN-M
MELO3C013167.2	0.870	1.870	5.197	1.295	1.524	4.163	0.874	Ubiquitin thioesterase OTU1-like protein	AN-M
MELO3C013262.2	2.852	2.536	2.242	3.403	2.035	7.153	2.151	SEC1 family transport protein SLY1	AN-M
MELO3C013309.2	11.653	14.102	5.620	16.700	10.198	43.723	14.174	Coiled-coil protein	AN-M
MELO3C013310.2	0.341	0.586	1.699	0.795	0.484	12.633	0.530	O-methyltransferase, putative	AN-M
MELO3C013321.2	6.658	6.786	3.884	5.652	6.130	16.449	9.022	F-box/LRR protein	AN-M
MELO3C013330.2	2.006	1.805	1.559	2.759	1.006	19.167	1.554	Zinc finger, DNL-type	AN-M
MELO3C013351.2	1.513	1.402	1.216	1.506	1.241	3.238	1.226	Urease accessory protein ureH, putative	AN-M
MELO3C013353.2	4.557	6.116	13.885	4.739	8.764	23.460	9.062	heavy metal-associated isoprenylated plant protein 26-like	AN-M
MELO3C013374.2	8.776	10.490	3.926	13.159	9.042	70.860	7.245	Magnesium transporter NIPA	AN-M
MELO3C029039.2	0.640	0.808	0.788	0.591	1.003	1.925	1.166	COBRA-like protein 2	AN-M
MELO3C013389.2	0.323	0.659	1.318	0.441	0.751	2.038	0.401	U-box domain-containing protein 35-like	AN-M
MELO3C013431.2	2.463	1.684	1.056	2.724	2.094	15.482	2.986	TBC1 domain family member 2A	AN-M
MELO3C012550.2	7.783	5.213	1.736	5.457	5.837	17.387	7.027	polyol transporter 5-like	AN-M

Gene ID	FPKM							Gene Description	Specific in episode
	FS	GI-M	GM-M	AN-M	GI-H	GM-H	AN-H		
MELO3C012551.2	0.564	0.993	0.200	0.394	NA	6.327	0.404	polyol transporter 5-like	AN-M
MELO3C012646.2	5.094	6.271	7.987	5.646	5.542	21.988	6.114	Acyl-CoA N-acyltransferase with RING/FYVE/PHD-type zinc finger protein, putative	AN-M
MELO3C012766.2	11.404	13.279	16.725	9.514	9.518	32.622	10.228	AT3g24740/K7P8_3	AN-M
MELO3C021013.2	0.913	0.748	0.748	0.636	0.471	1.899	0.937	E3 ubiquitin-protein ligase PRT1 isoform X1	AN-M
MELO3C015698.2	0.653	1.010	1.920	0.957	0.239	2.307	0.742	Pentatricopeptide repeat-containing protein	AN-M
MELO3C015761.2	3.359	3.363	4.730	3.867	4.258	8.738	3.326	protein OSB2, chloroplastic-like	AN-M
MELO3C015774.2	0.497	0.568	1.542	0.814	0.329	4.767	0.345	Hexosyltransferase	AN-M
MELO3C015794.2	0.306	0.350	1.338	0.571	0.627	1.692	0.696	homocysteine S-methyltransferase 2	AN-M
MELO3C015795.2	0.604	1.151	1.481	0.537	0.346	14.961	0.315	Serine/Threonine kinase family protein	AN-M
MELO3C015804.2	0.423	0.633	1.033	0.290	NA	2.082	0.423	heat stress transcription factor A-6b-like isoform X1	AN-M
MELO3C015830.2	3.709	3.480	7.657	3.602	2.075	9.578	2.957	Time for coffee	AN-M
MELO3C015851.2	5.458	4.942	4.349	5.811	6.235	16.462	6.008	R3H domain-containing protein 4	AN-M
MELO3C015879.2	9.471	10.253	6.809	8.397	7.150	31.633	11.257	WD-repeat protein, putative	AN-M
MELO3C015888.2	2.176	2.277	4.444	1.354	NA	9.411	0.529	Protein TIC 214	AN-M
MELO3C015889.2	1.752	2.564	6.781	2.240	5.408	7.201	4.587	RNA-binding protein BRN1	AN-M
MELO3C015890.2	1.283	0.870	1.486	1.782	1.364	7.153	1.462	Mitochondrial substrate carrier family protein	AN-M
MELO3C015913.2	5.729	6.174	4.249	5.508	4.903	36.648	6.768	Pentatricopeptide repeat-containing protein	AN-M
MELO3C015922.2	3.977	6.174	15.980	5.482	5.328	12.575	4.364	cytochrome c oxidase subunit 5C-like	AN-M
MELO3C015958.2	7.632	5.577	5.590	5.825	6.394	15.941	6.463	Mitochondrial carrier protein	AN-M
MELO3C028931.2	0.461	1.206	0.545	0.939	1.289	3.494	1.570	TOBAMOVIRUS MULTIPLICATION 1 family protein	AN-M
MELO3C016015.2	9.835	9.755	9.655	8.379	9.811	21.488	11.624	AAA-type ATPase family protein	AN-M
MELO3C016017.2	3.620	3.863	0.619	3.598	1.440	13.009	1.629	translocator protein homolog	AN-M
MELO3C016035.2	31.130	35.405	12.182	37.319	23.546	106.774	24.624	WD-repeat protein, putative	AN-M
MELO3C016048.2	2.701	2.922	4.583	2.985	3.634	8.486	4.019	spermatogenesis-associated protein 20 isoform X2	AN-M
MELO3C016051.2	0.419	0.497	1.549	0.679	0.948	3.063	1.520	BTB/POZ domain-containing protein	AN-M
MELO3C023609.2	0.741	0.996	0.343	1.454	0.702	4.065	1.162	TolB protein-like protein	AN-M
MELO3C023587.2	2.117	3.451	0.641	0.797	1.092	7.143	0.191	Protein BPS1 chloroplastic	AN-M
MELO3C023573.2	19.961	12.732	10.868	17.287	19.304	80.972	8.125	At3g57450	AN-M
MELO3C023555.2	8.406	7.312	6.739	9.010	7.703	38.531	8.839	Carotenoid cleavage dioxygenase	AN-M
MELO3C023541.2	3.773	2.874	5.090	3.403	4.639	10.245	4.345	E3 ubiquitin-protein ligase makorin	AN-M

Gene ID	FPKM							Gene Description	Specific in episode
	FS	GI-M	GM-M	AN-M	GI-H	GM-H	AN-H		
MELO3C023540.2	1.477	2.214	1.955	4.821	2.494	9.854	1.055	Unknown protein	AN-M
MELO3C023520.2	8.434	9.110	2.547	6.101	5.936	23.427	7.560	uroporphyrinogen-III C-methyltransferase	AN-M
MELO3C023515.2	5.972	3.398	6.594	4.391	3.912	17.274	4.303	DNA-binding bromodomain-containing family protein	AN-M
MELO3C023501.2	1.118	1.696	1.877	1.295	1.000	9.784	1.313	Major facilitator superfamily domain-containing protein 12	AN-M
MELO3C023491.2	0.552	0.440	0.704	0.607	0.329	2.446	0.344	Ribulose bisphosphate carboxylase/oxygenase activase	AN-M
MELO3C023486.2	0.693	1.055	1.747	1.048	0.809	4.877	0.869	Snf1-related kinase interactor 1, putative	AN-M
MELO3C023474.2	94.683	94.375	16.610	131.600	39.002	593.217	38.850	Hydroxycinnamoyl-CoA shikimate/quinate hydroxycinnamoyltransferase	AN-M
MELO3C023442.2	14.152	16.670	6.872	20.046	9.990	71.975	13.016	glycerol kinase	AN-M
MELO3C023430.2	2.523	1.887	2.803	1.956	3.625	7.003	2.620	Hus1-like protein	AN-M
MELO3C023402.2	1.160	1.151	3.894	1.263	1.763	3.518	1.624	Biotin carboxyl carrier protein of acetyl-CoA carboxylase	AN-M
MELO3C023364.2	4.287	3.425	5.949	3.882	3.017	8.889	3.390	Kinase, putative	AN-M
MELO3C024245.2	2.032	2.404	4.920	2.652	3.138	5.601	2.427	Phox-associated domain,Phox-like,Sorting nexin isoform 1	AN-M
MELO3C024246.2	1.457	2.102	3.723	1.786	0.638	4.840	1.809	ras-related protein Rab7	AN-M
MELO3C024291.2	15.572	12.548	10.023	12.443	10.701	39.193	13.946	Calcium-transporting ATPase	AN-M
MELO3C024298.2	16.836	11.893	5.382	21.155	16.406	95.648	17.001	exocyst complex component EXO84B	AN-M
MELO3C024325.2	12.349	9.311	4.313	9.930	14.479	28.674	10.267	Dormancy/auxin associated family protein, putative	AN-M
MELO3C024351.2	24.965	40.365	7.780	39.713	13.602	99.506	9.269	RELA/SPOT	AN-M
MELO3C024378.2	9.221	10.772	13.570	11.188	11.275	24.100	13.512	Phospholipid-transporting ATPase	AN-M
MELO3C015236.2	0.630	0.411	1.227	0.635	0.535	1.941	0.929	pentatricopeptide repeat-containing protein At3g24000, mitochondrial-like	AN-M
MELO3C015237.2	0.712	0.982	4.027	0.998	0.608	2.658	0.600	titin isoform X2	AN-M
MELO3C015252.2	1.352	1.389	2.441	2.215	1.052	18.776	1.643	IGR motif protein	AN-M
MELO3C015272.2	0.283	0.176	0.391	0.523	0.126	3.365	0.314	kinesin-4 isoform X1	AN-M
MELO3C015299.2	23.001	20.120	12.689	22.318	17.582	64.750	19.636	serine/arginine-rich splicing factor SR30	AN-M
MELO3C015310.2	20.976	27.444	9.754	30.864	14.631	85.446	12.147	Rubber elongation factor protein (REF)	AN-M
MELO3C015400.2	13.612	13.781	12.786	19.754	11.459	43.341	14.029	mediator-associated protein 1	AN-M
MELO3C015402.2	18.515	31.330	10.458	21.633	7.080	85.884	7.597	guanine deaminase	AN-M
MELO3C015459.2	3.136	3.872	1.651	8.021	3.041	16.962	2.468	Cytidine deaminase	AN-M
MELO3C015478.2	2.100	0.911	3.128	3.655	0.809	28.223	0.281	Cytokinin oxidase/dehydrogenase	AN-M
MELO3C015501.2	3.033	3.650	10.992	3.121	3.871	7.834	3.883	myosin-17-like	AN-M
MELO3C015510.2	0.675	1.148	3.797	0.686	0.947	2.487	0.629	protein ELF4-LIKE 4	AN-M

Gene ID	FPKM							Gene Description	Specific in episode
	FS	GI-M	GM-M	AN-M	GI-H	GM-H	AN-H		
MELO3C015518.2	2.874	3.313	1.993	2.943	3.675	9.938	2.424	Acyl-CoA N-acyltransferase (NAT) superfamily protein	AN-M
MELO3C015525.2	43.087	42.671	24.489	40.687	45.437	174.482	34.441	RING-box protein	AN-M
MELO3C015532.2	1.714	1.986	3.727	2.125	3.106	4.485	2.572	Inter-alpha-trypsin inhibitor heavy chain-like protein	AN-M
MELO3C015535.2	2.012	2.137	2.032	2.868	1.847	7.704	1.580	ubiquinone biosynthesis protein COQ9-B, mitochondrial	AN-M
MELO3C015554.2	5.999	6.910	3.968	4.974	6.403	22.543	6.485	Zinc finger, FYVE-type	AN-M
MELO3C015603.2	2.818	2.173	2.563	2.446	1.767	5.883	3.691	E3 ubiquitin-protein ligase RING1-like	AN-M
MELO3C015199.2	3.476	4.790	4.705	3.363	2.008	13.798	2.115	Xanthine dehydrogenase, putative	AN-M
MELO3C015193.2	1.960	2.438	4.666	2.262	1.295	7.816	1.249	Chaperonin-like RbcX protein 2, chloroplastic	AN-M
MELO3C015180.2	7.597	10.342	6.282	8.706	5.061	21.920	6.260	NAD(H) kinase 1	AN-M
MELO3C015169.2	4.356	2.953	2.900	2.586	2.945	26.248	2.399	Phosphotransferase	AN-M
MELO3C015151.2	9.868	13.756	2.993	12.470	8.731	92.848	13.956	Alanine:glyoxylate aminotransferase	AN-M
MELO3C015071.2	2.944	2.792	0.413	3.859	2.450	8.620	2.300	aluminum-activated malate transporter 2-like	AN-M
MELO3C015040.2	8.330	7.508	3.083	9.554	6.530	24.200	7.823	Serine/threonine-protein phosphatase	AN-M
MELO3C010008.2	6.410	5.627	1.682	6.199	3.868	27.546	3.836	AT1G65230-like protein	AN-M
MELO3C029597.2	0.115	0.131	0.078	0.328	0.409	1.840	0.215	zinc finger BED domain-containing protein RICESLEEPER 2-like	AN-M
MELO3C010064.2	15.333	14.212	6.541	18.010	10.046	47.687	12.453	Zinc finger CCCH domain protein	AN-M
MELO3C010113.2	4.091	3.252	1.950	4.158	4.010	10.321	3.217	Receptor kinase-like protein	AN-M
MELO3C010180.2	138.070	149.992	46.052	128.613	64.198	538.811	34.997	tetraspanin-8-like	AN-M
MELO3C010183.2	2.191	4.329	0.244	3.701	0.851	33.180	1.362	glycine-rich cell wall structural protein 2-like	AN-M
MELO3C010186.2	2.692	1.973	1.327	2.326	1.994	13.916	0.898	UDP-glucuronate 4-epimerase 1-like	AN-M
MELO3C010202.2	0.750	1.294	4.618	1.086	1.628	3.104	1.285	Pentatricopeptide repeat-containing protein	AN-M
MELO3C010211.2	3.079	5.572	4.985	5.369	1.928	13.049	1.953	UPF0057 membrane protein At4g30660	AN-M
MELO3C010275.2	5.009	7.436	2.194	6.134	2.527	25.610	3.604	Serine-rich protein-like protein	AN-M
MELO3C010334.2	1.514	2.193	2.628	2.079	1.777	9.315	2.481	Non-specific serine/threonine protein kinase	AN-M
MELO3C010353.2	15.014	19.637	11.656	12.257	11.229	50.943	13.007	protein NRT1/ PTR FAMILY 6.2-like	AN-M
MELO3C010356.2	12.608	13.261	9.950	18.397	8.995	38.459	11.472	Chaperone protein dnaJ, putative	AN-M
MELO3C010357.2	6.391	8.250	3.072	8.610	6.867	18.405	7.102	Chaperone protein dnaJ, putative	AN-M
MELO3C026630.2	4.430	3.564	2.589	3.292	2.834	11.440	1.608	AT3g15630/MSJ11_3	AN-M
MELO3C026627.2	6.371	8.269	15.266	8.298	8.406	21.627	9.029	26S protease regulatory subunit, putative	AN-M
MELO3C025286.2	6.057	4.150	6.199	5.083	5.594	19.753	4.853	Soul heme-binding family protein	AN-M
MELO3C025295.2	0.655	1.089	0.705	1.170	0.244	2.863	0.336	Two-component response regulator	AN-M

Gene ID	FPKM							Gene Description	Specific in episode
	FS	GI-M	GM-M	AN-M	GI-H	GM-H	AN-H		
MELO3C025306.2	1.676	1.817	1.372	1.568	1.481	3.644	1.648	tudor domain-containing protein 3 isoform X1	AN-M
MELO3C025307.2	1.197	1.740	2.107	2.004	1.135	7.153	1.264	Transmembrane 19	AN-M
MELO3C025310.2	21.402	17.427	8.006	21.713	18.713	57.490	19.850	DnaJ homolog subfamily C GRV2	AN-M
MELO3C024635.2	33.360	28.707	9.568	34.877	23.204	83.121	30.298	protein phosphatase 2C and cyclic nucleotide-binding/kinase domain-containing protein isoform X1	AN-M
MELO3C017514.2	10.293	7.984	7.372	11.647	6.714	92.608	5.816	chaperone protein ClpB	AN-M
MELO3C017500.2	49.310	43.207	15.863	33.880	22.674	302.468	26.368	zinc finger protein CONSTANS-LIKE 5-like	AN-M
MELO3C017499.2	2.543	3.252	2.200	1.977	1.333	20.182	1.378	1-deoxy-D-xylulose-5-phosphate synthase	AN-M
MELO3C017489.2	10.433	12.241	5.376	9.265	7.765	26.364	9.784	F-box family protein, putative	AN-M
MELO3C017481.2	10.736	12.903	9.691	13.175	4.355	255.804	1.690	Xyloglucan endotransglucosylase/hydrolase	AN-M
MELO3C017480.2	9.850	11.993	9.811	7.375	8.215	151.722	11.955	Xyloglucan endotransglucosylase/hydrolase	AN-M
MELO3C017467.2	4.213	4.069	5.283	3.433	1.588	8.888	2.545	Syntaxin/T-SNARE family protein	AN-M
MELO3C017455.2	0.553	0.849	4.743	0.663	0.558	5.222	0.606	VIN3-like protein 2	AN-M
MELO3C017447.2	2.128	2.842	5.522	2.190	3.767	5.785	3.771	LOW QUALITY PROTEIN: villin-4	AN-M
MELO3C017389.2	4.160	6.771	9.719	6.580	5.716	16.729	5.534	ethanolamine-phosphate cytidylyltransferase-like	AN-M
MELO3C017385.2	0.193	0.202	0.177	0.319	0.115	1.889	NA	pollen receptor-like kinase 1	AN-M
MELO3C017376.2	2.155	1.630	1.427	3.795	NA	23.529	NA	Gb|AAF02136.1	AN-M
MELO3C017375.2	4.937	4.866	3.942	4.170	4.062	15.926	4.042	Outer arm dynein light chain 1 protein	AN-M
MELO3C017320.2	3.279	3.652	8.941	2.452	3.414	7.507	3.761	Zinc finger protein, putative	AN-M
MELO3C017271.2	4.506	5.969	4.756	6.158	4.480	12.604	4.837	Acetyl-coenzyme A synthetase	AN-M
MELO3C017251.2	3.957	1.647	1.304	3.493	1.153	11.210	1.909	Dirigent protein	AN-M
MELO3C017212.2	2.511	3.123	3.039	3.850	2.360	8.413	2.531	Leucine-rich repeat (LRR) family protein	AN-M
MELO3C017207.2	5.660	8.981	4.547	8.189	7.134	20.626	4.665	LYR motif-containing protein 4	AN-M
MELO3C017206.2	4.526	5.456	6.236	5.140	9.355	19.193	11.334	heparanase-like protein 1	AN-M
MELO3C017192.2	3.456	3.159	4.567	3.164	3.661	7.038	3.794	Kinase, putative	AN-M
MELO3C017183.2	0.907	0.969	3.167	0.994	1.756	3.767	1.700	YGL010w-like protein	AN-M
MELO3C017178.2	3.082	2.198	2.262	2.133	1.269	9.169	1.214	cyclic dof factor 3	AN-M
MELO3C017155.2	2.426	3.850	1.030	2.530	1.979	8.169	1.998	Hypersensitive-induced response protein 1	AN-M
MELO3C017147.2	0.243	0.397	0.829	0.430	0.130	1.266	0.307	Zinc finger protein, putative	AN-M
MELO3C017145.2	2.340	2.516	1.913	3.029	2.264	6.528	2.744	protein NUCLEAR FUSION DEFECTIVE 4-like	AN-M
MELO3C017131.2	13.279	11.776	4.009	12.692	7.516	36.534	9.039	General negative regulator of transcription subunit 4	AN-M

Gene ID	FPKM							Gene Description	Specific in episode
	FS	GI-M	GM-M	AN-M	GI-H	GM-H	AN-H		
MELO3C017116.2	0.365	0.768	6.414	0.236	0.985	3.109	0.717	Kinesin-like protein	AN-M
MELO3C017110.2	22.882	22.636	16.219	27.027	17.928	59.604	23.079	BAH domain-containing protein, putative	AN-M
MELO3C026263.2	11.906	13.234	10.242	10.841	9.419	29.014	10.144	DEAD-box ATP-dependent RNA helicase-like protein	AN-M
MELO3C026252.2	4.375	4.941	1.249	6.154	1.995	28.497	1.077	Octicosapeptide/Phox/Bem1p domain-containing protein kinase	AN-M
MELO3C026247.2	4.433	4.997	11.982	3.676	8.449	13.980	7.514	Raffinose synthase	AN-M
MELO3C026245.2	4.371	4.650	1.153	4.320	1.747	9.986	2.624	Protein-L-isoaspartate O-methyltransferase	AN-M
MELO3C026223.2	1.428	3.225	1.524	2.675	1.712	10.762	1.271	Mitochondrial pyruvate carrier	AN-M
MELO3C026200.2	2.423	3.022	5.323	3.446	2.272	7.260	2.615	CBS domain-containing protein	AN-M
MELO3C026194.2	4.478	3.903	7.179	5.381	4.478	37.011	3.731	heavy metal-associated isoprenylated plant protein 3	AN-M
MELO3C026186.2	3.006	4.304	5.194	2.575	2.346	11.818	1.875	membrane-anchored ubiquitin-fold protein 3	AN-M
MELO3C026177.2	15.551	11.362	7.810	16.320	11.169	127.649	14.607	Ubiquitin-conjugating enzyme E2, putative	AN-M
MELO3C026162.2	10.744	13.733	9.230	13.592	8.201	30.913	12.524	ATP-dependent clp protease	AN-M
MELO3C008096.2	0.451	0.718	3.391	0.475	0.545	3.305	0.391	Galactose-binding domain-like protein	AN-M
MELO3C008097.2	1.865	1.849	2.808	1.665	2.252	9.228	2.432	Polyprotein	AN-M
MELO3C008134.2	0.431	0.391	1.924	0.366	0.478	2.583	0.275	Glutaredoxin domain-containing protein/DEP domain-containing protein/DUF547 domain-containing protein	AN-M
MELO3C008139.2	20.526	25.999	3.240	19.242	9.510	119.511	9.504	Metal transporter protein	AN-M
MELO3C008140.2	1.853	2.597	0.978	0.996	1.128	12.466	0.813	Transmembrane protein, putative	AN-M
MELO3C008160.2	0.476	0.801	0.646	0.514	0.534	1.661	0.419	Lactoylglutathione lyase / glyoxalase I family protein	AN-M
MELO3C008163.2	4.742	4.488	1.288	5.613	2.881	21.687	3.091	Lipid phosphate phosphatase 2	AN-M
MELO3C008180.2	16.680	17.327	4.959	19.052	15.316	50.855	16.219	Protein nuclear fusion defective 4	AN-M
MELO3C008197.2	10.611	11.016	6.264	13.388	10.137	29.634	12.912	Chloride channel protein	AN-M
MELO3C008202.2	1.226	2.250	5.161	1.005	1.547	5.052	1.469	Ribulose-phosphate 3-epimerase	AN-M
MELO3C008224.2	4.474	2.068	1.526	2.824	2.744	11.000	2.065	Aspartic proteinase nepenthesin-1	AN-M
MELO3C008225.2	0.856	0.468	0.195	0.811	0.284	4.532	0.380	glycosyltransferase family 92 protein RCOM_0530710-like	AN-M
MELO3C008260.2	1.504	2.289	2.611	1.109	2.105	5.410	1.784	At5g05930	AN-M
MELO3C008275.2	27.471	25.359	8.474	26.618	23.260	67.455	24.025	Translation initiation factor 2 subunit gamma	AN-M
MELO3C008287.2	0.199	0.380	0.426	0.724	1.049	19.330	1.270	Auxin transporter-like protein 2	AN-M
MELO3C008391.2	1.437	1.843	2.290	1.746	1.549	4.909	1.913	Regulator of chromosome condensation (RCC1) family with FYVE zinc finger domain-containing protein	AN-M
MELO3C008433.2	2.973	1.828	1.734	4.339	1.537	30.853	2.072	Pentatricopeptide repeat-containing protein	AN-M

Gene ID	FPKM							Gene Description	Specific in episode
	FS	GI-M	GM-M	AN-M	GI-H	GM-H	AN-H		
MELO3C008478.2	1.212	2.651	1.718	1.196	0.227	11.149	0.545	Protein phosphatase 2c, putative	AN-M
MELO3C010654.2	1.694	2.089	3.078	1.209	1.585	4.666	1.532	NAD kinase 2, chloroplastic-like protein	AN-M
MELO3C010605.2	1.382	1.393	1.500	1.736	0.488	4.424	1.146	At1g21820	AN-M
MELO3C010580.2	3.030	3.279	2.598	2.926	2.717	7.557	2.710	Pentatricopeptide repeat-containing protein	AN-M
MELO3C011640.2	14.374	13.994	9.771	14.918	9.849	40.402	14.738	Zinc finger, C6HC-type	AN-M
MELO3C011632.2	2.042	2.414	2.669	2.353	2.004	5.389	2.407	Aspartic proteinase-like protein 2	AN-M
MELO3C026131.2	2.511	2.143	1.263	2.956	1.897	5.958	2.406	Signal peptide peptidase-like protein	AN-M
MELO3C030042.2	4.066	4.478	2.703	4.850	3.775	10.832	2.641	Transmembrane protein, putative	AN-M
MELO3C019941.2	3.131	3.410	7.355	3.360	5.233	10.253	4.586	T-complex protein 11	AN-M
MELO3C019935.2	27.008	31.583	22.318	32.672	41.606	74.606	43.063	LOW QUALITY PROTEIN: type 1 phosphatases regulator ypi1	AN-M
MELO3C019874.2	1.573	1.862	1.843	2.744	1.649	6.588	2.167	Chloroplast outer envelope 24 kD protein	AN-M
MELO3C019871.2	3.168	2.421	1.288	3.938	1.630	8.719	2.760	Phytol kinase	AN-M
MELO3C019819.2	15.093	20.440	15.087	13.576	17.490	44.827	17.940	ras-related protein RABF1	AN-M
MELO3C019796.2	0.248	0.193	0.664	0.681	0.488	1.827	1.245	Mannan endo-1,4-beta-mannosidase	AN-M
MELO3C019782.2	7.912	10.991	5.999	5.587	7.310	23.703	6.396	Hypersensitive-induced response protein 1	AN-M
MELO3C019780.2	1.513	2.693	1.892	1.608	1.360	5.700	1.539	protein kinase 2B, chloroplastic-like	AN-M
MELO3C026500.2	32.090	43.890	9.269	11.666	13.420	105.975	3.663	Actin cross-linking protein	AN-M
MELO3C026501.2	1.848	1.295	1.701	2.521	0.861	14.919	0.980	exocyst complex component EXO84A	AN-M
MELO3C026530.2	0.485	0.737	4.465	0.918	0.570	9.395	0.368	Pentatricopeptide repeat-containing protein	AN-M
MELO3C026531.2	3.760	4.070	1.585	4.269	2.138	11.138	2.281	Receptor Serine/Threonine kinase	AN-M
MELO3C011438.2	13.513	12.314	4.978	11.992	8.720	28.588	13.159	Ankyrin repeat protein SKIP35	AN-M
MELO3C011371.2	36.811	47.218	49.611	45.136	40.954	114.370	38.387	eukaryotic translation initiation factor 5-like	AN-M
MELO3C011345.2	29.645	23.721	36.976	25.353	30.052	75.769	29.781	Skp1, putative	AN-M
MELO3C011343.2	3.492	3.774	6.520	5.430	6.545	12.609	4.539	GDSL esterase/lipase EXL3	AN-M
MELO3C011211.2	6.438	5.013	5.077	7.638	7.345	16.580	5.906	syntaxin-32	AN-M
MELO3C011201.2	13.924	16.866	5.909	17.484	10.299	40.146	12.369	Plastid lipid-associated protein	AN-M
MELO3C011189.2	1.003	1.515	3.715	1.535	0.936	5.507	0.792	E3 ubiquitin-protein ligase arkadia	AN-M
MELO3C011178.2	1.687	1.757	1.506	2.382	0.957	7.422	0.711	U-box domain-containing protein 51	AN-M
MELO3C011154.2	15.787	11.204	6.402	20.739	13.547	43.546	12.981	target of Myb protein 1	AN-M
MELO3C011153.2	14.062	14.660	8.408	16.405	10.650	36.410	12.406	Ubiquitin-conjugating enzyme, E2	AN-M
MELO3C011119.2	7.277	7.858	4.521	10.307	6.828	20.785	4.175	Inositol-tetrakisphosphate 1-kinase	AN-M

Gene ID	FPKM							Gene Description	Specific in episode
	FS	GI-M	GM-M	AN-M	GI-H	GM-H	AN-H		
MELO3C011118.2	17.367	17.934	8.817	24.954	14.506	60.668	12.948	Poly [ADP-ribose] polymerase	AN-M
MELO3C011116.2	0.545	0.330	0.265	0.622	0.296	10.311	0.487	transcription factor HBP-1b(C38)	AN-M
MELO3C011084.2	1.536	2.569	2.247	1.011	1.164	8.285	1.151	Ribosomal RNA small subunit methyltransferase A	AN-M
MELO3C011045.2	34.495	51.091	29.221	52.565	43.565	106.531	25.284	Histone H3	AN-M
MELO3C011030.2	9.110	11.447	4.201	8.912	9.238	41.455	7.910	5'-adenylylsulfate reductase-like 4	AN-M
MELO3C010918.2	9.925	8.801	1.884	10.707	3.585	27.486	3.640	1-acyl-sn-glycerol-3-phosphate acyltransferase	AN-M
MELO3C010842.2	14.619	12.678	10.727	16.080	9.149	47.831	13.825	E3 ubiquitin-protein ligase MBR2	AN-M
MELO3C010785.2	7.121	6.968	4.294	6.792	3.264	26.227	5.460	Transmembrane protein, putative	AN-M
MELO3C010762.2	21.032	28.093	7.340	18.912	13.310	83.745	10.049	Ferrochelatase	AN-M
MELO3C010746.2	30.777	22.198	7.962	28.931	15.603	68.055	18.201	Mitochondrial carrier protein	AN-M
MELO3C010691.2	1.297	1.658	0.563	0.941	0.606	5.190	NA	Octicosapeptide/Phox/Bem1p domain-containing protein kinase	AN-M
MELO3C010688.2	9.827	9.287	2.190	9.506	8.682	27.537	9.162	F-box protein At1g70590	AN-M
MELO3C010684.2	22.722	16.045	8.426	24.030	14.780	178.464	17.906	Lipase	AN-M
MELO3C010682.2	2.472	2.551	2.075	2.903	4.316	10.299	2.864	pectinesterase inhibitor-like	AN-M
MELO3C010677.2	1.021	0.817	1.253	1.507	1.300	5.778	1.752	protein trichome birefringence-like	AN-M
MELO3C010666.2	94.212	101.107	44.193	102.801	81.284	266.208	73.803	ADP-ribosylation factor-like	AN-M
MELO3C003314.2	2.993	3.522	6.789	4.279	2.455	38.908	5.864	Remorin	AN-M
MELO3C003324.2	1.553	1.992	2.635	1.522	1.113	4.608	1.119	transmembrane ascorbate ferrireductase 1	AN-M
MELO3C003329.2	7.267	7.622	2.193	5.679	7.428	21.446	7.546	FAD-dependent urate hydroxylase-like	AN-M
MELO3C003332.2	78.562	85.091	23.064	85.498	54.464	294.253	52.259	BAX inhibitor-1	AN-M
MELO3C003376.2	27.155	30.519	12.275	32.201	26.029	94.291	28.516	tobamovirus multiplication protein 2A	AN-M
MELO3C003418.2	3.342	4.300	7.062	1.890	1.707	10.653	1.379	PLATZ transcription factor family protein	AN-M
MELO3C003425.2	22.823	25.096	8.855	33.613	18.533	88.946	14.317	SNARE associated Golgi protein family	AN-M
MELO3C003426.2	7.609	10.407	5.371	12.743	10.598	45.852	9.119	Inositol-tetrakisphosphate 1-kinase	AN-M
MELO3C003429.2	1.955	3.624	2.766	2.003	2.412	12.044	2.709	E3 ubiquitin-protein ligase RNF14	AN-M
MELO3C003444.2	5.981	5.936	5.433	5.602	4.173	14.083	3.902	transcription factor IWS1	AN-M
MELO3C003447.2	9.302	8.841	2.572	9.149	5.892	28.076	6.313	Major facilitator superfamily transporter	AN-M
MELO3C003455.2	2.223	2.178	2.179	3.175	3.310	7.793	2.071	target of Myb protein 1-like isoform X1	AN-M
MELO3C003486.2	1.126	1.715	1.075	2.216	0.724	4.774	1.072	Receptor protein kinase, putative	AN-M
MELO3C003507.2	0.314	0.344	0.390	0.599	NA	2.345	0.229	Rhamnogalacturonate lyase family protein	AN-M

Gene ID	FPKM							Gene Description	Specific in episode
	FS	GI-M	GM-M	AN-M	GI-H	GM-H	AN-H		
MELO3C003518.2	2.741	3.630	0.730	4.113	0.806	121.125	0.550	serine/threonine-protein kinase CTR1	AN-M
MELO3C003526.2	2.528	1.267	1.073	1.537	0.558	12.003	0.392	AT-hook motif nuclear-localized protein 17	AN-M
MELO3C003542.2	3.634	4.029	4.782	2.793	2.322	11.192	2.984	Bet1-like protein family	AN-M
MELO3C003562.2	95.642	84.871	35.794	110.955	89.310	226.383	87.549	14-3-3-like protein	AN-M
MELO3C003565.2	1.741	0.620	1.427	2.039	1.655	5.201	2.035	Protein EXORDIUM-like 1	AN-M
MELO3C003572.2	4.341	5.690	4.677	4.196	3.493	17.276	2.949	14-3-3-like protein	AN-M
MELO3C003602.2	4.663	3.470	1.998	4.078	3.275	13.452	2.950	AIG2-like protein D	AN-M
MELO3C003606.2	0.348	0.333	0.339	0.414	0.161	2.732	0.137	U-box domain-containing protein 52	AN-M
MELO3C003645.2	17.457	15.366	9.126	19.385	14.957	52.228	13.628	Transmembrane protein, putative	AN-M
MELO3C003649.2	30.265	29.048	8.076	24.823	16.631	65.120	13.271	Transmembrane protein, putative	AN-M
MELO3C003680.2	0.400	0.355	0.975	0.575	0.443	3.501	0.337	Potassium channel AKT1	AN-M
MELO3C030466.2	0.885	0.836	1.785	1.065	0.508	2.164	0.397	FAR1-related sequence 6 isoform 1	AN-M
MELO3C003745.2	14.986	16.881	5.526	8.791	4.705	151.699	2.754	Thaumatin-like protein 1	AN-M
MELO3C003788.2	8.317	6.891	3.793	11.425	8.578	33.312	9.549	lon protease 2-like	AN-M
MELO3C003830.2	5.796	3.659	1.112	5.711	4.333	12.040	4.019	sugar transporter ERD6-like 6	AN-M
MELO3C003853.2	9.882	9.111	7.242	9.081	9.372	20.596	7.007	outer envelope pore protein 24, chloroplastic-like	AN-M
MELO3C003867.2	5.414	4.953	2.899	6.634	5.254	13.866	5.964	Alpha-1,6-mannosyl-glycoprotein 2-beta-N-acetylglucosaminyltransferase	AN-M
MELO3C018237.2	1.085	1.477	0.229	0.953	0.219	4.559	0.594	NAC domain-containing protein 18-like	AN-M
MELO3C018195.2	4.279	5.727	8.272	5.165	4.493	13.100	5.232	Arf GTPase activating protein	AN-M
MELO3C018134.2	2.709	2.499	5.261	2.756	2.354	8.047	3.553	Formin-like protein	AN-M
MELO3C018123.2	11.054	14.308	7.840	10.042	12.245	40.023	14.821	transcription factor ILR3-like	AN-M
MELO3C018112.2	2.907	3.952	2.459	3.679	2.448	10.854	2.860	Inositol-1,4,5-trisphosphate 5-phosphatase	AN-M
MELO3C018080.2	2.167	1.792	3.254	2.781	2.346	19.225	1.496	Septum-promoting GTP-binding protein 1	AN-M
MELO3C018066.2	14.592	19.811	6.255	13.579	15.854	40.177	12.943	Bet1-like SNARE 1-1 family protein	AN-M
MELO3C012812.2	4.550	5.669	5.895	6.345	7.424	14.809	6.293	Tetraspanin family protein, putative	AN-M
MELO3C012854.2	5.560	7.182	5.989	6.451	6.671	19.463	8.178	Long-chain-alcohol oxidase	AN-M
MELO3C012929.2	4.435	4.308	5.230	4.239	7.064	12.165	6.018	Transmembrane protein, putative	AN-M
MELO3C012960.2	1.941	1.122	4.090	1.030	1.307	5.359	1.138	BZIP protein, putative	AN-M
MELO3C012977.2	4.671	3.116	2.420	4.170	2.477	12.601	4.272	peroxisome biogenesis protein 2	AN-M
MELO3C013076.2	4.441	6.238	13.535	4.744	6.068	12.790	7.236	casein kinase I	AN-M

Gene ID	FPKM							Gene Description	Specific in episode
	FS	GI-M	GM-M	AN-M	GI-H	GM-H	AN-H		
MELO3C025790.2	42.450	32.662	11.220	46.303	25.671	102.181	28.974	mitogen-activated protein kinase kinase 5-like	AN-M
MELO3C022693.2	1.549	0.982	0.259	1.016	0.632	22.792	0.636	GDSL esterase/lipase 5	AN-M
MELO3C026723.2	2.932	3.177	0.814	4.359	2.327	9.613	3.479	Plastidal glycolate/glycerate translocator 1, chloroplastic	AN-M
MELO3C026712.2	7.997	10.059	4.136	8.651	9.076	23.096	8.645	Plastid lipid-associated protein	AN-M
MELO3C024032.2	2.318	2.441	2.029	2.722	2.735	5.853	3.126	S-acyltransferase	AN-M
MELO3C024000.2	1.786	3.425	2.119	3.247	2.124	9.956	2.217	Protein bps1, chloroplastic	AN-M
MELO3C023965.2	51.316	60.148	42.074	53.406	48.239	140.541	30.266	zinc finger A20 and AN1 domain-containing stress-associated protein 5-like	AN-M
MELO3C026902.2	2.600	3.304	9.650	4.393	5.377	12.659	4.585	Receptor protein kinase, putative	AN-M
MELO3C026885.2	3.685	3.894	2.545	4.271	3.232	11.996	2.228	Adenylyl-sulfate kinase	AN-M
MELO3C026602.2	1.391	1.701	2.085	1.120	1.672	4.918	1.748	Dual specificity protein phosphatase, putative	AN-M
MELO3C026597.2	5.551	5.973	5.150	5.514	5.461	14.058	6.863	ubiquitin-associated domain-containing protein 2	AN-M
MELO3C026581.2	0.840	1.029	0.426	1.668	0.484	3.372	0.750	F-box/LRR-repeat protein 15	AN-M
MELO3C009969.2	2.307	2.870	2.332	3.102	2.171	7.799	2.170	ADP-ribosylation factor-like	AN-M
MELO3C009963.2	2.068	1.876	0.568	3.577	0.941	18.466	1.065	Cinnamoyl-CoA reductase, putative	AN-M
MELO3C009944.2	1.051	1.622	1.360	1.842	0.808	5.195	1.423	Cyclic nucleotide-gated ion channel-like protein	AN-M
MELO3C009941.2	28.690	28.549	9.618	37.683	28.231	166.024	38.029	Potassium transporter	AN-M
MELO3C009929.2	6.749	5.845	8.428	6.610	5.113	17.376	6.145	BAH domain-containing protein	AN-M
MELO3C009898.2	8.931	8.396	7.387	11.965	7.612	45.664	8.458	beta-glucuronosyltransferase GlcAT14B	AN-M
MELO3C009863.2	3.359	1.683	0.786	2.206	0.773	20.457	1.116	F-box protein CPR30	AN-M
MELO3C009846.2	12.081	10.084	7.204	12.344	10.188	29.506	12.025	3-oxoacyl-[acyl-carrier-protein] reductase FabG-like	AN-M
MELO3C009778.2	7.710	5.666	3.038	5.086	2.940	21.717	4.107	Regulatory protein NPR5	AN-M
MELO3C009720.2	0.926	0.979	0.813	0.649	0.784	2.585	1.072	S-acyltransferase	AN-M
MELO3C009719.2	2.342	3.294	2.004	2.909	2.098	9.213	2.721	Aquaporin SIP1.1	AN-M
MELO3C009685.2	2.209	3.291	6.060	3.133	2.848	6.934	2.850	insulin-degrading enzyme-like 1, peroxisomal	AN-M
MELO3C009661.2	3.361	3.527	5.949	5.382	3.399	11.459	3.850	DNA-directed RNA polymerase subunit	AN-M
MELO3C009615.2	1.811	1.807	2.397	1.249	1.596	3.751	1.574	E3 ubiquitin-protein ligase RNF5	AN-M
MELO3C009569.2	2.795	3.342	2.890	5.609	1.730	25.309	2.453	Gb|AAF02136.1	AN-M
MELO3C009565.2	0.289	0.206	0.105	0.164	NA	4.552	NA	calcium-dependent protein kinase 26-like	AN-M
MELO3C009564.2	1.892	1.578	0.649	2.053	0.866	5.130	1.172	Mal d 1-associated protein	AN-M
MELO3C009553.2	15.610	11.568	4.199	12.532	8.981	41.138	10.052	CSL zinc finger domain-containing protein	AN-M

Gene ID	FPKM							Gene Description	Specific in episode
	FS	GI-M	GM-M	AN-M	GI-H	GM-H	AN-H		
MELO3C009506.2	0.804	0.399	0.077	1.222	NA	640.364	NA	secoisolariciresinol dehydrogenase-like	AN-M
MELO3C009503.2	5.034	5.253	0.625	6.319	3.502	27.967	2.406	IAA-amino acid hydrolase ILR1-like 3	AN-M
MELO3C009482.2	7.879	6.836	3.517	8.769	7.041	30.574	7.526	Rho GTPase-activating protein	AN-M
MELO3C009480.2	1.256	0.790	0.993	1.282	1.037	3.321	1.338	ABC transporter family protein	AN-M
MELO3C009470.2	3.625	4.219	1.541	6.214	1.629	40.384	2.055	Protein BIC1	AN-M
MELO3C009408.2	6.193	5.957	3.744	6.466	4.093	15.490	4.743	Tetratricopeptide repeat (TPR)-like superfamily protein	AN-M
MELO3C009407.2	4.475	4.586	5.605	5.605	5.021	12.187	4.148	Vacuolar protein-sorting-associated protein 37 homolog 1	AN-M
MELO3C009391.2	0.329	1.043	0.349	0.453	NA	11.096	0.467	Glycosyltransferase	AN-M
MELO3C009370.2	8.244	7.878	9.606	6.505	11.905	23.261	14.463	ACT domain-containing protein	AN-M
MELO3C009309.2	18.309	15.524	15.245	17.997	16.786	44.676	15.486	Nuclear transcription factor Y subunit B	AN-M
MELO3C009305.2	2.300	1.667	6.483	1.601	2.700	8.026	2.546	protein REVEILLE 8-like isoform X1	AN-M
MELO3C009301.2	0.316	0.431	1.524	0.502	0.338	1.187	0.257	Protein kinase-like protein	AN-M
MELO3C009294.2	0.096	0.180	0.028	0.387	NA	1.618	NA	Protein ALUMINUM SENSITIVE 3	AN-M
MELO3C009288.2	3.836	3.063	1.560	5.357	3.087	11.697	2.102	exocyst complex component EXO70B1-like	AN-M
MELO3C009276.2	6.168	7.097	10.125	5.386	5.434	19.843	5.583	Acyl-CoA N-acyltransferase with RING/FYVE/PHD-type zinc finger domain-containing protein	AN-M
MELO3C009271.2	0.645	0.924	6.963	0.373	1.048	18.136	0.267	Short-chain dehydrogenase TIC 32, chloroplastic	AN-M
MELO3C009226.2	6.372	5.538	3.250	6.704	6.672	16.246	6.828	serine/threonine-protein kinase ATG1c	AN-M
MELO3C009207.2	8.167	8.968	7.709	10.949	7.221	56.618	6.115	phosphatidylinositol:ceramide inositolphosphotransferase 1	AN-M
MELO3C009193.2	4.761	4.105	2.736	5.055	4.013	12.326	3.396	Transmembrane protein	AN-M
MELO3C009170.2	0.445	0.843	0.110	0.738	0.118	3.561	0.138	vacuolar cation/proton exchanger 3-like	AN-M
MELO3C009166.2	4.913	6.070	4.402	6.718	5.249	14.239	5.629	CTD small phosphatase-like protein	AN-M
MELO3C009164.2	1.779	1.849	2.223	1.741	1.394	8.802	1.499	PHD finger protein ALFIN-LIKE 1-like	AN-M
MELO3C009146.2	1.761	1.538	2.176	2.206	1.089	12.726	2.028	DnaJ protein ERDJ3A	AN-M
MELO3C009143.2	0.384	0.959	0.381	0.449	0.356	1.978	0.149	Receptor-like protein	AN-M
MELO3C009092.2	1.139	0.961	0.309	0.686	0.572	5.830	0.381	Protein LURP-one-related 17	AN-M
MELO3C009089.2	3.571	3.676	8.140	4.604	5.691	13.591	5.823	Receptor protein kinase, putative	AN-M
MELO3C014726.2	0.468	0.489	1.409	0.338	0.470	1.180	0.494	Unknown protein	AN-M
MELO3C014678.2	28.191	24.576	5.672	35.608	7.752	123.636	8.755	Kelch repeat-containing F-box family protein	AN-M
MELO3C014662.2	16.842	16.133	19.471	14.108	19.158	45.794	23.097	Casein kinase, putative	AN-M
MELO3C014647.2	0.743	0.722	2.859	0.847	0.390	1.781	0.408	Early endosome antigen 1	AN-M

Gene ID	FPKM							Gene Description	Specific in episode
	FS	GI-M	GM-M	AN-M	GI-H	GM-H	AN-H		
MELO3C014617.2	0.418	1.058	0.327	0.730	0.933	3.132	0.493	NB-ARC domain containing protein, expressed	AN-M
MELO3C014562.2	25.750	25.545	11.498	25.664	17.403	58.697	17.064	E3 ubiquitin-protein ligase ICP0	AN-M
MELO3C014551.2	13.397	18.658	9.512	16.683	6.163	75.730	8.465	F-box protein	AN-M
MELO3C014519.2	0.742	0.485	1.399	1.081	0.676	8.975	1.198	BEL1-like homeodomain protein 1	AN-M
MELO3C014483.2	6.051	6.466	7.495	9.210	5.160	19.498	5.248	Vacuole membrane protein KMS1	AN-M
MELO3C014433.2	0.800	0.681	0.826	0.999	1.392	2.409	1.384	clathrin coat assembly protein AP180	AN-M
MELO3C014420.2	1.943	3.254	2.535	3.447	2.970	33.765	3.153	Transmembrane protein, putative	AN-M
MELO3C014384.2	1.203	1.531	1.996	1.506	1.045	4.574	1.205	ABSCISIC ACID-INSENSITIVE 5-like protein 4 isoform X1	AN-M
MELO3C014352.2	2.263	1.677	1.429	3.010	1.999	6.027	2.042	3-oxoacyl-[acyl-carrier-protein] synthase-like protein	AN-M
MELO3C014347.2	1.790	2.826	1.044	1.397	0.495	6.849	1.596	EID1-like F-box protein 3	AN-M
MELO3C014341.2	1.793	1.573	2.598	1.088	1.780	3.853	1.514	Intracellular protein transport protein USO1 isoform 3	AN-M
MELO3C014334.2	36.241	47.424	85.902	45.971	59.125	100.912	52.748	histone H1	AN-M
MELO3C014307.2	7.921	6.777	2.775	8.199	9.211	26.994	7.347	PRA1 family protein	AN-M
MELO3C014302.2	17.774	17.531	5.756	18.093	14.330	54.496	15.616	Bax inhibitor	AN-M
MELO3C014280.2	0.997	1.206	0.276	1.772	0.564	32.060	0.596	ATP-sulfurylase	AN-M
MELO3C014256.2	0.035	0.067	0.049	0.094	0.029	2.956	0.024	Transmembrane protein, putative	AN-M
MELO3C014254.2	55.592	56.194	12.051	51.767	25.567	131.667	22.361	Cell number regulator 8	AN-M
MELO3C014242.2	2.432	2.554	3.549	2.254	1.946	6.012	2.281	Ecotropic viral integration site 5 protein isogeny	AN-M
MELO3C014240.2	2.297	2.340	0.434	3.141	1.681	11.114	1.710	Aquaporin PIP2	AN-M
MELO3C023022.2	6.740	5.652	3.613	5.242	6.765	21.932	9.801	protein NRT1/ PTR FAMILY 5.2-like	AN-M
MELO3C023041.2	0.150	0.228	0.175	0.281	0.123	1.119	0.151	Zinc finger-like protein	AN-M
MELO3C023066.2	36.749	30.518	10.806	40.073	22.454	102.595	26.516	Transducin family protein / WD-40 repeat family protein	AN-M
MELO3C023069.2	15.080	14.057	3.244	12.900	9.618	32.575	10.200	Equilibrative nucleoside transporter	AN-M
MELO3C023078.2	0.722	1.318	1.688	1.342	0.851	3.948	0.620	LRR receptor-like kinase family protein	AN-M
MELO3C008520.2	3.060	2.681	2.661	2.775	3.051	6.392	3.643	Cysteine protease	AN-M
MELO3C008543.2	3.654	3.703	1.437	4.793	2.787	14.143	1.941	sphinganine C4-monooxygenase 1-like	AN-M
MELO3C008555.2	3.896	2.410	1.414	3.573	1.883	8.482	2.401	Transmembrane protein, putative	AN-M
MELO3C008559.2	3.389	2.904	3.159	4.476	2.898	13.134	2.591	aspartic proteinase-like protein 2	AN-M
MELO3C008571.2	33.371	37.925	30.080	37.466	36.977	96.979	33.048	Basic leucine zipper and W2 domain-containing protein 2	AN-M
MELO3C008714.2	2.768	3.438	6.106	3.665	2.917	11.257	2.848	Transcription factor jumonji family protein	AN-M
MELO3C008717.2	1.760	1.839	3.318	2.222	1.524	4.898	1.676	Isopentenyl diphosphate isomerase	AN-M

Gene ID	FPKM							Gene Description	Specific in episode
	FS	GI-M	GM-M	AN-M	GI-H	GM-H	AN-H		
MELO3C031452.2	1.444	2.133	3.031	2.313	0.901	6.558	0.949	Amino acid permease	AN-M
MELO3C008752.2	2.990	3.095	2.657	2.761	2.695	11.363	2.198	TATA box-binding protein-associated factor RNA polymerase I subunit B	AN-M
MELO3C003908.2	11.857	14.012	6.890	12.253	9.081	39.962	10.917	Serine/Threonine kinase family protein	AN-M
MELO3C003911.2	0.149	0.156	0.427	0.370	0.081	1.708	0.269	protein BONZAI 3	AN-M
MELO3C003959.2	1.219	1.371	1.978	1.281	1.101	3.078	1.288	CLEC16A-like protein	AN-M
MELO3C004003.2	2.149	1.883	0.884	2.371	0.642	12.114	0.810	La-related protein 6 isoform 1	AN-M
MELO3C004028.2	0.570	0.875	2.277	0.723	1.088	1.921	0.518	Syntaxin/T-SNARE family protein	AN-M
MELO3C004040.2	3.233	4.325	2.958	3.164	3.096	15.077	2.403	Chaperone DnaJ	AN-M
MELO3C004050.2	7.906	5.902	1.256	11.106	5.712	62.259	6.100	zinc finger protein CONSTANS-LIKE 5-like	AN-M
MELO3C004061.2	0.820	0.479	2.631	0.244	0.203	64.441	NA	(R)-mandelonitrile lyase 1-like	AN-M
MELO3C004078.2	3.061	2.167	0.718	2.365	1.658	7.906	1.803	Protein DETOXIFICATION	AN-M
MELO3C004133.2	8.934	9.985	5.147	9.445	7.786	25.095	10.295	Cyclic nucleotide-gated ion channel, putative	AN-M
MELO3C004170.2	2.819	4.328	3.225	4.293	3.719	27.434	3.110	Neutral/alkaline invertase	AN-M
MELO3C004211.2	0.166	0.485	0.961	0.250	0.091	1.066	0.229	Phospholipase D	AN-M
MELO3C004274.2	5.441	8.125	5.614	6.340	5.412	16.886	2.681	peroxisomal adenine nucleotide carrier 1-like	AN-M
MELO3C004286.2	6.032	6.274	1.402	9.720	1.949	20.492	1.686	E3 ubiquitin-protein ligase ATL6	AN-M
MELO3C004289.2	0.452	0.929	1.417	0.499	0.754	1.991	0.420	TMV resistance protein N-like	AN-M
MELO3C004299.2	0.254	0.415	0.428	0.590	0.943	4.069	1.094	GDSL esterase/lipase At1g54790-like	AN-M
MELO3C004334.2	8.864	10.050	5.295	9.700	9.172	29.254	8.175	ERAD-associated E3 ubiquitin-protein ligase HRD1B-like	AN-M
MELO3C004434.2	2.163	2.364	2.238	2.027	1.666	5.112	2.108	Serine-rich protein-like protein	AN-M
MELO3C004460.2	2.897	2.151	2.877	2.469	2.651	8.143	2.019	BRCA1-associated protein	AN-M
MELO3C004464.2	9.360	11.276	9.539	10.808	9.920	27.057	8.439	Ubiquitin system component Cue	AN-M
MELO3C004465.2	5.066	7.430	2.322	7.957	2.560	31.119	1.530	calmodulin-like protein 7	AN-M
MELO3C004476.2	8.226	7.988	2.573	10.116	6.805	28.255	6.691	Major facilitator superfamily	AN-M
MELO3C004478.2	2.803	4.278	2.251	3.757	4.013	21.634	2.005	Unknown protein	AN-M
MELO3C004498.2	11.196	10.502	3.191	9.691	6.024	24.869	9.165	EIN3-binding F box protein 1	AN-M
MELO3C004544.2	1.807	2.280	1.598	2.117	1.753	12.520	1.902	2-oxoglutarate (2OG) and Fe(II)-dependent oxygenase superfamily protein	AN-M
MELO3C004560.2	2.681	3.255	4.154	2.043	3.609	6.593	3.534	Protein LIKE COV 1	AN-M
MELO3C004603.2	11.519	8.975	3.226	9.594	10.599	48.292	9.748	Protein-tyrosine phosphatase mitochondrial 1	AN-M
MELO3C004611.2	0.838	1.515	5.676	0.884	1.326	3.669	1.547	vacuolar protein sorting-associated protein 32 homolog 2	AN-M

Gene ID	FPKM							Gene Description	Specific in episode
	FS	GI-M	GM-M	AN-M	GI-H	GM-H	AN-H		
MELO3C004631.2	19.735	18.019	20.631	21.187	17.575	45.111	25.728	casein kinase I	AN-M
MELO3C004643.2	3.646	1.796	1.069	3.926	1.071	14.016	1.337	replication protein A 70 kDa DNA-binding subunit A	AN-M
MELO3C006062.2	1.318	1.860	1.499	2.608	2.888	23.891	3.421	Arabinogalactan peptide-like protein	AN-M
MELO3C006074.2	0.439	0.747	3.023	0.749	0.928	1.885	0.497	U11/U12 small nuclear ribonucleoprotein 59 kDa protein isoform X1	AN-M
MELO3C006084.2	8.708	8.502	4.021	8.972	6.247	18.661	5.663	Erythronate-4-phosphate dehydrogenase family protein, putative	AN-M
MELO3C006093.2	5.997	3.009	2.000	5.918	1.953	41.432	0.862	UDP-glycosyltransferase 91A1-like	AN-M
MELO3C006109.2	8.943	7.812	8.146	7.403	8.515	30.406	8.504	Phosphatidylinositol N-acetyglucosaminlytransferase subunit P-like protein	AN-M
MELO3C006160.2	14.423	13.906	11.907	10.382	11.143	34.476	14.328	Histone deacetylase complex subunit SAP18	AN-M
MELO3C006168.2	0.894	0.943	4.720	1.619	2.069	4.839	1.292	Cytochrome P450	AN-M
MELO3C006268.2	4.855	5.827	2.875	6.000	9.651	16.151	8.214	10 kDa chaperonin isoform X1	AN-M
MELO3C006275.2	3.741	4.693	4.950	5.470	4.665	11.542	5.273	Metacaspase-1	AN-M
MELO3C006288.2	0.540	0.599	2.175	0.608	0.375	1.863	0.226	Heat Stress Transcription Factor family protein	AN-M
MELO3C006326.2	0.254	0.081	0.419	0.600	0.034	4.460	NA	Cellulose synthase	AN-M
MELO3C006332.2	1.648	1.357	3.750	1.177	1.693	3.353	1.539	E3 ubiquitin-protein ligase SDIR1	AN-M
MELO3C006365.2	0.947	0.508	0.366	0.980	0.899	91.300	0.581	FBT8	AN-M
MELO3C006384.2	7.548	13.454	8.723	8.407	10.766	26.968	10.060	Proton gradient regulation 5	AN-M
MELO3C006404.2	0.535	0.837	0.583	0.622	0.298	5.388	0.359	phosphate transporter PHO1	AN-M
MELO3C006434.2	11.953	16.444	3.587	15.329	6.162	44.426	6.988	electron transfer flavoprotein subunit beta, mitochondrial	AN-M
MELO3C006483.2	0.697	0.612	0.160	0.519	0.511	9.621	0.709	Non-specific serine/threonine protein kinase	AN-M
MELO3C006505.2	7.118	8.706	2.421	12.577	2.777	116.886	3.402	sulfate transporter 3.1	AN-M
MELO3C006509.2	4.492	4.144	31.164	4.521	8.524	17.029	8.374	protein NETWORKED 1A	AN-M
MELO3C006519.2	3.488	2.546	2.643	2.820	2.048	13.577	0.959	non-specific lipid-transfer protein-like protein At2g13820	AN-M
MELO3C006554.2	0.761	1.333	2.950	1.372	0.736	3.676	0.865	Unknown protein	AN-M
MELO3C006579.2	1.955	2.149	0.740	3.039	1.101	23.282	1.199	BSD domain-containing protein, putative	AN-M
MELO3C006615.2	20.058	20.862	11.185	25.406	16.757	92.591	22.058	RING finger and CHY zinc finger protein	AN-M
MELO3C006617.2	8.706	10.090	4.437	8.251	5.470	29.658	5.828	lipase-like	AN-M
MELO3C006656.2	8.482	6.763	1.964	9.393	7.643	35.278	4.644	Protein SRC2	AN-M
MELO3C006690.2	13.266	9.665	6.311	16.618	16.305	44.642	19.863	Pectin lyase-like superfamily protein	AN-M
MELO3C006691.2	3.038	2.815	1.051	4.663	2.004	11.931	2.648	extensin-like	AN-M

Gene ID	FPKM							Gene Description	Specific in episode
	FS	GI-M	GM-M	AN-M	GI-H	GM-H	AN-H		
MELO3C006703.2	21.024	22.200	9.086	19.503	14.897	45.324	17.885	CoA ligase	AN-M
MELO3C006741.2	1.547	2.049	2.584	1.683	1.714	4.155	1.624	Calcium-dependent lipid-binding family protein	AN-M
MELO3C006771.2	1.448	2.062	0.481	2.949	0.431	23.432	1.678	auxin-responsive protein SAUR36-like	AN-M
MELO3C006789.2	1.380	1.622	4.332	1.989	1.338	9.653	1.349	transcription factor bHLH62-like	AN-M
MELO3C006841.2	6.114	6.309	5.099	8.800	4.632	26.842	6.112	K(+) efflux antiporter 5	AN-M
MELO3C006845.2	0.964	1.489	1.960	2.277	3.607	5.928	2.462	Embryo-specific protein 3	AN-M
MELO3C006862.2	0.330	0.756	0.134	0.859	NA	4.201	NA	Peroxidase	AN-M
MELO3C031824.2	0.598	0.510	1.304	0.719	0.612	2.008	0.566	DUF4283 domain-containing protein	AN-M
MELO3C006969.2	6.439	8.962	8.955	4.673	9.084	69.587	10.013	ABC transporter C family member 10	AN-M
MELO3C006983.2	1.261	2.042	1.823	1.403	1.708	5.770	2.022	Protein phosphatase 2C	AN-M
MELO3C026930.2	10.680	11.899	8.162	12.657	10.908	55.074	13.056	Abscisic acid receptor PYL8	AN-M
MELO3C008489.2	9.204	14.295	3.182	7.885	13.236	31.478	11.453	Serine/threonine-protein kinase stt7, chloroplastic	AN-M
MELO3C008494.2	6.954	7.379	4.331	8.333	7.598	20.265	8.136	E3 ubiquitin-protein ligase	AN-M
MELO3C019373.2	11.601	9.820	4.359	11.902	9.529	47.943	11.646	Protein phosphatase 2C	AN-M
MELO3C019389.2	7.099	12.842	19.484	10.946	8.642	42.460	7.848	transcription elongation factor 1 homolog	AN-M
MELO3C019395.2	0.602	0.431	0.937	0.607	0.634	1.264	0.627	LOW QUALITY PROTEIN: uncharacterized protein LOC103496210	AN-M
MELO3C019399.2	4.480	3.916	4.717	4.892	3.253	10.669	4.175	p-loop nucleoside triphosphate hydrolase superfamily protein, putative	AN-M
MELO3C019429.2	0.891	1.033	3.036	1.194	0.462	3.521	1.213	Basic-leucine zipper (BZIP) transcription factor family protein	AN-M
MELO3C019485.2	11.603	14.016	3.233	9.771	6.224	28.400	7.198	Patatin	AN-M
MELO3C019504.2	14.045	14.277	6.252	14.106	7.370	31.401	8.238	Meiosis arrest female protein 1-like protein	AN-M
MELO3C020328.2	1.563	2.757	0.662	2.105	0.810	26.265	0.781	aspartic proteinase PCS1	AN-M
MELO3C031717.2	0.788	1.453	0.515	1.254	0.825	5.351	1.338	E3 ubiquitin-protein ligase MIEL1	AN-M
MELO3C020316.2	1.308	2.823	2.429	2.095	1.791	7.438	0.973	RING/FYVE/PHD zinc finger protein	AN-M
MELO3C020291.2	6.448	11.595	2.612	6.635	2.949	26.884	6.543	Little protein 1	AN-M
MELO3C020276.2	0.607	1.102	0.957	0.835	1.237	2.555	1.486	Tobamovirus multiplication 1	AN-M
MELO3C032039.2	3.427	3.797	0.438	3.363	1.661	8.657	1.792	Thioredoxin-like protein	AN-M
MELO3C020263.2	10.358	6.296	13.945	8.208	5.719	38.903	3.541	Calcium binding protein	AN-M
MELO3C020192.2	3.627	3.785	4.403	2.732	3.448	9.542	3.827	ATA15 protein	AN-M
MELO3C023731.2	1.208	1.242	1.633	1.387	1.221	4.152	1.321	phosphatidylcholine transfer protein-like isoform X1	AN-M
MELO3C023775.2	3.043	2.747	3.016	4.140	4.625	23.005	5.056	isoprenylcysteine alpha-carbonyl methylesterase ICME-like	AN-M

Gene ID	FPKM							Gene Description	Specific in episode
	FS	GI-M	GM-M	AN-M	GI-H	GM-H	AN-H		
MELO3C023787.2	454.389	441.005	147.785	387.988	266.781	1359.398	312.525	S-adenosylmethionine decarboxylase proenzyme	AN-M
MELO3C014972.2	3.096	2.316	2.247	3.058	2.746	10.451	2.904	Nucleobase-ascorbate transporter-like protein	AN-M
MELO3C014900.2	20.315	17.543	4.969	21.744	14.896	62.575	17.846	Metal-nicotianamine transporter	AN-M
MELO3C014897.2	8.100	9.375	4.391	11.381	7.725	91.224	8.231	Short-chain dehydrogenase/reductase family protein	AN-M
MELO3C014882.2	3.464	2.269	0.767	4.970	3.261	27.542	3.316	Peptidase family M48 family protein	AN-M
MELO3C014879.2	0.380	0.507	0.106	0.203	0.168	3.128	0.450	Cytochrome P450 family protein	AN-M
MELO3C014826.2	1.482	1.249	2.765	0.851	1.393	4.517	1.278	Pentatricopeptide repeat-containing family protein	AN-M
MELO3C014760.2	8.899	7.588	4.369	9.742	7.983	21.261	8.140	Magnesium transporter NIPA	AN-M
MELO3C014759.2	1.147	1.923	3.622	3.006	1.439	7.842	0.614	Avr9/Cf-9 rapidly elicited protein	AN-M
MELO3C016568.2	0.416	0.914	1.455	0.464	0.723	2.085	0.499	Diacylglycerol kinase	AN-M
MELO3C016550.2	3.104	3.236	4.580	3.239	2.547	8.000	2.460	Unknown protein	AN-M
MELO3C016541.2	24.196	25.452	7.472	20.788	14.728	76.599	14.226	Peroxisomal membrane 22 kDa (Mpv17/PMP22) family protein	AN-M
MELO3C016513.2	4.982	16.091	23.339	16.904	0.957	63.535	1.940	Metallothionein	AN-M
MELO3C016512.2	1.875	3.057	5.638	2.478	0.865	6.581	0.621	Cytochrome c oxidase copper chaperone	AN-M
MELO3C016496.2	0.223	0.148	0.221	0.419	0.293	1.174	0.137	Octicosapeptide/phox/Bem1p domain kinase superfamily protein	AN-M
MELO3C016420.2	1.507	1.789	1.097	1.586	0.880	8.024	1.503	TVP38/TMEM64 family membrane protein slr0305 family	AN-M
MELO3C016418.2	3.401	4.023	3.813	3.309	3.955	9.602	3.592	Mechanosensitive ion channel protein 2, chloroplastic	AN-M
MELO3C025406.2	13.615	15.203	9.548	14.951	14.154	34.637	15.047	TBC1 domain family member 15	AN-M
MELO3C013711.2	0.090	0.223	0.422	0.176	NA	7.049	0.005	Auxin efflux carrier family protein	AN-M
MELO3C013724.2	6.008	6.746	5.005	6.470	5.464	17.058	5.865	Cancer-related nucleoside-triphosphatase	AN-M
MELO3C013727.2	0.615	0.786	1.297	0.998	1.204	4.319	1.070	WUSCHEL-related homeobox 13	AN-M
MELO3C013740.2	5.347	7.255	5.828	5.492	5.143	20.982	6.599	bifunctional nuclease 2 isoform X1	AN-M
MELO3C013758.2	1.823	1.930	3.182	2.167	1.476	4.481	1.189	Histidine kinase 1	AN-M
MELO3C013784.2	1.146	1.055	0.507	1.982	0.318	4.566	0.324	Kelch repeat-containing F-box family protein	AN-M
MELO3C013799.2	26.463	29.629	30.932	32.010	26.380	89.022	27.802	cyclin-dependent kinase F-4-like isoform X1	AN-M
MELO3C013813.2	4.113	2.935	2.107	6.762	3.150	16.514	2.731	Cystic fibrosis transmembrane conductance regulator	AN-M
MELO3C013834.2	3.044	4.021	8.653	4.216	4.545	9.898	3.788	syntaxin-22	AN-M
MELO3C013854.2	6.990	7.856	2.814	5.858	6.951	18.839	6.915	Abscisic acid receptor	AN-M
MELO3C013865.2	4.023	5.175	3.022	4.712	4.865	10.554	4.849	Ubiquitin family protein	AN-M
MELO3C013904.2	1.932	2.491	4.493	2.860	2.033	11.014	2.949	GATA transcription factor 26-like	AN-M

Gene ID	FPKM							Gene Description	Specific in episode
	FS	GI-M	GM-M	AN-M	GI-H	GM-H	AN-H		
MELO3C013920.2	0.395	0.869	0.182	0.338	0.163	2.681	0.361	palmitoyl-protein thioesterase 1-like	AN-M
MELO3C013922.2	0.627	0.666	1.332	1.724	0.628	3.467	0.885	Glycoprotein	AN-M
MELO3C013940.2	8.742	10.920	5.961	8.548	6.678	23.829	6.453	peroxisome biogenesis protein 19-1-like	AN-M
MELO3C013941.2	0.659	1.360	0.759	0.393	0.433	3.007	0.242	Glycogenin-2	AN-M
MELO3C013943.2	2.749	2.914	0.853	3.864	3.220	78.687	4.276	aspartic proteinase nepenthesin-1	AN-M
MELO3C013988.2	32.742	34.543	16.451	38.911	23.839	81.006	27.097	Myelodysplasia-myeloid leukemia factor 1-interacting protein	AN-M
MELO3C013991.2	4.601	2.829	2.226	4.468	3.778	12.949	3.546	methylsterol monooxygenase 1-2-like	AN-M
MELO3C014004.2	0.207	0.147	0.519	0.183	0.227	1.559	0.213	S-type anion channel	AN-M
MELO3C014022.2	10.689	12.499	3.932	12.295	9.143	36.856	9.538	E3 ubiquitin-protein ligase family	AN-M
MELO3C014028.2	7.520	6.623	4.393	8.932	5.247	21.112	6.654	Peroxisome biogenesis protein 5	AN-M
MELO3C014057.2	13.589	9.734	10.215	14.320	9.243	30.909	12.460	Plant UBX domain-containing protein 8	AN-M
MELO3C031971.2	7.208	8.989	4.792	6.640	2.220	27.412	5.515	Xanthine dehydrogenase, putative	AN-M
MELO3C014123.2	10.337	11.661	12.146	11.504	11.517	29.372	11.098	Transducin/WD40 repeat-like superfamily protein	AN-M
MELO3C014127.2	5.532	5.378	2.509	5.893	3.978	21.182	4.615	Actin-related protein 8	AN-M
MELO3C014150.2	1.550	2.645	5.193	2.453	1.882	8.542	1.534	Fimbrin, putative	AN-M
MELO3C014157.2	2.931	4.919	7.319	4.399	4.616	13.590	3.882	Rab3 GTPase-activating protein catalytic subunit	AN-M
MELO3C014172.2	1.112	1.188	1.578	1.062	1.005	2.650	1.170	Protein HOMOLOG OF MAMMALIAN LYST-INTERACTING PROTEIN 5	AN-M
MELO3C032296.2	15.446	14.734	1.816	16.613	7.053	35.607	9.119	Unknown protein	AN-M
MELO3C017071.2	0.730	0.402	0.149	1.356	0.531	3.209	0.241	Cysteine/Histidine-rich C1 domain family protein	AN-M
MELO3C017064.2	18.243	16.110	12.446	12.471	7.832	65.411	6.380	bZIP transcription factor 60	AN-M
MELO3C017061.2	33.510	41.311	10.358	35.738	19.455	100.303	29.854	Cinnamoyl-CoA reductase, putative	AN-M
MELO3C017049.2	10.365	9.204	4.553	9.361	6.843	31.504	6.400	protein PLASTID MOVEMENT IMPAIRED 1	AN-M
MELO3C017031.2	83.914	81.553	221.643	64.891	82.601	184.685	92.797	High mobility group B protein 2	AN-M
MELO3C017023.2	75.865	63.723	15.266	99.157	35.811	371.339	35.116	Catalase	AN-M
MELO3C017008.2	9.657	7.839	7.501	9.655	8.907	20.329	9.344	Ubiquitin family protein	AN-M
MELO3C017002.2	0.283	0.481	0.364	1.014	NA	2.966	0.186	Alpha-amylase	AN-M
MELO3C016962.2	2.927	3.753	2.474	2.563	1.910	7.657	1.820	Chorismate mutase	AN-M
MELO3C016935.2	5.091	5.768	5.704	4.699	6.023	12.177	4.766	ubiquitin carboxyl-terminal hydrolase 18-like	AN-M
MELO3C016933.2	2.043	2.587	5.700	3.946	3.364	10.076	1.886	E3 ubiquitin-protein ligase AIP2	AN-M
MELO3C016924.2	14.642	13.410	11.466	10.269	14.072	34.832	17.251	pentatricopeptide repeat-containing protein At4g33170-like	AN-M
MELO3C016917.2	3.370	3.228	1.786	2.184	2.593	14.076	2.350	Damaged dna-binding 2, putative isoform 1	AN-M

Gene ID	FPKM							Gene Description	Specific in episode
	FS	GI-M	GM-M	AN-M	GI-H	GM-H	AN-H		
MELO3C016875.2	16.993	15.880	15.452	19.977	19.591	51.485	24.381	ATP-dependent zinc metalloprotease FTSH 4, mitochondrial	AN-M
MELO3C016868.2	1.908	1.670	2.404	2.396	1.334	7.552	1.841	serine/threonine-protein kinase EDR1	AN-M
MELO3C016866.2	3.501	7.016	2.840	6.127	1.120	15.211	4.295	Reversion-to-ethylene sensitivity1-like protein	AN-M
MELO3C016855.2	87.271	77.743	66.599	115.269	86.920	502.613	59.332	CRG16	AN-M
MELO3C016813.2	12.196	15.741	39.328	12.174	12.635	31.531	11.109	Interferon-related developmental regulator family protein	AN-M
MELO3C016789.2	22.964	26.155	8.637	21.105	19.573	107.491	17.959	transcription factor MYB44-like	AN-M
MELO3C016787.2	5.789	9.973	1.023	16.193	1.624	71.197	1.777	L-asparaginase	AN-M
MELO3C016753.2	2.677	4.231	2.520	3.873	2.972	20.014	2.479	Coenzyme Q-binding protein	AN-M
MELO3C016751.2	7.180	10.281	6.039	8.942	4.920	28.032	5.192	Peroxisomal multifunctional enzyme type 2	AN-M
MELO3C016749.2	1.082	1.142	2.456	0.760	1.191	3.098	0.748	Protein WEAK CHLOROPLAST MOVEMENT UNDER BLUE LIGHT 1	AN-M
MELO3C016737.2	2.564	3.325	1.877	3.909	2.336	7.840	3.205	Alpha/beta-Hydrolases superfamily protein	AN-M
MELO3C016694.2	1.580	1.632	2.495	2.706	1.808	7.772	1.521	VQ motif-containing protein	AN-M
MELO3C016640.2	0.203	0.457	0.602	0.385	0.295	1.591	0.262	Xanthine dehydrogenase/oxidase	AN-M
MELO3C022961.2	34.172	48.821	9.786	40.219	14.448	134.138	13.423	4-hydroxyphenylpyruvate dioxygenase	AN-M
MELO3C026966.2	2.445	2.185	3.241	1.642	2.829	5.613	2.485	2-oxoglutarate (2OG) and Fe(II)-dependent oxygenase superfamily protein	AN-M
MELO3C026968.2	0.619	1.673	0.690	1.042	1.697	4.063	0.612	Myosin-G heavy chain-like protein	AN-M
MELO3C026974.2	9.939	12.907	4.230	6.998	5.003	42.949	4.067	aspartyl protease family protein 2	AN-M
MELO3C010538.2	2.122	1.730	1.225	2.345	1.443	5.234	2.029	RNA-binding (RRM/RBD/RNP motifs) family protein	AN-M
MELO3C010495.2	0.823	1.457	0.387	1.306	0.238	19.059	0.193	chaperone protein DnaJ	AN-M
MELO3C010494.2	1.359	2.125	2.943	1.737	2.315	19.738	2.326	Aldehyde dehydrogenase	AN-M
MELO3C010430.2	0.939	0.698	0.486	0.871	0.410	7.637	0.321	At1g78110	AN-M
MELO3C010423.2	1.801	2.623	1.788	1.647	0.929	7.355	0.646	Transducin/WD40 repeat-like superfamily protein	AN-M
MELO3C010422.2	4.742	4.179	5.348	4.903	4.792	11.465	4.671	P-loop containing nucleoside triphosphate hydrolase superfamily protein	AN-M
MELO3C010381.2	16.787	16.126	2.671	15.839	10.693	82.615	11.174	2-hydroxyacyl-CoA lyase	AN-M
MELO3C010371.2	0.367	0.351	2.092	0.745	0.310	3.557	0.231	transcription factor TCP17-like isoform X1	AN-M
MELO3C018930.2	0.233	0.172	1.421	0.291	0.282	1.792	0.163	zinc finger protein CONSTANS-LIKE 2	AN-M
MELO3C018977.2	2.509	2.137	2.837	1.656	2.191	5.508	1.574	TLD-domain containing nucleolar protein	AN-M
MELO3C024865.2	60.927	56.488	31.756	63.798	56.670	133.498	61.228	NAC domain-containing protein 62	AN-M
MELO3C024848.2	5.000	4.789	1.571	10.919	4.123	31.816	2.706	Serine-type endopeptidase inhibitor	AN-M
MELO3C016084.2	3.331	4.353	4.308	3.573	2.439	11.555	2.317	Subtilisin-like protease	AN-M

Gene ID	FPKM							Gene Description	Specific in episode
	FS	GI-M	GM-M	AN-M	GI-H	GM-H	AN-H		
MELO3C016115.2	25.511	28.649	6.740	29.136	20.316	195.676	23.279	B-box zinc finger protein 22	AN-M
MELO3C016154.2	1.672	1.327	4.455	1.172	1.294	3.392	1.375	protein SIP5	AN-M
MELO3C016180.2	11.400	9.173	6.475	14.376	10.502	30.516	10.496	FAD/NAD(P)-binding oxidoreductase family protein	AN-M
MELO3C016196.2	12.073	8.460	4.925	11.510	7.429	35.908	9.878	Topoisomerase II-associated protein PAT1, putative isoform 1	AN-M
MELO3C016220.2	88.758	107.730	39.340	110.502	69.989	433.281	62.096	Glycine and proline-rich protein	AN-M
MELO3C016270.2	0.523	1.014	0.384	0.296	0.179	2.787	0.178	cellulose synthase-like protein G3	AN-M
MELO3C016292.2	0.720	0.589	0.095	1.312	0.279	4.009	0.495	Ring finger protein, putative	AN-M
MELO3C016351.2	5.603	7.853	3.645	8.078	3.985	27.975	3.400	Haloacid dehalogenase-like hydrolase superfamily protein	AN-M
MELO3C017537.2	6.044	5.826	14.618	5.659	4.811	25.983	4.699	zinc finger CCCH domain-containing protein 44-like isoform X2	AN-M
MELO3C017590.2	14.854	16.558	6.964	14.296	8.570	87.872	6.976	Acyl-coenzyme A oxidase	AN-M
MELO3C017600.2	17.248	17.273	5.591	17.049	13.243	58.670	13.054	LanC-like protein GCL1	AN-M
MELO3C032618.2	62.098	57.542	8.324	58.707	46.968	177.412	38.885	LanC-like protein GCL1	AN-M
MELO3C017606.2	0.664	1.095	2.314	1.080	1.113	3.340	1.553	Polyadenylate-binding protein 2	AN-M
MELO3C017612.2	3.593	3.412	2.386	3.055	2.324	18.681	3.456	Alpha/beta-hydrolase superfamily protein	AN-M
MELO3C017618.2	3.739	5.302	1.108	5.864	3.145	13.449	1.931	Chitin synthase, putative (DUF1218)	AN-M
MELO3C017630.2	1.742	5.179	0.639	2.381	3.598	11.075	3.140	At5g61660	AN-M
MELO3C017694.2	7.448	4.128	2.323	10.304	8.472	23.411	9.902	Unknown protein	AN-M
MELO3C017700.2	0.210	0.310	2.631	0.482	0.330	1.450	0.206	disease resistance protein RGA2-like	AN-M
MELO3C017731.2	5.534	6.791	8.326	6.893	4.209	19.948	4.854	BnaC03g24120D protein	AN-M
MELO3C017764.2	13.707	18.633	12.198	14.066	7.476	72.767	4.522	Transmembrane protein, putative	AN-M
MELO3C017783.2	6.880	9.783	4.419	9.424	7.004	21.044	6.529	GEM-like protein 1	AN-M
MELO3C017788.2	38.991	41.660	12.534	36.038	25.729	112.591	23.299	Peroxisomal membrane protein 13	AN-M
MELO3C017790.2	3.105	3.796	2.266	3.495	2.043	9.618	2.445	Plant regulator RWP-RK family protein	AN-M
MELO3C017798.2	1.858	2.036	7.172	2.157	2.375	4.682	2.942	Hydroxyacylglutathione hydrolase	AN-M
MELO3C017799.2	1.527	1.943	4.917	1.650	1.933	5.206	1.436	F2P16.20 protein, putative isoform 1	AN-M
MELO3C017852.2	1.037	0.364	0.187	1.093	1.059	11.931	0.440	UPF0481 plant-like protein	AN-M
MELO3C017890.2	3.771	5.259	2.270	6.066	2.120	31.559	2.738	DCC family protein At1g52590, chloroplastic	AN-M
MELO3C017953.2	3.000	2.701	1.429	3.709	2.206	42.144	2.386	Magnesium/proton exchanger, putative	AN-M
MELO3C018024.2	6.478	5.515	5.461	8.332	4.561	16.996	7.010	F-box protein SKIP22	AN-M
MELO3C018026.2	2.199	2.033	1.580	1.706	1.987	11.192	1.780	Tubulin alpha chain	AN-M
MELO3C018031.2	10.393	6.693	2.842	11.888	10.922	61.461	8.293	DUF1005 family protein (DUF1005)	AN-M

Gene ID	FPKM							Gene Description	Specific in episode
	FS	GI-M	GM-M	AN-M	GI-H	GM-H	AN-H		
MELO3C018040.2	6.680	6.367	2.405	8.005	4.367	30.454	5.642	Major facilitator superfamily	AN-M
MELO3C006996.2	3.377	3.059	3.156	3.398	2.610	10.174	4.004	Ycf3-interacting protein 1, chloroplastic	AN-M
MELO3C006997.2	8.739	6.376	2.372	10.097	6.961	27.893	8.190	galactan beta-1,4-galactosyltransferase GALS3-like	AN-M
MELO3C007058.2	4.385	2.951	3.431	4.944	5.002	10.852	3.357	Fatty acid 2-hydroxylase	AN-M
MELO3C007074.2	1.343	2.917	1.402	3.020	1.212	10.619	1.318	Transmembrane protein, putative	AN-M
MELO3C007219.2	5.606	7.697	2.090	5.553	9.230	118.414	9.731	Protein trichome birefringence	AN-M
MELO3C007242.2	1.775	1.595	1.075	0.956	NA	9.340	1.122	Ethylene-responsive transcription factor, putative	AN-M
MELO3C007253.2	6.927	14.107	4.827	9.759	3.187	35.813	1.982	Glutaredoxin	AN-M
MELO3C007260.2	14.239	12.112	11.765	11.565	10.106	57.792	11.854	homeobox-DDT domain protein RLT1 isoform X1	AN-M
MELO3C007265.2	13.009	12.822	12.306	11.812	10.036	45.018	10.873	Protein DEHYDRATION-INDUCED 19	AN-M
MELO3C007285.2	3.561	3.066	3.216	2.738	1.527	22.311	1.825	E3 ubiquitin-protein ligase RMA1H1-like	AN-M
MELO3C007312.2	17.920	16.094	8.096	23.013	10.798	81.645	9.422	VPS28-2: Vacuolar protein sorting-associated protein 28 2	AN-M
MELO3C007320.2	24.670	25.927	12.100	30.299	26.350	68.032	24.411	Secretory carrier-associated membrane protein	AN-M
MELO3C007340.2	1.164	1.617	1.784	0.807	2.450	6.815	2.987	NAD(P)H dehydrogenase (Quinone) FQR1-like	AN-M
MELO3C007350.2	3.957	2.526	4.981	5.665	2.612	16.608	2.887	Transmembrane protein, putative (DUF1218)	AN-M
MELO3C007393.2	1.623	2.553	4.566	2.733	2.591	6.378	1.881	Autophagy-related protein	AN-M
MELO3C007413.2	2.032	1.667	3.390	2.155	2.129	7.198	2.145	Core-2/I-branching beta-1,6-N-acetylglucosaminyltransferase family protein	AN-M
MELO3C007414.2	3.324	2.445	1.834	3.193	2.785	8.056	3.364	Leucine--tRNA ligase	AN-M
MELO3C007432.2	1.333	2.196	3.841	1.895	1.399	6.329	1.398	Phosphatidate cytidylyltransferase	AN-M
MELO3C007461.2	5.408	5.589	4.128	5.019	4.145	11.627	4.590	Spatacsin carboxy-terminus protein	AN-M
MELO3C007482.2	1.147	2.010	4.998	1.617	2.268	109.149	1.090	Cytochrome P450 family protein	AN-M
MELO3C007506.2	2.127	2.265	1.262	2.745	0.945	6.304	1.508	CASP-like protein	AN-M
MELO3C007555.2	5.412	6.422	3.690	5.637	4.844	24.978	5.672	Ran-binding protein in the microtubule-organising centre protein	AN-M
MELO3C007655.2	2.650	4.153	1.473	1.870	1.523	9.287	1.534	BZIP transcription factor	AN-M
MELO3C007668.2	26.150	21.605	15.627	33.597	23.081	76.641	31.218	Cysteine desulfurase-like protein	AN-M
MELO3C007689.2	3.072	3.068	2.915	2.368	2.650	11.257	2.608	protein RMD5 homolog A	AN-M
MELO3C007712.2	10.597	18.579	6.880	11.621	9.088	49.007	10.584	nicotinamidase 1	AN-M
MELO3C007713.2	0.745	1.225	5.854	1.412	1.673	3.845	0.808	DNA ligase	AN-M
MELO3C007728.2	2.497	2.246	2.131	2.515	1.520	10.680	1.560	GLABRA2 expression modulator	AN-M
MELO3C007729.2	32.026	39.942	10.439	23.764	20.509	132.982	8.740	Unknown protein	AN-M

Gene ID	FPKM							Gene Description	Specific in episode
	FS	GI-M	GM-M	AN-M	GI-H	GM-H	AN-H		
MELO3C007738.2	3.957	3.915	3.535	4.753	3.044	11.583	4.144	LMBR1-like membrane protein	AN-M
MELO3C007789.2	5.838	5.051	2.042	8.619	3.270	18.286	3.880	gamma-glutamyltranspeptidase 1-like	AN-M
MELO3C007885.2	14.666	14.324	8.186	18.424	13.774	67.970	12.820	UPF0183 protein	AN-M
MELO3C007897.2	2.150	2.636	5.277	2.796	2.088	6.490	3.265	2OG-Fe(II) oxygenase family oxidoreductase	AN-M
MELO3C007921.2	0.474	0.984	3.012	0.645	0.741	2.020	0.902	At4g35980	AN-M
MELO3C007972.2	8.363	9.201	9.808	9.315	6.218	30.924	6.632	Kinase family protein	AN-M
MELO3C007984.2	2.927	2.570	1.421	4.491	1.790	14.202	2.280	Thioredoxin reductase	AN-M
MELO3C008016.2	4.219	4.798	5.855	3.961	3.193	10.111	3.606	serine/threonine-protein kinase/endoribonuclease IRE1a-like	AN-M
MELO3C008040.2	11.827	10.363	7.334	11.845	12.061	46.814	14.394	RING-type E3 ubiquitin transferase	AN-M
MELO3C008041.2	2.521	3.340	4.476	2.561	2.999	12.534	2.136	WD repeat-containing protein 44	AN-M
MELO3C008058.2	16.763	16.547	5.779	12.743	6.957	50.927	6.230	mitogen-activated protein kinase kinase kinase YODA isoform X1	AN-M
MELO3C024566.2	3.443	3.137	5.879	3.311	3.177	34.309	3.440	LETM1-like protein	AN-M
MELO3C024562.2	8.143	11.846	5.135	10.879	6.898	29.298	6.452	Vacuolar protein sorting 55 family protein	AN-M
MELO3C024538.2	29.129	27.144	14.027	50.767	17.211	114.568	16.838	zinc finger A20 and AN1 domain-containing stress-associated protein 4	AN-M
MELO3C024523.2	0.210	0.182	0.146	0.148	NA	1.787	0.168	ABC transporter G family member 39-like	AN-M
MELO3C024478.2	3.729	4.246	5.090	4.331	3.046	17.499	3.632	serine/threonine protein phosphatase 2A 57 kDa regulatory subunit B' beta isoform	AN-M
MELO3C020721.2	4.639	5.773	4.261	3.703	2.507	14.798	4.057	triacylglycerol lipase SDP1	AN-M
MELO3C033230.2	0.262	0.162	0.246	0.204	0.227	3.607	0.222	BZIP transcription factor	AN-M
MELO3C023166.2	0.311	0.682	0.357	0.694	0.286	2.473	0.188	C2 domain-containing family protein	AN-M
MELO3C009014.2	4.699	6.002	7.296	4.881	6.592	15.728	7.549	LOW QUALITY PROTEIN: cyclin-dependent kinase E-1	AN-M
MELO3C033070.2	11.460	13.503	5.100	12.109	6.881	33.023	18.928	MYB-domain protein	AN-M
MELO3C008983.2	15.517	11.670	7.069	15.894	10.308	33.565	10.589	cytosolic Fe-S cluster assembly factor NBP35	AN-M
MELO3C008979.2	10.106	9.367	4.463	12.972	6.331	30.356	6.567	GPI inositol-deacylase PGAP1-like protein	AN-M
MELO3C008946.2	4.097	4.543	1.843	3.484	3.487	10.094	2.736	alternative NAD(P)H-ubiquinone oxidoreductase C1, chloroplastic/mitochondrial	AN-M
MELO3C008871.2	2.875	2.220	1.320	2.601	1.832	7.545	1.547	Chaperone protein dnaJ, putative	AN-M
MELO3C008854.2	136.298	131.776	21.032	169.575	92.011	368.159	130.486	Cationic amino acid transporter	AN-M
MELO3C008851.2	3.504	4.263	2.967	2.865	2.549	15.348	3.188	Cationic amino acid transporter, putative	AN-M
MELO3C008813.2	4.036	3.939	2.879	3.773	2.727	8.674	4.380	C2 domain-containing protein	AN-M
MELO3C008793.2	9.756	7.475	3.178	6.994	5.271	19.852	5.335	aquaporin SIP1-1-like	AN-M

Gene ID	FPKM							Gene Description	Specific in episode
	FS	GI-M	GM-M	AN-M	GI-H	GM-H	AN-H		
MELO3C025260.2	3.520	3.154	2.207	3.983	2.518	16.231	2.895	RING finger and transmembrane domain-containing protein 2	AN-M
MELO3C025232.2	38.872	51.005	35.650	50.981	35.854	115.857	36.133	Ubiquitin-conjugating enzyme, E2	AN-M
MELO3C033425.2	1.507	2.784	2.124	2.823	2.004	6.585	1.143	Unknown protein	AN-M
MELO3C025230.2	2.293	1.705	4.274	1.326	2.465	4.993	1.982	Thioredoxin	AN-M
MELO3C025224.2	9.551	9.244	9.410	10.944	6.820	32.953	8.844	Phospholipid-transporting ATPase	AN-M
MELO3C026030.2	2.417	2.408	6.522	2.394	3.264	5.444	2.464	Protein phosphatase-2c, putative	AN-M
MELO3C033451.2	1.119	0.992	1.461	0.621	1.126	2.312	0.920	protein FAR1-RELATED SEQUENCE 11 isoform X1	AN-M
MELO3C014211.2	87.764	139.304	48.708	62.016	46.328	306.748	36.749	Protein EARLY RESPONSIVE TO DEHYDRATION 15	AN-M
MELO3C014214.2	0.786	0.527	0.708	0.618	0.595	2.590	0.661	ABC transporter G family-like protein	AN-M
MELO3C014220.2	1.072	0.717	0.772	1.146	0.345	2.589	0.800	Transmembrane protein, putative	AN-M
MELO3C014224.2	6.090	5.564	2.110	7.686	6.786	195.883	4.662	phenylalanine ammonia-lyase-like	AN-M
MELO3C014230.2	18.476	18.726	12.729	16.584	15.748	176.357	22.037	Ethylene insensitive 2	AN-M
MELO3C003088.2	1.124	1.494	1.664	0.617	0.751	4.822	0.325	Phosphatidate phosphatase PAH1	AN-M
MELO3C003139.2	5.778	6.485	12.435	4.897	5.798	21.049	5.535	ATP-dependent zinc metalloprotease FtsH	AN-M
MELO3C003164.2	0.555	0.623	0.093	0.348	NA	1.905	0.172	Cysteine/Histidine-rich C1 domain family protein, putative	AN-M
MELO3C003185.2	13.566	11.481	8.506	13.257	8.886	32.929	10.778	Proteasome inhibitor-related	AN-M
MELO3C003205.2	25.178	21.624	11.244	15.616	21.499	83.773	25.376	Mitochondrial pyruvate dehydrogenase kinase	AN-M
MELO3C003262.2	0.578	0.668	1.595	0.575	0.839	1.414	0.628	FRIGIDA-like protein	AN-M
MELO3C003277.2	6.357	6.527	6.960	7.929	5.863	26.131	4.977	4,5-dioxygenase-like protein	AN-M
MELO3C003286.2	6.851	6.937	5.574	7.217	6.394	14.694	7.406	Serine/threonine-protein kinase	AN-M
MELO3C022226.2	45.253	38.165	14.896	38.536	52.941	345.889	63.565	patellin-3-like	AN-M
MELO3C022178.2	5.900	7.944	6.806	6.717	4.632	23.310	4.863	Protein phosphatase methylesterase 1	AN-M
MELO3C022106.2	0.234	0.590	0.400	0.582	NA	3.615	0.055	thioredoxin H-type	AN-M
MELO3C022069.2	2.388	2.637	0.872	2.987	2.790	6.298	2.840	Endo-1,3(4)-beta-glucanase 1	AN-M
MELO3C022055.2	10.621	6.978	5.356	8.210	10.878	34.081	10.071	F-box/kelch-repeat protein At5g15710	AN-M
MELO3C022048.2	14.292	13.254	3.092	18.365	10.944	55.521	10.906	Regulator of chromosome condensation (RCC1) family protein	AN-M
MELO3C022043.2	2.641	2.689	1.833	2.764	2.982	8.770	3.093	Mitochondrial carrier protein	AN-M
MELO3C022014.2	2.289	2.314	3.603	2.284	1.409	6.168	2.369	WRKY transcription factor, putative	AN-M
MELO3C022008.2	9.785	11.099	4.581	13.128	8.886	41.336	11.423	Pectinesterase	AN-M
MELO3C021484.2	10.911	11.611	3.816	12.348	7.903	39.365	9.567	Methyltransferase	AN-M
MELO3C033554.2	0.289	0.434	1.787	0.556	0.307	1.353	0.312	BnaA02g25840D protein	AN-M

Gene ID	FPKM							Gene Description	Specific in episode
	FS	GI-M	GM-M	AN-M	GI-H	GM-H	AN-H		
MELO3C021534.2	7.256	7.334	2.642	6.606	3.229	25.683	5.462	homeobox-leucine zipper protein HAT5-like	AN-M
MELO3C021603.2	2.302	3.206	4.987	3.468	2.826	10.000	1.610	alpha/beta hydrolase domain-containing protein 17B-like	AN-M
MELO3C021612.2	3.829	5.666	4.694	3.040	4.340	13.495	3.417	Cystinosin like	AN-M
MELO3C021629.2	3.527	6.978	6.992	2.571	1.441	15.441	1.541	Signal transduction histidine-protein kinase ArlS	AN-M
MELO3C021630.2	1.627	1.765	3.549	2.244	2.169	22.676	1.646	Microneme/rhoptry antigen	AN-M
MELO3C025479.2	0.508	1.140	3.846	0.614	1.126	2.298	1.009	Phosphatidylinositol N-acetyglucosaminlytransferase subunit P-like protein	AN-M
MELO3C025491.2	1.167	1.326	5.250	0.942	1.294	3.082	1.315	transcription initiation factor TFIID subunit 1-like	AN-M
MELO3C003037.2	0.287	0.279	0.112	0.669	NA	2.143	0.469	Pathogenesis-related thaumatin-like protein	AN-M
MELO3C022772.2	7.723	9.329	2.990	10.413	2.966	184.405	3.333	Nitrate reductase	AN-M
MELO3C022799.2	0.995	1.209	2.877	0.559	1.095	2.890	0.888	sulfate transporter 1.3-like	AN-M
MELO3C022845.2	0.601	0.606	1.369	1.114	0.463	2.310	0.332	RING-type E3 ubiquitin transferase	AN-M
MELO3C025111.2	7.446	8.728	2.345	10.941	1.869	285.976	2.156	CoA ligase	AN-M
MELO3C025105.2	0.442	0.385	0.162	0.492	0.266	88.122	0.239	CoA ligase	AN-M
MELO3C005068.2	5.095	5.338	2.671	7.521	3.356	19.494	4.164	transmembrane protein 53	AN-M
MELO3C005116.2	0.815	1.147	1.661	0.326	0.970	3.976	0.970	Glycerol-3-phosphate dehydrogenase [NAD(+)]	AN-M
MELO3C005121.2	0.222	0.415	1.210	0.252	0.377	1.163	0.321	ADP-ribosylation factor GTPase-activating protein AGD12-like	AN-M
MELO3C005169.2	0.488	0.362	0.223	0.176	0.334	4.136	0.167	protein STRICTOSIDINE SYNTHASE-LIKE 10-like	AN-M
MELO3C005185.2	1.876	1.758	1.958	1.945	2.121	4.640	1.945	O-linked n-acetylglucosamine transferase, ogt, putative	AN-M
MELO3C005224.2	2.113	2.029	0.992	0.608	5.616	44.322	1.191	Glutamate decarboxylase	AN-M
MELO3C005241.2	4.817	5.040	15.436	5.599	5.218	11.382	6.870	65-kDa microtubule-associated protein 6	AN-M
MELO3C005245.2	15.786	12.365	6.433	14.549	19.205	62.687	14.799	Xyloglucan endotransglucosylase/hydrolase	AN-M
MELO3C005281.2	3.155	3.062	2.442	3.493	2.698	7.196	2.928	plant UBX domain-containing protein 2	AN-M
MELO3C005288.2	4.402	6.123	1.697	3.947	2.491	22.460	4.403	F-box family protein	AN-M
MELO3C005303.2	6.757	4.825	2.065	5.817	7.266	14.711	5.902	BnaC06g32050D protein	AN-M
MELO3C005309.2	6.490	5.497	2.967	7.030	5.206	100.525	5.311	RING/FYVE/PHD zinc finger protein	AN-M
MELO3C005310.2	3.432	4.897	5.911	6.377	7.786	19.265	13.940	Ribose-5-phosphate isomerase A	AN-M
MELO3C005338.2	0.880	1.162	2.544	0.824	0.777	2.389	1.174	Alpha/beta fold hydrolase	AN-M
MELO3C005343.2	2.175	1.249	1.965	2.777	NA	9.879	2.391	50S ribosomal protein-related, putative	AN-M
MELO3C005346.2	2.558	4.016	5.967	3.464	6.345	9.423	3.841	Chaperone protein dnaJ 15	AN-M
MELO3C005386.2	7.221	7.319	9.689	7.207	5.834	18.101	7.623	serine/threonine-protein kinase minibrain isoform X2	AN-M

Gene ID	FPKM							Gene Description	Specific in episode
	FS	GI-M	GM-M	AN-M	GI-H	GM-H	AN-H		
MELO3C005388.2	9.446	6.973	4.856	10.385	7.215	21.959	6.745	DNA-binding SMUBP-2	AN-M
MELO3C005405.2	6.230	7.863	2.275	8.409	4.885	28.859	5.267	CDGSH iron-sulfur domain-containing protein NEET	AN-M
MELO3C005410.2	2.325	2.565	1.647	2.052	2.010	14.203	3.415	Hydrolase, putative	AN-M
MELO3C005447.2	1.966	2.194	5.131	2.456	2.020	5.632	1.453	SWR1 complex subunit 2	AN-M
MELO3C005493.2	1.596	1.976	2.917	1.903	1.272	7.956	1.660	acid phosphatase 1	AN-M
MELO3C005516.2	0.951	0.737	0.893	1.550	NA	6.870	0.304	Exostosin family protein	AN-M
MELO3C005565.2	8.254	5.511	5.905	5.676	6.596	23.744	12.196	Elongation factor G, chloroplastic	AN-M
MELO3C005573.2	0.288	0.116	0.173	0.357	0.607	1.549	0.261	flavonoid 3',5'-hydroxylase 1-like	AN-M
MELO3C005624.2	3.402	4.484	4.768	4.382	3.097	10.213	2.953	SPX domain-containing membrane protein	AN-M
MELO3C005627.2	8.459	10.837	5.214	10.134	6.612	40.828	7.280	UDP-galactose/UDP-glucose transporter 2	AN-M
MELO3C005637.2	6.591	12.253	5.899	5.607	2.990	73.562	2.057	Rhomboid-like protein	AN-M
MELO3C005672.2	3.110	4.887	7.869	4.094	5.564	9.907	4.429	Receptor protein kinase, putative	AN-M
MELO3C005688.2	42.958	55.719	43.247	46.378	48.467	139.034	52.761	LOW QUALITY PROTEIN: puromycin-sensitive aminopeptidase	AN-M
MELO3C005691.2	8.138	13.751	11.345	10.007	6.014	28.915	6.790	Ubiquitin-conjugating enzyme, E2	AN-M
MELO3C005692.2	0.809	0.884	2.559	0.970	0.302	2.721	0.753	E3 ubiquitin-protein ligase At3g02290-like	AN-M
MELO3C005727.2	16.232	17.919	9.328	27.134	10.128	55.839	13.992	F22C12.10	AN-M
MELO3C005736.2	2.033	2.440	3.252	0.602	NA	7.571	2.002	Cytoplasmic tRNA 2-thiolation protein	AN-M
MELO3C005738.2	4.924	6.488	9.651	4.955	5.051	28.928	6.099	protein SPA1-RELATED 2	AN-M
MELO3C005773.2	4.436	5.093	4.878	5.621	3.377	12.095	5.976	WD repeat-containing protein 44	AN-M
MELO3C005783.2	0.916	1.709	1.334	1.433	1.783	3.620	2.711	metal-nicotianamine transporter YSL1	AN-M
MELO3C005814.2	19.578	16.317	7.472	24.164	14.109	49.414	14.788	transmembrane protein 120 homolog	AN-M
MELO3C005862.2	2.785	3.652	3.120	3.950	2.079	10.833	3.859	Proline-rich cell wall protein-like	AN-M
MELO3C005868.2	11.646	9.338	2.535	13.123	7.257	41.008	7.193	mitochondrial carrier protein MTM1	AN-M
MELO3C005890.2	2.138	2.884	5.504	3.325	5.109	9.090	4.559	Alpha/beta fold hydrolase	AN-M
MELO3C005914.2	2.568	2.130	2.449	3.546	2.847	9.754	1.944	CDPK-related kinase 6	AN-M
MELO3C005921.2	4.606	6.390	6.418	3.427	2.889	16.326	2.829	two-component response regulator-like APRR5	AN-M
MELO3C012494.2	3.194	4.893	3.324	3.629	3.497	10.827	1.911	Proline-rich family protein	AN-M
MELO3C012476.2	1.774	2.051	0.345	1.813	NA	8.516	1.195	NADPH:quinone oxidoreductase-like	AN-M
MELO3C012403.2	0.892	1.339	1.066	0.908	0.627	29.910	0.969	cysteine proteinase 15A	AN-M
MELO3C012395.2	0.759	0.869	0.096	0.838	0.899	1.922	0.942	Protein nuclear fusion defective 4	AN-M
MELO3C012392.2	0.454	0.649	0.470	0.755	0.829	3.312	0.794	monocopper oxidase-like protein SKU5	AN-M

Gene ID	FPKM							Gene Description	Specific in episode
	FS	GI-M	GM-M	AN-M	GI-H	GM-H	AN-H		
MELO3C012339.2	2.431	3.606	2.331	3.498	1.709	20.139	0.802	Nuclear transport factor 2 (NTF2) family protein	AN-M
MELO3C012338.2	2.378	3.220	8.444	3.009	3.963	7.590	3.259	exocyst complex component EXO70A1	AN-M
MELO3C012334.2	0.696	1.012	1.920	0.894	0.799	2.052	0.675	Apoptosis-inducing factor-like protein A	AN-M
MELO3C012329.2	1.390	2.587	2.248	1.774	1.088	5.201	1.364	EEIG1/EHBP1 protein amino-terminal domain protein	AN-M
MELO3C012303.2	1.785	2.965	5.550	2.906	2.861	6.177	1.844	Trichome birefringence-like protein	AN-M
MELO3C012288.2	9.442	12.660	4.211	13.007	6.855	28.296	8.772	sec-independent protein translocase protein TATC, chloroplastic	AN-M
MELO3C012281.2	7.667	9.155	2.694	8.038	8.750	22.446	7.022	amino-acid permease BAT1 homolog	AN-M
MELO3C012265.2	0.921	0.844	1.312	0.965	1.960	3.810	1.197	Zinc finger protein, putative	AN-M
MELO3C012256.2	15.116	11.828	9.954	18.443	7.663	92.332	6.918	Transmembrane protein, putative	AN-M
MELO3C012239.2	35.463	31.145	16.004	37.634	23.366	172.183	22.480	Bowman-Birk type bran trypsin inhibitor	AN-M
MELO3C012238.2	17.025	14.080	7.939	15.871	12.704	47.629	16.285	ubiquitin receptor RAD23c-like	AN-M
MELO3C012236.2	0.952	1.042	0.681	1.183	0.323	7.889	0.417	accelerated cell death 11	AN-M
MELO3C012228.2	14.330	10.934	3.332	9.608	2.942	33.771	1.900	Alpha/beta hydrolase-3	AN-M
MELO3C012209.2	1.697	2.114	3.931	2.393	1.473	92.767	1.447	Protein CHUP1, chloroplastic	AN-M
MELO3C012185.2	0.991	1.807	4.225	1.984	2.367	5.341	1.521	F-box/FBD/LRR-repeat protein At1g13570	AN-M
MELO3C012183.2	0.152	0.167	0.179	0.508	NA	1.186	0.112	Peroxidase	AN-M
MELO3C012154.2	0.300	0.097	0.093	0.698	NA	6.144	NA	proline-rich receptor-like protein kinase PERK14	AN-M
MELO3C012152.2	1.568	1.726	2.702	0.945	2.231	3.844	1.953	ureide permease 1-like	AN-M
MELO3C012095.2	7.552	6.593	2.126	8.708	4.637	46.646	3.979	RING/FYVE/PHD zinc finger superfamily protein	AN-M
MELO3C012088.2	15.448	12.809	4.283	13.203	12.092	41.899	12.684	sphinganine C4-monooxygenase 2	AN-M
MELO3C012042.2	15.253	14.371	12.277	14.872	16.977	46.618	26.076	Serine/threonine-protein phosphatase	AN-M
MELO3C012041.2	0.921	1.204	2.043	1.148	1.663	4.683	1.777	Mediator of DNA damage checkpoint protein 1	AN-M
MELO3C012035.2	0.889	0.375	0.695	0.970	0.964	3.273	0.711	Pyruvate dehydrogenase E1 component subunit alpha	AN-M
MELO3C012034.2	8.986	7.823	7.274	15.227	6.821	36.762	3.909	Hexosyltransferase	AN-M
MELO3C012032.2	0.794	1.038	1.017	0.535	1.052	2.194	1.076	Formin-like protein	AN-M
MELO3C012004.2	0.458	1.029	2.260	0.443	0.423	12.190	0.457	Xyloglucan endotransglucosylase/hydrolase	AN-M
MELO3C011995.2	0.620	0.343	0.122	0.268	NA	6.378	0.325	Protein kinase family protein	AN-M
MELO3C011984.2	6.319	7.306	1.795	7.993	4.894	47.568	4.681	pyrrolidone-carboxylate peptidase	AN-M
MELO3C011944.2	21.830	27.164	10.728	20.366	15.180	76.154	16.950	Beta-1,4-mannosyl-glycoprotein 4-beta-N-acetylglucosaminyltransferase	AN-M
MELO3C011897.2	16.233	18.670	8.791	22.131	12.195	51.891	14.803	F-box protein SKIP14	AN-M

Gene ID	FPKM							Gene Description	Specific in episode
	FS	GI-M	GM-M	AN-M	GI-H	GM-H	AN-H		
MELO3C011868.2	70.597	104.891	35.675	98.331	46.034	281.195	47.818	Rubber elongation factor protein (REF)	AN-M
MELO3C011862.2	1.649	1.833	1.458	1.626	1.848	4.063	1.392	Phosphoribosylformylglycinamidine synthase	AN-M
MELO3C011825.2	6.673	6.903	3.540	5.495	4.938	29.977	5.864	nifU-like protein 2, chloroplastic	AN-M
MELO3C011795.2	6.500	4.547	2.880	7.036	3.469	14.916	4.961	protein ELC-like	AN-M
MELO3C011777.2	1.052	1.119	2.594	1.371	0.750	4.406	1.138	F-box/LRR-repeat protein At4g29420	AN-M
MELO3C011776.2	22.958	22.227	8.646	23.194	21.671	53.368	24.971	Sphingoid long-chain bases kinase 1	AN-M
MELO3C011754.2	9.952	7.689	5.140	9.267	5.444	31.273	9.398	transcription factor TCP7-like	AN-M
MELO3C023847.2	15.848	17.105	8.566	10.755	4.500	49.251	3.296	glucan endo-1,3-beta-glucosidase	AN-M
MELO3C023850.2	5.308	7.231	5.192	7.307	9.983	14.760	7.584	dolichyl-phosphate beta-glucosyltransferase-like	AN-M
MELO3C023875.2	8.444	8.415	3.003	8.839	3.980	19.111	3.931	E3 ubiquitin-protein ligase RING1-like	AN-M
MELO3C026573.2	18.394	19.467	8.817	23.377	27.496	58.737	22.541	Proline transporter	AN-M
MELO3C025166.2	6.320	4.120	2.200	7.853	4.732	66.547	5.063	Aquaporin	AN-M
MELO3C025175.2	0.741	1.202	1.841	0.907	0.278	2.906	0.314	Protein yippee-like	AN-M
MELO3C020113.2	4.864	4.356	2.106	5.431	3.571	11.969	3.773	ADP,ATP carrier protein	AN-M
MELO3C020140.2	4.563	3.398	1.860	2.557	4.942	11.287	4.111	tRNA (guanine(26)-N(2))-dimethyltransferase	AN-M
MELO3C020142.2	2.221	1.928	0.997	2.842	1.232	5.802	1.304	Appr-1-P processing enzyme family protein	AN-M
MELO3C018353.2	25.205	17.593	7.089	30.374	12.083	134.095	15.020	Auxin efflux carrier component	AN-M
MELO3C034452.2	0.300	0.262	0.357	0.342	0.254	1.037	0.323	Alpha/beta-Hydrolases superfamily protein	AN-M
MELO3C018333.2	1.871	2.060	0.811	2.353	1.423	7.486	1.391	Six-bladed beta-propeller, TolB-like protein	AN-M
MELO3C022909.2	11.925	10.461	4.329	14.170	4.775	34.636	6.491	Auxin-responsive protein-like protein	AN-M
MELO3C026475.2	0.885	0.875	1.619	1.104	0.862	4.846	0.803	Protein ABC transporter 1, mitochondrial	AN-M
MELO3C026691.2	0.929	1.418	2.243	1.023	0.954	3.574	0.912	Paramyosin	AN-M
MELO3C026686.2	0.541	0.654	0.490	0.627	0.331	1.783	0.448	U2 small nuclear ribonucleoprotein a	AN-M
MELO3C023239.2	1.333	1.398	1.672	1.235	0.932	7.904	1.204	GDP-mannose transporter	AN-M
MELO3C023258.2	0.311	0.427	0.585	0.535	0.436	1.969	0.627	RING-type E3 ubiquitin transferase	AN-M
MELO3C023268.2	0.329	0.436	0.978	0.327	0.464	2.975	0.318	Helicase required for RNAi-mediated heterochromatin assembly 1	AN-M
MELO3C023288.2	0.069	0.077	0.200	0.134	0.033	19.902	0.100	terpene synthase 10-like isoform X1	AN-M
MELO3C023333.2	7.771	7.335	12.914	6.564	6.460	17.125	9.471	BEL1-like homeodomain protein 7	AN-M
MELO3C020910.2	0.943	1.344	1.676	1.492	0.358	13.887	0.515	Glycosyl hydrolase family 43 protein	AN-M
MELO3C020877.2	1.447	1.164	1.718	1.198	1.205	13.913	3.594	Membrane insertase, putative (DUF1685)	AN-M

Gene ID	FPKM							Gene Description	Specific in episode
	FS	GI-M	GM-M	AN-M	GI-H	GM-H	AN-H		
MELO3C020865.2	23.845	28.194	8.291	29.326	17.943	83.901	23.606	Sigma factor sigB regulation protein rsbQ	AN-M
MELO3C020863.2	2.383	2.874	1.842	2.260	0.932	6.280	2.192	DUF1230 family protein	AN-M
MELO3C020856.2	4.206	7.527	1.476	2.799	2.078	22.609	1.199	F-box family protein	AN-M
MELO3C020829.2	16.661	16.503	6.731	17.543	14.986	41.816	16.493	Phosphatase 2C family protein	AN-M
MELO3C020817.2	1.380	2.066	7.070	1.474	5.356	8.502	5.556	serine/threonine-protein kinase At3g07070	AN-M
MELO3C020812.2	2.548	2.493	4.804	5.210	1.157	14.210	1.377	MYB transcription factor	AN-M
MELO3C020786.2	25.320	33.364	18.808	35.767	23.604	75.552	25.230	eukaryotic translation initiation factor 1A	AN-M
MELO3C021865.2	8.373	5.788	5.504	9.473	9.025	25.178	7.335	Cinnamoyl-CoA reductase, putative	AN-M
MELO3C021823.2	1.244	0.947	3.548	1.990	0.474	12.406	0.765	Pleiotropic drug resistance ABC transporter	AN-M
MELO3C024757.2	0.083	0.101	0.229	0.076	0.014	2.658	NA	septin and tuftelin-interacting protein 1 homolog 1	AN-M
MELO3C019225.2	0.765	0.477	0.837	0.491	0.405	1.971	0.300	Formin-like protein	AN-M
MELO3C019256.2	2.475	3.018	6.112	1.068	2.446	10.638	2.629	Glutamate receptor	AN-M
MELO3C019271.2	5.061	7.771	7.325	4.032	3.778	18.900	5.906	Autophagy-related protein	AN-M
MELO3C019278.2	5.511	7.756	2.829	8.110	3.162	31.583	2.044	tetraspanin-8-like	AN-M
MELO3C034694.2	2.003	1.702	0.286	2.659	2.101	14.347	2.173	vacuolar cation/proton exchanger 3	AN-M
MELO3C019301.2	2.098	2.645	2.793	2.841	4.140	9.452	3.306	Core-2/I-branching beta-1,6-N-acetylglucosaminyltransferase family protein	AN-M
MELO3C019302.2	1.740	1.831	2.109	1.590	1.456	6.926	0.970	Acid phosphatase/vanadium-dependent haloperoxidase-related protein	AN-M
MELO3C019359.2	6.546	4.796	3.839	7.602	6.345	25.333	8.928	TBC1 domain family member 2A	AN-M
MELO3C025977.2	1.477	1.859	0.525	1.449	2.203	4.770	1.401	OBERON-like protein	AN-M
MELO3C025951.2	2.338	2.150	1.283	2.039	0.756	6.885	1.331	nuclear transcription factor Y subunit B-3	AN-M
MELO3C013480.2	12.323	10.068	3.769	15.648	10.414	44.075	9.589	3-hydroxy-3-methylglutaryl coenzyme A reductase	AN-M
MELO3C013588.2	2.877	3.516	4.570	3.616	4.395	15.525	3.911	Transmembrane E3 ubiquitin-protein ligase 1	AN-M
MELO3C013618.2	1.672	0.977	1.040	2.608	0.333	19.269	0.709	Phospho-N-acetylmuramoyl-pentapeptide-transferase, putative	AN-M
MELO3C013619.2	4.401	3.636	2.937	2.526	3.624	9.859	3.661	Mitochondrial glycoprotein	AN-M
MELO3C013634.2	2.657	3.141	4.274	1.880	1.593	6.528	2.453	DNA-directed RNA polymerase subunit beta	AN-M
MELO3C013665.2	8.503	6.502	8.565	10.555	6.668	35.242	6.396	mechanosensitive ion channel protein 6-like	AN-M
MELO3C013694.2	4.715	4.750	4.291	4.236	3.519	19.346	4.341	RING-type E3 ubiquitin transferase	AN-M
MELO3C025917.2	6.268	5.746	2.720	6.483	2.556	16.262	2.619	CAX-interacting protein 4	AN-M
MELO3C025844.2	18.688	20.624	9.156	20.110	16.404	99.354	19.983	zinc finger CCCH domain-containing protein 66	AN-M

Gene ID	FPKM							Gene Description	Specific in episode
	FS	GI-M	GM-M	AN-M	GI-H	GM-H	AN-H		
MELO3C026835.2	1.934	2.622	1.992	2.305	2.472	8.018	3.433	Pentatricopeptide repeat-containing protein At2g30100, chloroplastic	AN-M
MELO3C001616.2	39.046	29.627	3.973	41.166	33.955	222.431	29.420	U-box domain-containing protein 7	AN-M
MELO3C019643.2	2.761	2.585	4.018	2.056	2.241	6.535	2.582	Filamentous hemagglutinin transporter	AN-M
MELO3C019637.2	6.518	7.290	14.299	7.042	7.954	21.877	9.962	LOW QUALITY PROTEIN: uncharacterized protein LOC103496289	AN-M
MELO3C019589.2	43.183	44.149	21.965	40.816	32.142	123.515	35.055	Ubiquitin-conjugating enzyme, E2	AN-M
MELO3C025725.2	6.752	7.243	11.033	5.331	6.971	15.651	6.372	RNA binding (RRM/RBD/RNP motifs) family protein	AN-M
MELO3C025771.2	2.206	1.304	2.267	2.586	2.110	8.760	3.368	Hydroxyproline-rich glycoprotein	AN-M
MELO3C025772.2	178.399	192.440	42.858	227.706	123.214	843.756	152.153	Aquaporin PIP2	AN-M
MELO3C025775.2	3.919	4.653	1.995	2.573	2.668	10.706	3.220	CASP-like protein	AN-M
MELO3C025786.2	45.086	32.017	17.972	39.516	21.804	103.301	28.356	phenylalanine ammonia-lyase-like	AN-M
MELO3C026959.2	1.545	1.144	1.884	1.016	0.862	6.426	1.454	Acyl-CoA N-acyltransferase with RING/FYVE/PHD-type zinc finger domain, putative	AN-M
MELO3C021471.2	23.032	19.567	5.632	24.104	16.315	68.331	19.181	aspartic proteinase-like protein 2	AN-M
MELO3C021464.2	4.418	3.938	1.958	5.133	3.098	14.454	3.764	Protein RETICULATA, chloroplastic	AN-M
MELO3C021400.2	14.172	14.316	7.229	14.426	11.975	32.299	12.378	DUF21 domain-containing protein	AN-M
MELO3C021398.2	12.315	13.715	2.797	12.225	8.002	44.191	7.469	serine incorporator 3	AN-M
MELO3C021392.2	3.004	2.932	2.768	3.678	2.183	8.137	2.472	Alpha/beta-Hydrolases superfamily protein	AN-M
MELO3C021350.2	2.523	3.606	1.130	2.080	2.662	10.410	1.649	monothiol glutaredoxin-S2-like	AN-M
MELO3C021331.2	18.766	17.589	6.270	14.916	13.162	111.696	6.170	Rapid alkalinization factor	AN-M
MELO3C021305.2	4.874	5.267	3.029	5.861	2.921	18.956	3.420	ATP-dependent RNA helicase A-like	AN-M
MELO3C021242.2	6.369	6.525	13.302	4.481	4.795	16.473	3.872	YTH domain family protein 2	AN-M
MELO3C021219.2	6.674	7.435	6.862	8.422	5.967	18.239	6.335	C2 domain-containing family protein	AN-M
MELO3C021214.2	5.145	4.997	3.616	4.228	4.622	12.258	5.455	Beta-amylase	AN-M
MELO3C021198.2	4.072	4.351	3.013	3.869	3.171	14.707	2.948	Transmembrane protein 8B	AN-M
MELO3C021171.2	0.946	1.282	1.570	0.799	0.814	4.974	0.634	BAG family molecular chaperone regulator 6	AN-M
MELO3C021163.2	2.212	2.117	3.349	3.455	2.513	19.537	1.932	Xyloglucan galactosyltransferase KATAMARI1	AN-M
MELO3C021156.2	0.199	0.562	0.528	0.579	0.364	3.038	0.693	crocetin glucosyltransferase, chloroplastic-like	AN-M
MELO3C021152.2	1.856	1.931	2.721	2.351	2.641	6.210	2.724	At3g60680	AN-M
MELO3C021147.2	7.391	6.471	2.969	13.422	4.295	34.007	2.683	14 kDa proline-rich protein DC2.15, putative	AN-M
MELO3C022308.2	1.569	1.855	3.759	1.423	1.675	4.752	1.276	Beclin 1 protein	AN-M
MELO3C022321.2	2.561	3.678	6.414	3.555	1.687	9.848	1.326	sphingosine-1-phosphate lyase	AN-M

Gene ID	FPKM							Gene Description	Specific in episode
	FS	GI-M	GM-M	AN-M	GI-H	GM-H	AN-H		
MELO3C022326.2	16.186	14.860	10.989	12.869	18.755	33.008	21.406	F12F1.11	AN-M
MELO3C022346.2	11.147	11.399	6.527	14.315	9.108	32.274	9.052	LITAF-domain-containing protein	AN-M
MELO3C022356.2	7.416	5.816	2.532	9.367	5.525	26.442	5.944	Trihelix transcription factor ASR3	AN-M
MELO3C022358.2	1.629	3.054	4.876	3.547	NA	27.513	NA	ethylene-responsive transcription factor ERF113-like	AN-M
MELO3C022369.2	7.300	7.207	9.220	7.844	7.449	18.091	8.774	eukaryotic peptide chain release factor subunit 1-3-like	AN-M
MELO3C022406.2	18.537	16.050	12.940	16.512	12.455	48.958	10.675	phosphatidylinositol 4-kinase gamma 4	AN-M
MELO3C022407.2	3.812	3.879	4.065	4.483	3.372	10.016	2.923	E3 ubiquitin-protein ligase	AN-M
MELO3C022408.2	1.008	1.442	4.242	1.509	1.410	4.980	0.818	UV-stimulated scaffold protein A homolog	AN-M
MELO3C022460.2	0.535	1.143	0.690	1.400	NA	3.124	0.759	Purine permease family protein	AN-M
MELO3C022528.2	0.580	0.506	1.057	0.508	0.753	3.158	0.449	AP2-like ethylene-responsive transcription factor SNZ	AN-M
MELO3C022535.2	0.933	0.814	1.827	0.897	0.717	2.228	0.581	Methionine adenosyltransferase 2 subunit beta	AN-M
MELO3C020516.2	0.905	0.488	0.637	1.118	0.264	3.292	0.152	Cation/H(+) antiporter	AN-M
MELO3C020532.2	3.510	2.822	2.589	5.000	2.572	22.254	2.392	Potassium transporter	AN-M
MELO3C020551.2	16.993	17.556	15.638	14.987	18.705	35.785	17.496	NudC domain-containing protein 2	AN-M
MELO3C020567.2	0.866	1.091	0.881	0.925	1.045	2.193	1.212	beta-glucosidase BoGH3B-like	AN-M
MELO3C020575.2	26.960	32.362	12.606	32.624	17.658	98.612	17.525	serine/arginine-rich splicing factor SR45a-like	AN-M
MELO3C035274.2	3.565	4.531	27.628	2.297	3.067	10.204	1.350	17.5 kDa class I heat shock protein	AN-M
MELO3C020601.2	37.422	48.955	5.421	53.584	16.561	166.508	15.578	Protein phosphatase 2C	AN-M
MELO3C020620.2	6.352	7.313	5.948	7.512	5.915	52.048	5.933	telomere repeat-binding protein 5-like	AN-M
MELO3C020623.2	2.797	2.488	1.353	3.555	1.853	16.937	2.332	PGR5-like protein 1B, chloroplastic	AN-M
MELO3C020678.2	2.038	2.211	2.342	1.837	2.261	5.194	2.525	E3 UFM1-protein ligase 1 homolog	AN-M
MELO3C005056.2	1.975	2.822	2.452	3.653	1.512	9.295	1.900	Kelch repeat-containing F-box family protein	AN-M
MELO3C005009.2	0.966	0.456	1.195	0.729	0.337	4.266	0.196	LOB domain-containing protein 1	AN-M
MELO3C004919.2	1.419	1.063	0.695	1.845	1.514	7.536	2.259	TBC1 domain family member 2A	AN-M
MELO3C004871.2	12.951	11.053	10.830	12.294	6.005	42.521	7.622	Glycosyl transferase, family 31	AN-M
MELO3C004795.2	10.868	16.528	2.199	9.811	6.566	75.813	3.934	Arogenate dehydratase	AN-M
MELO3C004704.2	1.378	1.913	2.248	1.887	1.112	3.967	0.880	Glucan endo-1,3-beta-glucosidase, putative	AN-M
MELO3C004681.2	4.683	4.245	2.657	5.054	4.078	12.311	3.532	Mitochondrial fission protein ELM1	AN-M
MELO3C025535.2	1.642	1.429	2.950	1.080	1.498	3.430	1.468	Cytosolic Fe-S cluster assembly factor NARFL	AN-M
MELO3C025521.2	2.804	5.034	3.155	2.334	1.423	12.403	0.226	Hexosyltransferase	AN-M
MELO3C021707.2	1.022	1.031	0.535	1.506	0.587	6.144	0.821	Extracellular ligand-gated ion channel protein (DUF3537)	AN-M

Gene ID	FPKM							Gene Description	Specific in episode
	FS	GI-M	GM-M	AN-M	GI-H	GM-H	AN-H		
MELO3C021721.2	19.403	16.025	6.253	21.553	13.937	52.019	12.837	Plant UBX domain-containing protein 4	AN-M
MELO3C021759.2	5.395	7.118	5.516	5.157	4.664	20.462	7.030	lecithin-cholesterol acyltransferase-like 4	AN-M
MELO3C035473.2	3.614	5.770	3.986	5.174	3.079	14.941	4.957	Glycine cleavage T-protein aminomethyltransferase	AN-M
MELO3C021769.2	0.731	0.850	4.203	0.562	1.637	2.811	0.886	Ribonuclease H2, subunit C	AN-M
MELO3C021806.2	7.942	8.112	7.893	9.098	7.748	35.333	10.100	Zinc finger family protein	AN-M
MELO3C021809.2	2.703	3.882	9.273	3.191	4.736	9.972	3.814	lysine-specific demethylase JMJ18-like isoform X2	AN-M
MELO3C035678.2	2.790	2.278	2.577	2.104	2.764	13.299	3.084	Pentatricopeptide repeat-containing family protein	AN-M
MELO3C002729.2	0.520	0.460	1.563	0.391	0.374	1.270	0.671	Purine permease	AN-M
MELO3C002690.2	0.845	1.322	0.892	0.738	1.199	7.167	1.206	Myosin heavy chain kinase B	AN-M
MELO3C002659.2	1.390	2.352	4.541	2.348	1.900	6.432	1.695	Receptor protein kinase, putative	AN-M
MELO3C002564.2	1.093	1.244	5.801	1.611	1.631	5.700	1.351	Lysine ketoglutarate reductase trans-splicing-like protein	AN-M
MELO3C002468.2	9.790	8.854	13.426	9.452	11.975	20.266	12.161	Tobamovirus multiplication protein 1	AN-M
MELO3C002403.2	20.941	15.016	6.820	16.751	13.645	109.686	25.663	Allantoate deiminase	AN-M
MELO3C002387.2	7.597	6.370	4.925	6.625	5.433	19.577	5.982	chromophore lyase CRL, chloroplastic	AN-M
MELO3C002373.2	0.327	0.632	0.067	0.495	0.171	4.091	NA	Acyl-[acyl-carrier-protein] hydrolase	AN-M
MELO3C002359.2	9.637	9.876	5.820	10.651	5.309	21.739	6.065	exocyst complex component EXO70B1	AN-M
MELO3C002313.2	57.479	60.492	52.339	61.691	68.309	130.280	58.727	Serine/threonine-protein kinase AFC2	AN-M
MELO3C002271.2	1.631	1.690	2.919	1.435	1.324	3.533	1.370	F-box protein SKIP16	AN-M
MELO3C002268.2	29.177	31.024	22.381	25.100	23.229	77.129	25.966	Iron-sulfur cluster assembly protein	AN-M
MELO3C002267.2	7.232	7.615	4.929	9.446	6.789	30.458	7.353	pantothenate kinase 2	AN-M
MELO3C002263.2	0.558	0.503	0.914	0.354	0.243	1.610	0.332	Poly [ADP-ribose] polymerase	AN-M
MELO3C002253.2	1.268	2.119	0.927	0.785	0.934	4.891	0.678	RING-type E3 ubiquitin transferase	AN-M
MELO3C002218.2	2.287	2.362	3.301	2.362	2.389	7.900	2.447	WW domain-binding protein 11 isoform X1	AN-M
MELO3C002215.2	4.655	5.174	3.878	5.599	5.742	43.304	6.310	Calcium-dependent protein kinase	AN-M
MELO3C002199.2	1.850	0.903	0.611	3.129	1.376	7.199	1.155	E3 ubiquitin-protein ligase	AN-M
MELO3C002160.2	0.158	0.184	0.720	0.248	0.034	1.684	0.062	ARF-GAP domain 15	AN-M
MELO3C002138.2	13.532	9.040	6.056	14.858	8.906	35.830	12.416	Pseudouridine synthase, catalytic domain-containing protein	AN-M
MELO3C002077.2	23.323	23.148	9.274	24.826	16.750	51.578	16.931	gastric triacylglycerol lipase isoform X2	AN-M
MELO3C035763.2	0.308	0.531	0.354	0.141	NA	2.251	0.084	Unknown protein	AN-M
MELO3C002052.2	2.251	1.879	1.080	1.813	1.399	6.837	2.511	ADP,ATP carrier protein, mitochondrial	AN-M
MELO3C002049.2	0.349	0.807	1.619	0.451	0.118	1.709	0.109	MADS-box transcription factor	AN-M

Gene ID	FPKM							Gene Description	Specific in episode
	FS	GI-M	GM-M	AN-M	GI-H	GM-H	AN-H		
MELO3C002021.2	14.365	11.954	3.732	14.720	8.752	53.489	7.058	transmembrane protein adipocyte-associated 1 homolog	AN-M
MELO3C002020.2	7.394	5.429	3.092	6.105	3.942	56.346	5.654	Chaperone protein	AN-M
MELO3C002004.2	1.731	2.103	0.848	3.110	0.863	7.473	1.082	At2g03350	AN-M
MELO3C001987.2	2.222	1.747	4.286	1.511	1.293	16.012	0.602	cyclic dof factor 3	AN-M
MELO3C001985.2	46.453	36.630	14.401	36.174	44.745	109.171	42.964	RING/FYVE/PHD zinc finger protein	AN-M
MELO3C001956.2	0.806	0.986	3.547	0.989	0.705	2.843	0.846	Sucrose synthase	AN-M
MELO3C001944.2	0.338	0.536	2.237	0.409	1.227	2.157	1.901	Cotton fiber expressed protein	AN-M
MELO3C024239.2	1.086	0.890	1.046	1.480	0.384	4.589	NA	Mitochondrial carrier protein	AN-M
MELO3C015498.2	1.583	0.728	0.426	1.428	1.157	3.815	0.232	Hydroxyproline-rich glycoprotein family protein	AN-M
MELO3C030290.2	0.388	0.230	2.213	0.531	0.414	1.260	0.325	Unknown protein	AN-M
MELO3C003409.2	0.419	0.278	0.418	0.259	NA	1.881	0.477	VQ motif-containing protein, putative	AN-M
MELO3C009698.2	5.742	4.693	0.967	6.717	5.654	13.706	5.938	PQ-loop repeat family protein / transmembrane family protein	AN-M
MELO3C014521.2	1.195	0.726	1.554	0.689	1.502	2.408	2.047	Protein FAF-like, chloroplastic	AN-M
MELO3C016766.2	0.934	0.845	1.286	1.090	0.334	2.428	0.368	S-RIBONUCLEASE BINDING protein 1	AN-M
MELO3C016400.2	6.501	3.696	6.640	1.894	NA	17.634	1.851	bZIP transcription factor 53	AN-M
MELO3C017712.2	2.410	1.650	3.578	2.537	2.414	5.870	1.954	calmodulin-like protein 3	AN-M
MELO3C019651.2	2.048	1.521	3.308	1.896	1.225	4.191	0.693	peptidyl-prolyl cis-trans isomerase FKBP5	AN-M
MELO3C016920.2	1.770	1.491	0.667	0.843	0.693	4.229	0.955	Receptor-like protein kinase	AN-M
MELO3C005829.2	2.465	3.259	0.472	1.812	NA	7.457	0.778	Plant/K24M7-17 protein	AN-M
MELO3C012161.2	0.555	0.563	0.118	0.373	0.401	1.772	0.663	Adenine nucleotide alpha hydrolases-like superfamily protein, putative	AN-M
MELO3C027442.2	0.439	0.957	1.561	0.507	0.510	0.827	0.663	Beta-glucosidase 18	GI-H
MELO3C027032.2	19.778	50.651	171.236	79.381	52.656	16.984	59.183	ATP synthase subunit a	GI-H
MELO3C027040.2	3.121	13.377	48.648	11.336	5.679	4.906	4.583	17 kDa phloem lectin	GI-H
MELO3C027075.2	0.320	0.456	1.709	0.418	0.371	0.357	0.389	Zinc finger family protein	GI-H
MELO3C027054.2	36.423	39.233	238.368	57.438	37.218	20.740	36.136	NAD(P)H-quinone oxidoreductase subunit H, chloroplastic	GI-H
MELO3C027055.2	24.302	24.109	235.531	36.437	22.799	9.166	21.145	NAD(P)H-quinone oxidoreductase subunit 1, chloroplastic	GI-H
MELO3C027101.2	6.380	5.711	77.611	12.566	6.679	2.277	5.382	DNA-directed RNA polymerase subunit beta	GI-H
MELO3C027102.2	37.386	17.396	193.368	25.915	12.116	6.268	9.012	DNA-directed RNA polymerase subunit beta'	GI-H
MELO3C027088.2	97.188	55.264	326.120	72.383	39.990	23.638	36.429	50S ribosomal protein L20, chloroplastic	GI-H
MELO3C027089.2	566.962	670.882	4869.544	620.022	730.286	598.968	594.448	ATP-dependent Clp protease proteolytic subunit	GI-H

Gene ID	FPKM							Gene Description	Specific in episode
	FS	GI-M	GM-M	AN-M	GI-H	GM-H	AN-H		
MELO3C027090.2	370.786	576.688	1926.155	419.127	448.723	472.634	351.421	ATP-dependent Clp protease proteolytic subunit	GI-H
MELO3C027462.2	361.667	411.576	1645.718	450.983	338.925	187.182	337.573	Cytochrome b559 subunit alpha	GI-H
MELO3C027106.2	50.643	41.551	199.067	59.899	36.307	30.052	41.451	Cytochrome f	GI-H
MELO3C027093.2	0.140	0.210	1.257	NA	0.311	NA	0.345	DExH-box ATP-dependent RNA helicase DExH12-like	GI-H
MELO3C027119.2	1.029	1.439	4.331	0.666	0.919	0.276	1.051	1-aminocyclopropane-1-carboxylate oxidase 2	GI-H
MELO3C027351.2	18.984	13.465	350.097	28.553	18.952	8.639	13.556	Photosystem I assembly protein Ycf3	GI-H
MELO3C027485.2	70.682	32.096	489.956	73.138	48.022	13.632	33.837	30S ribosomal protein S4, chloroplastic	GI-H
MELO3C027413.2	128.482	69.656	1009.419	120.225	78.745	19.788	71.470	Maturase K	GI-H
MELO3C027497.2	308.604	166.899	837.488	292.614	171.632	63.127	140.221	Maturase K	GI-H
MELO3C027174.2	137.519	154.861	508.140	201.748	179.396	89.449	171.856	ATP synthase subunit beta, chloroplastic	GI-H
MELO3C027676.2	0.572	1.015	7.925	1.234	1.168	0.275	1.151	Vacuolar protein sorting-associated protein 33 like	GI-H
MELO3C027524.2	24.104	39.220	142.827	60.676	43.123	9.985	50.319	ATP synthase subunit a	GI-H
MELO3C027218.2	0.432	0.588	6.934	NA	0.832	1.152	1.263	O-fucosyltransferase family protein	GI-H
MELO3C027236.2	367.971	620.852	1237.456	439.966	449.359	362.835	314.627	Photosystem I iron-sulfur center	GI-H
MELO3C027239.2	8.481	4.333	19.984	2.826	6.538	0.637	4.561	high mobility group B protein 7	GI-H
MELO3C027537.2	0.189	NA	1.487	NA	0.097	NA	0.351	ENTH/ANTH/VHS superfamily protein	GI-H
MELO3C027261.2	3.374	3.957	11.673	2.487	4.816	1.474	4.569	lon protease homolog, mitochondrial-like	GI-H
MELO3C027271.2	2.042	1.683	5.629	1.326	2.444	0.733	2.019	alanine--tRNA ligase-like	GI-H
MELO3C027288.2	0.668	1.477	8.761	1.132	2.531	0.376	1.199	Threonine dehydratase	GI-H
MELO3C027823.2	0.805	1.041	2.297	0.688	0.780	0.508	1.019	Ty3/gypsy retrotransposon protein	GI-H
MELO3C027860.2	2.157	2.118	10.915	1.136	1.721	1.559	2.858	NBS-LRR type resistance protein	GI-H
MELO3C027877.2	0.414	0.476	1.601	0.337	0.231	0.247	0.308	methyltransferase-like protein 13	GI-H
MELO3C027373.2	1.746	2.555	8.569	2.577	1.789	5.968	0.982	ADP-ribosylation factor GTPase-activating protein AGD12-like	GI-H
MELO3C027417.2	22.016	22.302	82.397	27.837	14.845	6.184	11.554	NADH dehydrogenase subunit 5	GI-H
MELO3C027395.2	28.157	25.017	258.574	39.494	29.651	11.179	19.702	ATP synthase subunit a, chloroplastic	GI-H
MELO3C027390.2	1.381	1.168	7.089	1.111	2.383	0.739	2.246	Centromere protein Mis12	GI-H
MELO3C027392.2	4.118	2.555	24.438	2.291	5.917	1.578	3.978	60S ribosomal protein L7-1	GI-H
MELO3C000003.2	5.446	10.739	38.199	15.528	7.390	14.798	7.874	Stress-related ozone-induced family protein	GI-H
MELO3C000108.2	46.088	34.536	344.355	52.774	30.537	22.193	30.555	50S ribosomal protein L16, chloroplastic	GI-H
MELO3C000106.2	38.805	36.647	399.580	61.788	41.392	33.847	35.300	30S ribosomal protein S12, chloroplastic	GI-H
MELO3C027605.2	1.197	3.960	31.015	4.509	3.873	3.331	2.927	Ribosomal protein S7	GI-H

Gene ID	FPKM							Gene Description	Specific in episode
	FS	GI-M	GM-M	AN-M	GI-H	GM-H	AN-H		
MELO3C000130.2	1361.562	2672.783	6361.442	2286.286	2642.163	2342.780	1900.816	Ribulose bisphosphate carboxylase large chain	GI-H
MELO3C027614.2	16.451	17.648	90.170	25.765	17.656	17.082	14.332	50S ribosomal protein L2, chloroplastic	GI-H
MELO3C000111.2	42.290	30.725	285.835	54.071	38.088	20.585	31.648	50S ribosomal protein L2, chloroplastic	GI-H
MELO3C000126.2	1.435	1.920	11.692	2.910	1.570	NA	0.838	Protein disulfide-isomerase	GI-H
MELO3C000179.2	30.265	23.521	232.575	53.902	18.919	8.277	14.953	NAD(P)H-quinone oxidoreductase subunit 1, chloroplastic	GI-H
MELO3C000180.2	34.284	24.147	381.460	50.889	28.468	13.173	18.907	NAD(P)H-quinone oxidoreductase subunit I, chloroplastic	GI-H
MELO3C027635.2	0.532	0.407	5.820	0.708	0.560	0.262	0.354	Unknown protein	GI-H
MELO3C027646.2	3.953	8.539	35.227	12.734	9.130	13.003	6.079	Unknown protein	GI-H
MELO3C000196.2	65.886	111.985	530.710	109.894	91.360	88.215	92.945	ATP synthase subunit alpha, chloroplastic	GI-H
MELO3C000827.2	20.250	19.269	472.121	36.639	24.845	10.777	14.479	ATP synthase subunit b, chloroplastic	GI-H
MELO3C000199.2	2.838	4.609	17.993	7.746	4.672	0.362	5.301	Protein disulfide-isomerase	GI-H
MELO3C028189.2	0.237	1.070	1.900	1.450	0.499	1.575	0.766	Unknown protein	GI-H
MELO3C000210.2	28.088	20.345	92.718	11.418	41.047	4.697	41.436	Unknown protein	GI-H
MELO3C000241.2	14.315	11.019	150.377	14.603	8.560	6.943	6.872	50S ribosomal protein L14, chloroplastic	GI-H
MELO3C000228.2	1.363	0.709	3.763	0.476	1.659	5.786	0.296	UBN2_3 domain-containing protein	GI-H
MELO3C001225.2	3.413	3.512	28.719	5.049	3.773	2.884	4.571	Acetyl-coenzyme A carboxylase carboxyl transferase subunit beta, chloroplastic	GI-H
MELO3C000334.2	77.893	52.566	175.500	101.998	34.416	23.307	39.311	Acetyl-coenzyme A carboxylase carboxyl transferase subunit beta, chloroplastic	GI-H
MELO3C000330.2	63.199	42.523	352.848	55.400	29.537	15.138	29.132	DNA-directed RNA polymerase subunit beta''	GI-H
MELO3C027682.2	6.520	6.388	67.551	10.788	5.385	3.723	6.410	DNA-directed RNA polymerase subunit beta''	GI-H
MELO3C001093.2	82.650	123.943	321.383	113.720	114.049	74.386	93.723	DNA-directed RNA polymerase subunit beta''	GI-H
MELO3C000332.2	20.546	28.714	83.993	39.612	28.459	8.516	21.887	DNA-directed RNA polymerase subunit beta	GI-H
MELO3C028357.2	0.620	0.413	2.831	1.719	0.581	15.435	0.514	Serine/threonine-protein phosphatase 7 long form-like protein	GI-H
MELO3C000399.2	194.438	398.966	738.166	336.380	295.940	279.521	242.413	Cytochrome b6-f complex subunit 4	GI-H
MELO3C001317.2	10.977	16.692	88.376	NA	18.464	11.349	11.450	Coiled-coil protein (DUF572)	GI-H
MELO3C000490.2	51.451	31.870	233.749	50.932	12.508	6.139	19.496	Unknown protein	GI-H
MELO3C027742.2	1.851	1.124	6.861	1.189	1.905	0.938	1.804	kinesin-like protein KIN12B	GI-H
MELO3C027746.2	0.316	0.830	9.800	NA	0.730	0.858	0.814	Unknown protein	GI-H
MELO3C027753.2	0.245	1.264	13.587	NA	0.951	1.022	1.048	Unknown protein	GI-H
MELO3C001352.2	13.000	9.863	178.641	22.653	15.262	7.063	14.823	30S ribosomal protein S2, chloroplastic	GI-H
MELO3C001268.2	13.866	9.432	252.169	24.136	12.848	5.033	12.751	DNA-directed RNA polymerase subunit beta''	GI-H

Gene ID	FPKM							Gene Description	Specific in episode
	FS	GI-M	GM-M	AN-M	GI-H	GM-H	AN-H		
MELO3C000601.2	62.411	88.764	230.809	79.017	70.143	69.529	66.302	DNA-directed RNA polymerase subunit beta'	GI-H
MELO3C027778.2	7.630	8.954	88.339	15.114	8.690	3.404	7.567	DNA-directed RNA polymerase subunit beta''	GI-H
MELO3C000655.2	1.307	0.700	7.756	NA	1.353	0.558	0.395	cytochrome P450 76C4	GI-H
MELO3C000684.2	1.491	1.352	6.496	NA	2.953	NA	1.915	Lipoxygenase	GI-H
MELO3C000701.2	3.558	4.580	43.689	9.505	4.957	2.452	4.282	DNA-directed RNA polymerase subunit beta''	GI-H
MELO3C027806.2	46.054	41.523	736.557	76.409	51.399	39.864	35.055	Ribosomal protein S7	GI-H
MELO3C027805.2	40.727	38.815	276.633	49.529	25.958	32.771	24.197	30S ribosomal protein S7, chloroplastic	GI-H
MELO3C000736.2	0.677	0.668	9.313	2.274	1.697	1.937	1.109	Exocyst complex component	GI-H
MELO3C000759.2	40.291	38.194	233.492	58.257	53.438	18.241	41.819	Ribosomal protein L5	GI-H
MELO3C001387.2	69.053	93.030	227.150	76.629	93.889	110.108	96.021	ATP synthase subunit c, chloroplastic	GI-H
MELO3C028468.2	37.267	51.819	179.043	61.787	62.612	38.498	35.710	Unknown protein	GI-H
MELO3C027844.2	34.143	47.921	253.727	50.034	35.227	43.443	52.308	ATP synthase subunit alpha, chloroplastic	GI-H
MELO3C027845.2	26.005	27.448	582.982	44.545	30.936	12.412	19.304	ATP synthase subunit b, chloroplastic	GI-H
MELO3C000822.2	0.190	0.365	1.034	0.336	0.136	NA	0.289	Receptor-like protein kinase, putative	GI-H
MELO3C027856.2	5.105	5.536	35.655	3.512	13.214	2.369	7.446	Coiled-coil domain-containing 73	GI-H
MELO3C001415.2	35.261	28.554	183.678	49.801	32.373	31.563	32.415	50S ribosomal protein L2, chloroplastic	GI-H
MELO3C000879.2	290.204	164.390	833.747	276.882	168.896	56.557	136.607	Maturase K	GI-H
MELO3C000900.2	18.464	24.557	159.261	33.581	22.756	18.799	18.331	Protein Ycf2	GI-H
MELO3C028482.2	3.517	1.899	36.018	3.595	2.519	1.276	1.773	Transposon Ty3-G Gag-Pol polyprotein	GI-H
MELO3C000924.2	21.871	13.947	151.485	29.357	16.458	5.173	19.369	NAD(P)H-quinone oxidoreductase subunit K, chloroplastic	GI-H
MELO3C027885.2	3.867	3.375	13.791	4.145	3.610	4.779	3.365	RNA polymerase subunit	GI-H
MELO3C027886.2	1.632	2.465	19.602	4.016	2.156	1.549	3.028	DNA-directed RNA polymerase subunit beta''	GI-H
MELO3C000995.2	11.810	14.973	250.317	23.580	17.953	9.649	14.972	ATP synthase subunit a, chloroplastic	GI-H
MELO3C028505.2	1.742	1.433	7.874	NA	2.379	1.533	2.388	Pyruvate kinase	GI-H
MELO3C027909.2	2.133	2.126	11.490	1.870	2.320	0.835	1.744	Polyadenylate-binding 2	GI-H
MELO3C001082.2	11.502	10.957	107.650	23.151	9.564	7.722	11.716	Protein TIC 214	GI-H
MELO3C027943.2	17.630	13.161	152.553	33.184	17.270	9.013	12.761	Protein TIC 214	GI-H
MELO3C028522.2	2.023	1.707	9.410	3.054	2.109	0.824	1.178	Protein Ycf2	GI-H
MELO3C001174.2	39.783	42.966	222.182	65.460	40.519	26.794	34.692	Protein Ycf2	GI-H
MELO3C001246.2	19.605	20.492	58.427	33.295	11.777	15.413	6.503	Unknown protein	GI-H
MELO3C001175.2	0.603	0.507	3.665	0.759	0.669	0.499	0.689	Glutathione s-transferase	GI-H

Gene ID	FPKM							Gene Description	Specific in episode
	FS	GI-M	GM-M	AN-M	GI-H	GM-H	AN-H		
MELO3C001187.2	37.571	20.746	176.015	27.257	17.121	16.272	19.419	30S ribosomal protein S11, chloroplastic	GI-H
MELO3C027978.2	15.169	15.514	123.149	20.030	17.166	10.725	20.661	Acetyl-coenzyme A carboxylase carboxyl transferase subunit beta, chloroplastic	GI-H
MELO3C027991.2	27.539	38.195	134.495	49.206	24.630	27.441	20.368	Unknown protein	GI-H
MELO3C028015.2	8.232	5.108	143.175	14.021	7.470	2.599	7.047	DNA-directed RNA polymerase subunit beta''	GI-H
MELO3C000623.2	17.657	11.461	129.129	22.117	7.630	4.806	7.085	30S ribosomal protein S2, chloroplastic	GI-H
MELO3C028074.2	0.587	3.638	4.084	0.891	2.032	NA	0.697	Unknown protein	GI-H
MELO3C028091.2	39.005	32.359	200.406	57.445	38.656	30.935	35.424	50S ribosomal protein L2, chloroplastic	GI-H
MELO3C001514.2	83.998	46.347	437.259	54.414	52.765	20.777	51.209	50S ribosomal protein L33, chloroplastic	GI-H
MELO3C001812.2	21.116	38.522	66.944	9.470	25.440	5.945	19.218	Terpene cyclase/mutase family member	GI-H
MELO3C018407.2	4.990	7.229	17.108	9.330	4.569	22.592	3.786	4-hydroxy-3-methylbut-2-enyl diphosphate reductase	GI-H
MELO3C018417.2	0.428	1.200	1.849	0.550	0.613	1.124	0.880	Pentatricopeptide repeat-containing protein	GI-H
MELO3C018421.2	0.200	0.360	4.585	0.227	0.457	0.409	0.392	BAT2 domain protein	GI-H
MELO3C018426.2	6.208	16.349	38.080	3.107	12.108	1.540	16.143	AT3g19030/K13E13_15	GI-H
MELO3C018437.2	0.222	0.317	2.975	0.282	0.379	0.469	0.243	Pentatricopeptide repeat-containing protein At5g39710	GI-H
MELO3C018438.2	1.011	0.672	2.953	0.551	0.802	0.257	0.502	WW domain-binding protein 4	GI-H
MELO3C018440.2	0.571	0.718	2.724	0.542	0.792	0.538	0.356	Sulfotransferase	GI-H
MELO3C018448.2	0.855	0.586	2.148	1.377	0.856	0.333	0.791	LAG1 longevity assurance homolog 2	GI-H
MELO3C018450.2	0.488	1.808	14.828	10.974	1.000	16.761	2.013	Caffeoyl-CoA O-methyltransferase	GI-H
MELO3C018454.2	0.930	2.730	2.763	2.019	1.015	1.688	0.793	Cold regulated gene 27, putative isoform 3	GI-H
MELO3C018476.2	3.646	3.085	22.643	0.738	6.075	NA	3.974	cyclin-D3-3	GI-H
MELO3C018491.2	0.584	0.635	3.638	1.490	0.588	0.302	1.133	RNA pseudouridine synthase 4, mitochondrial	GI-H
MELO3C018502.2	0.451	0.707	4.622	0.649	0.760	0.890	1.120	MAR-binding filament-like protein	GI-H
MELO3C018512.2	7.892	12.299	55.184	11.399	8.460	3.916	8.887	40S ribosomal protein S29	GI-H
MELO3C018521.2	0.413	0.245	3.293	0.183	0.618	0.243	0.414	DNA polymerase kappa	GI-H
MELO3C018523.2	2.159	3.120	20.801	3.839	4.142	6.491	3.835	ADP-ribosylation factor, putative	GI-H
MELO3C018525.2	1.160	1.259	3.204	0.963	1.026	0.836	0.795	Myosin heavy chain-like protein	GI-H
MELO3C018551.2	1.813	0.692	11.964	0.458	2.942	0.215	2.329	Neurofilament heavy protein	GI-H
MELO3C018552.2	1.500	5.309	4.310	2.159	0.741	8.202	1.036	receptor-like protein kinase HAIKU2	GI-H
MELO3C018559.2	2.829	1.924	8.659	1.314	3.654	0.467	3.646	DNA polymerase V	GI-H
MELO3C018560.2	17.960	17.912	39.467	5.929	19.476	11.217	7.201	Transcription factor GTE4	GI-H

Gene ID	FPKM							Gene Description	Specific in episode
	FS	GI-M	GM-M	AN-M	GI-H	GM-H	AN-H		
MELO3C018577.2	0.439	0.984	2.455	0.973	0.669	0.710	0.823	Inositol-tetrakisphosphate 1-kinase	GI-H
MELO3C018578.2	0.622	1.180	2.197	0.490	0.608	0.415	0.735	L-type lectin-domain containing receptor kinase IX.1-like	GI-H
MELO3C018579.2	1.455	5.372	17.603	5.935	1.957	7.697	1.195	Cysteine proteinase inhibitor	GI-H
MELO3C018586.2	1.182	1.639	4.152	1.199	1.105	1.592	1.524	Partner of Y14-mago	GI-H
MELO3C018589.2	0.146	0.396	4.570	NA	0.916	0.547	0.460	Pentatricopeptide repeat-containing protein, mitochondrial	GI-H
MELO3C018591.2	2.678	3.606	13.421	1.402	3.794	1.579	2.884	Transcription factor, putative	GI-H
MELO3C018595.2	1.609	2.793	4.911	2.045	1.730	3.721	0.634	Transmembrane protein, putative	GI-H
MELO3C018596.2	4.391	4.786	19.087	6.822	8.260	4.685	7.620	Peptidylprolyl isomerase	GI-H
MELO3C018601.2	0.180	0.089	2.130	0.049	0.123	0.016	0.049	MADS box protein	GI-H
MELO3C018606.2	0.947	2.833	7.732	1.453	2.375	1.998	0.950	magnesium-dependent phosphatase 1	GI-H
MELO3C018632.2	0.684	0.723	2.132	1.132	0.690	2.090	0.647	7-deoxyloganetin glucosyltransferase-like	GI-H
MELO3C018639.2	1.313	0.346	31.271	0.264	1.307	0.109	0.979	Protein PATRONUS 1	GI-H
MELO3C018646.2	0.323	0.624	3.731	0.664	0.593	0.433	0.544	CD2-binding family protein	GI-H
MELO3C018648.2	1.480	22.107	9.236	11.217	3.292	9.320	3.098	Annexin	GI-H
MELO3C018687.2	3.883	3.036	9.039	2.497	3.028	1.899	3.158	Forkhead-associated (FHA) domain-containing protein	GI-H
MELO3C018689.2	1.769	2.355	4.803	1.773	0.936	4.698	1.116	nuclear transcription factor Y subunit C-2	GI-H
MELO3C018706.2	1.642	0.750	4.758	0.289	2.283	NA	1.390	mitotic checkpoint serine/threonine-protein kinase BUB1	GI-H
MELO3C018707.2	1.656	1.968	25.888	0.456	4.062	NA	3.643	protein TPX2	GI-H
MELO3C018710.2	1.262	2.870	6.034	2.665	1.275	4.809	1.666	Phospholipid-transporting ATPase	GI-H
MELO3C018716.2	0.320	0.707	1.351	0.307	0.382	1.177	0.450	ras-related protein Rab11D	GI-H
MELO3C018721.2	0.798	1.684	8.224	1.040	0.960	1.191	0.522	Protein arginine N-methyltransferase 7	GI-H
MELO3C018726.2	12.030	19.722	82.119	13.656	26.241	13.677	23.232	14-3-3 protein-like protein	GI-H
MELO3C018741.2	0.923	1.025	4.764	0.547	1.087	0.317	1.306	Pentatricopeptide repeat-containing family protein	GI-H
MELO3C018764.2	1.371	1.908	3.520	1.959	1.619	3.164	1.098	Vacuolar protein sorting-associated protein 18 like	GI-H
MELO3C018768.2	1.236	3.060	9.200	3.174	2.382	1.711	2.045	Wound-responsive family protein	GI-H
MELO3C018769.2	1.951	3.316	11.273	3.109	3.484	0.452	2.313	Wound-responsive family protein	GI-H
MELO3C018770.2	2.048	3.666	13.087	4.073	5.084	2.078	3.393	Wound-responsive family protein	GI-H
MELO3C018777.2	1.501	3.280	7.239	1.102	3.186	0.881	1.875	Serine/threonine-protein kinase	GI-H
MELO3C018781.2	3.262	5.430	13.259	4.287	5.564	9.782	4.265	Leucine-rich repeat family protein	GI-H
MELO3C018803.2	0.328	0.378	4.614	0.434	0.586	0.247	0.674	zinc finger CCCH domain-containing protein 40 isoform X1	GI-H
MELO3C018813.2	1.571	1.401	7.328	1.917	1.681	0.542	1.399	Ribosomal protein L6	GI-H

Gene ID	FPKM							Gene Description	Specific in episode
	FS	GI-M	GM-M	AN-M	GI-H	GM-H	AN-H		
MELO3C018819.2	2.763	3.455	6.266	8.072	1.950	12.532	1.751	Hexosyltransferase	GI-H
MELO3C018840.2	0.892	0.916	3.968	1.182	1.143	NA	1.699	RmlC-like cupins superfamily protein	GI-H
MELO3C018841.2	2.985	2.467	7.843	2.810	3.220	5.114	3.296	Beta-adaptin-like protein	GI-H
MELO3C018866.2	0.518	0.650	6.141	0.995	1.486	0.698	1.102	dnaJ homolog subfamily B member 1	GI-H
MELO3C018870.2	9.902	13.329	33.479	8.101	12.004	6.781	9.678	Nucleic acid-binding, OB-fold-like protein	GI-H
MELO3C018873.2	0.328	1.980	5.968	2.897	1.384	0.415	0.833	Protein IQ-DOMAIN 1	GI-H
MELO3C018882.2	6.607	70.957	53.570	2.803	3.350	33.863	2.917	Unknown protein	GI-H
MELO3C018887.2	0.581	1.463	5.640	2.286	1.417	3.669	1.070	Fimbrin-like family protein	GI-H
MELO3C024214.2	2.089	2.309	6.724	3.914	1.105	6.476	0.568	Cytochrome b561 and domon domain-containing protein	GI-H
MELO3C024213.2	0.848	2.072	5.773	1.378	2.638	0.651	2.426	N-acetyltransferase 9-like protein	GI-H
MELO3C028685.2	0.253	0.376	1.490	0.450	0.692	0.945	0.520	FAR1-related sequence 10	GI-H
MELO3C024196.2	1.730	2.250	4.848	1.170	1.759	3.097	2.051	Phytochrome	GI-H
MELO3C024191.2	0.739	0.833	5.992	0.626	0.690	0.843	0.481	Nudix hydrolase	GI-H
MELO3C024185.2	0.222	0.333	3.223	NA	0.577	0.205	0.557	ribonuclease E/G-like protein, chloroplastic	GI-H
MELO3C024180.2	0.527	0.320	2.831	NA	0.864	0.186	0.437	RRP15-like protein	GI-H
MELO3C024178.2	0.951	0.787	3.811	0.336	1.044	0.727	1.233	Histidine--tRNA ligase	GI-H
MELO3C024174.2	0.465	0.598	5.099	NA	0.570	0.777	0.322	protein CHROMATIN REMODELING 4-like isoform X2	GI-H
MELO3C024167.2	1.659	2.196	21.493	2.948	4.097	3.072	2.323	AP-1 complex subunit sigma-1-like	GI-H
MELO3C028705.2	0.414	0.380	1.726	0.485	0.718	0.238	0.462	Unknown protein	GI-H
MELO3C024149.2	0.183	0.429	1.464	0.269	0.275	0.304	0.516	RAN GTPase-activating protein 1	GI-H
MELO3C024147.2	2.910	3.822	7.510	4.129	2.955	3.263	3.245	Phosphatidylinositol N-acetyglucosaminlytransferase subunit P-related	GI-H
MELO3C024146.2	0.278	0.279	1.483	0.173	0.204	0.219	0.252	Unknown protein	GI-H
MELO3C024139.2	0.183	0.251	1.745	0.085	0.214	0.255	0.160	cyclic pyranopterin monophosphate synthase accessory protein, mitochondrial	GI-H
MELO3C024126.2	0.726	0.874	6.087	0.629	0.917	0.554	1.018	Bystin	GI-H
MELO3C028752.2	1.457	1.027	4.418	1.327	1.573	0.168	1.967	Histone H3	GI-H
MELO3C023727.2	0.251	NA	1.614	0.144	0.531	NA	0.512	CDT1-like protein a, chloroplastic	GI-H
MELO3C023725.2	1.181	2.393	6.936	1.323	2.668	0.846	2.485	Protein ROOT PRIMORDIUM DEFECTIVE 1	GI-H
MELO3C023716.2	2.968	3.429	8.275	2.985	3.407	4.941	3.467	Eukaryotic translation initiation factor 2 family protein isoform 2	GI-H
MELO3C023707.2	0.573	0.766	2.614	0.722	0.529	0.554	0.716	Vps51/Vps67 family (Components of vesicular transport) protein isoform 1	GI-H

Gene ID	FPKM							Gene Description	Specific in episode
	FS	GI-M	GM-M	AN-M	GI-H	GM-H	AN-H		
MELO3C023675.2	0.367	0.258	3.466	0.332	0.224	0.195	0.415	factor of DNA methylation 1-like	GI-H
MELO3C023672.2	1.880	2.043	6.722	1.642	1.806	2.059	2.259	DUF668 family protein	GI-H
MELO3C023668.2	1.161	2.300	6.628	2.633	1.461	3.409	2.289	protein SENSITIVE TO PROTON RHIZOTOXICITY 1	GI-H
MELO3C023650.2	0.144	0.493	1.960	0.265	0.631	0.357	0.533	Pentatricopeptide repeat-containing family protein	GI-H
MELO3C023647.2	0.148	0.672	4.088	NA	0.422	NA	0.448	GDSL esterase/lipase At4g16230-like	GI-H
MELO3C024088.2	0.335	0.535	1.926	0.503	0.672	0.395	0.493	F-box family protein, putative	GI-H
MELO3C024101.2	0.783	1.002	2.145	0.460	1.064	1.245	0.908	RING/FYVE/PHD zinc finger protein, putative	GI-H
MELO3C024106.2	1.073	1.503	4.256	0.679	1.731	0.151	1.411	Homeobox domain-containing protein/DDT domain-containing protein	GI-H
MELO3C024961.2	4.699	4.585	13.195	5.966	4.482	4.482	5.872	heterogeneous nuclear ribonucleoprotein 1	GI-H
MELO3C024949.2	1.782	5.643	8.501	3.887	4.134	24.631	3.517	RPM1-interacting protein 4	GI-H
MELO3C024942.2	0.597	0.680	1.205	0.341	0.410	0.278	0.495	NAD-dependent protein deacetylase SRT1	GI-H
MELO3C024926.2	0.247	0.450	1.945	0.472	0.231	0.311	0.254	two-pore potassium channel 1 isoform X1	GI-H
MELO3C024912.2	0.573	0.662	1.725	0.761	0.694	1.961	0.566	Receptor-like kinase	GI-H
MELO3C024908.2	0.532	0.604	1.588	0.543	0.770	0.575	0.734	RING-type E3 ubiquitin transferase	GI-H
MELO3C013104.2	2.822	2.574	11.969	1.674	3.010	1.049	3.337	Upstream activation factor subunit spp27	GI-H
MELO3C013125.2	0.428	0.865	1.221	0.393	0.484	1.742	0.446	Lipid-binding serum glycoprotein family protein, putative	GI-H
MELO3C013141.2	0.531	0.435	1.266	0.337	0.524	0.098	0.452	1,4-alpha-glucan branching enzyme, putative	GI-H
MELO3C013149.2	0.350	0.430	1.173	0.253	0.408	0.282	0.312	UPF0505 protein C16orf62 homolog isoform X1	GI-H
MELO3C013163.2	5.919	8.798	24.566	8.732	5.455	20.893	3.604	Protein translation factor sui1-like protein	GI-H
MELO3C013167.2	0.870	1.870	5.197	1.295	1.524	4.163	0.874	Ubiquitin thioesterase OTU1-like protein	GI-H
MELO3C013178.2	1.549	3.061	9.522	1.952	2.622	3.818	1.757	1-phosphatidylinositol-3-phosphate 5-kinase	GI-H
MELO3C013179.2	6.199	6.269	19.269	3.709	5.813	4.518	5.087	small nuclear ribonucleoprotein E-like	GI-H
MELO3C013180.2	1.988	4.257	10.811	5.583	4.374	3.767	3.460	Ras-like protein	GI-H
MELO3C013185.2	1.591	1.443	5.259	1.046	1.985	0.198	1.889	p-loop nucleoside triphosphate hydrolase superfamily protein	GI-H
MELO3C013188.2	0.515	0.400	2.187	0.228	0.720	NA	0.646	Pentatricopeptide repeat-containing protein	GI-H
MELO3C013197.2	0.234	0.482	1.196	0.381	0.452	0.228	0.311	Regulatory protein recX	GI-H
MELO3C013198.2	0.598	0.559	4.336	NA	1.092	0.064	0.815	kinesin KP1	GI-H
MELO3C013201.2	1.359	1.316	4.233	1.344	1.207	2.048	1.420	nitric oxide synthase-interacting protein-like	GI-H
MELO3C013202.2	0.584	0.760	4.602	1.075	0.995	1.194	0.901	Uridine kinase	GI-H
MELO3C013212.2	0.378	0.454	5.217	1.041	0.490	0.629	0.516	BTB/POZ domain-containing family protein	GI-H

Gene ID	FPKM							Gene Description	Specific in episode
	FS	GI-M	GM-M	AN-M	GI-H	GM-H	AN-H		
MELO3C013225.2	3.942	3.563	11.144	3.613	3.022	4.743	3.305	Phosphatidylinositol/phosphatidylcholine transfer protein SFH13	GI-H
MELO3C013233.2	0.624	0.629	2.849	NA	1.188	0.535	1.075	Rho GTPase-activating protein	GI-H
MELO3C013247.2	0.528	0.879	1.650	0.356	0.808	0.660	0.474	Pentatricopeptide repeat (PPR) superfamily protein	GI-H
MELO3C013249.2	0.274	0.860	5.684	0.873	0.634	0.476	0.427	Alpha-1,3-mannosyl-glycoprotein 2-beta-N-acetylglucosaminyltransferase	GI-H
MELO3C013256.2	0.808	0.513	1.830	0.449	0.617	0.682	0.693	Pentatricopeptide repeat-containing protein At2g17140	GI-H
MELO3C013289.2	1.208	3.601	6.276	1.359	1.104	1.397	2.493	expansin-like A2	GI-H
MELO3C013308.2	0.130	0.469	1.251	NA	0.406	0.433	0.209	PTI1-like tyrosine-protein kinase 2 isoform X1	GI-H
MELO3C013310.2	0.341	0.586	1.699	0.795	0.484	12.633	0.530	O-methyltransferase, putative	GI-H
MELO3C013333.2	1.308	1.707	8.742	1.908	1.942	1.269	1.519	dnaJ homolog subfamily C member 17	GI-H
MELO3C013346.2	0.117	0.464	6.691	0.643	0.406	0.601	0.362	Phospholipase-like protein (PEARLI 4) family protein	GI-H
MELO3C013349.2	3.442	6.793	21.067	4.612	4.763	4.049	5.053	SKP1-like protein	GI-H
MELO3C013410.2	1.190	2.910	11.449	2.726	2.847	2.460	2.030	NADP dependent sorbitol 6-phosphate dehydrogenase family protein	GI-H
MELO3C013421.2	0.115	0.065	1.116	NA	0.089	0.098	0.114	DNA-directed RNA polymerase subunit beta	GI-H
MELO3C013443.2	1.038	0.843	3.945	0.655	1.069	1.400	1.105	RNA-binding protein 28	GI-H
MELO3C012530.2	1.569	1.231	3.169	1.297	1.209	1.858	1.401	SWI/SNF complex subunit SWI3D	GI-H
MELO3C012564.2	4.784	5.991	24.596	5.909	4.295	4.023	4.251	Ubiquitin-conjugating enzyme	GI-H
MELO3C012571.2	1.532	1.889	8.812	1.032	2.569	0.507	1.928	1-acyl-sn-glycerol-3-phosphate acyltransferase	GI-H
MELO3C012575.2	4.254	8.524	21.495	8.508	10.537	2.706	8.072	S-adenosyl-L-methionine-dependent methyltransferase superfamily protein	GI-H
MELO3C012577.2	0.292	0.267	1.395	0.324	0.279	0.335	0.324	Zinc finger CCCH domain protein	GI-H
MELO3C012583.2	0.664	0.627	3.539	0.426	0.574	0.370	0.680	nuclear-pore anchor-like	GI-H
MELO3C012592.2	1.614	1.217	4.158	0.426	0.880	0.199	0.809	Ent-kaurene synthase	GI-H
MELO3C012596.2	0.657	0.284	8.339	NA	1.286	NA	0.665	Cyclin	GI-H
MELO3C028792.2	0.034	NA	1.603	0.189	0.190	NA	0.122	callose synthase 7	GI-H
MELO3C012664.2	2.798	3.685	9.275	2.281	3.495	2.033	3.424	Regulator of nonsense transcripts 1	GI-H
MELO3C028797.2	0.545	0.499	1.694	0.503	0.514	0.362	0.482	Unknown protein	GI-H
MELO3C012683.2	1.006	0.890	3.974	0.908	1.693	0.510	0.615	Pentatricopeptide repeat-containing protein, mitochondrial	GI-H
MELO3C012695.2	0.585	0.522	1.886	0.607	0.644	0.296	0.591	la-related protein 6B isoform X1	GI-H
MELO3C012708.2	0.991	0.971	3.893	0.889	1.621	0.601	0.877	Pentatricopeptide repeat-containing protein	GI-H
MELO3C012713.2	0.424	1.388	7.740	1.219	1.084	1.068	0.910	Zinc finger, CCCH-type	GI-H

Gene ID	FPKM							Gene Description	Specific in episode
	FS	GI-M	GM-M	AN-M	GI-H	GM-H	AN-H		
MELO3C012715.2	0.188	0.170	2.032	0.291	0.418	0.199	0.152	mediator of RNA polymerase II transcription subunit 19a-like	GI-H
MELO3C012724.2	3.244	3.335	19.701	1.374	5.619	NA	5.425	Lipid transfer protein	GI-H
MELO3C012727.2	0.711	0.490	13.724	1.401	1.379	2.731	1.145	Fimbrin-like family protein	GI-H
MELO3C012728.2	0.521	1.069	6.594	0.733	1.462	0.658	1.812	Vacuolar sorting-associated protein 2-like protein	GI-H
MELO3C012730.2	0.842	0.887	2.728	0.585	0.675	0.642	0.675	Structural maintenance of chromosomes protein	GI-H
MELO3C012749.2	0.663	1.490	6.005	1.355	1.476	0.865	1.720	Mitochondrial inner membrane protease subunit 1	GI-H
MELO3C012751.2	0.048	0.125	1.208	0.209	0.179	0.329	0.161	Kinase family protein	GI-H
MELO3C012752.2	2.443	6.729	9.287	2.780	4.227	5.616	3.424	Receptor-like kinase	GI-H
MELO3C012759.2	0.308	0.423	1.390	0.316	0.381	0.088	0.261	Phosphoglycolate phosphatase	GI-H
MELO3C012764.2	0.566	2.073	2.940	36.998	0.798	34.986	1.053	BnaCnng14650D protein	GI-H
MELO3C021024.2	0.689	0.692	2.593	0.595	0.548	1.042	0.601	Myosin-binding protein 3	GI-H
MELO3C021025.2	0.572	0.984	3.272	1.654	1.116	2.241	0.754	Phosphatidylinositol/phosphatidylcholine transfer protein SFH9	GI-H
MELO3C021033.2	0.586	0.946	3.579	0.756	1.211	0.258	0.843	Spindle and kinetochore-associated protein 1-like protein	GI-H
MELO3C021034.2	0.307	0.308	4.739	0.503	0.918	0.133	0.141	Gibberellin regulated protein	GI-H
MELO3C028853.2	0.477	0.497	1.989	0.376	0.434	0.380	0.353	sm-like protein LSM2	GI-H
MELO3C028851.2	0.164	0.109	2.135	0.151	0.089	0.086	0.156	Unknown protein	GI-H
MELO3C021054.2	1.201	1.445	4.230	1.001	0.908	1.436	1.376	Sterile alpha motif (SAM) domain-containing protein	GI-H
MELO3C021072.2	2.566	3.153	8.271	1.745	2.287	2.541	2.391	p-loop containing nucleoside triphosphate hydrolases superfamily protein, putative	GI-H
MELO3C029169.2	3.012	3.305	34.798	6.542	3.824	2.778	4.194	At3g07230	GI-H
MELO3C028860.2	0.458	NA	2.725	0.365	0.343	0.133	0.068	ethylene-responsive transcription factor-like protein At4g13040	GI-H
MELO3C021084.2	4.331	3.066	11.441	2.017	3.594	1.557	3.173	Membrane protein of er body-like protein	GI-H
MELO3C021095.2	0.764	0.819	2.142	0.428	0.775	0.942	1.041	Transcription initiation factor tfiid subunit 1	GI-H
MELO3C021101.2	0.579	0.762	1.936	1.153	0.521	1.658	0.579	transcription factor bHLH78	GI-H
MELO3C021122.2	0.791	0.865	3.196	0.482	1.002	0.459	0.729	Nucleolar pre-ribosomal-associated protein	GI-H
MELO3C021127.2	0.493	0.463	1.285	0.528	0.442	0.200	0.572	Rhodanese-like domain-containing protein 4A, chloroplastic	GI-H
MELO3C021129.2	5.568	6.044	17.244	4.282	6.488	4.777	5.767	FRIGIDA-like protein	GI-H
MELO3C015685.2	0.588	0.993	3.444	0.910	0.820	NA	0.998	Mitochondrial transcription termination factor family protein	GI-H
MELO3C015688.2	2.094	1.980	4.941	1.112	1.833	3.114	1.626	protein FAR1-RELATED SEQUENCE 5	GI-H
MELO3C015698.2	0.653	1.010	1.920	0.957	0.239	2.307	0.742	Pentatricopeptide repeat-containing protein	GI-H
MELO3C015712.2	0.443	0.780	14.656	0.850	0.595	0.411	0.777	DNA-directed RNA polymerases II and IV subunit 5A	GI-H

Gene ID	FPKM							Gene Description	Specific in episode
	FS	GI-M	GM-M	AN-M	GI-H	GM-H	AN-H		
MELO3C015719.2	2.281	2.111	11.650	1.362	3.074	1.859	3.132	RING-type E3 ubiquitin transferase	GI-H
MELO3C015722.2	0.495	0.762	2.882	0.422	0.759	0.439	0.540	Pentatricopeptide repeat-containing family protein	GI-H
MELO3C029191.2	0.220	0.493	1.193	0.290	0.314	0.378	0.137	protein FAR1-RELATED SEQUENCE 5	GI-H
MELO3C015733.2	1.910	1.631	5.296	0.838	1.270	1.485	1.486	Pre-mRNA-splicing factor ATP-dependent RNA helicase	GI-H
MELO3C015740.2	0.761	0.899	3.479	0.348	0.997	NA	0.989	Nephrocystin-3	GI-H
MELO3C029199.2	0.109	0.156	4.664	0.283	0.329	0.133	0.314	26S proteasome non-ATPase regulatory subunit 12	GI-H
MELO3C015748.2	0.639	1.003	5.896	0.852	2.018	0.599	1.336	transcription factor EMB1444	GI-H
MELO3C015762.2	0.601	0.563	6.057	3.229	0.503	9.864	0.504	Auxin-responsive protein	GI-H
MELO3C015774.2	0.497	0.568	1.542	0.814	0.329	4.767	0.345	Hexosyltransferase	GI-H
MELO3C028898.2	0.589	0.521	2.025	0.417	0.591	0.328	0.295	Pentatricopeptide repeat-containing family protein	GI-H
MELO3C015779.2	1.399	1.696	8.799	1.193	1.595	0.486	2.155	peptidyl-prolyl cis-trans isomerase CYP63	GI-H
MELO3C015780.2	0.212	0.514	2.159	0.170	0.296	NA	0.250	Calcium-transporting ATPase	GI-H
MELO3C028901.2	6.690	6.352	26.693	5.675	5.228	7.853	5.084	NBS-LRR type resistance protein	GI-H
MELO3C028900.2	3.900	3.761	15.647	3.270	2.757	6.383	2.845	LOW QUALITY PROTEIN: gamma-aminobutyrate transaminase POP2, mitochondrial-like	GI-H
MELO3C015795.2	0.604	1.151	1.481	0.537	0.346	14.961	0.315	Serine/Threonine kinase family protein	GI-H
MELO3C015797.2	1.097	2.421	16.768	2.203	1.865	2.504	1.287	protein NETWORKED 1D	GI-H
MELO3C015801.2	1.077	1.330	5.436	0.777	1.850	0.396	1.108	2-oxoglutarate (2OG) and Fe(II)-dependent oxygenase superfamily protein	GI-H
MELO3C015809.2	1.182	1.207	8.389	0.942	1.337	0.484	1.537	guanine nucleotide-binding protein-like NSN1	GI-H
MELO3C015821.2	0.439	0.489	1.432	0.484	0.535	0.932	0.438	Pentatricopeptide repeat-containing protein	GI-H
MELO3C015830.2	3.709	3.480	7.657	3.602	2.075	9.578	2.957	Time for coffee	GI-H
MELO3C015833.2	2.139	4.666	10.826	1.946	3.637	2.355	2.292	cyclin-P3-1	GI-H
MELO3C015835.2	0.184	0.407	1.005	0.271	0.360	0.722	0.282	Exostosin family protein	GI-H
MELO3C015840.2	3.769	2.725	8.271	1.696	3.302	2.047	3.534	Pentatricopeptide repeat-containing family protein	GI-H
MELO3C015855.2	0.491	0.405	1.717	0.296	0.365	0.325	0.478	Protein ROOT PRIMORDIUM DEFECTIVE 1	GI-H
MELO3C015856.2	1.252	2.240	7.962	1.335	1.410	2.947	1.408	ELKS/Rab6-interacting/CAST family protein	GI-H
MELO3C015869.2	2.561	2.905	9.260	3.057	3.984	6.066	2.417	BSD domain-containing protein, putative	GI-H
MELO3C015900.2	0.255	0.346	4.960	0.248	0.239	0.311	0.179	DNA ligase 1-like	GI-H
MELO3C015909.2	2.424	3.099	7.241	1.549	2.802	5.385	2.053	Zinc finger protein 469	GI-H
MELO3C015916.2	0.611	1.205	6.856	1.833	0.973	1.493	0.997	Peptidyl-prolyl cis-trans isomerase	GI-H
MELO3C015922.2	3.977	6.174	15.980	5.482	5.328	12.575	4.364	cytochrome c oxidase subunit 5C-like	GI-H

Gene ID	FPKM							Gene Description	Specific in episode
	FS	GI-M	GM-M	AN-M	GI-H	GM-H	AN-H		
MELO3C015938.2	2.254	2.743	5.964	3.915	2.244	4.399	1.946	dnaJ protein ERDJ3B	GI-H
MELO3C015944.2	0.277	0.570	1.479	NA	0.479	NA	0.449	LOW QUALITY PROTEIN: methionine aminotransferase-like	GI-H
MELO3C015955.2	2.627	5.761	7.878	2.497	3.372	3.675	2.874	Protein DEHYDRATION-INDUCED 19	GI-H
MELO3C015957.2	0.380	0.572	5.559	0.309	0.849	0.288	0.481	Myb/SANT-like DNA-binding domain protein	GI-H
MELO3C015960.2	0.804	1.566	4.651	0.720	1.339	1.071	1.076	ATP-dependent RNA helicase, putative	GI-H
MELO3C015988.2	35.695	32.066	79.084	36.170	29.261	14.489	33.354	Ribosomal protein L19	GI-H
MELO3C015996.2	0.368	0.597	3.272	NA	0.763	0.269	0.353	Poly [ADP-ribose] polymerase	GI-H
MELO3C015997.2	1.440	1.504	6.739	1.188	1.337	0.573	1.509	U3 small nucleolar RNA-associated protein 14	GI-H
MELO3C016019.2	1.115	1.304	5.149	1.138	2.234	2.252	1.573	heat shock factor-binding protein 1	GI-H
MELO3C016045.2	0.649	0.801	3.573	0.794	0.625	0.782	0.804	homeobox protein LUMINIDEPENDENS	GI-H
MELO3C016046.2	2.216	3.215	6.378	1.962	2.365	5.289	2.282	Zinc finger, CW-type	GI-H
MELO3C016052.2	0.504	1.909	3.151	0.789	0.987	4.256	0.419	Zinc metalloproteinase aureolysin	GI-H
MELO3C016054.2	1.156	1.586	4.517	0.769	1.099	1.142	0.890	OTU-like cysteine protease family protein	GI-H
MELO3C023620.2	0.848	2.076	6.542	NA	1.960	NA	1.363	Short-chain dehydrogenase/reductase	GI-H
MELO3C029241.2	0.217	0.252	1.704	0.396	0.350	0.172	0.294	Unknown protein	GI-H
MELO3C023605.2	1.125	1.714	3.256	0.509	0.764	0.575	0.597	Receptor-like kinase	GI-H
MELO3C023602.2	0.644	0.771	3.372	0.608	0.695	0.642	0.684	Protein disulfide-isomerase	GI-H
MELO3C023601.2	3.455	4.820	10.605	3.597	3.175	4.635	4.756	ATPase, AAA-type, core	GI-H
MELO3C023594.2	7.482	9.465	133.200	15.751	20.075	3.761	18.002	60S ribosomal protein L44	GI-H
MELO3C023582.2	2.930	2.277	6.514	3.031	2.624	0.672	2.646	Ribosomal protein	GI-H
MELO3C023581.2	2.986	3.235	15.281	2.210	2.826	2.925	2.365	Structural maintenance of chromosomes protein 5	GI-H
MELO3C023572.2	1.299	1.217	2.972	1.037	0.973	0.806	1.178	Pentatricopeptide repeat-containing family protein	GI-H
MELO3C023565.2	4.132	5.012	8.728	3.605	3.488	5.660	3.440	OBERON-like protein	GI-H
MELO3C023564.2	0.780	1.192	2.010	0.808	0.925	1.943	0.926	ARM repeat superfamily protein	GI-H
MELO3C023561.2	1.640	1.960	4.301	1.043	1.735	1.461	1.811	Zinc finger, PHD-type	GI-H
MELO3C023554.2	1.168	2.252	13.938	1.388	5.005	0.825	5.234	nuclear transcription factor Y subunit A-9-like	GI-H
MELO3C023552.2	0.085	0.185	2.485	NA	0.100	0.091	0.098	Folate-sensitive fragile site protein	GI-H
MELO3C023546.2	0.389	0.442	2.578	0.211	0.392	NA	0.521	E3 ubiquitin ligase BIG BROTHER	GI-H
MELO3C023532.2	1.092	4.085	5.544	0.356	1.310	3.947	0.689	transcription factor bHLH130-like isoform X2	GI-H
MELO3C023523.2	0.421	1.113	5.054	2.058	0.679	NA	1.628	RNA-binding family protein	GI-H
MELO3C023518.2	0.367	0.484	2.144	0.572	0.924	0.293	0.496	Box C/D snoRNA protein 1	GI-H

Gene ID	FPKM							Gene Description	Specific in episode
	FS	GI-M	GM-M	AN-M	GI-H	GM-H	AN-H		
MELO3C023505.2	0.370	1.235	4.253	0.609	0.777	1.100	0.582	cyclic pyranopterin monophosphate synthase, mitochondrial	GI-H
MELO3C023495.2	0.035	0.060	1.225	0.328	0.111	1.464	0.082	myosin-12	GI-H
MELO3C023486.2	0.693	1.055	1.747	1.048	0.809	4.877	0.869	Snf1-related kinase interactor 1, putative	GI-H
MELO3C023485.2	0.852	0.956	4.910	0.675	1.635	NA	2.167	aspartyl protease family protein At5g10770	GI-H
MELO3C023481.2	1.528	2.093	4.084	1.124	1.562	1.606	1.470	nudix hydrolase 3-like	GI-H
MELO3C023479.2	0.648	0.993	2.278	0.883	1.110	0.422	0.764	nudix hydrolase 3-like	GI-H
MELO3C023476.2	13.342	21.533	73.271	25.733	26.383	29.668	21.391	26S protease regulatory subunit	GI-H
MELO3C023470.2	2.217	1.714	6.539	0.753	2.401	0.759	3.108	Receptor-like kinase	GI-H
MELO3C023464.2	3.013	3.302	9.677	1.675	4.399	1.129	3.226	sister chromatid cohesion protein PDS5 homolog B-B-like	GI-H
MELO3C023445.2	7.315	16.071	16.915	5.809	6.806	21.164	7.448	F-box/LRR-repeat protein 14	GI-H
MELO3C023443.2	0.379	2.211	3.013	0.514	1.346	1.404	0.777	calcineurin subunit B	GI-H
MELO3C023419.2	0.266	0.466	6.907	0.503	0.668	0.097	0.358	methyl-CpG-binding domain-containing protein 11	GI-H
MELO3C023408.2	1.779	11.078	11.578	11.381	2.908	11.405	3.075	Lactoylglutathione lyase	GI-H
MELO3C023404.2	1.295	1.178	6.491	2.478	1.311	1.367	1.207	DNA replication complex GINS protein PSF1	GI-H
MELO3C023402.2	1.160	1.151	3.894	1.263	1.763	3.518	1.624	Biotin carboxyl carrier protein of acetyl-CoA carboxylase	GI-H
MELO3C023395.2	2.179	2.403	7.386	2.285	2.289	1.418	2.642	39S ribosomal protein L41-A, mitochondrial-like	GI-H
MELO3C023383.2	0.171	0.184	1.538	3.104	0.298	6.641	0.296	Pleiotropic drug resistance ABC transporter	GI-H
MELO3C023372.2	0.390	0.671	3.984	NA	1.088	0.203	0.707	cyclin-A2-4-like	GI-H
MELO3C023366.2	1.174	1.342	6.183	NA	2.268	0.463	1.590	Centromere protein C	GI-H
MELO3C023361.2	3.761	2.968	12.135	2.352	5.150	0.871	4.011	Thionin-like protein 2	GI-H
MELO3C024228.2	0.818	0.793	3.190	0.652	0.611	0.397	0.505	MAR-binding filament-like protein 1-1	GI-H
MELO3C024231.2	0.913	0.805	3.553	0.457	1.368	0.491	1.425	regulator of telomere elongation helicase 1 isoform X1	GI-H
MELO3C024246.2	1.457	2.102	3.723	1.786	0.638	4.840	1.809	ras-related protein Rab7	GI-H
MELO3C024262.2	0.336	0.995	7.421	NA	1.600	0.515	1.244	activating signal cointegrator 1 complex subunit 1	GI-H
MELO3C024279.2	0.816	0.690	4.095	0.902	0.617	1.136	0.963	Pre-mRNA-processing protein 40C	GI-H
MELO3C024290.2	4.128	3.747	65.663	4.630	7.886	0.992	7.841	60S ribosomal protein L7	GI-H
MELO3C024292.2	6.028	8.470	27.880	12.905	12.972	5.387	10.839	O-fucosyltransferase family protein	GI-H
MELO3C024297.2	1.522	3.121	12.698	4.084	3.445	16.112	2.400	ATP synthase subunit epsilon, mitochondrial	GI-H
MELO3C024305.2	2.362	3.095	8.815	1.568	3.322	1.790	2.789	pumilio homolog 23	GI-H
MELO3C024324.2	2.501	2.340	8.644	1.426	2.853	0.698	1.326	Chaperone protein DnaJ	GI-H
MELO3C024331.2	3.790	3.787	8.735	6.075	3.142	6.185	2.452	25.3 kDa vesicle transport protein	GI-H

Gene ID	FPKM							Gene Description	Specific in episode
	FS	GI-M	GM-M	AN-M	GI-H	GM-H	AN-H		
MELO3C024357.2	0.651	0.842	3.992	0.796	0.957	1.263	1.011	transcription initiation factor TFIID subunit 12b	GI-H
MELO3C024364.2	4.552	3.964	9.789	3.257	3.807	4.873	3.970	phospholipid:diacylglycerol acyltransferase 1	GI-H
MELO3C024365.2	0.454	0.395	3.030	0.598	0.745	0.468	0.270	PHD finger alfin-like protein	GI-H
MELO3C024369.2	2.858	2.620	5.822	1.327	2.710	0.467	2.788	Midasin	GI-H
MELO3C024372.2	2.254	1.333	8.289	2.093	2.781	0.736	3.251	DNA-directed RNA polymerase I subunit rpa49	GI-H
MELO3C024383.2	0.302	0.565	3.043	0.572	0.814	0.523	0.210	beta-fructofuranosidase, insoluble isoenzyme CWINV1-like	GI-H
MELO3C024385.2	0.724	1.059	3.866	0.297	1.569	0.657	1.391	protein indeterminate-domain 7	GI-H
MELO3C024395.2	1.781	2.877	4.652	2.071	1.381	3.784	1.536	WAS/WASL-interacting family protein	GI-H
MELO3C024398.2	0.843	1.532	2.365	0.746	0.941	1.085	0.881	ABC transporter C family member 8	GI-H
MELO3C024403.2	0.610	0.721	5.258	0.685	0.505	0.605	0.371	RWD domain-containing protein 1	GI-H
MELO3C024404.2	1.894	1.236	4.284	0.934	1.060	1.814	1.563	Tudor/PWWP/MBT superfamily protein	GI-H
MELO3C024405.2	0.388	0.175	2.122	NA	0.523	0.371	0.226	BnaA05g36700D protein	GI-H
MELO3C024410.2	1.768	1.387	4.556	0.499	1.354	8.161	1.170	Protein EARLY FLOWERING 3	GI-H
MELO3C024423.2	0.618	1.443	2.326	0.495	0.413	0.302	0.567	Receptor-like protein kinase	GI-H
MELO3C024426.2	0.345	0.465	2.715	NA	0.477	0.247	0.592	Receptor-like protein kinase family	GI-H
MELO3C024432.2	0.335	0.395	1.820	0.369	0.553	0.735	0.288	DNA-directed RNA polymerase III subunit RPC7-like isoform X1	GI-H
MELO3C024438.2	1.332	1.414	5.972	1.920	1.692	1.898	1.522	RNA polymerase II-associated, Paf1	GI-H
MELO3C024455.2	0.475	0.490	1.410	0.337	0.542	0.183	0.567	DNA-directed RNA polymerase subunit beta	GI-H
MELO3C028985.2	0.871	0.672	2.273	0.350	0.469	0.613	0.801	ATP-dependent zinc metalloprotease FtsH	GI-H
MELO3C015225.2	1.912	1.861	6.032	1.023	2.827	0.417	2.529	Flap endonuclease 1	GI-H
MELO3C015227.2	4.744	5.493	12.553	6.640	6.190	11.136	5.521	glutamate--tRNA ligase, cytoplasmic	GI-H
MELO3C015237.2	0.712	0.982	4.027	0.998	0.608	2.658	0.600	titin isoform X2	GI-H
MELO3C015241.2	0.552	0.318	5.795	0.198	0.272	0.303	0.309	G patch domain-containing protein 8	GI-H
MELO3C015263.2	0.237	0.337	1.798	0.198	0.511	0.148	0.109	Werner syndrome-like exonuclease	GI-H
MELO3C015269.2	15.933	18.124	36.150	22.028	16.536	6.899	13.085	60S ribosomal protein L39	GI-H
MELO3C015274.2	1.447	1.425	4.908	0.939	1.234	2.738	0.888	PHD and RING finger domain-containing 1	GI-H
MELO3C015281.2	0.904	1.132	6.459	1.355	1.358	0.981	0.939	calmodulin-binding protein 60 G-like	GI-H
MELO3C015283.2	0.201	0.573	5.787	0.344	0.642	0.327	0.251	calmodulin-binding protein 60 B-like isoform X2	GI-H
MELO3C015288.2	0.252	0.523	1.829	0.710	0.639	0.487	0.363	Pectinesterase	GI-H
MELO3C015295.2	49.839	38.345	170.204	63.801	73.524	27.419	78.832	Heat shock protein 90	GI-H
MELO3C015304.2	16.825	20.667	45.974	16.705	17.163	7.761	17.280	60S ribosomal protein L22-2	GI-H

Gene ID	FPKM							Gene Description	Specific in episode
	FS	GI-M	GM-M	AN-M	GI-H	GM-H	AN-H		
MELO3C015335.2	0.727	0.318	2.699	1.046	0.296	0.106	1.109	signal peptidase complex subunit 3B-like	GI-H
MELO3C015336.2	0.976	0.986	10.153	0.881	1.382	0.324	1.305	cell wall protein IFF6-like	GI-H
MELO3C015343.2	0.373	0.266	2.677	0.461	0.525	NA	0.630	39S ribosomal protein L45, mitochondrial isoform X1	GI-H
MELO3C015345.2	1.323	2.049	4.594	2.398	1.259	4.783	1.419	Glutathione synthetase	GI-H
MELO3C015359.2	0.651	1.735	11.746	1.083	0.820	0.320	0.880	histidine-containing phosphotransfer protein 1-like	GI-H
MELO3C015360.2	0.707	0.406	1.710	0.244	0.690	0.615	0.469	transcription factor IIIB 60 kDa subunit	GI-H
MELO3C015366.2	1.718	1.003	10.190	0.909	1.519	0.938	1.845	DnaJ subfamily B member 14	GI-H
MELO3C015383.2	2.260	2.442	10.668	1.575	4.013	0.549	3.406	kinesin-like protein KIFC3	GI-H
MELO3C015398.2	0.869	1.616	8.506	0.785	1.662	0.326	0.971	cysteine synthase-like	GI-H
MELO3C015403.2	9.411	24.140	22.384	7.212	6.369	32.765	6.104	Avr9/Cf-9 rapidly elicited protein	GI-H
MELO3C015421.2	2.871	3.370	11.069	2.669	2.179	0.693	3.084	programmed cell death protein 4-like	GI-H
MELO3C015422.2	2.982	2.883	10.682	3.183	4.781	1.371	3.580	Arginine--tRNA ligase	GI-H
MELO3C015429.2	0.707	1.178	5.816	1.923	1.376	1.402	0.628	AP-4 complex subunit sigma	GI-H
MELO3C015430.2	4.861	7.659	23.898	4.972	3.244	8.065	2.608	Inositol oxygenase	GI-H
MELO3C015431.2	13.755	29.983	81.678	24.285	21.786	10.565	27.204	40S ribosomal protein S30	GI-H
MELO3C015433.2	4.197	5.747	23.202	6.615	5.057	12.884	3.224	Unknown protein	GI-H
MELO3C015438.2	2.976	1.808	40.786	4.079	4.352	0.605	3.889	60S ribosomal protein L31	GI-H
MELO3C015441.2	0.760	1.289	5.040	1.160	0.675	1.262	0.618	IST1-like protein	GI-H
MELO3C015443.2	0.550	1.300	6.530	1.847	1.057	0.950	1.093	Mitochondrial ATP synthase subunit G protein	GI-H
MELO3C015447.2	2.226	2.652	16.179	2.221	4.463	1.979	3.341	Protein arginine N-methyltransferase	GI-H
MELO3C015448.2	0.993	1.431	9.208	3.433	2.618	0.924	1.986	AT4G29520-like protein	GI-H
MELO3C015470.2	6.888	29.274	37.826	25.703	12.744	166.235	11.403	beta-galactosidase	GI-H
MELO3C015471.2	0.831	1.550	2.540	5.702	0.960	7.382	1.245	beta-galactosidase-like	GI-H
MELO3C029330.2	0.721	1.282	7.337	0.998	1.005	0.732	0.967	Translation machinery-associated protein 22	GI-H
MELO3C015483.2	0.197	0.303	2.878	0.204	0.292	NA	0.220	Alpha/beta-Hydrolases superfamily protein	GI-H
MELO3C015490.2	0.458	1.688	2.580	0.326	0.431	0.293	0.684	Calmodulin-binding family protein	GI-H
MELO3C015494.2	0.913	1.793	7.047	1.139	1.499	1.711	1.286	Lactoylglutathione lyase / glyoxalase I family protein	GI-H
MELO3C015496.2	1.250	1.907	7.901	1.224	1.837	2.002	1.023	Plant/F27B13-30 protein	GI-H
MELO3C029338.2	0.794	1.259	4.606	0.936	0.961	1.150	1.166	GSVIVT00016514001	GI-H
MELO3C015497.2	0.582	1.224	13.922	0.581	1.994	0.514	1.557	LOW QUALITY PROTEIN: mitogen-activated protein kinase kinase 2-like	GI-H

Gene ID	FPKM							Gene Description	Specific in episode
	FS	GI-M	GM-M	AN-M	GI-H	GM-H	AN-H		
MELO3C015501.2	3.033	3.650	10.992	3.121	3.871	7.834	3.883	myosin-17-like	GI-H
MELO3C015502.2	0.892	1.551	4.364	1.679	1.499	14.940	1.036	Integral membrane protein	GI-H
MELO3C015505.2	0.512	1.042	9.156	1.253	0.345	2.980	0.403	vacuolar protein sorting-associated protein 2 homolog 1	GI-H
MELO3C015510.2	0.675	1.148	3.797	0.686	0.947	2.487	0.629	protein ELF4-LIKE 4	GI-H
MELO3C015512.2	2.143	2.814	6.785	1.012	3.266	1.773	3.154	protein CTR9 homolog	GI-H
MELO3C015563.2	2.130	1.994	5.592	1.904	2.556	2.748	1.910	TATA-box-binding protein	GI-H
MELO3C015565.2	0.187	0.485	1.032	0.283	0.354	0.248	0.180	Gamma-tubulin complex component	GI-H
MELO3C015579.2	1.400	1.722	4.635	NA	1.782	0.387	0.968	At1g55340	GI-H
MELO3C029305.2	1.497	1.498	4.749	1.080	1.273	1.195	1.111	elongation factor P	GI-H
MELO3C015607.2	7.372	26.162	52.863	19.005	19.844	25.741	10.679	Cysteine proteinase inhibitor	GI-H
MELO3C015611.2	2.475	3.799	5.431	4.490	1.961	4.788	2.543	ABSCISIC ACID-INSENSITIVE 5-like protein 2	GI-H
MELO3C015612.2	1.943	3.024	6.341	1.996	2.874	1.711	2.597	stomatal closure-related actin-binding protein 1	GI-H
MELO3C015625.2	1.597	2.938	5.863	1.480	2.272	4.046	2.120	protein FAR1-RELATED SEQUENCE 3	GI-H
MELO3C015635.2	3.967	4.996	12.177	6.585	4.003	9.580	3.065	somatic embryogenesis receptor kinase 1	GI-H
MELO3C015663.2	2.519	3.207	11.450	1.639	2.456	1.380	1.657	ribosome biogenesis regulatory protein homolog	GI-H
MELO3C015670.2	0.511	0.587	1.988	0.575	0.411	0.900	0.568	Phosphatidylinositol N-acetylglucosaminyltransferase subunit A	GI-H
MELO3C015682.2	0.993	1.945	6.756	0.977	0.730	0.676	0.844	Zinc finger BED domain-containing protein DAYSLEEPER	GI-H
MELO3C015221.2	0.384	0.997	15.403	0.979	1.090	NA	0.745	cyanogenic beta-glucosidase-like	GI-H
MELO3C015216.2	7.023	9.555	49.876	10.475	9.100	1.369	2.552	beta-glucosidase 12	GI-H
MELO3C015207.2	0.413	0.491	3.777	0.586	0.578	0.372	0.583	transcription factor bHLH122 isoform X1	GI-H
MELO3C029471.2	0.308	0.305	4.185	NA	0.632	0.190	0.299	E3 ubiquitin ligase BIG BROTHER-like	GI-H
MELO3C015193.2	1.960	2.438	4.666	2.262	1.295	7.816	1.249	Chaperonin-like RbcX protein 2, chloroplastic	GI-H
MELO3C015192.2	1.116	1.191	3.821	1.137	1.422	1.121	1.768	Armadillo repeat-containing protein 8	GI-H
MELO3C015185.2	1.300	1.596	38.250	0.443	2.986	NA	0.956	MP domain-containing protein	GI-H
MELO3C015184.2	2.388	4.284	22.289	NA	3.624	1.341	1.941	MP domain-containing protein	GI-H
MELO3C015176.2	0.657	0.943	3.401	0.705	1.000	0.718	0.834	NADH dehydrogenase ubiquinone complex I, assembly factor-like protein (DUF185)	GI-H
MELO3C015171.2	0.133	0.351	1.449	0.447	0.341	0.956	0.154	Ribonuclease I	GI-H
MELO3C015170.2	1.528	1.941	4.278	0.989	2.081	0.507	1.476	LOW QUALITY PROTEIN: THUMP domain-containing protein 1 homolog	GI-H
MELO3C015157.2	1.310	1.576	3.956	0.916	1.277	2.126	1.145	DDT domain-containing protein PTM	GI-H

Gene ID	FPKM							Gene Description	Specific in episode
	FS	GI-M	GM-M	AN-M	GI-H	GM-H	AN-H		
MELO3C029500.2	0.097	0.179	1.404	0.463	0.282	NA	0.280	phytochromobilin:ferredoxin oxidoreductase, chloroplastic	GI-H
MELO3C015133.2	0.514	0.546	2.560	0.384	0.596	0.619	0.637	Integrator complex subunit 3	GI-H
MELO3C015131.2	0.674	1.237	3.623	1.034	0.765	0.731	0.835	Pentatricopeptide repeat-containing family protein	GI-H
MELO3C015126.2	2.032	2.841	6.179	2.137	2.820	1.541	2.283	Signal recognition particle subunit SRP68	GI-H
MELO3C015109.2	0.569	0.764	2.259	0.294	0.701	0.454	0.279	Pentatricopeptide repeat-containing protein	GI-H
MELO3C015107.2	0.397	0.666	4.143	0.625	0.410	0.790	0.607	Protein CASP, putative	GI-H
MELO3C015103.2	1.194	1.062	3.304	0.793	1.357	0.886	1.277	Histone deacetylase	GI-H
MELO3C015101.2	0.319	0.412	2.090	0.061	0.821	0.081	0.600	RNA cytidine acetyltransferase 1-like	GI-H
MELO3C015086.2	0.707	0.781	2.200	0.655	0.976	0.593	0.909	3-hydroxyisobutyryl-CoA hydrolase-like protein	GI-H
MELO3C015043.2	0.445	0.917	2.858	0.539	0.534	0.784	0.346	DnaJ-like protein	GI-H
MELO3C015028.2	0.412	0.440	1.682	0.301	0.312	0.398	0.293	CWF19-like protein 2 homolog	GI-H
MELO3C015006.2	0.216	0.410	2.520	0.399	1.211	0.200	0.365	Unknown protein	GI-H
MELO3C015000.2	0.341	0.417	1.187	0.225	0.371	0.224	0.417	APO protein 3, mitochondrial isoform X2	GI-H
MELO3C029409.2	182.600	312.575	765.585	385.593	369.661	194.223	346.199	ATP synthase subunit a	GI-H
MELO3C009991.2	1.415	1.828	9.118	1.299	3.069	0.836	2.667	Protein IQ-DOMAIN 1-like protein	GI-H
MELO3C009998.2	1.656	1.130	6.591	0.548	2.872	NA	2.776	microtubule-associated protein TORTIFOLIA1 isoform X1	GI-H
MELO3C010012.2	0.558	0.764	2.359	0.494	0.659	0.661	0.527	Transmembrane protein	GI-H
MELO3C010033.2	0.290	0.474	1.529	0.191	0.365	0.393	0.288	Vacuolar protein sorting-associated protein, putative (DUF1162)	GI-H
MELO3C010072.2	0.362	0.302	1.239	0.282	0.503	0.163	0.380	Pentatricopeptide repeat-containing family protein	GI-H
MELO3C010074.2	0.277	0.480	1.179	0.345	0.476	0.713	0.256	Abc transporter, putative	GI-H
MELO3C029427.2	0.231	0.232	1.916	0.231	0.277	0.368	0.356	pentatricopeptide repeat-containing protein At4g32450, mitochondrial-like	GI-H
MELO3C010097.2	0.147	0.257	1.676	0.223	0.171	0.096	0.265	Plastid-lipid associated protein pap	GI-H
MELO3C010103.2	1.891	1.769	4.401	1.375	1.594	2.317	1.601	MAG2-interacting protein 2	GI-H
MELO3C010126.2	0.361	0.449	3.401	NA	0.549	0.235	0.404	Pentatricopeptide repeat-containing protein	GI-H
MELO3C010129.2	0.863	1.474	6.299	NA	2.023	0.092	0.986	Pentatricopeptide repeat-containing family protein	GI-H
MELO3C010145.2	0.361	0.366	2.536	0.274	0.436	0.089	0.481	ATP-dependent zinc metalloprotease FtsH	GI-H
MELO3C029630.2	0.549	0.395	1.776	0.234	0.776	0.436	0.607	Serine/threonine-protein kinase	GI-H
MELO3C010175.2	0.974	0.871	3.749	0.735	1.602	0.150	1.304	Pentatricopeptide repeat-containing family protein	GI-H
MELO3C010202.2	0.750	1.294	4.618	1.086	1.628	3.104	1.285	Pentatricopeptide repeat-containing protein	GI-H
MELO3C010205.2	1.549	2.116	5.269	1.734	2.385	3.302	2.319	Myosin-binding protein 7	GI-H

Gene ID	FPKM							Gene Description	Specific in episode
	FS	GI-M	GM-M	AN-M	GI-H	GM-H	AN-H		
MELO3C010213.2	0.788	1.207	5.790	1.011	0.868	1.525	0.779	Ribosomal RNA small subunit methyltransferase G	GI-H
MELO3C010214.2	0.365	0.368	6.935	0.831	0.269	0.379	0.767	Nucleoid-associated protein At2g24020, chloroplastic	GI-H
MELO3C010236.2	0.609	0.924	1.467	0.461	0.494	0.489	0.670	Unknown protein	GI-H
MELO3C010243.2	2.065	3.482	12.937	2.418	2.255	5.448	3.885	VAMP-like protein YKT61	GI-H
MELO3C010257.2	0.916	2.600	2.402	1.251	1.155	1.154	0.942	sugar transport protein 7	GI-H
MELO3C010260.2	1.827	2.836	7.172	2.187	2.063	5.088	1.246	G-patch domain-containing protein	GI-H
MELO3C010264.2	1.300	1.251	6.001	0.780	1.168	NA	0.157	Zinc/RING finger 3	GI-H
MELO3C010266.2	0.245	0.446	3.926	0.212	0.412	0.150	0.273	Pentatricopeptide repeat-containing family protein	GI-H
MELO3C010267.2	0.272	NA	10.165	NA	1.325	NA	0.797	Peptidyl-prolyl cis-trans isomerase cyp8	GI-H
MELO3C010277.2	0.805	1.277	3.832	1.572	1.413	1.779	1.400	Protein OS-9	GI-H
MELO3C010279.2	0.350	1.298	7.933	1.434	1.266	1.791	0.836	Octicosapeptide/Phox/Bem1p (PB1) domain-containing protein / tetratricopeptide repeat (TPR)-containing protein	GI-H
MELO3C029461.2	0.244	0.127	1.041	0.120	0.238	NA	0.460	Telomere-associated protein RIF1, putative	GI-H
MELO3C010327.2	0.478	0.932	9.213	0.422	1.016	1.096	0.658	Transposon protein, putative, CACTA, En/Spm sub-class	GI-H
MELO3C010336.2	1.450	1.450	3.406	0.849	1.233	6.713	0.982	Ribosomal protein	GI-H
MELO3C010343.2	2.257	1.329	11.043	1.976	0.838	1.822	1.024	SPX domain-containing protein 1	GI-H
MELO3C010355.2	4.387	5.408	9.726	4.522	4.715	1.353	4.857	H/ACA ribonucleoprotein complex, subunit Nhp2, eukaryote	GI-H
MELO3C010358.2	9.043	11.434	52.449	6.061	14.729	1.549	10.712	Alba DNA/RNA-binding protein	GI-H
MELO3C010361.2	1.177	1.682	3.831	2.675	1.137	4.398	1.557	Transmembrane protein, putative	GI-H
MELO3C026635.2	0.391	0.531	1.999	1.823	0.565	6.324	0.298	Plant Tudor-like RNA-binding protein	GI-H
MELO3C026626.2	0.426	0.554	2.031	NA	0.638	0.303	0.629	Pentatricopeptide repeat-containing protein	GI-H
MELO3C025302.2	0.650	0.616	1.712	0.641	0.461	0.589	0.590	Catalytic/ hydrolase	GI-H
MELO3C025337.2	1.914	1.625	8.951	1.335	2.465	0.271	1.558	Kinesin-like protein	GI-H
MELO3C029692.2	2.142	2.324	7.671	0.956	2.469	0.305	2.696	Transcriptional corepressor LEUNIG	GI-H
MELO3C025352.2	1.765	1.745	8.901	0.596	2.203	2.446	1.526	Pentatricopeptide repeat-containing protein	GI-H
MELO3C025355.2	0.948	1.344	7.921	0.626	0.835	0.692	0.684	vacuolar-processing enzyme-like	GI-H
MELO3C024632.2	0.640	0.745	4.841	0.319	1.112	0.606	0.844	Proteinaceous RNase P 1, chloroplastic/mitochondrial	GI-H
MELO3C024656.2	1.654	2.427	14.868	0.971	2.127	0.654	2.277	Protein WVD2-like 1	GI-H
MELO3C024660.2	0.439	0.331	6.153	0.203	0.543	NA	0.457	Auxin-responsive protein	GI-H
MELO3C024666.2	0.504	0.759	2.567	0.762	1.006	0.520	0.911	Peptidase C13 family	GI-H
MELO3C024669.2	0.391	0.464	1.869	0.295	0.604	0.072	0.634	Pseudouridine synthase family protein	GI-H

Gene ID	FPKM							Gene Description	Specific in episode
	FS	GI-M	GM-M	AN-M	GI-H	GM-H	AN-H		
MELO3C024673.2	0.203	0.286	9.512	0.777	1.069	0.119	0.710	Trihelix transcription factor	GI-H
MELO3C024678.2	0.077	0.344	2.904	0.629	0.324	0.291	0.360	Kinase, putative	GI-H
MELO3C024679.2	0.398	0.288	1.050	0.235	0.315	0.528	0.268	Myelin-associated oligodendrocyte basic protein isoform 1	GI-H
MELO3C024683.2	0.530	0.814	2.943	0.584	1.279	NA	0.902	Pentatricopeptide repeat-containing family protein	GI-H
MELO3C024684.2	7.253	8.350	91.029	9.829	10.744	2.625	8.392	40S ribosomal protein S24	GI-H
MELO3C029708.2	0.423	0.362	1.403	0.244	0.540	0.381	0.401	Unknown protein	GI-H
MELO3C024696.2	1.003	2.409	7.104	1.802	2.590	9.906	1.879	transcription factor bHLH68 isoform X1	GI-H
MELO3C024701.2	0.969	1.541	6.537	1.126	2.022	0.686	1.834	Elongation factor Ts, mitochondrial	GI-H
MELO3C024707.2	1.028	0.983	5.089	1.014	1.754	1.452	1.402	7SK snRNA methylphosphate capping enzyme	GI-H
MELO3C024715.2	0.557	1.200	2.959	0.326	1.204	NA	0.893	Exostosin family protein	GI-H
MELO3C026990.2	0.130	0.280	1.197	0.196	0.179	0.175	0.266	Exostosin family protein	GI-H
MELO3C017522.2	0.950	0.851	2.584	0.452	0.938	0.559	0.976	Cell division cycle 5-like protein	GI-H
MELO3C017458.2	2.928	2.865	9.403	1.019	4.570	1.077	4.098	replication protein A 32 kDa subunit B-like	GI-H
MELO3C017455.2	0.553	0.849	4.743	0.663	0.558	5.222	0.606	VIN3-like protein 2	GI-H
MELO3C017440.2	0.437	0.674	3.339	0.329	0.835	0.191	0.710	U3 small nucleolar RNA-associated protein 25-like	GI-H
MELO3C017439.2	1.619	2.317	9.045	1.434	3.582	0.556	2.811	Mitochondrial outer membrane import complex protein metaxin	GI-H
MELO3C017438.2	2.175	5.079	7.578	4.362	2.908	11.709	2.723	cadmium/zinc-transporting ATPase HMA3-like	GI-H
MELO3C017437.2	1.174	1.331	5.104	1.098	1.137	0.376	1.239	Core-2/I-branching beta-16-N-acetylglucosaminyltransferase family protein	GI-H
MELO3C017430.2	2.333	5.404	17.039	8.661	4.457	6.102	3.737	Fiber protein Fb15	GI-H
MELO3C017429.2	0.319	0.270	2.713	0.190	0.348	NA	0.332	pathogenesis-related homeodomain protein	GI-H
MELO3C017428.2	0.397	0.653	2.234	0.486	0.840	0.564	0.676	RING/FYVE/PHD zinc finger superfamily protein, putative isoform 1	GI-H
MELO3C017424.2	0.266	0.180	1.904	0.156	0.416	0.463	0.114	transcription factor bHLH35	GI-H
MELO3C017420.2	0.775	1.014	3.618	0.893	1.194	0.134	1.131	protein SMAX1-LIKE 4-like	GI-H
MELO3C017411.2	3.406	7.668	9.059	5.999	3.381	2.515	3.361	glutaredoxin-C4-like	GI-H
MELO3C017409.2	0.488	0.413	1.195	0.289	0.375	0.205	0.396	Lysine-specific demethylase NO66	GI-H
MELO3C017388.2	1.314	2.381	9.866	3.021	0.945	2.997	2.403	Neuronal acetylcholine receptor subunit alpha-5	GI-H
MELO3C017381.2	1.044	1.084	4.546	0.493	1.391	0.497	0.973	LOW QUALITY PROTEIN: serine/threonine-protein kinase TOUSLED	GI-H
MELO3C017377.2	1.119	0.956	6.962	0.573	3.200	NA	2.580	phragmoplast orienting kinesin 2	GI-H
MELO3C029540.2	30.829	37.618	150.630	62.104	29.147	28.749	18.381	Unknown protein	GI-H

Gene ID	FPKM							Gene Description	Specific in episode
	FS	GI-M	GM-M	AN-M	GI-H	GM-H	AN-H		
MELO3C017354.2	0.478	0.599	2.776	NA	0.518	0.237	0.628	Pentatricopeptide repeat-containing protein	GI-H
MELO3C017351.2	0.413	0.779	3.260	0.331	0.529	0.419	0.538	golgin candidate 2	GI-H
MELO3C029742.2	0.182	0.269	1.335	0.178	0.218	NA	0.328	Pentatricopeptide repeat-containing protein	GI-H
MELO3C017340.2	0.554	0.988	2.037	0.590	0.910	0.114	0.667	PAP fibrillin domain proteinexpressed protein	GI-H
MELO3C017339.2	2.877	2.904	6.104	2.742	2.459	3.163	2.244	peroxisome biogenesis protein 3-2	GI-H
MELO3C017325.2	1.056	0.978	5.117	0.564	0.841	0.326	1.007	Ribosomal protein S24/S35, mitochondrial	GI-H
MELO3C017320.2	3.279	3.652	8.941	2.452	3.414	7.507	3.761	Zinc finger protein, putative	GI-H
MELO3C017319.2	0.378	0.574	1.844	1.482	0.329	3.180	0.210	BnaA06g03540D protein	GI-H
MELO3C017303.2	0.534	0.697	3.044	0.636	0.773	0.388	0.795	Carbon catabolite repressor protein 4 like 5	GI-H
MELO3C017293.2	0.479	0.575	1.691	0.505	0.631	0.165	0.487	ATP-dependent helicase/nuclease subunit A isoform 1	GI-H
MELO3C017291.2	5.481	3.689	31.791	3.781	4.719	2.154	5.205	40S ribosomal protein S28	GI-H
MELO3C017285.2	0.548	1.599	3.978	1.720	1.387	2.731	0.816	Transcription initiation factor IIE alpha subunit family protein	GI-H
MELO3C017278.2	1.220	0.660	4.608	NA	1.022	0.169	1.275	Kinesin-like protein	GI-H
MELO3C017274.2	0.612	0.780	3.652	0.953	0.836	0.771	0.769	G patch domain-containing protein TGH	GI-H
MELO3C017265.2	0.702	1.377	4.377	0.883	0.878	0.637	0.713	Uridine-cytidine kinase C	GI-H
MELO3C017259.2	0.972	2.336	6.822	1.400	1.118	NA	1.082	Protoheme IX farnesyltransferase, mitochondrial	GI-H
MELO3C017258.2	1.442	1.226	8.479	0.881	3.853	0.246	3.704	WD repeat and HMG-box DNA-binding protein 1 isoform X1	GI-H
MELO3C017257.2	1.017	1.866	4.375	3.909	1.756	2.921	1.621	transcription factor BIM1	GI-H
MELO3C017253.2	5.222	6.823	10.852	7.208	5.008	12.648	4.621	dof zinc finger protein DOF5.4	GI-H
MELO3C017236.2	1.254	0.909	4.217	NA	1.335	0.272	1.652	Host cell factor	GI-H
MELO3C017235.2	2.400	2.270	4.862	1.595	1.833	3.298	2.138	Zinc finger CCCH domain-containing protein 55	GI-H
MELO3C017234.2	1.121	1.018	4.838	1.777	1.550	1.093	1.524	Zinc finger CCCH domain-containing protein 55	GI-H
MELO3C017233.2	0.307	0.484	5.024	0.683	0.618	0.718	0.592	Haloacid dehalogenase-like hydrolase (HAD) superfamily protein	GI-H
MELO3C017231.2	0.687	0.694	2.458	0.366	0.255	0.215	0.324	protein LONGIFOLIA 2	GI-H
MELO3C029752.2	0.759	0.752	2.889	0.691	0.816	0.371	0.555	Unknown protein	GI-H
MELO3C017228.2	4.123	2.122	19.375	3.299	5.621	0.149	6.317	Replication protein A 70 kDa DNA-binding subunit	GI-H
MELO3C017216.2	0.531	0.424	2.135	0.399	0.448	0.224	0.565	ATP-dependent RNA helicase	GI-H
MELO3C017214.2	0.297	1.331	3.344	1.159	0.785	0.997	0.558	N-acetylglucosaminyl-phosphatidylinositol de-n-acetylase, putative	GI-H
MELO3C017211.2	3.487	3.861	10.316	2.639	4.293	5.783	4.103	AT-rich interactive domain-containing 2-like protein	GI-H
MELO3C017205.2	1.209	1.271	3.179	0.560	0.952	1.060	0.808	S phase cyclin A-associated in the endoplasmic reticulum	GI-H

Gene ID	FPKM							Gene Description	Specific in episode
	FS	GI-M	GM-M	AN-M	GI-H	GM-H	AN-H		
MELO3C017204.2	0.664	0.516	2.849	0.497	1.129	0.596	1.194	DNA annealing helicase and endonuclease ZRANB3 isoform X1	GI-H
MELO3C017199.2	0.585	0.487	1.825	NA	0.407	0.506	0.351	WEB family protein At5g55860	GI-H
MELO3C017165.2	2.769	6.021	13.861	4.662	5.631	4.104	3.787	HVA22-like protein	GI-H
MELO3C017144.2	2.718	2.106	9.000	2.211	3.297	2.731	3.457	DDT domain-containing protein DDR4	GI-H
MELO3C017137.2	0.863	1.843	8.845	3.044	2.318	2.741	1.680	5-formyltetrahydrofolate cyclo-ligase	GI-H
MELO3C017127.2	0.434	0.849	4.147	NA	1.144	NA	1.166	E2F transcription factor-like E2FE	GI-H
MELO3C017116.2	0.365	0.768	6.414	0.236	0.985	3.109	0.717	Kinesin-like protein	GI-H
MELO3C017113.2	0.857	0.830	13.081	1.247	1.126	1.181	0.680	Serine/threonine-protein kinase TAO3	GI-H
MELO3C017109.2	1.869	3.614	9.511	3.122	1.963	1.110	1.963	Rac-like GTP-binding protein	GI-H
MELO3C017106.2	2.968	1.790	7.564	1.267	2.147	0.371	2.194	RNA-dependent RNA polymerase	GI-H
MELO3C026281.2	1.182	0.820	5.744	0.593	1.404	0.364	1.462	Carboxyl-terminal peptidase, putative (DUF239)	GI-H
MELO3C026277.2	0.708	0.708	4.911	NA	0.810	0.315	0.441	Kinetochore protein	GI-H
MELO3C026222.2	4.978	5.357	11.790	5.250	3.598	6.100	4.639	Transcription factor GTE10	GI-H
MELO3C026211.2	2.972	3.447	9.505	2.754	2.226	1.558	3.087	SAP domain-containing protein	GI-H
MELO3C026200.2	2.423	3.022	5.323	3.446	2.272	7.260	2.615	CBS domain-containing protein	GI-H
MELO3C026196.2	1.233	0.711	9.066	0.773	2.629	NA	1.534	squamosa promoter-binding-like protein 13A	GI-H
MELO3C026190.2	2.293	1.996	12.453	1.006	2.492	1.805	1.438	SWI/SNF complex subunit SWI3A	GI-H
MELO3C026189.2	1.040	1.097	13.403	0.831	1.813	0.798	1.661	Protein DEK, putative	GI-H
MELO3C029774.2	0.926	1.013	3.608	1.184	1.593	1.029	1.287	Copia protein	GI-H
MELO3C008069.2	0.864	1.316	5.220	1.134	1.382	1.652	0.909	Phosphoinositide phosphatase family protein	GI-H
MELO3C008073.2	0.300	0.240	1.235	0.112	0.353	0.108	0.281	Fanconi anemia group M protein isoform X1	GI-H
MELO3C008096.2	0.451	0.718	3.391	0.475	0.545	3.305	0.391	Galactose-binding domain-like protein	GI-H
MELO3C008109.2	0.931	1.381	3.516	1.242	1.375	1.195	1.226	Vacuolar protein sorting-associated protein 52 A	GI-H
MELO3C008118.2	0.664	0.747	5.542	1.358	1.075	1.151	1.167	Inner membrane protein oxaA	GI-H
MELO3C008127.2	1.006	2.038	3.927	1.692	1.806	2.795	1.623	Disulfide isomerase-like protein	GI-H
MELO3C008133.2	0.406	0.421	1.311	0.282	0.489	NA	0.189	Endoribonuclease E-like protein	GI-H
MELO3C008134.2	0.431	0.391	1.924	0.366	0.478	2.583	0.275	Glutaredoxin domain-containing protein/DEP domain-containing protein/DUF547 domain-containing protein	GI-H
MELO3C008138.2	0.993	0.690	2.731	0.479	1.000	1.110	1.057	ENTH/VHS family protein	GI-H
MELO3C029822.2	0.925	1.154	3.653	0.769	1.061	1.344	0.446	serine/threonine-protein kinase SAPK3-like	GI-H
MELO3C008158.2	0.929	1.279	3.173	NA	0.502	0.701	0.439	Protein PLASTID MOVEMENT IMPAIRED 2	GI-H

Gene ID	FPKM							Gene Description	Specific in episode
	FS	GI-M	GM-M	AN-M	GI-H	GM-H	AN-H		
MELO3C008200.2	0.655	0.785	2.565	0.339	0.682	0.201	0.805	Nucleolar complex protein 2 homolog	GI-H
MELO3C008202.2	1.226	2.250	5.161	1.005	1.547	5.052	1.469	Ribulose-phosphate 3-epimerase	GI-H
MELO3C008208.2	0.773	0.993	3.271	0.779	0.815	0.358	0.957	Mitochondrial protoporphyrinogen oxidase	GI-H
MELO3C008210.2	0.454	1.225	9.577	0.494	0.888	NA	0.978	ribosome-recycling factor	GI-H
MELO3C008211.2	0.361	0.532	4.962	NA	0.401	NA	0.185	sister chromatid cohesion 1 protein 2 isoform X1	GI-H
MELO3C008212.2	0.576	0.650	5.220	NA	1.205	NA	0.929	Actin-related protein 2/3 complex subunit 5	GI-H
MELO3C008213.2	0.830	0.889	7.256	1.735	1.250	0.608	0.730	COP9 signalosome complex subunit 3	GI-H
MELO3C008218.2	2.077	4.459	9.552	4.691	3.371	25.287	2.005	Plant UBX domain-containing protein 1	GI-H
MELO3C008221.2	5.261	5.274	13.990	6.636	5.400	10.247	4.183	serine/threonine protein phosphatase 2A 57 kDa regulatory subunit B' theta isoform-like	GI-H
MELO3C008230.2	1.048	0.855	4.440	0.874	1.606	0.531	1.133	HAT transposon superfamily, putative	GI-H
MELO3C008270.2	1.814	1.469	4.538	2.970	1.358	2.423	2.110	syntaxin-81	GI-H
MELO3C008289.2	0.384	1.033	5.969	0.633	0.979	2.006	0.912	Protein yippee-like	GI-H
MELO3C008293.2	0.726	0.962	2.723	0.603	1.016	1.245	1.026	Receptor-kinase, putative	GI-H
MELO3C029909.2	3.528	4.011	24.021	6.693	6.460	0.884	5.763	Unknown protein	GI-H
MELO3C029829.2	47.729	67.548	109.658	52.145	48.073	37.684	36.522	Photosystem I iron-sulfur center	GI-H
MELO3C008318.2	0.962	1.260	3.272	NA	1.054	2.716	0.308	Dehydration-responsive element-binding protein 2C	GI-H
MELO3C008329.2	1.419	1.426	6.034	0.575	0.983	1.662	1.239	Histone-lysine N-methyltransferase	GI-H
MELO3C008331.2	0.328	0.913	1.885	5.029	0.268	83.500	0.513	ethylene-responsive transcription factor ERF113-like	GI-H
MELO3C008344.2	2.972	6.172	23.482	3.896	6.747	2.813	4.877	Small nuclear ribonucleoprotein	GI-H
MELO3C008345.2	0.652	0.669	5.750	NA	0.611	0.267	0.699	Protein ROOT PRIMORDIUM DEFECTIVE 1	GI-H
MELO3C008375.2	7.348	11.328	40.497	NA	13.709	NA	16.508	Unknown protein	GI-H
MELO3C029952.2	1.181	1.623	3.942	1.462	1.079	0.410	1.514	Alanine--tRNA ligase	GI-H
MELO3C029848.2	0.580	0.938	3.459	1.025	0.527	0.611	0.887	Unknown protein	GI-H
MELO3C008404.2	0.470	0.700	1.663	0.208	0.459	0.515	0.520	Pathogen-related protein	GI-H
MELO3C030001.2	0.384	0.606	2.189	0.556	0.834	0.338	0.692	Unknown protein	GI-H
MELO3C008440.2	0.132	0.418	1.168	0.309	0.241	0.160	0.062	wall-associated receptor kinase 2-like	GI-H
MELO3C010658.2	0.220	0.383	2.612	0.173	0.540	0.258	0.339	Pentatricopeptide repeat-containing family protein	GI-H
MELO3C010653.2	0.394	0.450	1.889	0.302	0.385	0.348	0.384	Wound-responsive family protein	GI-H
MELO3C010640.2	1.451	0.986	5.017	1.474	1.458	0.614	1.033	zinc finger CCCH domain-containing protein 1	GI-H
MELO3C010633.2	3.784	6.512	10.827	4.837	4.153	2.747	3.797	Thioredoxin domain-containing protein, putative	GI-H

Gene ID	FPKM							Gene Description	Specific in episode
	FS	GI-M	GM-M	AN-M	GI-H	GM-H	AN-H		
MELO3C029885.2	2.898	NA	28.771	0.884	1.048	NA	1.178	NAD(P)H-quinone oxidoreductase subunit I, chloroplastic	GI-H
MELO3C030091.2	1.500	2.004	23.444	4.676	2.823	1.055	1.961	Unknown protein	GI-H
MELO3C010611.2	1.183	1.088	8.538	0.567	1.763	0.391	1.686	Filament-like plant protein	GI-H
MELO3C010600.2	0.322	0.136	3.682	0.118	0.223	0.064	0.162	DNA ligase 1 isoform X3	GI-H
MELO3C010599.2	1.546	2.408	8.210	2.884	1.055	2.080	1.700	Gamma-glutamylcyclotransferase	GI-H
MELO3C010585.2	1.900	1.780	4.932	1.682	1.993	2.360	2.080	Transcriptional corepressor SEUSS	GI-H
MELO3C010584.2	0.818	1.563	10.993	1.464	1.360	2.271	1.735	ribosome maturation protein SBDS	GI-H
MELO3C010582.2	0.599	0.703	1.947	0.488	0.874	0.709	0.585	small RNA degrading nuclease 5	GI-H
MELO3C011705.2	2.636	12.691	13.256	3.873	2.717	7.372	1.819	Pyruvate dehydrogenase E1 component subunit alpha	GI-H
MELO3C011660.2	0.357	0.514	1.113	0.279	0.393	0.683	0.451	eIF-2-alpha kinase GCN2 isoform X1	GI-H
MELO3C011647.2	0.762	0.829	2.616	0.501	0.780	0.744	0.650	4'-phosphopantetheinyl transferase	GI-H
MELO3C011646.2	0.133	0.090	1.113	0.078	0.170	0.065	0.193	BnaC08g44510D protein	GI-H
MELO3C011610.2	10.541	10.477	60.704	8.581	26.877	2.361	20.885	Histone H3	GI-H
MELO3C011578.2	2.014	2.920	6.748	1.873	2.454	2.464	2.445	DIS3-like exonuclease 2	GI-H
MELO3C030176.2	0.672	2.585	14.286	2.184	2.522	2.392	1.213	Beta-glucosidase	GI-H
MELO3C024977.2	0.120	0.461	1.483	0.661	0.224	NA	0.680	Protein LURP-one-related 7	GI-H
MELO3C025023.2	0.260	0.923	2.526	1.822	0.965	0.864	0.572	thioredoxin-like protein slr0233	GI-H
MELO3C025039.2	1.447	1.210	4.095	1.027	1.971	0.461	1.977	Plastid division protein CDP1, chloroplastic	GI-H
MELO3C025043.2	0.570	0.771	2.035	0.378	0.487	0.226	0.726	programmed cell death protein 2-like	GI-H
MELO3C020022.2	1.417	1.729	6.967	2.696	1.298	1.128	1.065	DNA-directed RNA polymerase II subunit 4	GI-H
MELO3C020021.2	1.512	3.013	7.190	1.352	2.097	2.775	1.544	Extra-large G-like protein, putative (DUF3133)	GI-H
MELO3C020016.2	0.506	0.609	1.887	0.362	0.614	0.426	0.530	E3 ubiquitin-protein ligase SHPRH isoform X1	GI-H
MELO3C020013.2	4.959	7.394	13.008	8.567	4.346	11.822	4.297	Ubiquinol-cytochrome c reductase complex 6.7 kDa protein	GI-H
MELO3C019999.2	4.726	4.647	11.233	3.791	4.251	1.945	5.279	WEB family protein At2g40480	GI-H
MELO3C019997.2	5.860	6.971	44.534	6.280	12.316	7.145	9.510	Histone H4	GI-H
MELO3C019996.2	5.237	5.335	26.427	4.798	6.177	6.570	10.043	Histone H4	GI-H
MELO3C019988.2	0.951	0.708	18.538	1.284	1.462	0.827	1.029	Alpha-soluble NSF attachment protein	GI-H
MELO3C019982.2	0.433	0.566	5.002	NA	0.365	1.045	0.555	heavy metal-associated isoprenylated plant protein 20-like	GI-H
MELO3C019974.2	1.150	1.762	2.910	1.408	0.848	2.447	0.519	Type I inositol polyphosphate 5-phosphatase, putative	GI-H
MELO3C019971.2	0.288	0.899	5.170	0.305	1.229	0.145	0.860	30S ribosomal protein S1	GI-H
MELO3C019945.2	1.119	0.987	3.751	1.107	1.105	1.632	1.042	exocyst complex component SEC6	GI-H

Gene ID	FPKM							Gene Description	Specific in episode
	FS	GI-M	GM-M	AN-M	GI-H	GM-H	AN-H		
MELO3C019933.2	1.091	1.404	3.894	0.840	1.112	0.449	0.989	HAT transposon superfamily	GI-H
MELO3C030259.2	0.495	0.384	2.289	0.187	0.171	0.257	0.311	exocyst complex component SEC6	GI-H
MELO3C019922.2	1.611	1.246	6.333	1.199	1.861	NA	2.148	NUFIP	GI-H
MELO3C019919.2	1.684	1.989	9.404	2.234	3.352	0.063	3.617	Protein kinase	GI-H
MELO3C019911.2	0.905	0.665	7.678	0.677	1.130	0.120	1.064	protein SMAX1-LIKE 3-like	GI-H
MELO3C019899.2	1.472	1.612	3.615	2.400	1.782	3.172	1.636	AMMECR1 family	GI-H
MELO3C019898.2	1.062	5.432	9.726	3.332	4.109	3.875	2.726	Vesicle-associated membrane protein, putative	GI-H
MELO3C019896.2	27.432	40.492	88.237	23.581	36.036	15.933	34.256	40S ribosomal protein S25, putative	GI-H
MELO3C019889.2	1.978	2.609	6.336	1.867	2.279	4.339	2.032	Protein SUPPRESSOR OF GENE SILENCING 3	GI-H
MELO3C019887.2	1.050	2.426	6.511	1.091	1.948	0.987	1.137	Protein BUD31-like protein 1	GI-H
MELO3C019857.2	0.896	1.513	10.484	1.029	2.430	0.370	1.256	60S ribosomal protein L24	GI-H
MELO3C019853.2	1.142	0.800	6.772	0.735	1.273	NA	1.235	Plastid envelope DNA binding protein	GI-H
MELO3C019844.2	0.168	0.214	1.888	NA	0.515	NA	0.546	ras-related protein RABC2a-like	GI-H
MELO3C019842.2	0.964	0.800	9.131	0.391	3.233	1.644	2.404	Enabled-like protein (DUF1635)	GI-H
MELO3C019841.2	0.447	NA	13.441	NA	0.966	NA	0.501	shugoshin-1 isoform X1	GI-H
MELO3C019833.2	0.616	1.081	2.708	0.473	1.282	NA	1.312	Glycine-rich RNA-binding family protein	GI-H
MELO3C019827.2	0.347	0.908	2.869	0.597	1.233	1.013	0.509	MEF2BNB-like protein	GI-H
MELO3C019823.2	1.035	1.279	10.578	0.924	1.768	0.270	1.476	Kinesin-like protein	GI-H
MELO3C030280.2	0.935	NA	6.797	2.040	1.914	NA	0.552	Unknown protein	GI-H
MELO3C019800.2	1.778	2.231	18.312	3.510	2.487	6.914	3.276	Ubiquitin-like protein 5	GI-H
MELO3C019797.2	1.100	1.817	6.511	1.199	1.805	0.434	0.718	mRNA-decapping enzyme subunit 2-like	GI-H
MELO3C026498.2	1.987	4.646	7.187	5.115	2.145	21.173	1.187	Plant/protein	GI-H
MELO3C026504.2	0.608	0.651	1.545	0.660	0.471	0.508	0.611	centromere protein V isoform X1	GI-H
MELO3C026511.2	2.101	2.985	6.117	1.525	2.277	2.032	2.366	histone-lysine N-methyltransferase ATXR7	GI-H
MELO3C026520.2	0.927	1.186	4.748	0.696	1.701	0.805	1.456	Increased DNA methylation 3	GI-H
MELO3C026523.2	2.639	2.575	7.145	3.178	3.479	1.411	2.342	outer envelope pore protein 21, chloroplastic	GI-H
MELO3C026530.2	0.485	0.737	4.465	0.918	0.570	9.395	0.368	Pentatricopeptide repeat-containing protein	GI-H
MELO3C026533.2	2.855	2.461	8.225	3.178	2.804	1.303	2.536	50S ribosomal protein l21 mitochondrial	GI-H
MELO3C026538.2	0.514	0.752	4.772	0.634	0.570	1.289	1.099	protein DELETION OF SUV3 SUPPRESSOR 1(I)-like	GI-H
MELO3C026542.2	1.178	0.671	4.039	0.864	0.996	0.649	0.716	TSA: Wollemia nobilis Ref_Wollemi_Transcript_18968_1826 transcribed RNA sequence	GI-H

Gene ID	FPKM							Gene Description	Specific in episode
	FS	GI-M	GM-M	AN-M	GI-H	GM-H	AN-H		
MELO3C026550.2	3.447	7.027	8.528	13.718	3.108	36.919	3.188	phosphoprotein ECPP44-like	GI-H
MELO3C026555.2	0.824	1.668	4.778	1.202	2.083	1.510	1.436	Histone-lysine N-methyltransferase ATX2	GI-H
MELO3C011490.2	1.893	1.868	5.494	0.898	2.474	1.088	2.172	Myosin	GI-H
MELO3C011486.2	1.701	2.417	4.795	1.734	1.659	1.994	1.305	DWNN domain, a CCHC-type zinc finger-like protein	GI-H
MELO3C011474.2	4.278	5.283	52.672	1.713	3.194	0.359	0.983	Ankyrin repeat family protein	GI-H
MELO3C011459.2	0.809	1.256	3.982	0.786	1.814	0.784	1.043	Transcription and mRNA export factor SUS1	GI-H
MELO3C011444.2	1.579	2.009	5.589	1.425	1.917	2.907	1.638	Mitogen-activated protein kinase	GI-H
MELO3C011443.2	0.170	0.574	1.084	0.598	0.319	NA	0.376	Glycosyltransferase	GI-H
MELO3C011434.2	0.873	2.003	5.975	0.839	1.163	3.035	1.045	F-box only protein 21	GI-H
MELO3C011433.2	1.493	2.894	9.303	2.239	2.874	4.094	2.378	DNA-directed RNA polymerase subunit beta	GI-H
MELO3C030290.2	0.388	0.230	2.213	0.531	0.414	1.260	0.325	Unknown protein	GI-H
MELO3C011425.2	1.458	1.553	4.819	2.429	1.602	3.833	1.518	erlin-2-B	GI-H
MELO3C011406.2	0.105	0.230	2.174	0.206	0.224	0.530	0.312	Chaperone DnaJ-domain superfamily protein	GI-H
MELO3C011403.2	0.712	0.854	8.800	0.586	0.971	NA	1.042	Translin family protein	GI-H
MELO3C011401.2	0.341	0.784	6.964	0.904	1.200	2.019	0.710	18S pre-ribosomal assembly protein gar2-like protein	GI-H
MELO3C011395.2	0.530	1.787	4.755	2.090	0.915	0.862	0.668	Hydroxyproline O-arabinosyltransferase 1	GI-H
MELO3C011393.2	0.670	0.389	2.031	0.408	0.279	NA	0.990	thioredoxin-like protein CITRX, chloroplastic	GI-H
MELO3C011385.2	1.092	1.216	2.784	0.905	1.384	1.453	1.293	vacuolar protein sorting-associated protein 36	GI-H
MELO3C011383.2	1.292	1.132	4.724	0.757	1.753	0.500	1.920	DNA-directed RNA polymerase subunit beta	GI-H
MELO3C011373.2	0.358	0.706	2.530	0.932	0.338	0.765	0.718	protein GrpE	GI-H
MELO3C011359.2	0.523	0.645	3.377	0.939	0.304	0.590	0.634	At1g78110	GI-H
MELO3C011355.2	0.239	0.163	3.030	NA	0.542	0.171	0.467	DNA-directed RNA polymerase subunit	GI-H
MELO3C011321.2	1.068	0.958	6.077	0.652	1.144	0.776	1.492	Transcription initiation factor TFIID subunit 10	GI-H
MELO3C011313.2	0.582	1.073	5.397	NA	1.520	NA	1.536	Lariat debranching enzyme	GI-H
MELO3C011310.2	0.732	0.631	10.342	1.318	1.154	0.225	0.882	Ribosomal protein S19	GI-H
MELO3C011299.2	3.310	3.451	9.155	0.511	3.609	0.153	3.138	BTB/POZ domain-containing protein NPY1	GI-H
MELO3C011294.2	2.870	2.738	13.134	2.237	3.557	1.927	3.140	sister chromatid cohesion protein PDS5 homolog B-B-like	GI-H
MELO3C011263.2	1.400	3.242	4.554	0.454	1.365	0.665	0.448	EEIG1/EHBP1 protein amino-terminal domain protein	GI-H
MELO3C011245.2	0.590	0.129	2.830	NA	0.494	NA	0.072	UPF0481 protein At3g47200-like	GI-H
MELO3C011240.2	0.768	0.760	16.868	0.205	1.821	NA	1.388	UPF0481 protein At3g47200-like	GI-H
MELO3C011206.2	0.634	0.269	1.472	0.215	0.221	0.215	0.231	dentin sialophosphoprotein-like	GI-H

Gene ID	FPKM							Gene Description	Specific in episode
	FS	GI-M	GM-M	AN-M	GI-H	GM-H	AN-H		
MELO3C011203.2	2.071	1.643	5.311	0.723	1.818	1.127	1.927	Ubiquitin carboxyl-terminal hydrolase 26	GI-H
MELO3C011191.2	0.912	1.303	4.552	0.759	1.583	0.350	0.718	Centromere protein O	GI-H
MELO3C011189.2	1.003	1.515	3.715	1.535	0.936	5.507	0.792	E3 ubiquitin-protein ligase arkadia	GI-H
MELO3C011187.2	4.134	6.563	44.310	NA	1.490	5.863	0.285	Methionyl-tRNA synthetase	GI-H
MELO3C011186.2	4.276	4.479	9.199	4.647	3.081	1.387	3.674	Peptidyl-tRNA hydrolase II (PTH2) family protein	GI-H
MELO3C011177.2	1.674	5.059	6.684	1.944	2.891	1.282	3.155	Protein disulfide isomerase (PDI)-like protein	GI-H
MELO3C011165.2	1.075	2.521	5.186	1.412	1.256	4.774	0.999	Myosin heavy chain-related	GI-H
MELO3C011160.2	0.614	1.098	3.558	1.788	0.419	0.425	0.644	2-on-2 hemoglobin	GI-H
MELO3C011152.2	0.140	0.190	1.389	0.100	0.248	0.140	0.021	protein EMBRYONIC FLOWER 1-like isoform X1	GI-H
MELO3C030319.2	0.077	0.160	1.414	NA	0.059	0.048	0.190	Histidine-containing phosphotransfer protein, putative	GI-H
MELO3C011127.2	0.600	1.938	3.023	0.276	0.538	1.002	0.292	receptor-like protein kinase HSL1	GI-H
MELO3C011121.2	1.145	1.596	3.910	1.583	1.462	1.874	1.163	RNA polymerase sigma factor sigC	GI-H
MELO3C011110.2	2.641	3.416	26.575	2.170	6.939	0.545	5.768	transcription factor bHLH93-like	GI-H
MELO3C011106.2	0.519	1.715	6.964	0.685	1.589	0.642	1.758	Pentatricopeptide repeat-containing protein	GI-H
MELO3C011105.2	1.432	1.682	3.418	0.888	1.250	1.078	0.544	Chaperone protein dnaJ	GI-H
MELO3C011097.2	3.035	2.389	9.671	2.026	2.881	2.946	2.865	ubiquitin carboxyl-terminal hydrolase 16-like isoform X1	GI-H
MELO3C011095.2	0.630	5.408	10.150	NA	0.818	1.874	0.544	Carbonic anhydrase	GI-H
MELO3C011086.2	0.234	0.187	4.161	NA	0.111	NA	0.193	Kinesin-like protein	GI-H
MELO3C011081.2	1.635	2.334	5.469	2.064	1.188	0.924	1.617	double-stranded RNA-binding protein 1 isoform X2	GI-H
MELO3C011080.2	1.742	1.400	5.509	0.828	1.900	NA	1.727	VIN3-like protein 2	GI-H
MELO3C011076.2	1.475	2.190	3.692	1.591	1.508	1.544	1.378	Golgi-body localization protein domain isoform 1	GI-H
MELO3C011052.2	0.327	NA	7.067	0.217	1.425	0.054	1.185	transcription factor bHLH96	GI-H
MELO3C011047.2	0.373	0.594	2.239	NA	0.432	0.186	0.421	phosphatidylinositol-glycan biosynthesis class X protein	GI-H
MELO3C011038.2	1.981	2.826	5.235	1.917	2.096	5.109	1.487	F-box protein	GI-H
MELO3C011029.2	0.847	1.039	3.452	1.042	1.491	0.275	1.447	Melanoma-associated antigen G1	GI-H
MELO3C011028.2	3.258	5.046	9.363	3.457	3.716	3.832	4.603	14-3-3-like protein	GI-H
MELO3C011020.2	1.032	0.674	4.880	0.552	1.662	0.083	1.552	kinesin-like protein KIN-6 isoform X1	GI-H
MELO3C010974.2	6.294	9.857	16.199	6.859	5.050	6.015	7.174	thioredoxin H1-like	GI-H
MELO3C010944.2	1.258	2.606	3.941	4.033	0.867	6.540	1.303	5' nucleotidase family protein	GI-H
MELO3C010931.2	0.429	0.302	3.855	NA	0.280	0.160	0.380	Endoribonuclease Dicer-like protein	GI-H
MELO3C010923.2	1.606	3.341	6.382	1.577	2.023	3.009	0.986	NAC domain-containing protein 13-like	GI-H

Gene ID	FPKM							Gene Description	Specific in episode
	FS	GI-M	GM-M	AN-M	GI-H	GM-H	AN-H		
MELO3C010913.2	1.419	1.953	3.086	1.193	0.792	1.416	1.222	Protein DA1-related 1	GI-H
MELO3C010894.2	0.390	0.785	1.935	0.742	0.882	0.351	0.704	Thiol:disulfide interchange protein txlA-like protein	GI-H
MELO3C010877.2	0.129	0.388	1.873	0.332	0.330	0.578	0.271	Phosphatidate phosphatase PAH2	GI-H
MELO3C010874.2	0.977	2.099	7.352	6.741	1.639	7.090	0.946	calcium-binding protein-like	GI-H
MELO3C010866.2	4.085	9.037	19.076	15.702	3.882	16.171	3.000	Calcium-binding EF-hand family protein, putative	GI-H
MELO3C010860.2	1.415	1.386	6.593	1.275	1.755	1.360	1.659	thioredoxin-like 4, chloroplastic	GI-H
MELO3C010857.2	2.566	2.844	8.345	3.768	2.923	5.669	2.846	Protein kinase family protein	GI-H
MELO3C010850.2	3.106	5.187	6.959	2.517	1.976	3.941	2.905	ABSCISIC ACID-INSENSITIVE 5-like protein 5	GI-H
MELO3C010805.2	0.687	0.387	2.329	0.284	0.604	0.266	0.752	DAR GTPase 2, mitochondrial isoform X1	GI-H
MELO3C010804.2	2.558	2.446	11.357	0.809	3.410	0.218	2.518	Tetratricopeptide repeat protein 7A	GI-H
MELO3C010787.2	0.505	0.707	7.037	NA	1.080	NA	0.582	Kinesin-like protein	GI-H
MELO3C010780.2	1.191	1.603	4.046	0.852	1.363	2.845	1.045	Chaperone protein DnaJ	GI-H
MELO3C010744.2	1.888	1.952	11.439	1.610	4.007	2.262	4.244	CTD small phosphatase-like protein 2	GI-H
MELO3C010743.2	0.273	0.189	2.124	NA	0.145	0.053	0.060	HORMA domain-containing protein 1	GI-H
MELO3C010735.2	3.007	2.991	21.152	2.187	4.053	1.577	4.077	nucleolar GTP-binding protein 1-like	GI-H
MELO3C010720.2	1.848	4.432	12.482	2.060	3.528	3.323	2.983	serine/threonine protein phosphatase 2A 57 kDa regulatory subunit B' theta isoform-like	GI-H
MELO3C010716.2	2.848	3.998	11.461	1.994	5.030	2.210	4.603	Protein HHL1, chloroplastic	GI-H
MELO3C010709.2	1.481	1.916	7.639	0.736	1.852	1.745	0.689	telomere-associated protein RIF1-like isoform X2	GI-H
MELO3C010706.2	0.642	0.606	7.334	1.012	0.405	0.951	0.521	Pectate lyase	GI-H
MELO3C010699.2	1.814	1.091	4.153	0.491	1.536	0.143	1.501	Coilin	GI-H
MELO3C010694.2	0.371	0.221	1.323	NA	0.305	0.085	0.135	Protein phosphatase 2c, putative	GI-H
MELO3C010686.2	1.686	2.381	5.300	0.541	2.385	0.688	2.612	Alanine aminotransferase 2	GI-H
MELO3C010664.2	2.402	4.604	11.041	1.290	2.478	1.721	1.584	F28C11.19	GI-H
MELO3C010663.2	2.085	3.076	14.586	4.793	2.271	2.428	2.799	Nucleic acid-binding, OB-fold-like protein	GI-H
MELO3C010660.2	0.818	0.800	2.122	0.409	0.500	0.465	0.778	Reticulocyte-binding protein 2 a	GI-H
MELO3C003311.2	0.633	1.034	3.025	0.781	0.780	0.803	0.514	2-phosphoglycerate kinase	GI-H
MELO3C003323.2	0.635	0.920	7.556	0.745	0.806	0.783	0.832	Myosin heavy chain-like protein	GI-H
MELO3C003333.2	0.484	0.867	4.361	1.693	1.333	1.602	0.782	AT4g17010/dl4535w	GI-H
MELO3C003335.2	0.327	0.526	2.311	0.206	0.677	0.260	0.312	GATA transcription factor 26-like	GI-H
MELO3C003339.2	1.111	2.540	3.600	3.725	0.929	9.097	0.908	Calcineurin B-like protein	GI-H

Gene ID	FPKM							Gene Description	Specific in episode
	FS	GI-M	GM-M	AN-M	GI-H	GM-H	AN-H		
MELO3C003342.2	0.515	1.000	7.578	NA	0.682	0.497	0.506	60S ribosomal protein L37a	GI-H
MELO3C003343.2	0.266	0.203	1.580	NA	0.090	0.096	0.095	SNF2 domain-containing protein CLASSY 4-like	GI-H
MELO3C030347.2	0.840	1.446	6.254	1.193	1.608	0.268	1.733	ATP-dependent Clp protease proteolytic subunit	GI-H
MELO3C003369.2	1.290	2.446	7.362	3.173	3.411	2.533	3.086	Ras-related protein	GI-H
MELO3C003371.2	0.099	1.535	1.070	0.429	0.149	35.863	0.230	Expansin-like protein	GI-H
MELO3C003375.2	3.210	2.856	9.190	4.097	4.538	2.708	3.890	Two-component response regulator-like protein APRR2	GI-H
MELO3C003391.2	0.647	1.266	4.070	0.603	0.990	1.203	0.792	transcription initiation factor TFIID subunit 4b	GI-H
MELO3C003403.2	0.375	1.073	5.556	0.540	1.797	NA	0.465	Auxin responsive SAUR protein	GI-H
MELO3C003410.2	0.308	0.444	2.078	NA	0.280	0.334	0.141	At1g32630	GI-H
MELO3C003430.2	0.565	1.169	3.378	2.333	1.384	0.443	0.906	Valine--tRNA ligase	GI-H
MELO3C003436.2	0.686	0.458	1.591	0.281	0.455	0.495	0.372	Transcription factor, putative	GI-H
MELO3C003437.2	2.066	3.836	13.024	1.814	2.969	4.377	2.295	inactive poly [ADP-ribose] polymerase RCD1	GI-H
MELO3C003440.2	1.027	1.051	3.035	1.246	0.722	1.133	1.037	Glycerol-3-phosphate acyltransferase, chloroplastic	GI-H
MELO3C003457.2	0.405	0.722	2.366	0.227	0.602	0.655	0.531	protein EMBRYONIC FLOWER 1-like isoform X3	GI-H
MELO3C003478.2	1.101	1.847	5.478	1.233	2.196	1.087	1.951	Calcineurin-binding protein cabin-1	GI-H
MELO3C003480.2	1.234	1.204	3.799	0.984	1.114	0.745	1.584	Alpha/beta-Hydrolases superfamily protein, putative	GI-H
MELO3C003481.2	1.445	2.390	5.184	3.282	2.229	0.751	1.882	ergosterol biosynthetic protein 28	GI-H
MELO3C003490.2	4.179	8.177	13.121	6.476	6.316	6.637	5.511	Ubiquitin-fold modifier-conjugating enzyme 1	GI-H
MELO3C003509.2	0.807	0.782	3.350	0.349	1.193	0.155	1.092	Leucine zipper factor-related family protein	GI-H
MELO3C003512.2	1.162	1.506	3.302	1.144	1.266	1.295	1.206	BEACH domain-containing protein B	GI-H
MELO3C003519.2	0.489	0.364	2.265	0.311	0.663	0.259	0.641	geranylgeranyl transferase type-2 subunit alpha	GI-H
MELO3C003524.2	2.405	1.297	12.585	NA	3.700	0.157	2.592	kinesin-like protein KIN-4C	GI-H
MELO3C003534.2	0.507	0.599	3.446	1.110	0.338	0.409	0.342	Chaperone protein dnaJ	GI-H
MELO3C003550.2	1.302	1.305	3.777	1.204	1.403	1.271	1.453	FAM91 carboxy-terminus protein	GI-H
MELO3C003574.2	0.778	0.840	4.124	NA	0.607	1.517	0.607	Phosphatase 2C family protein	GI-H
MELO3C003593.2	0.273	0.534	3.616	0.201	0.414	0.370	0.309	Bromo-adjacent-like (BAH) domain protein	GI-H
MELO3C003595.2	1.383	2.387	7.530	3.016	2.357	4.903	0.999	Myosin heavy chain-related	GI-H
MELO3C003597.2	1.293	1.887	4.134	2.489	0.662	11.300	1.070	ABC1 family protein	GI-H
MELO3C003598.2	0.451	9.032	1.345	16.468	0.573	28.352	0.339	Plant/F12B17-70 protein	GI-H
MELO3C003603.2	0.974	1.293	23.802	0.867	2.229	0.428	3.016	Gamma-glutamylcyclotransferase	GI-H
MELO3C003609.2	0.980	1.006	3.906	0.538	1.132	0.240	1.093	DNA topoisomerase 1 isoform X2	GI-H

Gene ID	FPKM							Gene Description	Specific in episode
	FS	GI-M	GM-M	AN-M	GI-H	GM-H	AN-H		
MELO3C003621.2	2.733	3.040	6.623	3.548	2.286	4.764	2.215	Keratin, type I cytoskeletal 27	GI-H
MELO3C003626.2	1.282	1.464	7.216	0.824	1.676	0.585	1.508	E3 ubiquitin-protein ligase arkadia	GI-H
MELO3C003636.2	1.540	2.656	7.582	2.775	3.652	1.926	2.961	cytoplasmic 60S subunit biogenesis factor REI1 homolog 1	GI-H
MELO3C003637.2	2.412	2.681	7.002	1.565	2.988	3.033	2.321	Elongation factor 1-alpha, putative	GI-H
MELO3C003638.2	1.052	0.715	8.913	0.668	1.806	NA	1.367	replication protein A 32 kDa subunit A-like	GI-H
MELO3C003652.2	0.174	0.157	1.738	NA	0.283	0.105	0.312	DDT domain-containing protein DDR4	GI-H
MELO3C003661.2	0.363	0.805	1.515	0.556	0.634	0.652	0.575	SWIM zinc finger family protein	GI-H
MELO3C003669.2	3.127	5.146	14.863	5.080	4.890	7.050	4.147	Polyadenylate-binding protein-interacting protein 5	GI-H
MELO3C003671.2	3.236	2.464	8.226	2.475	3.371	3.941	3.409	SPOC domain/transcription elongation factor S-II, putative	GI-H
MELO3C003672.2	0.812	0.734	4.642	0.260	0.541	NA	0.870	cyclin-A2-2 isoform X1	GI-H
MELO3C003686.2	1.100	3.734	3.582	1.628	0.373	6.913	0.374	transcription factor HY5	GI-H
MELO3C003697.2	1.393	1.427	21.507	1.725	3.201	0.724	2.471	Protein WVD2-like 5	GI-H
MELO3C003706.2	0.391	0.569	6.637	0.807	0.298	1.448	0.304	Myosin heavy chain-related protein	GI-H
MELO3C003707.2	3.490	4.423	15.235	1.996	4.663	0.907	5.008	DNA-directed RNA polymerases II, IV and V subunit 6A-like	GI-H
MELO3C003730.2	0.677	1.401	1.885	2.456	0.744	3.161	0.794	calcium-dependent protein kinase 29	GI-H
MELO3C003731.2	0.530	1.227	1.443	0.892	0.317	2.693	0.502	Protein disulfide isomerase (PDI)-like protein	GI-H
MELO3C003733.2	3.781	2.783	10.107	3.166	2.154	1.787	3.401	ribonuclease P protein subunit p25-like protein isoform X1	GI-H
MELO3C003748.2	0.830	0.597	7.115	0.678	1.429	0.336	1.454	Translation initiation factor IF-2	GI-H
MELO3C003766.2	0.650	0.969	6.287	0.657	1.232	0.277	1.005	DNA-directed RNA polymerase I subunit rpa43	GI-H
MELO3C003773.2	5.709	5.899	18.569	3.326	9.108	1.774	6.995	Protein IQ-DOMAIN 32	GI-H
MELO3C003778.2	1.860	2.139	7.213	4.918	1.174	3.240	0.837	MADS-box transcription factor	GI-H
MELO3C003813.2	1.896	2.824	6.409	2.567	2.512	2.224	2.610	Mannan endo-1,4-beta-mannosidase-like protein	GI-H
MELO3C030545.2	0.763	0.616	3.779	0.763	0.545	0.282	0.328	Ty1-copia retrotransposon protein	GI-H
MELO3C003825.2	0.243	0.471	2.183	NA	0.726	0.554	0.737	Cysteine/Histidine-rich C1 domain family protein, putative	GI-H
MELO3C003831.2	0.750	0.928	4.576	0.806	2.033	0.407	1.314	Maf-like protein	GI-H
MELO3C003834.2	3.659	2.048	18.288	3.116	4.675	1.090	2.920	60S ribosomal protein L24	GI-H
MELO3C003837.2	1.146	1.606	3.120	1.063	1.102	2.116	1.335	DUF248-1	GI-H
MELO3C003838.2	0.369	0.585	2.543	0.457	1.127	0.355	0.575	Erythronate-4-phosphate dehydrogenase family protein	GI-H
MELO3C003839.2	0.405	0.829	5.180	1.290	0.198	0.302	0.521	Random slug protein 5	GI-H
MELO3C003888.2	0.874	0.875	3.196	0.391	1.072	0.215	0.853	Pentatricopeptide repeat-containing family protein	GI-H
MELO3C026489.2	2.391	5.540	6.236	3.855	2.803	3.844	2.719	cytochrome P450 CYP82D47-like	GI-H

Gene ID	FPKM							Gene Description	Specific in episode
	FS	GI-M	GM-M	AN-M	GI-H	GM-H	AN-H		
MELO3C018242.2	11.824	28.303	29.663	11.261	5.894	64.605	7.599	NAC domain-containing protein 72-like	GI-H
MELO3C018220.2	0.767	0.417	3.229	0.646	1.343	0.191	0.773	small nuclear ribonucleoprotein Sm D1-like	GI-H
MELO3C030675.2	0.795	0.407	2.404	0.692	0.713	NA	0.415	Unknown protein	GI-H
MELO3C030440.2	0.461	1.084	6.013	2.643	1.671	1.472	1.340	transmembrane protein 184 homolog DDB_G0279555-like	GI-H
MELO3C018189.2	2.114	3.229	14.215	1.426	3.034	2.723	2.832	myosin-2	GI-H
MELO3C030695.2	1.378	1.863	8.672	3.902	3.242	0.438	2.882	Plasma membrane ATPase	GI-H
MELO3C018168.2	1.152	2.246	4.815	1.596	2.251	0.919	1.686	Serine/threonine-protein kinase	GI-H
MELO3C018160.2	0.244	0.292	2.661	0.290	0.444	0.024	0.353	factor of DNA methylation 1-like	GI-H
MELO3C018138.2	0.350	0.353	3.549	NA	0.690	NA	0.515	C2H2-like zinc finger protein	GI-H
MELO3C018132.2	0.728	0.932	11.617	0.665	1.304	0.188	0.697	Kinesin-like protein	GI-H
MELO3C018128.2	0.305	0.331	2.664	0.239	0.458	0.088	0.275	Pentatricopeptide repeat-containing protein	GI-H
MELO3C030723.2	0.765	0.694	2.241	0.607	0.892	0.389	0.475	peroxiredoxin Q, chloroplastic-like	GI-H
MELO3C030736.2	3.531	2.642	14.032	2.297	3.532	0.862	3.688	FHA domain-containing protein DDL	GI-H
MELO3C030496.2	0.475	0.605	2.615	0.379	0.454	0.415	0.435	ATPase 10, plasma membrane-type-like isoform X1	GI-H
MELO3C030762.2	0.211	0.327	2.949	0.273	0.386	NA	0.257	Protein DETOXIFICATION	GI-H
MELO3C012813.2	1.699	1.785	7.593	1.024	2.253	0.336	2.005	Phosphatidylinositol N-acetylglucosaminlytransferase subunit P-related	GI-H
MELO3C012831.2	0.144	0.250	1.193	0.176	0.145	0.259	0.208	WD repeat-containing protein 26	GI-H
MELO3C012848.2	1.732	2.165	12.143	1.320	3.163	2.065	2.287	Myb-like transcription factor family protein	GI-H
MELO3C012881.2	0.917	1.533	8.903	1.314	1.146	1.243	0.895	rho-N domain-containing protein 1, chloroplastic isoform X3	GI-H
MELO3C012894.2	2.214	4.715	11.026	6.856	2.950	11.683	4.011	actin-depolymerizing factor	GI-H
MELO3C012899.2	0.133	0.510	2.950	0.204	0.649	0.191	0.345	transcription termination factor MTEF18, mitochondrial-like	GI-H
MELO3C012901.2	0.491	0.538	2.143	0.576	0.525	0.645	1.032	cell division control protein 48 homolog C-like	GI-H
MELO3C012906.2	0.313	0.213	4.947	NA	0.658	NA	1.084	CDT1-like protein a, chloroplastic	GI-H
MELO3C012921.2	0.581	1.694	4.782	1.172	0.526	0.346	0.454	GDSL esterase/lipase	GI-H
MELO3C012922.2	0.154	0.244	2.395	NA	0.666	NA	0.302	Pentatricopeptide repeat-containing protein, putative	GI-H
MELO3C012928.2	5.189	11.545	33.347	7.949	12.143	1.466	11.171	long chain acyl-CoA synthetase 1	GI-H
MELO3C012930.2	2.225	2.506	6.723	0.797	1.461	1.631	1.932	Zinc knuckle family protein	GI-H
MELO3C012945.2	1.002	4.384	3.246	22.965	0.857	41.073	1.587	Pleiotropic drug resistance ABC transporter	GI-H
MELO3C012957.2	0.259	0.275	4.568	0.495	0.493	0.867	0.367	Pentatricopeptide repeat-containing protein	GI-H
MELO3C012958.2	0.538	0.347	1.091	0.255	0.321	0.370	0.281	methyltransferase-like protein 22 isoform X1	GI-H

Gene ID	FPKM							Gene Description	Specific in episode
	FS	GI-M	GM-M	AN-M	GI-H	GM-H	AN-H		
MELO3C012960.2	1.941	1.122	4.090	1.030	1.307	5.359	1.138	BZIP protein, putative	GI-H
MELO3C012969.2	2.145	2.301	7.696	3.743	2.892	0.880	2.614	Protein disulfide isomerase, putative	GI-H
MELO3C012975.2	0.076	0.625	3.069	0.506	0.413	0.758	0.464	transcription factor bHLH68-like isoform X1	GI-H
MELO3C030795.2	0.136	0.131	1.441	NA	0.085	0.157	0.126	Serine/threonine-protein kinase EDR1	GI-H
MELO3C012982.2	0.221	0.421	3.098	0.427	0.343	0.437	0.607	Bet1-like SNARE 1-1	GI-H
MELO3C012998.2	2.922	8.548	6.454	2.551	2.021	6.225	2.901	ABC transporter G family member 29-like	GI-H
MELO3C013005.2	0.440	0.565	2.009	NA	0.782	0.533	0.230	Syntaxin-124 protein	GI-H
MELO3C013022.2	0.666	1.408	14.508	1.059	0.946	1.026	0.648	Serine/threonine-protein kinase ATM	GI-H
MELO3C013029.2	5.825	5.265	103.415	7.668	10.668	1.366	7.349	60S ribosomal protein L28, putative	GI-H
MELO3C013030.2	0.871	0.829	2.431	1.012	1.093	0.233	1.156	Tudor/PWWP/MBT superfamily protein	GI-H
MELO3C013034.2	4.432	6.352	17.989	3.834	5.524	6.477	6.122	Charged multivesicular body 1	GI-H
MELO3C013044.2	1.822	1.953	6.249	2.096	3.080	1.725	2.870	Serine/threonine-protein phosphatase 2A 55 kDa regulatory subunit B	GI-H
MELO3C030574.2	0.244	0.169	2.416	NA	0.195	0.307	0.380	mediator of RNA polymerase II transcription subunit 9	GI-H
MELO3C013078.2	0.284	0.424	3.706	0.459	0.509	0.491	0.151	telomere repeat-binding factor 4-like	GI-H
MELO3C013081.2	2.241	5.080	15.762	7.193	4.690	7.570	3.167	Acetyltransferase component of pyruvate dehydrogenase complex	GI-H
MELO3C013082.2	7.626	15.432	17.254	6.404	6.395	30.032	5.992	Protein phosphatase 2c	GI-H
MELO3C013084.2	0.478	1.756	3.475	0.595	0.897	1.540	0.588	Aminopeptidase	GI-H
MELO3C013095.2	0.459	0.440	1.044	0.289	0.499	0.556	0.329	General transcription factor 3C polypeptide 3	GI-H
MELO3C025799.2	0.806	1.253	3.125	1.256	1.122	1.974	0.927	Alpha-ketoglutarate-dependent dioxygenase AlkB	GI-H
MELO3C022674.2	0.812	0.724	2.214	0.882	0.916	0.657	1.089	protein SDA1 homolog	GI-H
MELO3C022698.2	1.502	1.852	7.606	0.213	3.053	0.054	2.236	Remorin family protein	GI-H
MELO3C022702.2	1.111	1.489	3.543	0.904	1.430	0.225	1.109	Mitotic spindle checkpoint protein bubr1	GI-H
MELO3C022704.2	1.791	0.930	3.598	0.869	1.459	0.352	1.314	pectinesterase-like	GI-H
MELO3C022712.2	0.546	0.581	2.061	0.384	0.969	0.282	0.700	B-cell receptor-associated protein 31-like	GI-H
MELO3C030883.2	1.163	1.069	4.234	0.810	0.688	0.722	1.108	RPM1-interacting protein 4	GI-H
MELO3C022715.2	0.214	0.328	2.341	0.400	0.402	0.595	0.365	E3 ubiquitin-protein ligase RNF4-like isoform X1	GI-H
MELO3C022719.2	0.231	0.207	2.036	NA	0.088	NA	0.163	Genomic DNA, chromosome 3, P1 clone: MJL12	GI-H
MELO3C022720.2	0.314	0.239	1.724	0.477	0.360	0.384	0.188	Gamma-tubulin complex component	GI-H
MELO3C022721.2	9.586	19.976	24.064	6.281	11.737	10.993	7.730	Ninja-family protein AFP3	GI-H
MELO3C022725.2	0.435	0.620	2.070	0.244	0.547	0.810	0.458	RING-type E3 ubiquitin transferase	GI-H

Gene ID	FPKM							Gene Description	Specific in episode
	FS	GI-M	GM-M	AN-M	GI-H	GM-H	AN-H		
MELO3C022763.2	0.385	0.958	2.286	1.481	0.911	1.310	0.419	Isoflavone reductase like	GI-H
MELO3C022766.2	0.214	0.164	1.324	0.378	0.434	0.226	0.238	50S ribosomal protein L35	GI-H
MELO3C026722.2	4.209	3.839	12.312	3.384	3.924	2.108	3.411	Phosphoribulokinase	GI-H
MELO3C026715.2	0.203	0.404	1.165	0.245	0.265	0.098	0.278	Tetratricopeptide repeat (TPR)-like superfamily protein	GI-H
MELO3C026711.2	0.265	0.413	2.401	0.213	0.299	0.409	0.192	Protein FAM32A-like	GI-H
MELO3C026701.2	0.713	0.659	3.309	1.013	0.991	0.144	0.892	DNA/RNA-binding protein Alba-like protein	GI-H
MELO3C026657.2	0.248	0.180	1.940	NA	0.415	0.076	0.235	Glycine-rich protein	GI-H
MELO3C026986.2	0.794	0.598	2.014	0.960	0.878	0.466	0.944	NHL domain-containing protein	GI-H
MELO3C026997.2	0.177	0.614	1.435	0.318	0.465	0.532	0.475	BSD domain-containing protein	GI-H
MELO3C030913.2	0.624	1.028	3.637	1.537	1.136	0.380	1.534	60S ribosomal protein L7	GI-H
MELO3C024039.2	4.630	4.072	11.367	2.368	5.340	1.494	5.357	Poly [ADP-ribose] polymerase	GI-H
MELO3C024023.2	0.978	1.886	2.930	1.020	0.755	3.644	1.067	F-box protein At1g55000	GI-H
MELO3C024020.2	4.342	6.015	49.751	8.388	11.421	3.649	8.063	60S ribosomal protein L44	GI-H
MELO3C030661.2	1.991	1.614	9.836	4.033	2.728	1.955	1.758	Protein TIC 214	GI-H
MELO3C023996.2	1.743	2.201	4.554	1.789	2.135	2.029	2.127	RNA polymerase sigma factor sigD, chloroplastic	GI-H
MELO3C023986.2	1.608	1.059	23.687	0.435	3.586	0.079	2.655	DNA topoisomerase 2	GI-H
MELO3C023984.2	1.953	2.708	7.150	2.041	2.104	0.781	2.330	Peptidylprolyl isomerase	GI-H
MELO3C023983.2	2.501	2.274	11.225	2.521	3.857	1.213	3.380	DNA-directed RNA polymerases II, IV and V subunit 11	GI-H
MELO3C023972.2	0.367	0.193	1.240	0.049	0.231	0.066	0.298	telomerase reverse transcriptase	GI-H
MELO3C023947.2	3.307	4.841	9.822	15.917	4.680	58.708	4.027	O-fucosyltransferase family protein	GI-H
MELO3C026732.2	24.169	31.305	49.294	6.923	9.648	36.789	8.270	Chaperone protein dnaj 8, chloroplastic	GI-H
MELO3C026749.2	6.185	3.816	32.437	2.747	11.141	1.302	11.150	DNA helicase	GI-H
MELO3C026886.2	0.869	1.391	3.491	1.151	1.617	1.270	1.238	Chaperone DnaJ	GI-H
MELO3C026617.2	0.480	0.366	1.436	0.399	0.489	0.300	0.337	Pentatricopeptide repeat-containing family protein	GI-H
MELO3C026612.2	2.171	2.050	5.203	1.281	1.584	2.126	1.890	Eukaryotic translation initiation factor 4E	GI-H
MELO3C026609.2	0.268	0.754	2.491	0.477	0.607	0.140	0.311	beta-glucosidase BoGH3B-like	GI-H
MELO3C026605.2	0.648	0.454	1.912	0.214	0.398	0.155	0.491	Double-stranded RNA-binding protein	GI-H
MELO3C026593.2	2.323	1.663	7.432	1.884	2.019	0.753	1.752	Surfeit locus protein 2 (SURF2)	GI-H
MELO3C026589.2	0.794	0.978	6.147	1.632	0.673	3.009	0.717	SGF29 tudor-like domain-containing protein	GI-H
MELO3C009974.2	0.099	0.784	1.292	NA	0.405	0.242	0.358	Transmembrane protein, putative	GI-H
MELO3C009946.2	1.937	1.566	4.737	1.636	1.505	1.861	1.875	methyl-CpG-binding domain-containing protein 9	GI-H

Gene ID	FPKM							Gene Description	Specific in episode
	FS	GI-M	GM-M	AN-M	GI-H	GM-H	AN-H		
MELO3C009943.2	0.323	0.335	1.521	0.863	0.751	0.127	0.272	Transmembrane protein	GI-H
MELO3C009933.2	0.145	0.543	3.589	NA	0.623	0.056	0.442	alpha-dioxygenase 1	GI-H
MELO3C009925.2	1.514	2.122	4.034	2.263	1.826	1.239	1.691	senescence-associated carboxylesterase 101	GI-H
MELO3C009879.2	2.099	2.685	9.177	2.145	3.336	2.767	2.245	Cullin family protein	GI-H
MELO3C009874.2	1.057	2.417	6.128	2.931	2.629	3.303	2.761	Myb family transcription factor family protein	GI-H
MELO3C030701.2	0.938	1.265	5.972	1.261	1.899	0.763	2.771	cytochrome P450 CYP72A219-like	GI-H
MELO3C009855.2	3.346	5.417	24.041	2.266	2.282	3.773	2.893	NAC domain-containing protein 100-like	GI-H
MELO3C030973.2	0.275	0.362	2.002	NA	0.151	0.194	0.453	TMV resistance protein N	GI-H
MELO3C030975.2	0.598	0.767	3.478	0.873	0.721	0.943	0.745	Branched-chain-amino-acid aminotransferase	GI-H
MELO3C009834.2	0.630	0.734	4.222	0.422	0.872	NA	0.766	Chorismate mutase	GI-H
MELO3C009826.2	0.741	4.517	3.313	0.701	0.696	0.986	0.355	Carboxypeptidase	GI-H
MELO3C009824.2	0.790	0.866	4.405	NA	0.491	0.626	0.724	CRM-domain containing factor CFM3, chloroplastic/mitochondrial	GI-H
MELO3C009803.2	0.718	0.484	2.742	0.418	0.816	NA	0.579	Pentatricopeptide repeat-containing family protein	GI-H
MELO3C030716.2	0.569	0.639	2.543	0.491	0.875	0.132	0.493	Pentatricopeptide repeat-containing family protein	GI-H
MELO3C009771.2	0.765	1.047	3.090	0.660	1.037	1.345	0.791	Ectonucleotide pyrophosphatase/phosphodiesterase family member 3	GI-H
MELO3C009764.2	0.995	1.501	15.203	0.978	1.208	NA	1.608	ribosomal RNA small subunit methyltransferase NEP1	GI-H
MELO3C009763.2	0.293	0.326	3.780	NA	0.954	0.066	0.701	centromere-associated protein E	GI-H
MELO3C009754.2	0.949	1.258	4.315	0.664	0.782	0.585	0.711	ARM repeat superfamily protein	GI-H
MELO3C030717.2	5.056	5.013	15.950	2.654	6.598	0.971	6.368	Pentatricopeptide repeat-containing protein At1g80270, mitochondrial	GI-H
MELO3C009734.2	0.238	0.515	1.188	0.537	0.289	0.753	0.363	Exocyst complex component	GI-H
MELO3C009725.2	0.463	0.566	2.229	NA	0.597	NA	0.735	Protein ROOT PRIMORDIUM DEFECTIVE 1	GI-H
MELO3C009713.2	2.340	3.074	5.771	1.736	2.515	1.562	2.444	translation factor GUF1 homolog, chloroplastic	GI-H
MELO3C009708.2	0.334	0.895	6.001	0.964	1.051	0.547	1.676	MYB transcription factor	GI-H
MELO3C009691.2	3.469	8.533	13.964	5.259	5.311	4.587	4.933	14 kDa zinc-binding protein	GI-H
MELO3C009685.2	2.209	3.291	6.060	3.133	2.848	6.934	2.850	insulin-degrading enzyme-like 1, peroxisomal	GI-H
MELO3C009683.2	0.046	NA	2.739	NA	0.304	NA	0.033	Serine hydrolase FSH	GI-H
MELO3C009674.2	0.585	0.826	1.835	0.292	0.352	0.283	0.216	Beta-glucosidase, putative	GI-H
MELO3C009665.2	0.794	0.704	1.986	1.119	0.991	0.701	0.590	LOW QUALITY PROTEIN: LRR receptor-like serine/threonine-protein kinase FLS2	GI-H
MELO3C009655.2	0.277	0.315	6.991	NA	0.353	0.187	1.196	Chaperone protein dnaJ 6	GI-H

Gene ID	FPKM							Gene Description	Specific in episode
	FS	GI-M	GM-M	AN-M	GI-H	GM-H	AN-H		
MELO3C009644.2	1.364	3.382	5.566	3.616	2.186	4.063	2.325	Outer envelope pore 16-3, chloroplastic/mitochondrial-like protein	GI-H
MELO3C009633.2	2.662	2.751	7.128	2.113	2.455	1.378	2.448	PHOTOPERIOD-INDEPENDENT EARLY FLOWERING 1 family protein	GI-H
MELO3C009608.2	0.589	0.413	2.084	0.884	0.517	0.102	0.369	DNA-directed RNA polymerase subunit beta	GI-H
MELO3C009592.2	1.101	1.482	4.909	1.698	1.656	0.771	1.245	mediator of RNA polymerase II transcription subunit 7a-like	GI-H
MELO3C009586.2	1.997	2.259	14.709	2.197	3.340	2.618	2.390	Ubiquitin carboxyl-terminal hydrolase, putative	GI-H
MELO3C009583.2	1.653	2.001	3.919	2.888	1.334	4.337	1.524	E3 ubiquitin-protein ligase BAH1-like protein, putative	GI-H
MELO3C009580.2	0.768	0.927	5.962	1.205	0.604	0.898	1.320	suppressor of mec-8 and unc-52 protein homolog 2	GI-H
MELO3C009566.2	2.085	2.792	14.473	2.686	3.142	1.878	2.611	Protein WVD2-like 4	GI-H
MELO3C009560.2	1.542	2.330	4.293	2.146	1.869	3.698	1.530	Polyketide cyclase/dehydrase/lipid transport superfamily protein	GI-H
MELO3C009559.2	1.924	2.446	3.965	1.353	1.899	2.596	1.931	ubiquitin carboxyl-terminal hydrolase 8-like	GI-H
MELO3C009547.2	1.056	1.156	4.125	1.394	1.304	1.003	1.266	Trafficking protein particle complex subunit 8	GI-H
MELO3C009546.2	1.923	1.648	5.053	1.336	2.085	1.766	2.230	ATP-dependent RNA helicase, putative	GI-H
MELO3C009510.2	0.467	0.615	2.349	1.317	0.432	1.733	0.783	TLD domain-containing protein 1	GI-H
MELO3C009493.2	2.281	3.035	11.064	2.166	3.516	1.093	3.104	Peptidyl-prolyl cis-trans isomerase	GI-H
MELO3C009486.2	2.745	2.244	18.633	1.722	4.395	3.291	3.888	Kinesin-related protein	GI-H
MELO3C009473.2	2.212	1.425	6.876	1.988	1.162	0.966	1.413	glucosamine 6-phosphate N-acetyltransferase	GI-H
MELO3C009472.2	1.390	3.840	7.343	3.907	2.969	5.464	3.576	cytochrome b-c1 complex subunit 9	GI-H
MELO3C009443.2	1.332	1.661	9.939	0.697	2.157	0.628	1.161	Ankyrin repeat 30A-like protein (DUF662)	GI-H
MELO3C009432.2	3.886	3.993	10.556	3.065	2.675	2.990	3.141	protein ROS1	GI-H
MELO3C009430.2	2.828	3.520	25.674	4.048	3.492	1.449	3.028	histone deacetylase HDT1	GI-H
MELO3C009417.2	1.343	1.655	4.117	0.952	1.801	0.798	0.961	E3 ubiquitin-protein ligase UPL7	GI-H
MELO3C009414.2	0.413	0.532	1.327	0.212	0.289	0.530	0.234	wall-associated receptor kinase-like 20	GI-H
MELO3C009413.2	1.791	1.418	6.260	1.370	2.551	1.136	1.561	Cactin	GI-H
MELO3C009401.2	0.145	0.864	2.619	0.853	0.743	0.344	0.295	Myosin heavy chain-like protein, putative	GI-H
MELO3C009392.2	1.197	0.922	5.448	1.118	1.378	0.269	1.115	F-box protein CPR30	GI-H
MELO3C009382.2	3.431	4.592	14.569	3.129	6.739	3.276	7.183	Phosphatidylinositol-4-phosphate 5-kinase, putative	GI-H
MELO3C009362.2	0.558	0.942	1.657	2.297	0.525	8.968	0.556	4-coumarate--CoA ligase-like 7	GI-H
MELO3C009355.2	0.619	0.899	5.210	2.100	0.357	5.564	0.733	heavy metal-associated isoprenylated plant protein 3-like	GI-H
MELO3C009354.2	2.409	2.433	30.594	2.560	2.753	2.745	2.014	Glutathione gamma-glutamylcysteinyltransferase 1	GI-H
MELO3C009351.2	0.821	0.887	3.147	NA	0.700	0.328	0.583	Phosphoglycerate kinase	GI-H

Gene ID	FPKM							Gene Description	Specific in episode
	FS	GI-M	GM-M	AN-M	GI-H	GM-H	AN-H		
MELO3C009336.2	2.835	5.985	17.733	10.969	3.727	7.826	4.366	V-type proton ATPase subunit G	GI-H
MELO3C009332.2	0.409	0.565	1.755	NA	0.403	0.898	0.657	protein indeterminate-domain 9	GI-H
MELO3C009327.2	4.157	8.900	9.452	8.634	3.667	9.735	4.668	DNA-binding protein S1FA-like	GI-H
MELO3C009318.2	0.158	0.207	2.354	0.136	0.143	0.136	0.184	Ribonuclease P protein subunit p29	GI-H
MELO3C009316.2	2.938	1.165	16.988	0.713	3.553	0.148	3.277	Interactor of constitutive active ROPs-like protein	GI-H
MELO3C009305.2	2.300	1.667	6.483	1.601	2.700	8.026	2.546	protein REVEILLE 8-like isoform X1	GI-H
MELO3C009301.2	0.316	0.431	1.524	0.502	0.338	1.187	0.257	Protein kinase-like protein	GI-H
MELO3C009290.2	1.747	2.737	8.332	3.573	2.011	5.683	2.031	Phosphatidate phosphatase PAH1	GI-H
MELO3C009286.2	0.344	0.729	4.047	1.032	0.670	0.853	0.787	heavy metal-associated isoprenylated plant protein 3-like	GI-H
MELO3C009285.2	1.721	1.845	3.726	0.752	1.481	2.216	1.406	Phosphatidylinositol N-acetylglucosaminlytransferase subunit P-like protein	GI-H
MELO3C009284.2	3.286	2.763	9.435	1.238	4.026	1.082	3.867	Protein SLOW GREEN 1, chloroplastic	GI-H
MELO3C009281.2	2.103	1.814	4.837	2.503	2.241	3.361	1.852	Ras-related protein-like protein	GI-H
MELO3C009280.2	0.978	1.964	6.389	1.944	1.813	0.488	3.135	AT3g53630/F4P12_330	GI-H
MELO3C009279.2	9.874	5.697	52.024	11.338	13.397	1.388	14.459	Histone H2B	GI-H
MELO3C009271.2	0.645	0.924	6.963	0.373	1.048	18.136	0.267	Short-chain dehydrogenase TIC 32, chloroplastic	GI-H
MELO3C030739.2	8.122	3.528	17.121	2.771	8.014	1.402	5.897	Myb family transcription factor	GI-H
MELO3C009253.2	2.247	2.912	4.951	2.104	1.795	0.829	1.780	SCARECROW-like protein	GI-H
MELO3C009252.2	6.809	11.145	35.212	20.524	10.651	22.925	10.245	Haloacid dehalogenase-like hydrolase	GI-H
MELO3C009241.2	0.462	1.710	7.864	1.771	1.139	0.816	1.198	High-light-induced protein chloroplastic	GI-H
MELO3C009231.2	0.508	0.682	9.300	0.592	1.003	0.101	0.686	transcription factor bHLH155-like	GI-H
MELO3C009183.2	7.401	7.914	30.742	12.270	13.693	10.147	11.195	26S proteasome non-ATPase regulatory subunit 7 homolog A	GI-H
MELO3C009172.2	2.809	6.474	8.989	1.889	3.113	2.413	2.268	RING finger protein	GI-H
MELO3C009149.2	0.970	1.344	10.013	1.829	1.236	0.628	0.976	Copper chaperone SCO1/SenC	GI-H
MELO3C009135.2	0.897	0.635	2.305	0.532	0.748	0.117	0.962	protein BREAST CANCER SUSCEPTIBILITY 2 homolog B-like	GI-H
MELO3C009115.2	3.189	3.118	13.102	1.380	5.133	0.667	6.181	WEB family protein At2g38370	GI-H
MELO3C009096.2	0.855	1.144	6.460	1.021	1.271	1.348	0.735	Mitochondrial ATP synthase D chain-related protein	GI-H
MELO3C014731.2	2.388	2.657	4.880	2.869	2.238	3.696	1.811	Glucosidase 2 subunit beta	GI-H
MELO3C014726.2	0.468	0.489	1.409	0.338	0.470	1.180	0.494	Unknown protein	GI-H
MELO3C014719.2	0.405	0.680	1.875	1.931	0.900	5.941	0.660	Aldehyde oxidase, putative	GI-H
MELO3C014717.2	0.197	0.323	1.606	0.175	0.262	NA	0.189	Unknown protein	GI-H

Gene ID	FPKM							Gene Description	Specific in episode
	FS	GI-M	GM-M	AN-M	GI-H	GM-H	AN-H		
MELO3C014716.2	0.921	1.485	4.103	0.851	1.329	0.752	1.013	Aldehyde oxidase, putative	GI-H
MELO3C014704.2	3.211	4.840	8.141	5.348	3.297	8.275	2.898	BZIP transcription factor, putative (DUF630 and DUF632)	GI-H
MELO3C014700.2	3.221	4.942	19.295	6.009	5.270	15.514	3.980	Non-specific serine/threonine protein kinase	GI-H
MELO3C014699.2	1.968	2.401	12.226	1.936	2.594	2.155	1.867	COP9 signalosome complex subunit 2	GI-H
MELO3C014695.2	0.401	0.662	3.651	0.252	0.494	0.300	0.633	oligoribonuclease	GI-H
MELO3C014691.2	1.436	2.358	13.668	2.422	2.708	3.130	2.158	outer envelope protein 61	GI-H
MELO3C014689.2	1.489	1.788	10.551	1.622	1.725	2.005	1.393	Ubiquitin carboxyl-terminal hydrolase-like protein	GI-H
MELO3C014687.2	1.907	1.867	6.179	1.143	2.039	1.344	1.496	Structural maintenance of chromosomes protein	GI-H
MELO3C014686.2	0.189	0.175	1.306	NA	0.179	NA	0.082	IQ domain-containing protein IQM2	GI-H
MELO3C014671.2	0.634	0.946	1.977	0.711	0.440	0.789	0.835	branchpoint-bridging protein isoform X2	GI-H
MELO3C014670.2	0.824	0.776	5.734	1.267	1.039	0.938	1.269	E3 ubiquitin-protein ligase BRE1-like 2	GI-H
MELO3C014661.2	0.400	1.257	2.582	1.574	0.711	1.091	0.545	protein EMBRYO SAC DEVELOPMENT ARREST 3, chloroplastic isoform X1	GI-H
MELO3C014656.2	2.158	16.640	35.913	3.790	3.797	6.062	6.120	Peroxidase	GI-H
MELO3C014649.2	0.510	0.735	2.425	0.828	0.553	0.539	0.830	Metal tolerance protein C2	GI-H
MELO3C014647.2	0.743	0.722	2.859	0.847	0.390	1.781	0.408	Early endosome antigen 1	GI-H
MELO3C014639.2	0.353	0.817	6.440	0.320	0.779	NA	0.746	DNA topoisomerase 6 subunit B	GI-H
MELO3C014638.2	26.763	15.931	116.916	5.196	53.606	0.498	35.966	Lipoxygenase	GI-H
MELO3C014619.2	1.314	1.738	4.857	1.753	1.321	0.530	1.875	IRK-interacting protein-like	GI-H
MELO3C014616.2	0.875	0.524	3.723	NA	1.818	NA	1.627	At5g12930	GI-H
MELO3C014611.2	1.897	1.549	5.953	1.300	2.044	1.130	2.432	Pre-mRNA-splicing factor CWC22	GI-H
MELO3C014604.2	0.333	0.276	2.760	0.329	0.531	NA	0.304	F16F4.11 protein	GI-H
MELO3C014601.2	2.097	3.145	9.429	3.741	3.033	0.520	2.764	Aldehyde dehydrogenase	GI-H
MELO3C014572.2	0.206	0.259	3.879	NA	0.530	NA	0.254	Mg-protoporphyrin IX chelatase	GI-H
MELO3C014569.2	0.782	1.079	3.000	0.700	1.050	1.443	0.832	protein CHROMATIN REMODELING 5	GI-H
MELO3C014567.2	2.313	1.734	9.285	NA	1.290	6.201	2.033	Reactive oxygen species modulator 1	GI-H
MELO3C014565.2	1.200	1.321	7.256	0.861	1.600	0.796	1.461	Homeobox domain-containing protein	GI-H
MELO3C014555.2	3.946	4.751	8.946	3.662	4.029	3.944	3.763	splicing factor 3B subunit 6-like protein	GI-H
MELO3C031085.2	0.377	1.025	2.675	1.144	0.357	1.265	0.734	dihydropyrimidinase	GI-H
MELO3C014548.2	0.549	1.058	2.605	0.827	0.684	0.980	0.979	Methyltransferase family protein, putative	GI-H
MELO3C014546.2	1.088	1.257	8.191	2.257	1.074	0.412	1.070	Octanoyltransferase	GI-H

Gene ID	FPKM							Gene Description	Specific in episode
	FS	GI-M	GM-M	AN-M	GI-H	GM-H	AN-H		
MELO3C014534.2	1.772	1.147	21.106	0.339	4.387	NA	2.656	G2/mitotic-specific cyclin S13-7	GI-H
MELO3C014528.2	0.736	1.343	6.286	0.627	1.265	0.198	1.144	replication factor C subunit 1	GI-H
MELO3C014527.2	0.791	1.341	4.247	1.270	1.245	1.120	1.345	YABBY protein	GI-H
MELO3C014510.2	0.403	0.575	2.280	0.608	0.445	0.365	0.631	NAC domain-containing protein 40	GI-H
MELO3C014502.2	1.511	1.724	9.378	1.403	3.128	1.836	2.545	RNA-binding family protein isoform 1	GI-H
MELO3C014493.2	1.238	1.194	3.210	0.842	1.537	2.054	1.392	Chromatin remodeling factor, putative	GI-H
MELO3C014475.2	1.964	0.920	6.638	0.596	2.074	0.975	1.082	glycerol-3-phosphate acyltransferase 1	GI-H
MELO3C031104.2	1.367	1.668	6.509	1.129	1.966	1.812	2.525	zinc finger BED domain-containing protein RICESLEEPER 1-like isoform X1	GI-H
MELO3C014460.2	0.359	0.299	4.204	0.339	0.285	0.063	0.379	Unknown protein	GI-H
MELO3C014447.2	0.317	0.568	3.037	NA	0.890	NA	0.536	Nuclear ribonuclease Z	GI-H
MELO3C014437.2	5.735	15.325	28.407	3.272	6.601	2.911	4.312	1-aminocyclopropane-1-carboxylate oxidase 1	GI-H
MELO3C031114.2	0.793	0.596	2.889	0.532	1.062	0.411	0.898	lysine--tRNA ligase-like	GI-H
MELO3C014408.2	0.708	0.781	3.797	0.464	1.087	0.165	0.689	transcription factor bHLH69-like isoform X1	GI-H
MELO3C014401.2	0.788	1.524	5.109	0.796	1.352	0.453	1.359	Thiamine phosphate synthase	GI-H
MELO3C014394.2	6.007	7.548	15.587	5.455	6.464	3.841	5.378	Ribosomal N-lysine methyltransferase 5	GI-H
MELO3C014391.2	0.394	0.436	3.896	0.192	0.546	0.087	0.552	Ribosome recycling factor	GI-H
MELO3C014387.2	0.948	0.995	3.922	1.283	1.411	0.729	1.765	CDK5RAP1-like protein	GI-H
MELO3C014386.2	0.969	1.563	3.310	1.061	1.348	0.783	1.076	COP9 signalosome complex subunit 8-like	GI-H
MELO3C014351.2	4.360	3.385	11.228	2.155	3.417	2.290	2.715	Transcription factor GTE4	GI-H
MELO3C014348.2	1.110	1.363	12.728	1.200	0.652	0.913	0.692	Glutathione peroxidase	GI-H
MELO3C014346.2	0.902	0.943	2.939	1.009	1.016	0.598	0.885	protein PALE CRESS, chloroplastic	GI-H
MELO3C014340.2	0.794	0.597	2.225	0.430	0.738	0.640	0.672	Glutamine-rich protein	GI-H
MELO3C014317.2	9.740	10.024	40.129	7.514	18.846	2.008	18.182	Histone H2A	GI-H
MELO3C014309.2	0.757	1.495	3.615	1.017	1.631	0.127	1.029	Magnesium transporter MRS2-like protein	GI-H
MELO3C014308.2	0.240	0.310	1.976	NA	0.333	0.707	0.543	E3 ubiquitin protein ligase DRIP2-like	GI-H
MELO3C014299.2	0.834	1.010	12.727	0.328	1.631	0.224	1.324	protein NETWORKED 1D-like	GI-H
MELO3C014293.2	2.026	2.829	8.926	1.580	3.136	0.652	1.960	type I inositol polyphosphate 5-phosphatase 4	GI-H
MELO3C014292.2	49.157	58.078	158.463	57.797	70.017	16.872	51.794	40S ribosomal protein S3a-like	GI-H
MELO3C014291.2	1.656	1.730	8.243	0.947	2.535	0.612	1.752	Heat shock protein STI	GI-H
MELO3C014290.2	2.415	2.058	6.035	1.708	1.652	0.401	1.843	Protein IQ-DOMAIN 31	GI-H

Gene ID	FPKM							Gene Description	Specific in episode
	FS	GI-M	GM-M	AN-M	GI-H	GM-H	AN-H		
MELO3C014282.2	2.642	7.470	21.355	10.782	3.976	13.620	5.574	1,2-dihydroxy-3-keto-5-methylthiopentene dioxygenase	GI-H
MELO3C014260.2	0.569	1.226	2.736	1.153	0.484	0.248	0.524	Interactor of constitutive active ROPs-like protein	GI-H
MELO3C014252.2	1.373	1.414	4.416	1.053	1.115	NA	1.026	Peroxidase	GI-H
MELO3C026765.2	3.090	4.391	14.232	23.960	5.520	9.861	6.047	Protein SIEVE ELEMENT OCCLUSION B	GI-H
MELO3C026766.2	0.887	1.709	6.052	3.541	0.649	0.291	0.815	Protein SIEVE ELEMENT OCCLUSION B	GI-H
MELO3C026769.2	0.186	NA	1.180	0.146	0.094	NA	0.126	Protein SIEVE ELEMENT OCCLUSION B	GI-H
MELO3C026775.2	0.291	0.334	4.173	1.173	0.429	0.223	0.343	Protein SIEVE ELEMENT OCCLUSION B	GI-H
MELO3C026782.2	0.675	1.022	4.500	0.986	0.784	0.444	0.879	Protein WVD2-like 3	GI-H
MELO3C022991.2	28.824	34.910	346.757	3.129	4.051	NA	11.578	MADS-box protein AGL42-like isoform X1	GI-H
MELO3C023003.2	0.780	1.024	9.406	0.728	0.840	0.781	1.065	Kinase family protein	GI-H
MELO3C023018.2	2.854	3.532	6.194	0.889	2.378	2.008	2.306	splicing factor U2af large subunit B isoform X1	GI-H
MELO3C023027.2	0.928	3.709	7.511	2.787	1.102	1.421	0.989	17 kDa phloem lectin	GI-H
MELO3C023032.2	0.344	0.520	4.254	1.072	0.497	2.323	0.415	Cyclopropane-fatty-acyl-phospholipid synthase family protein	GI-H
MELO3C023050.2	0.678	1.258	8.698	0.653	0.695	0.987	0.634	Nucleolar matrix family protein	GI-H
MELO3C023064.2	1.258	1.161	4.772	1.272	1.472	1.874	0.992	BLISTER	GI-H
MELO3C023070.2	7.016	9.115	16.274	6.021	7.525	8.440	6.883	Small acidic protein 1	GI-H
MELO3C020443.2	1.136	2.168	4.478	1.238	1.807	0.716	0.940	ER lumen protein-retaining receptor-like	GI-H
MELO3C020427.2	1.006	1.268	2.059	0.818	0.922	2.348	0.931	DEAD-box ATP-dependent RNA helicase 42-like	GI-H
MELO3C020422.2	1.951	2.471	5.972	1.758	2.900	1.048	2.606	ARM repeat superfamily protein	GI-H
MELO3C020421.2	0.211	0.207	2.169	NA	0.265	0.078	0.304	Unknown protein	GI-H
MELO3C020417.2	0.365	0.229	2.522	0.511	0.657	0.792	0.525	transcription factor bHLH30-like	GI-H
MELO3C020413.2	2.238	2.512	5.398	2.041	2.440	2.992	2.554	SNARE-interacting protein KEULE isoform X1	GI-H
MELO3C031134.2	0.524	0.565	2.069	0.339	0.566	0.279	0.529	histone-lysine N-methyltransferase ASHH3	GI-H
MELO3C020397.2	0.028	0.324	1.218	0.427	0.032	0.166	0.066	Isoprenylcysteine alpha-carbonyl methylesterase ICME-like protein	GI-H
MELO3C020369.2	7.655	6.046	49.643	12.767	14.420	3.372	11.635	60S ribosomal protein L37a	GI-H
MELO3C020359.2	0.128	0.398	1.170	0.305	0.250	0.323	0.149	actin-related protein 2/3 complex subunit 4-like	GI-H
MELO3C020355.2	0.455	0.783	3.183	NA	0.890	NA	0.678	Pentatricopeptide repeat-containing family protein	GI-H
MELO3C008507.2	1.454	2.502	4.672	1.234	1.458	1.902	2.237	Protein disulfide-isomerase A5	GI-H
MELO3C008513.2	1.720	2.247	7.426	1.358	1.607	0.603	1.611	FAST kinase domain-containing 3	GI-H
MELO3C031364.2	0.747	1.588	10.688	0.973	1.395	0.976	1.070	glutamate--cysteine ligase, chloroplastic	GI-H

Gene ID	FPKM							Gene Description	Specific in episode
	FS	GI-M	GM-M	AN-M	GI-H	GM-H	AN-H		
MELO3C008519.2	1.484	0.893	3.040	0.999	0.579	1.123	0.905	Vacuolar protein sorting-associated protein 9A	GI-H
MELO3C031365.2	0.229	0.321	2.479	0.265	0.233	NA	0.232	Afadin/alpha-actinin-binding protein	GI-H
MELO3C008527.2	5.307	4.488	14.842	6.163	5.072	1.955	5.134	Cysteine and histidine-rich domain-containing protein RAR1	GI-H
MELO3C031382.2	1.601	0.907	7.129	1.048	1.785	0.836	0.901	myb family transcription factor APL	GI-H
MELO3C008569.2	19.174	18.638	68.419	36.108	22.498	5.512	22.854	60S ribosomal protein L38	GI-H
MELO3C008572.2	0.304	0.348	4.726	0.277	0.341	0.082	0.325	Disease resistance protein	GI-H
MELO3C008574.2	0.716	1.474	1.786	1.944	0.516	3.197	0.331	Beta-glucosidase	GI-H
MELO3C008582.2	0.276	15.156	2.090	21.772	0.688	3.428	0.796	sugar transport protein 10-like	GI-H
MELO3C008614.2	1.060	0.859	2.129	0.619	0.925	1.433	0.897	Hexosyltransferase	GI-H
MELO3C008634.2	0.611	0.706	2.628	0.313	0.567	0.690	0.659	INO80 complex subunit C	GI-H
MELO3C008673.2	0.296	0.632	1.710	NA	0.535	0.132	0.444	UDP-N-acetylmuramoyl-L-alanyl-D-glutamate--2, 6-diaminopimelate ligase	GI-H
MELO3C008695.2	0.490	0.269	2.440	NA	0.416	NA	0.408	Ankyrin repeat and protein kinase domain-containing protein 1	GI-H
MELO3C008699.2	0.861	0.781	3.632	0.386	1.374	0.265	1.717	Alpha-(1,6)-fucosyltransferase	GI-H
MELO3C008708.2	0.304	0.315	1.490	0.232	0.306	0.376	0.214	protein prenyltransferase alpha subunit repeat-containing protein 1	GI-H
MELO3C008710.2	0.416	0.380	1.228	0.305	0.336	0.423	0.358	RNA-binding protein 42-like	GI-H
MELO3C008714.2	2.768	3.438	6.106	3.665	2.917	11.257	2.848	Transcription factor jumonji family protein	GI-H
MELO3C008741.2	0.306	1.145	1.789	0.601	0.497	5.924	0.423	ABC transporter B family protein	GI-H
MELO3C008750.2	0.737	0.987	2.264	1.112	0.618	1.859	0.940	Monothiol glutaredoxin	GI-H
MELO3C031452.2	1.444	2.133	3.031	2.313	0.901	6.558	0.949	Amino acid permease	GI-H
MELO3C008753.2	0.315	0.445	1.165	0.453	0.472	0.579	0.551	Catalytic/ hydrolase	GI-H
MELO3C008757.2	0.361	0.344	3.469	0.263	0.382	0.571	0.339	U3 small nucleolar RNA-associated protein 11	GI-H
MELO3C008759.2	1.643	1.936	4.709	2.000	2.195	3.052	1.961	Monocopper oxidase-like protein SKU5	GI-H
MELO3C003910.2	0.304	0.292	5.337	0.901	0.486	0.108	0.873	Prefoldin subunit 1	GI-H
MELO3C003924.2	0.559	0.473	1.145	0.361	0.312	0.439	0.425	Nuclear speckle splicing regulatory-like protein (DUF2040)	GI-H
MELO3C003928.2	0.437	0.864	3.092	0.573	0.798	0.496	0.709	Pentatricopeptide repeat-containing family protein	GI-H
MELO3C003935.2	0.798	0.789	2.120	0.527	0.945	0.232	0.962	DExH-box ATP-dependent RNA helicase DExH10	GI-H
MELO3C003958.2	0.576	0.205	3.853	NA	0.503	NA	0.861	LOW QUALITY PROTEIN: protein SMAX1-LIKE 4-like	GI-H
MELO3C003975.2	0.440	0.781	3.061	0.184	0.520	0.572	0.327	protein CHLOROPLAST IMPORT APPARATUS 2 isoform X2	GI-H
MELO3C003981.2	0.282	0.338	1.849	0.291	0.124	0.658	0.172	Pentatricopeptide repeat-containing family protein	GI-H

Gene ID	FPKM							Gene Description	Specific in episode
	FS	GI-M	GM-M	AN-M	GI-H	GM-H	AN-H		
MELO3C003984.2	0.438	0.572	3.199	0.516	0.469	0.289	0.646	Core-2/I-branching beta-16-N-acetylglucosaminyltransferase family protein	GI-H
MELO3C003986.2	3.529	3.485	34.448	5.313	3.573	3.799	3.304	30S ribosomal protein S12, chloroplastic	GI-H
MELO3C003990.2	1.251	1.619	15.220	1.036	0.827	1.624	0.771	BURP domain protein RD22	GI-H
MELO3C031488.2	0.936	0.777	2.689	1.137	0.748	0.939	0.902	Heat shock 70 kDa protein 16	GI-H
MELO3C004002.2	1.195	1.662	2.961	0.663	1.129	1.105	1.203	nifU-like protein 3, chloroplastic	GI-H
MELO3C004005.2	4.806	5.231	25.444	3.928	5.710	1.315	6.714	Lysine--tRNA ligase	GI-H
MELO3C004009.2	1.221	0.956	2.867	0.918	0.888	0.478	1.248	Pentatricopeptide repeat-containing family protein	GI-H
MELO3C004022.2	0.841	0.974	8.230	0.823	1.553	0.128	1.583	Afadin/alpha-actinin-binding protein	GI-H
MELO3C004028.2	0.570	0.875	2.277	0.723	1.088	1.921	0.518	Syntaxin/T-SNARE family protein	GI-H
MELO3C004030.2	0.800	0.442	2.174	0.283	0.928	0.059	0.933	[ribulose-bisphosphate carboxylase]-lysine N-methyltransferase, putative	GI-H
MELO3C004054.2	2.236	3.635	18.727	5.901	1.842	3.242	1.282	(R)-mandelonitrile lyase 1-like	GI-H
MELO3C004059.2	0.284	0.412	3.099	NA	0.767	NA	0.275	(R)-mandelonitrile lyase 1-like	GI-H
MELO3C004081.2	2.465	2.924	6.108	2.610	2.549	3.984	2.677	Kinase family protein	GI-H
MELO3C004107.2	1.185	1.912	4.179	1.552	1.595	0.923	1.494	HD domain-containing protein 2	GI-H
MELO3C004108.2	0.500	1.106	7.236	1.010	1.005	1.279	1.064	Tetratricopeptide repeat-containing protein	GI-H
MELO3C004109.2	2.344	3.241	5.707	2.134	1.238	3.030	1.875	transmembrane protein 208 homolog	GI-H
MELO3C031521.2	0.454	0.585	1.483	0.275	0.445	0.642	0.347	Autophagy-related protein 18b	GI-H
MELO3C004116.2	2.086	2.264	4.489	2.066	1.110	4.499	1.313	Autophagy-related protein 11	GI-H
MELO3C004132.2	1.826	2.549	6.287	2.213	2.656	4.614	1.954	TOM1-like protein 2	GI-H
MELO3C004140.2	0.946	1.495	2.220	1.532	1.050	1.557	1.042	transmembrane protein 184A-like	GI-H
MELO3C004159.2	0.445	0.358	2.362	0.312	0.430	0.168	0.379	Glucose-6-phosphate 1-dehydrogenase	GI-H
MELO3C004172.2	2.203	2.203	5.835	1.315	1.879	1.175	2.773	Metallopeptidase M24 family protein	GI-H
MELO3C004181.2	2.572	3.725	14.459	3.154	2.691	0.112	1.196	homeobox protein knotted-1-like 1 isoform X1	GI-H
MELO3C031531.2	2.337	2.312	5.757	2.202	2.089	2.840	2.689	zinc finger BED domain-containing protein DAYSLEEPER isoform X2	GI-H
MELO3C004191.2	0.518	0.791	7.165	0.572	0.647	0.517	0.681	Antigenic heat-stable protein	GI-H
MELO3C004192.2	1.089	0.766	2.632	0.775	1.044	0.871	1.216	RNA pseudouridine synthase 1	GI-H
MELO3C004195.2	1.718	2.392	7.636	NA	3.105	2.764	3.736	universal stress protein A-like protein	GI-H
MELO3C004198.2	1.495	2.032	11.160	2.809	3.020	3.954	2.256	zinc finger matrin-type protein 2	GI-H
MELO3C004201.2	0.393	0.303	1.516	0.240	0.739	0.157	0.363	ATP-dependent DNA helicase Q-like 1	GI-H

Gene ID	FPKM							Gene Description	Specific in episode
	FS	GI-M	GM-M	AN-M	GI-H	GM-H	AN-H		
MELO3C004226.2	0.469	0.457	1.820	0.707	0.480	0.780	0.626	ATP-dependent DNA helicase Q-like SIM	GI-H
MELO3C004234.2	0.128	0.157	1.332	0.085	0.205	0.054	0.108	protein ALWAYS EARLY 2-like	GI-H
MELO3C004237.2	0.747	0.852	1.820	0.669	0.894	0.614	0.797	DNA polymerase	GI-H
MELO3C004244.2	5.352	13.775	57.191	9.379	8.452	0.716	5.007	Lipoxygenase	GI-H
MELO3C031318.2	3.863	22.937	47.525	10.077	4.308	0.937	3.531	Lipoxygenase	GI-H
MELO3C004247.2	0.175	1.023	1.884	1.054	0.279	0.915	0.209	Lipoxygenase	GI-H
MELO3C004249.2	0.422	4.148	4.379	1.187	0.762	0.151	0.564	Lipoxygenase	GI-H
MELO3C004259.2	0.119	0.423	2.037	0.354	0.311	NA	0.310	TMV resistance protein N-like isoform X1	GI-H
MELO3C004267.2	1.154	0.670	7.798	0.205	1.680	0.265	2.378	Kinesin-like protein	GI-H
MELO3C004268.2	0.157	0.224	4.440	NA	1.039	NA	0.760	Microtubule-binding protein TANGLED	GI-H
MELO3C031544.2	0.673	1.522	4.311	1.100	1.894	NA	1.055	Pentatricopeptide repeat-containing protein	GI-H
MELO3C004287.2	1.250	0.455	17.793	NA	0.955	NA	0.851	transcription factor bHLH94-like	GI-H
MELO3C004317.2	0.564	0.150	6.217	0.183	0.115	NA	0.220	Vat protein	GI-H
MELO3C004318.2	0.370	0.312	7.769	NA	0.872	NA	0.314	Vat protein	GI-H
MELO3C004329.2	1.405	2.296	4.290	1.082	1.277	0.403	1.309	Terpene cyclase/mutase family member	GI-H
MELO3C004333.2	2.169	2.824	10.885	1.727	2.802	2.884	2.174	DCD (Development and Cell Death) domain protein	GI-H
MELO3C004337.2	0.218	0.443	2.702	0.368	0.232	0.378	0.382	vesicle-associated protein 2-2 isoform X1	GI-H
MELO3C004345.2	0.828	1.002	3.675	0.692	1.573	0.743	1.115	Translation initiation factor eIF-2B subunit epsilon	GI-H
MELO3C004354.2	3.053	4.187	7.404	1.621	2.769	1.690	3.105	Cc-nbs-lrr resistance protein	GI-H
MELO3C004360.2	34.081	41.581	163.427	38.330	27.048	11.584	23.686	40S ribosomal protein S24	GI-H
MELO3C004361.2	0.792	1.357	6.968	1.174	1.435	0.415	0.735	WNK kinase	GI-H
MELO3C004366.2	1.689	3.834	7.404	3.088	3.574	3.519	2.614	Prenylyltransferase superfamily protein	GI-H
MELO3C004372.2	0.793	2.054	3.781	2.397	1.417	7.151	1.502	Peptide-N(4)-(N-acetyl-beta-glucosaminyl)asparagine amidase	GI-H
MELO3C004381.2	0.325	4.347	4.145	9.918	0.836	12.070	1.154	Auxin-responsive protein	GI-H
MELO3C004396.2	0.454	0.238	2.425	NA	0.214	0.460	0.127	Protein WVD2-like 1	GI-H
MELO3C004426.2	2.906	2.062	6.360	3.415	2.287	1.575	2.373	Reticulon-like protein	GI-H
MELO3C004433.2	0.497	0.352	4.435	0.203	0.346	0.429	0.226	calcium uptake protein 1, mitochondrial-like isoform X1	GI-H
MELO3C004450.2	0.588	1.073	2.212	0.749	0.601	1.334	0.887	protein ENHANCED DISEASE RESISTANCE 2	GI-H
MELO3C004457.2	1.495	1.379	6.632	1.206	2.366	0.340	1.853	Histone acetyltransferase	GI-H
MELO3C004466.2	0.605	0.558	3.257	0.452	0.363	0.828	0.409	Alpha-galactosidase	GI-H
MELO3C004481.2	1.177	1.491	3.173	1.845	1.181	1.299	1.217	triphosphate tunel metalloenzyme 3-like	GI-H

Gene ID	FPKM							Gene Description	Specific in episode
	FS	GI-M	GM-M	AN-M	GI-H	GM-H	AN-H		
MELO3C004486.2	1.164	1.310	5.828	1.622	1.957	2.832	2.602	Temperature-induced lipocalin	GI-H
MELO3C004489.2	0.682	0.629	3.328	0.300	0.951	0.306	0.730	two-component response regulator ARR12-like	GI-H
MELO3C004492.2	0.632	0.659	5.361	0.405	0.809	0.342	0.471	methyl-CpG-binding domain-containing protein 11-like	GI-H
MELO3C004504.2	7.332	8.001	15.986	3.774	3.294	19.081	3.002	SPX domain-containing protein 1	GI-H
MELO3C004505.2	0.898	0.362	8.932	1.001	0.954	0.312	0.904	Ribosomal protein L31	GI-H
MELO3C004506.2	2.329	3.473	10.557	3.603	4.283	0.580	4.357	Chaperone DnaJ	GI-H
MELO3C004507.2	1.747	2.099	5.020	2.679	1.719	0.343	1.923	DUF3119 family protein	GI-H
MELO3C004519.2	1.751	2.443	10.083	6.277	2.438	1.653	2.681	NADH-cytochrome b5 reductase-like protein	GI-H
MELO3C004522.2	0.334	NA	3.164	NA	1.436	2.875	1.313	Early nodulin 93 protein	GI-H
MELO3C004531.2	3.141	4.635	15.821	2.932	5.290	4.400	3.506	26S protease regulatory subunit, putative	GI-H
MELO3C004534.2	0.706	2.037	3.271	1.194	0.863	3.672	1.204	Glycine-rich RNA-binding protein, putative	GI-H
MELO3C004562.2	0.155	NA	3.672	NA	0.203	NA	0.133	E3 ubiquitin-protein ligase TRIM9	GI-H
MELO3C004575.2	1.212	2.122	5.555	1.827	2.536	3.398	1.604	ninja-family protein mc410	GI-H
MELO3C004581.2	3.167	3.317	7.673	2.826	2.707	5.354	2.545	Protein phosphatase 1 regulatory subunit pprA	GI-H
MELO3C004583.2	1.246	1.732	4.016	2.117	1.255	2.421	1.202	golgin candidate 1 isoform X1	GI-H
MELO3C004594.2	1.208	0.480	9.275	NA	1.628	0.305	1.668	mitogen-activated protein kinase kinase 6	GI-H
MELO3C004611.2	0.838	1.515	5.676	0.884	1.326	3.669	1.547	vacuolar protein sorting-associated protein 32 homolog 2	GI-H
MELO3C004613.2	18.457	26.770	72.516	19.912	19.939	7.188	19.302	60S ribosomal protein L31	GI-H
MELO3C004623.2	0.737	0.620	2.838	0.387	0.650	0.763	0.855	Dystrophin-1	GI-H
MELO3C004629.2	10.436	9.939	57.656	12.241	12.881	11.833	10.023	nucleosome assembly protein 13-like	GI-H
MELO3C004635.2	0.501	1.036	6.058	NA	2.207	0.115	1.484	Protein kinase	GI-H
MELO3C004652.2	0.897	1.654	6.502	1.199	1.430	1.318	1.167	O-fucosyltransferase family protein	GI-H
MELO3C004659.2	0.495	0.409	2.489	0.270	0.518	0.616	0.661	ATP-dependent DNA helicase Q-like 3 isoform X1	GI-H
MELO3C004660.2	1.016	1.234	3.103	0.968	0.879	1.250	0.869	Trichome birefringence-like 16	GI-H
MELO3C031585.2	0.482	0.554	1.989	0.447	0.829	0.496	0.447	Unknown protein	GI-H
MELO3C005943.2	0.482	0.461	1.997	0.452	0.320	0.478	0.419	GRAM domain family protein, putative isoform 1	GI-H
MELO3C005945.2	0.347	0.330	1.528	0.268	0.753	0.101	0.410	Dihydroorotate dehydrogenase (DUF3598)	GI-H
MELO3C005971.2	1.253	1.570	6.572	0.737	1.411	1.288	1.102	E3 ubiquitin-protein ligase BRE1-like 1	GI-H
MELO3C005976.2	1.926	0.744	5.258	0.570	1.185	0.279	1.210	Protein AATF	GI-H
MELO3C005981.2	1.204	1.181	2.785	1.206	0.976	1.402	0.746	Elongation factor 4	GI-H
MELO3C005984.2	2.028	2.423	8.893	0.620	3.141	0.369	2.788	Pentatricopeptide repeat-containing family protein	GI-H

Gene ID	FPKM							Gene Description	Specific in episode
	FS	GI-M	GM-M	AN-M	GI-H	GM-H	AN-H		
MELO3C005985.2	2.240	2.136	9.375	2.092	2.117	2.880	1.727	heterogeneous nuclear ribonucleoprotein R isoform X2	GI-H
MELO3C005992.2	1.707	3.082	12.814	0.424	0.900	5.202	1.343	homeobox-leucine zipper protein ATHB-7	GI-H
MELO3C006009.2	0.675	0.708	2.508	0.330	0.750	0.294	0.737	pre-rRNA-processing protein esf1	GI-H
MELO3C006019.2	0.407	1.227	2.650	1.143	0.498	4.260	0.265	lysine-specific demethylase JMJ25	GI-H
MELO3C006021.2	0.130	0.239	5.027	NA	0.275	NA	0.230	Embryo defective 1703	GI-H
MELO3C006028.2	0.953	2.237	5.788	0.681	2.579	0.057	2.794	cyclic nucleotide-gated ion channel 1	GI-H
MELO3C006031.2	1.380	1.875	5.194	1.206	1.818	2.663	1.670	cyclic nucleotide-gated ion channel 1-like	GI-H
MELO3C006050.2	0.488	0.767	2.912	0.375	0.525	0.765	0.392	Phosphatidylinositol-4-phosphate 5-kinase family protein	GI-H
MELO3C006052.2	1.183	0.990	4.387	0.943	1.553	0.626	1.494	Copper ion-binding protein	GI-H
MELO3C006065.2	0.742	0.940	3.763	0.770	1.855	0.759	1.402	Pentatricopeptide repeat-containing protein	GI-H
MELO3C006074.2	0.439	0.747	3.023	0.749	0.928	1.885	0.497	U11/U12 small nuclear ribonucleoprotein 59 kDa protein isoform X1	GI-H
MELO3C006086.2	1.142	3.292	4.560	9.374	1.927	26.873	1.565	protein REVEILLE 6 isoform X2	GI-H
MELO3C006087.2	6.956	8.028	17.222	5.480	8.495	9.050	7.638	Chorismate synthase	GI-H
MELO3C006096.2	0.714	0.608	2.610	0.620	0.619	0.760	0.584	DNA replication complex GINS protein SLD5	GI-H
MELO3C031604.2	0.404	0.650	3.440	0.496	1.642	0.470	0.962	Receptor-like kinase	GI-H
MELO3C006100.2	1.280	2.870	6.851	1.534	2.111	1.678	2.391	Ubiquitin-conjugating enzyme, E2	GI-H
MELO3C006101.2	6.315	7.349	16.501	5.466	6.374	6.367	7.028	ATP-dependent helicase BRM	GI-H
MELO3C006115.2	0.477	0.889	2.362	0.325	1.052	0.764	0.589	Unknown protein	GI-H
MELO3C006116.2	0.277	0.363	5.250	NA	0.177	0.147	0.755	Peptidyl-prolyl cis-trans isomerase	GI-H
MELO3C006119.2	1.250	2.631	9.306	1.301	1.754	3.352	2.115	Remorin	GI-H
MELO3C006121.2	6.701	7.135	27.746	6.078	8.345	5.239	8.001	Dead box ATP-dependent RNA helicase	GI-H
MELO3C006123.2	3.034	5.379	14.430	3.332	3.357	8.308	3.588	Acetyl-coenzyme A carboxylase carboxyl transferase subunit alpha	GI-H
MELO3C006130.2	1.087	1.422	4.448	2.289	2.074	1.625	1.325	Bifunctional protein FolD	GI-H
MELO3C006133.2	1.626	1.629	6.018	1.462	1.697	1.727	1.827	digalactosyldiacylglycerol synthase 1, chloroplastic-like	GI-H
MELO3C006154.2	0.435	0.914	4.402	0.236	0.713	0.410	0.634	Cyclin-D-binding Myb-like transcription factor 1	GI-H
MELO3C006168.2	0.894	0.943	4.720	1.619	2.069	4.839	1.292	Cytochrome P450	GI-H
MELO3C006173.2	1.089	0.930	7.589	0.418	2.070	NA	2.317	Serine/threonine-protein kinase, putative	GI-H
MELO3C006175.2	0.962	1.029	3.241	0.811	1.138	1.125	0.996	ankyrin repeat and zinc finger domain-containing protein 1	GI-H
MELO3C006177.2	0.421	0.312	2.193	0.297	0.453	0.144	0.431	SMAD/FHA domain-containing protein, putative isoform 1	GI-H
MELO3C006188.2	1.466	1.273	5.081	0.844	2.171	0.436	1.515	ATP-dependent DNA helicase 2 subunit KU80	GI-H

Gene ID	FPKM							Gene Description	Specific in episode
	FS	GI-M	GM-M	AN-M	GI-H	GM-H	AN-H		
MELO3C006194.2	0.642	1.210	2.880	0.466	1.261	NA	0.680	Pentatricopeptide repeat-containing family protein	GI-H
MELO3C006197.2	1.251	1.645	2.968	1.248	1.149	1.260	1.181	Polyphosphatidylinositol phosphatase	GI-H
MELO3C006205.2	2.912	2.961	12.355	2.725	3.296	1.892	1.800	Myosin-4 protein (DUF641)	GI-H
MELO3C006209.2	1.106	0.406	4.763	NA	1.034	0.467	1.323	chromo domain protein LHP1-like	GI-H
MELO3C006216.2	0.814	0.926	3.147	0.339	1.244	0.364	0.858	B3 domain-containing protein	GI-H
MELO3C006219.2	0.499	0.695	3.419	0.735	1.054	0.162	0.717	Pentatricopeptide repeat-containing protein	GI-H
MELO3C006238.2	0.662	0.940	6.271	1.719	0.732	1.458	0.554	Pentatricopeptide repeat-containing protein At1g18900	GI-H
MELO3C006247.2	0.727	2.235	1.537	1.370	0.377	0.401	0.448	ABC transporter family protein	GI-H
MELO3C006251.2	2.523	2.441	9.182	2.038	3.286	1.619	4.368	Zinc finger family protein	GI-H
MELO3C006259.2	1.107	1.083	5.936	0.815	1.786	0.591	0.788	Nucleolar GTP-binding protein 1	GI-H
MELO3C006271.2	1.932	1.897	5.035	3.351	1.778	1.861	1.957	At2g44670/F16B22.16	GI-H
MELO3C006273.2	1.208	1.839	3.495	0.724	1.117	3.361	1.324	Receptor-like kinase plant	GI-H
MELO3C006276.2	1.982	2.453	6.562	2.995	2.496	1.045	2.394	At5g17610	GI-H
MELO3C006277.2	0.886	1.845	4.415	1.498	1.278	4.112	0.857	Gb|AAF01580.1	GI-H
MELO3C006288.2	0.540	0.599	2.175	0.608	0.375	1.863	0.226	Heat Stress Transcription Factor family protein	GI-H
MELO3C006292.2	0.648	0.691	4.389	0.482	1.252	0.205	0.947	DNA repair protein RAD50	GI-H
MELO3C006294.2	1.179	1.613	3.121	1.250	0.873	0.760	1.193	Dna repair helicase xpb1	GI-H
MELO3C006299.2	1.772	2.303	7.271	1.162	3.273	0.727	2.143	RNA-directed DNA polymerase (Reverse transcriptase)-related family protein	GI-H
MELO3C006302.2	0.905	1.477	3.521	0.793	1.258	0.534	1.755	RPM1-interacting protein 4	GI-H
MELO3C006310.2	0.381	0.885	3.453	0.658	0.566	0.761	0.553	Ubiquitinyl hydrolase 1	GI-H
MELO3C006312.2	0.106	0.216	6.719	NA	0.473	0.307	0.165	Calcium-dependent protein kinase	GI-H
MELO3C006327.2	0.339	0.305	2.549	0.205	0.534	0.086	0.772	chromosome transmission fidelity protein 18 homolog	GI-H
MELO3C006332.2	1.648	1.357	3.750	1.177	1.693	3.353	1.539	E3 ubiquitin-protein ligase SDIR1	GI-H
MELO3C006341.2	0.536	0.312	2.841	0.268	0.755	0.128	1.048	Holliday junction resolvase	GI-H
MELO3C006348.2	0.395	0.354	5.331	NA	0.507	0.979	0.723	PHD finger protein ING	GI-H
MELO3C006360.2	0.159	0.268	1.150	0.249	0.372	0.104	0.170	Myosin	GI-H
MELO3C006372.2	0.961	3.930	4.943	7.410	1.597	11.698	1.761	Tetratricopeptide repeat (TPR)-like superfamily protein	GI-H
MELO3C006382.2	2.285	3.715	7.431	2.261	3.263	7.121	2.662	DNA ligase B	GI-H
MELO3C006386.2	0.241	0.246	2.546	0.400	0.250	NA	0.366	Arp2/3 complex 34 kDa subunit	GI-H
MELO3C006416.2	1.575	1.302	10.986	1.515	2.766	0.697	1.559	Splicing factor 3B, subunit 5	GI-H

Gene ID	FPKM							Gene Description	Specific in episode
	FS	GI-M	GM-M	AN-M	GI-H	GM-H	AN-H		
MELO3C006421.2	19.922	34.085	135.721	27.257	39.768	7.744	34.681	60S ribosomal protein L7, putative	GI-H
MELO3C006422.2	0.666	0.544	7.998	0.444	1.210	0.218	0.689	SRP40, carboxy-terminal domain protein	GI-H
MELO3C006423.2	0.971	1.345	4.293	1.538	1.246	1.143	1.396	Vesicle transport USE1	GI-H
MELO3C006424.2	0.722	0.961	2.474	0.564	1.176	0.980	0.459	Pre-mRNA-splicing factor SLU7	GI-H
MELO3C006459.2	0.703	0.743	2.078	0.172	0.724	0.331	0.875	FHA domain containing protein, expressed	GI-H
MELO3C006469.2	1.507	1.704	5.184	1.007	1.365	2.110	1.093	Zinc knuckle (CCHC-type) family protein, putative	GI-H
MELO3C006472.2	0.284	0.648	2.252	0.514	0.453	0.327	0.569	Chloroplastic group IIA intron splicing facilitator CRS1, chloroplastic	GI-H
MELO3C006473.2	2.752	3.987	6.369	1.932	2.227	1.622	1.301	DNA-directed RNA polymerase II subunit	GI-H
MELO3C006480.2	0.956	0.387	2.503	1.734	0.256	0.752	0.293	Ion channel pollux-like protein	GI-H
MELO3C006492.2	0.116	NA	1.521	NA	0.129	NA	0.263	At4g14650	GI-H
MELO3C006498.2	3.212	6.483	13.168	6.956	5.115	19.617	4.443	transmembrane emp24 domain-containing protein p24beta3	GI-H
MELO3C006507.2	24.703	32.855	97.914	28.712	45.798	7.961	34.480	40S ribosomal protein S3a	GI-H
MELO3C006509.2	4.492	4.144	31.164	4.521	8.524	17.029	8.374	protein NETWORKED 1A	GI-H
MELO3C006513.2	0.368	0.704	5.308	1.190	0.985	NA	1.073	protein RDM1	GI-H
MELO3C006528.2	0.657	0.484	2.445	0.351	1.004	0.447	0.708	At1g05410/T25N20_5	GI-H
MELO3C006538.2	4.916	2.723	52.190	2.563	4.698	0.207	4.104	Spermidine synthase	GI-H
MELO3C006554.2	0.761	1.333	2.950	1.372	0.736	3.676	0.865	Unknown protein	GI-H
MELO3C006573.2	1.748	1.653	3.600	1.402	1.275	2.910	1.701	BTB-POZ and MATH domain 2	GI-H
MELO3C031711.2	1.245	2.738	5.930	2.169	1.915	2.179	2.398	protein FAR1-RELATED SEQUENCE 7-like	GI-H
MELO3C006582.2	1.931	1.732	4.873	1.590	2.272	1.163	1.697	SAC3 family protein B	GI-H
MELO3C006601.2	7.922	8.413	17.919	5.230	8.038	7.504	7.959	chromatin structure-remodeling complex protein SYD isoform X1	GI-H
MELO3C006603.2	0.652	1.058	7.595	1.442	1.402	0.389	1.510	vesicle-associated protein 2-2-like isoform X1	GI-H
MELO3C006614.2	2.382	2.677	11.619	2.375	3.326	1.166	2.705	Adenylyl cyclase	GI-H
MELO3C006642.2	2.897	2.447	6.910	1.355	3.401	2.176	3.144	protein ALWAYS EARLY 3 isoform X2	GI-H
MELO3C006659.2	1.866	1.335	7.089	3.039	2.176	NA	2.308	Uveal autoantigen with coiled-coil domains and ankyrin repeats isoform 4	GI-H
MELO3C006689.2	0.650	0.615	3.457	1.099	0.948	0.935	1.199	Chaperone protein dnaJ 49	GI-H
MELO3C006702.2	0.077	0.271	1.875	0.338	0.254	0.498	0.138	cAMP-regulated phosphoprotein-like protein	GI-H
MELO3C006705.2	1.920	2.254	6.975	1.653	2.476	3.230	2.054	Binding protein	GI-H
MELO3C006736.2	0.159	0.202	1.143	NA	0.433	NA	0.241	Beta-1,3-galactosyltransferase-like protein	GI-H

Gene ID	FPKM							Gene Description	Specific in episode
	FS	GI-M	GM-M	AN-M	GI-H	GM-H	AN-H		
MELO3C006742.2	2.146	2.817	8.730	2.698	1.795	2.800	2.271	Complex 1 LYR protein	GI-H
MELO3C006747.2	0.667	0.816	1.945	0.905	0.771	0.840	0.875	signal recognition particle 19 kDa protein	GI-H
MELO3C006750.2	1.626	3.074	7.474	1.322	2.678	2.471	2.027	DNA binding protein	GI-H
MELO3C006758.2	1.735	3.255	8.970	1.694	1.865	4.490	2.352	Non-specific serine/threonine protein kinase	GI-H
MELO3C006765.2	0.386	0.666	3.128	NA	0.835	0.094	0.680	DEAD-box ATP-dependent RNA helicase 50	GI-H
MELO3C006781.2	4.802	4.531	18.920	3.319	5.755	1.899	5.688	titin homolog isoform X2	GI-H
MELO3C006788.2	1.067	1.073	2.428	0.709	0.879	0.609	0.911	DNA topoisomerase	GI-H
MELO3C006789.2	1.380	1.622	4.332	1.989	1.338	9.653	1.349	transcription factor bHLH62-like	GI-H
MELO3C006790.2	2.636	1.722	8.218	0.588	3.036	0.495	3.302	kinesin-like protein KIN12B	GI-H
MELO3C006793.2	0.592	0.909	1.951	0.524	0.584	1.281	0.538	electron transfer flavoprotein-ubiquinone oxidoreductase, mitochondrial	GI-H
MELO3C006797.2	1.000	0.932	2.604	0.402	0.835	0.488	0.836	B-block binding subunit of TFIIIC	GI-H
MELO3C006807.2	0.867	0.551	4.558	0.314	0.910	0.142	0.952	Structural maintenance of chromosomes protein	GI-H
MELO3C006817.2	0.668	0.847	2.347	NA	0.994	0.815	0.748	pectinesterase-like	GI-H
MELO3C006822.2	0.536	0.518	3.837	0.323	0.517	0.145	0.506	UMP-CMP kinase	GI-H
MELO3C006829.2	30.415	35.962	87.356	37.326	40.199	14.324	39.168	40S ribosomal protein S20, putative	GI-H
MELO3C006858.2	0.792	1.082	5.045	1.348	1.679	0.369	0.803	At5g51840	GI-H
MELO3C006861.2	1.289	1.401	6.268	0.641	1.178	0.807	0.571	2-oxoglutarate (2OG) and Fe(II)-dependent oxygenase superfamily protein	GI-H
MELO3C006875.2	1.392	1.963	4.868	3.750	2.395	9.282	0.708	BAG family molecular chaperone regulator 1-like	GI-H
MELO3C006898.2	0.378	0.283	4.079	NA	0.433	0.105	0.120	Methyl-CpG-binding domain-containing 13-like protein	GI-H
MELO3C006911.2	0.140	0.130	1.459	0.280	0.262	0.094	0.247	Zf-CCHC domain-containing protein/Cofilin_ADF domain-containing protein	GI-H
MELO3C006920.2	1.201	1.107	4.044	0.780	1.348	0.657	1.877	Kinetochore protein Ndc80	GI-H
MELO3C006922.2	12.881	14.372	124.721	17.510	15.063	5.122	16.573	60S ribosomal protein L36	GI-H
MELO3C031824.2	0.598	0.510	1.304	0.719	0.612	2.008	0.566	DUF4283 domain-containing protein	GI-H
MELO3C006940.2	4.735	8.978	12.572	6.248	5.325	13.503	4.109	MADS-box protein SOC1	GI-H
MELO3C006941.2	0.423	0.400	5.122	0.385	0.644	0.441	0.452	At4g25660	GI-H
MELO3C006948.2	3.251	4.587	16.840	4.726	5.859	4.082	4.656	Protein SGT1 homolog	GI-H
MELO3C006950.2	1.023	0.705	4.029	0.776	1.522	0.595	0.659	Pentatricopeptide repeat-containing protein At4g21190	GI-H
MELO3C006952.2	1.620	2.274	4.401	1.337	1.891	1.170	1.683	Pentatricopeptide repeat (PPR) superfamily protein	GI-H
MELO3C006980.2	0.496	0.900	4.687	0.615	1.470	0.411	1.125	cyclic nucleotide-gated ion channel 1	GI-H

Gene ID	FPKM							Gene Description	Specific in episode
	FS	GI-M	GM-M	AN-M	GI-H	GM-H	AN-H		
MELO3C026931.2	0.342	1.238	9.335	0.728	1.261	6.599	0.479	RNA polymerase sigma factor	GI-H
MELO3C026935.2	0.189	NA	2.664	NA	0.094	NA	0.075	Homeobox-leucine zipper HOX24	GI-H
MELO3C008491.2	0.534	1.650	1.973	2.897	0.635	0.582	0.377	Zinc finger protein CONSTANS	GI-H
MELO3C008498.2	1.709	1.384	6.734	0.769	3.311	0.185	2.425	Defective in meristem silencing 3	GI-H
MELO3C008499.2	0.330	0.389	1.889	0.970	0.108	0.636	0.552	Protein IQ-DOMAIN 1	GI-H
MELO3C008503.2	1.079	0.362	4.170	1.161	0.619	0.240	0.933	Protein ABIL2	GI-H
MELO3C019371.2	1.811	1.922	5.187	1.949	2.190	0.879	2.184	KH domain-containing protein At4g18375	GI-H
MELO3C019382.2	1.293	2.418	8.104	2.872	2.058	1.477	1.777	Rhodanese-like domain containing protein	GI-H
MELO3C031894.2	0.353	0.205	3.215	NA	0.275	1.830	0.271	Serine/threonine-protein kinase TOR	GI-H
MELO3C019389.2	7.099	12.842	19.484	10.946	8.642	42.460	7.848	transcription elongation factor 1 homolog	GI-H
MELO3C019407.2	0.504	0.776	1.886	0.206	0.605	0.089	0.828	2,3-diketo-5-methylthio-1-phosphopentane phosphatase	GI-H
MELO3C019423.2	0.423	0.393	1.011	0.263	0.402	0.250	0.380	ATP-dependent RNA helicase, putative	GI-H
MELO3C031917.2	0.382	0.624	1.850	0.432	0.584	0.437	0.125	Unknown protein	GI-H
MELO3C019429.2	0.891	1.033	3.036	1.194	0.462	3.521	1.213	Basic-leucine zipper (BZIP) transcription factor family protein	GI-H
MELO3C019433.2	2.977	3.565	9.493	1.908	3.259	5.244	2.635	dnaJ homolog subfamily C member 2-like	GI-H
MELO3C019447.2	0.512	0.598	3.255	0.376	0.735	0.164	0.540	ATP-dependent DNA helicase Q-like 4A isoform X1	GI-H
MELO3C019462.2	0.397	0.713	2.908	0.582	0.512	1.214	0.412	Membralin, putative	GI-H
MELO3C019488.2	0.664	1.204	2.449	0.457	1.212	0.646	1.068	Pentatricopeptide repeat-containing protein	GI-H
MELO3C019491.2	0.770	1.053	5.176	0.518	1.264	0.130	1.022	Nucleolar complex protein 2 homolog	GI-H
MELO3C019495.2	1.767	4.968	13.440	7.821	3.542	5.309	3.074	cytochrome b-c1 complex subunit 6	GI-H
MELO3C019502.2	0.712	1.137	2.949	0.368	0.779	0.173	0.454	B3 domain-containing protein Os01g0234100-like isoform X1	GI-H
MELO3C019522.2	0.578	0.786	4.818	1.145	1.363	0.750	1.357	Exocyst complex component	GI-H
MELO3C019537.2	1.015	1.140	12.488	1.854	2.996	1.227	4.579	Leucine-rich repeat receptor-like protein kinase family	GI-H
MELO3C019546.2	1.247	1.479	4.954	1.102	1.924	0.666	1.326	PRKR-interacting protein 1	GI-H
MELO3C019554.2	0.511	0.740	2.584	0.597	0.485	1.034	0.576	pathogenesis-related homeodomain protein	GI-H
MELO3C019559.2	0.332	0.853	2.714	0.178	0.868	0.240	0.535	Mitogen-activated protein kinase 12	GI-H
MELO3C032004.2	0.785	1.295	3.179	0.925	0.931	0.991	1.035	Unknown protein	GI-H
MELO3C019568.2	0.641	0.975	4.811	0.982	0.705	1.434	0.694	ALA-interacting subunit 3	GI-H
MELO3C019573.2	1.115	0.975	4.293	0.976	1.050	0.330	0.940	transcription repressor KAN1-like isoform X1	GI-H
MELO3C020334.2	0.567	0.860	3.015	0.767	0.649	0.892	0.723	Methionine aminopeptidase 2	GI-H
MELO3C031718.2	0.391	0.391	1.738	0.265	0.508	0.055	0.363	Kinesin light chain	GI-H

Gene ID	FPKM							Gene Description	Specific in episode
	FS	GI-M	GM-M	AN-M	GI-H	GM-H	AN-H		
MELO3C020320.2	1.706	2.009	5.436	1.721	2.380	1.303	2.276	elongation factor Ts, mitochondrial	GI-H
MELO3C020300.2	0.797	0.921	2.107	0.399	0.951	0.433	0.761	AAR2 family protein	GI-H
MELO3C020298.2	1.189	1.204	3.520	0.704	1.377	0.880	1.609	Gamma-tubulin complex component, putative	GI-H
MELO3C020253.2	1.301	1.120	2.879	1.109	1.406	0.925	1.191	importin-11	GI-H
MELO3C032061.2	0.663	0.815	2.324	1.233	0.911	0.945	0.877	Unknown protein	GI-H
MELO3C023776.2	0.727	1.107	4.905	1.169	0.881	1.224	0.909	Laminin subunit gamma-1	GI-H
MELO3C023782.2	0.417	0.283	1.844	0.206	0.535	0.177	0.432	Zinc finger (C3HC4-type RING finger) family protein / BRCT domain-containing protein	GI-H
MELO3C023794.2	0.423	0.416	1.065	0.416	0.375	0.251	0.438	Alpha-1,3-glucosyltransferase	GI-H
MELO3C032096.2	0.237	0.186	2.177	0.273	0.248	0.218	0.415	Retrovirus-related Pol polyprotein from transposon TNT 1-94	GI-H
MELO3C014944.2	2.250	4.140	5.292	8.312	1.249	5.678	0.876	homeobox-leucine zipper protein HAT22-like	GI-H
MELO3C014939.2	1.757	1.758	4.495	1.706	1.463	2.419	1.519	Meiosis chromosome segregation family protein	GI-H
MELO3C014921.2	0.227	0.266	1.008	NA	0.451	0.144	0.484	Pentatricopeptide repeat-containing family protein	GI-H
MELO3C014907.2	3.554	3.652	28.158	2.108	4.896	NA	3.064	Pollen Ole e 1 allergen and extensin family protein	GI-H
MELO3C014906.2	15.922	22.682	136.372	18.343	25.422	5.977	21.930	60S ribosomal protein L7a	GI-H
MELO3C014883.2	13.403	16.197	36.401	5.863	10.767	18.289	7.550	AT3g27090/MOJ10_18	GI-H
MELO3C014877.2	0.968	0.701	2.467	1.238	1.033	0.570	1.153	DNA/RNA-binding protein KIN17	GI-H
MELO3C014857.2	0.656	3.432	2.307	10.710	0.767	5.313	0.664	Lysine histidine transporter	GI-H
MELO3C014847.2	0.197	0.407	2.022	0.241	0.811	0.517	0.518	MYB-related transcription factor	GI-H
MELO3C014838.2	0.423	0.911	3.120	0.831	0.808	0.780	0.913	DUF1713 domain protein	GI-H
MELO3C014817.2	0.980	2.072	7.197	1.285	1.902	3.063	1.684	RING finger protein 10 isoform X1	GI-H
MELO3C014774.2	0.459	0.684	2.147	0.430	0.702	0.847	0.604	Aminopeptidase	GI-H
MELO3C031852.2	7.797	7.992	60.859	13.152	8.746	5.812	6.357	Protein Ycf2	GI-H
MELO3C014759.2	1.147	1.923	3.622	3.006	1.439	7.842	0.614	Avr9/Cf-9 rapidly elicited protein	GI-H
MELO3C014753.2	0.374	0.391	1.108	0.369	0.293	0.339	0.265	OTU-like cysteine protease family protein	GI-H
MELO3C031863.2	0.136	0.137	1.057	0.156	0.129	NA	0.127	Calcium uniporter protein, mitochondrial	GI-H
MELO3C031870.2	2.097	3.012	10.603	3.358	3.826	0.754	2.219	50S ribosomal protein L24, chloroplastic	GI-H
MELO3C032187.2	0.127	0.274	3.573	0.231	0.766	NA	0.405	dihydroflavonol-4-reductase-like	GI-H
MELO3C016579.2	0.285	0.595	1.919	0.454	0.607	0.139	0.568	Serine/threonine-protein kinase	GI-H
MELO3C016574.2	1.023	2.672	2.564	0.348	1.221	0.511	1.213	Serine/threonine-protein kinase	GI-H
MELO3C016573.2	0.129	0.278	1.141	NA	0.257	0.130	0.333	Serine/threonine-protein kinase	GI-H

Gene ID	FPKM							Gene Description	Specific in episode
	FS	GI-M	GM-M	AN-M	GI-H	GM-H	AN-H		
MELO3C016568.2	0.416	0.914	1.455	0.464	0.723	2.085	0.499	Diacylglycerol kinase	GI-H
MELO3C032193.2	0.807	1.133	6.395	0.943	0.825	0.575	1.200	Diacylglycerol kinase	GI-H
MELO3C016554.2	0.917	1.495	7.784	0.470	1.241	0.725	0.400	Triosephosphate isomerase	GI-H
MELO3C016536.2	3.229	15.716	46.441	0.999	0.480	9.235	0.841	NAC domain-containing protein 55	GI-H
MELO3C016520.2	0.716	1.041	4.545	0.707	0.736	0.636	0.903	Nucleic acid-binding protein	GI-H
MELO3C016513.2	4.982	16.091	23.339	16.904	0.957	63.535	1.940	Metallothionein	GI-H
MELO3C016512.2	1.875	3.057	5.638	2.478	0.865	6.581	0.621	Cytochrome c oxidase copper chaperone	GI-H
MELO3C016499.2	0.640	1.393	5.986	0.594	1.583	NA	1.272	Multidrug resistance 3	GI-H
MELO3C016491.2	1.639	2.527	12.692	2.764	2.644	2.715	2.806	ras-related protein RABA2a	GI-H
MELO3C016488.2	0.278	0.430	1.759	0.836	0.329	0.648	0.469	Transmembrane protein, putative	GI-H
MELO3C016486.2	3.898	4.088	20.389	3.379	3.351	NA	2.550	Ras-related GTP-binding protein	GI-H
MELO3C016482.2	0.247	0.237	1.221	0.138	0.240	0.020	0.283	Zinc finger CCCH domain protein	GI-H
MELO3C016474.2	0.444	0.377	2.058	0.226	0.484	0.145	0.341	guanine nucleotide-binding protein-like NSN1	GI-H
MELO3C016469.2	0.802	0.404	3.752	0.394	0.706	0.311	0.292	zinc finger CCCH domain-containing protein 5	GI-H
MELO3C016456.2	0.633	0.409	1.424	0.380	0.372	0.430	0.482	snRNA-activating protein complex subunit 4	GI-H
MELO3C016434.2	3.329	2.032	9.088	2.164	3.385	2.240	3.370	protein STICHEL	GI-H
MELO3C016417.2	0.686	0.614	3.203	0.667	0.432	1.356	0.592	protein NETWORKED 2B	GI-H
MELO3C025369.2	0.603	1.740	3.854	1.014	0.739	0.367	0.262	Reactive Intermediate Deaminase A, chloroplastic	GI-H
MELO3C025374.2	7.990	9.135	21.232	6.237	9.641	10.880	9.461	calcium-dependent protein kinase-like	GI-H
MELO3C025375.2	3.725	8.950	8.065	5.977	3.327	22.821	2.692	Protein translation factor sui1-like protein	GI-H
MELO3C025378.2	0.481	0.651	1.963	0.366	0.447	0.791	0.875	Maturase-like protein	GI-H
MELO3C025405.2	0.211	1.993	2.127	2.465	0.642	5.936	0.595	early nodulin-like protein 3	GI-H
MELO3C025417.2	1.581	5.017	6.755	10.648	2.091	41.402	1.697	Polyubiquitin	GI-H
MELO3C013702.2	0.445	0.529	3.743	0.429	0.886	0.522	0.798	kinesin-like calmodulin-binding protein	GI-H
MELO3C013723.2	8.610	12.570	94.033	15.268	11.574	10.485	10.420	High mobility group B protein 2	GI-H
MELO3C013726.2	0.131	0.277	1.279	0.849	0.140	1.799	0.077	RB1-inducible coiled-coil protein	GI-H
MELO3C013733.2	0.955	1.911	8.561	1.569	1.539	1.790	1.148	protein GRIP isoform X1	GI-H
MELO3C013750.2	0.338	0.472	1.358	NA	0.295	0.636	0.294	SKP1-like protein 1B	GI-H
MELO3C013771.2	0.558	0.447	6.029	0.354	0.647	NA	0.444	beta-carotene isomerase D27, chloroplastic	GI-H
MELO3C013773.2	1.920	4.397	17.865	2.502	1.761	4.838	1.449	Mediator of RNA polymerase II transcription subunit	GI-H

Gene ID	FPKM							Gene Description	Specific in episode
	FS	GI-M	GM-M	AN-M	GI-H	GM-H	AN-H		
MELO3C013775.2	3.243	3.764	9.453	4.064	4.687	3.062	4.636	UDP-N-acetylglucosamine--peptide N-acetylglucosaminyltransferase subunit	GI-H
MELO3C031937.2	2.494	3.685	62.976	14.396	6.799	5.419	6.372	acyl-CoA-binding protein	GI-H
MELO3C013801.2	1.253	1.288	6.025	0.675	1.804	0.842	1.967	protein PAM68, chloroplastic isoform X1	GI-H
MELO3C013805.2	1.167	1.169	3.700	0.559	1.755	0.575	1.780	At1g80480	GI-H
MELO3C013806.2	5.136	3.426	10.622	2.123	4.770	0.602	4.543	Cytosine-specific methyltransferase	GI-H
MELO3C013811.2	0.866	0.731	15.184	NA	0.684	0.330	0.889	Phosphoribulokinase	GI-H
MELO3C013815.2	3.266	3.589	13.153	3.199	4.284	5.439	3.677	Extra-large guanine nucleotide binding family protein	GI-H
MELO3C013816.2	0.253	0.643	8.400	0.941	1.170	0.414	0.956	Vacuolar protein sorting-associated protein 25	GI-H
MELO3C013823.2	0.193	0.118	1.636	NA	0.215	NA	0.168	Survival motor neuron protein	GI-H
MELO3C013824.2	5.644	7.681	18.562	4.524	8.634	9.345	7.008	inactive poly [ADP-ribose] polymerase RCD1-like	GI-H
MELO3C013825.2	0.602	0.909	3.005	0.450	0.712	1.100	0.660	Shikimate kinase	GI-H
MELO3C013831.2	0.062	0.207	5.089	NA	0.286	0.664	0.232	Envelope glycoprotein	GI-H
MELO3C013841.2	2.334	2.266	7.084	2.359	1.588	3.050	2.121	nucleolar protein 12	GI-H
MELO3C013860.2	0.445	0.447	3.377	0.467	1.078	NA	0.655	eyes absent homolog 2	GI-H
MELO3C013867.2	0.426	3.725	3.756	2.072	0.947	1.055	0.396	Cytochrome P450 family ent-kaurenoic acid oxidase	GI-H
MELO3C013872.2	0.645	1.078	2.936	NA	0.519	6.844	0.467	Type I inositol polyphosphate 5-phosphatase, putative	GI-H
MELO3C013889.2	0.373	0.225	5.376	0.748	0.634	0.316	0.649	Novel plant snare, putative	GI-H
MELO3C013898.2	1.640	1.299	4.079	0.486	1.806	0.134	1.518	tRNA/rRNA methyltransferase, SpoU	GI-H
MELO3C013901.2	0.840	0.863	2.039	0.615	0.639	0.913	0.644	histone-lysine N-methyltransferase, H3 lysine-9 specific SUVH6-like	GI-H
MELO3C013910.2	3.343	3.365	7.186	8.266	3.361	16.829	3.459	Ras-related GTP-binding protein	GI-H
MELO3C013937.2	0.468	0.596	2.285	0.341	0.408	0.573	0.286	Homeodomain-like superfamily protein	GI-H
MELO3C013942.2	1.088	0.941	7.061	0.504	1.957	0.518	1.341	Chaperone protein dnaJ 13	GI-H
MELO3C013961.2	0.830	1.434	6.832	1.310	2.954	2.736	2.786	Oxysterol-binding protein-related protein 2A	GI-H
MELO3C031950.2	1.472	2.583	14.165	5.192	4.207	1.163	6.998	Unknown protein	GI-H
MELO3C032275.2	1.485	1.719	8.440	2.560	2.457	NA	2.343	protein YABBY 4-like	GI-H
MELO3C013977.2	0.503	1.315	4.398	1.617	0.895	0.952	1.067	Copper ion-binding protein, putative	GI-H
MELO3C032274.2	0.136	0.226	1.007	0.150	0.094	0.133	0.183	HAT transposon superfamily	GI-H
MELO3C013989.2	1.493	1.403	4.483	1.187	0.987	1.099	0.721	IRK-interacting protein	GI-H
MELO3C014001.2	1.998	3.199	5.867	2.941	1.627	3.385	1.665	TVP38/TMEM64 family membrane protein	GI-H
MELO3C014003.2	1.480	1.882	7.507	0.449	2.155	0.103	1.440	protein TPX2 isoform X1	GI-H

Gene ID	FPKM							Gene Description	Specific in episode
	FS	GI-M	GM-M	AN-M	GI-H	GM-H	AN-H		
MELO3C014008.2	0.372	0.251	4.975	0.315	0.419	NA	0.308	Adenosine monophosphate-protein transferase and cysteine protease ibpA	GI-H
MELO3C014016.2	0.499	1.607	2.757	NA	0.964	0.400	0.477	Phospholipase D	GI-H
MELO3C014018.2	0.585	0.817	3.247	0.379	0.677	0.402	0.578	Pentatricopeptide repeat-containing protein family	GI-H
MELO3C014056.2	1.682	2.019	15.110	0.634	4.247	0.326	3.107	GRAS family transcription factor	GI-H
MELO3C014063.2	3.377	4.457	13.648	2.774	4.521	6.128	4.122	Elongation factor G	GI-H
MELO3C014069.2	4.486	5.837	14.935	6.337	6.983	12.291	4.367	Calcineurin B, putative	GI-H
MELO3C014099.2	1.174	2.472	7.283	NA	1.678	2.765	2.437	BnaC03g71690D protein	GI-H
MELO3C014102.2	0.528	0.412	6.653	0.225	0.271	0.131	0.305	LOW QUALITY PROTEIN: eukaryotic translation initiation factor 5B-like	GI-H
MELO3C014108.2	0.244	0.641	1.592	0.683	0.791	0.748	0.596	Coiled-coil protein	GI-H
MELO3C014111.2	0.525	0.686	2.486	0.322	0.819	0.064	0.763	DNA-directed RNA polymerase family protein	GI-H
MELO3C014112.2	0.528	1.086	4.261	1.227	0.852	0.944	1.097	Sec14p-like phosphatidylinositol transfer family protein	GI-H
MELO3C014122.2	0.899	1.662	13.195	1.139	1.567	2.509	1.711	protein CWC15 homolog	GI-H
MELO3C014128.2	0.387	1.539	5.145	0.544	0.505	0.594	0.306	Calcium-dependent protein kinase	GI-H
MELO3C014130.2	1.444	1.736	9.850	0.742	3.202	NA	4.067	RNA polymerase II elongation factor ELL3 isoform 1	GI-H
MELO3C014131.2	0.222	1.045	1.336	NA	0.265	0.050	0.605	zinc finger CCCH domain-containing protein 18-like	GI-H
MELO3C014134.2	1.428	1.177	3.687	1.598	1.835	0.547	1.769	WRKY transcription factor, putative	GI-H
MELO3C014139.2	1.059	1.626	4.074	2.909	1.059	4.596	1.699	heterogeneous nuclear ribonucleoprotein 1	GI-H
MELO3C014142.2	1.837	1.052	14.169	0.788	3.536	0.280	3.934	kinesin-like protein KIN12B	GI-H
MELO3C014150.2	1.550	2.645	5.193	2.453	1.882	8.542	1.534	Fimbrin, putative	GI-H
MELO3C014153.2	1.052	0.824	12.906	1.152	0.896	1.303	0.676	DNA topoisomerase 1-like	GI-H
MELO3C014154.2	0.546	0.613	2.613	NA	0.500	0.259	0.539	Syntaxin-51	GI-H
MELO3C014173.2	2.785	2.671	39.253	3.185	4.571	0.309	4.799	65-kDa microtubule-associated protein 1	GI-H
MELO3C014179.2	1.082	1.491	10.798	1.003	1.389	0.261	1.501	GrpE protein homolog	GI-H
MELO3C014183.2	2.016	3.277	12.275	2.044	3.345	2.335	2.829	Small ubiquitin-related modifier	GI-H
MELO3C014189.2	0.270	0.627	5.090	0.516	1.051	NA	1.286	aspartate carbamoyltransferase, chloroplastic	GI-H
MELO3C017077.2	1.113	1.422	3.271	1.151	1.579	2.188	1.415	Chaperone protein dnaJ 10	GI-H
MELO3C017066.2	1.968	1.976	16.346	NA	6.662	NA	4.114	mitotic spindle checkpoint protein BUBR1	GI-H
MELO3C017041.2	0.780	0.402	2.267	0.306	0.524	0.131	0.314	Protein canopy 1-like	GI-H
MELO3C017038.2	0.896	1.383	3.458	0.647	1.139	0.719	0.908	Pentatricopeptide repeat-containing family protein	GI-H
MELO3C017032.2	1.818	2.524	9.927	1.090	3.077	0.401	3.193	WUSCHEL-related homeobox 8-like	GI-H

Gene ID	FPKM							Gene Description	Specific in episode
	FS	GI-M	GM-M	AN-M	GI-H	GM-H	AN-H		
MELO3C017031.2	83.914	81.553	221.643	64.891	82.601	184.685	92.797	High mobility group B protein 2	GI-H
MELO3C017004.2	0.787	0.670	4.260	1.004	1.580	0.321	1.052	Pseudouridine synthase family protein	GI-H
MELO3C016991.2	0.520	1.257	1.745	0.698	0.475	3.163	0.491	Octicosapeptide/Phox/Bem1p (PB1) domain-containing protein / tetratricopeptide repeat (TPR)-containing protein	GI-H
MELO3C016981.2	2.633	1.459	35.565	10.389	7.619	1.844	10.361	60S ribosomal protein L39	GI-H
MELO3C016973.2	2.171	2.674	10.398	1.836	3.158	1.321	2.586	CHD3-type chromatin-remodeling factor PICKLE	GI-H
MELO3C016970.2	2.111	1.981	6.021	1.711	2.575	2.739	2.149	Chaperone protein	GI-H
MELO3C032315.2	0.691	0.422	3.529	NA	0.252	0.295	0.240	Pentatricopeptide repeat-containing protein, chloroplastic	GI-H
MELO3C016950.2	0.139	0.121	5.282	0.117	0.304	NA	0.309	Myb/SANT-like DNA-binding domain protein	GI-H
MELO3C016944.2	0.438	0.592	1.537	1.538	0.745	0.692	0.768	protein TIC 55, chloroplastic	GI-H
MELO3C016928.2	0.529	1.059	2.140	0.554	0.594	0.864	0.991	Protein ROOT PRIMORDIUM DEFECTIVE 1	GI-H
MELO3C016919.2	0.697	1.248	9.171	0.646	1.124	0.138	0.655	Guanine nucleotide binding protein (G-protein), alpha subunit	GI-H
MELO3C016918.2	1.677	2.740	6.074	2.069	2.412	4.281	1.279	fatty-acid-binding protein 2-like	GI-H
MELO3C016903.2	4.322	6.047	10.205	1.648	3.767	1.348	3.234	Protein BIG GRAIN 1-like B	GI-H
MELO3C016893.2	0.460	0.545	2.313	0.411	0.437	0.173	0.266	(S)-coclaurine N-methyltransferase-like	GI-H
MELO3C016892.2	0.881	1.475	6.674	1.603	1.246	0.537	0.870	(S)-coclaurine N-methyltransferase	GI-H
MELO3C016889.2	2.144	3.011	10.285	2.121	2.426	2.001	2.543	Protein kinase	GI-H
MELO3C016887.2	1.463	1.868	4.346	0.757	1.531	0.983	1.316	Peptidyl-prolyl cis-trans isomerase, putative	GI-H
MELO3C016877.2	1.430	2.117	3.075	1.986	1.156	0.812	0.801	Beta-fructofuranosidase, insoluble isoenzyme CWINV1	GI-H
MELO3C016876.2	0.442	0.737	6.855	0.955	1.603	0.337	2.034	Zinc finger family protein	GI-H
MELO3C016856.2	0.639	1.468	4.948	0.711	2.250	0.694	1.317	homeobox protein 4-like	GI-H
MELO3C016850.2	0.324	0.171	1.334	0.314	0.101	0.127	0.121	homeobox protein knotted-1-like 2	GI-H
MELO3C016840.2	0.705	0.483	7.624	NA	0.508	NA	0.637	AP2-like ethylene-responsive transcription factor	GI-H
MELO3C016829.2	2.147	2.798	30.674	2.597	4.409	2.641	2.729	serine carboxypeptidase-like 2	GI-H
MELO3C016828.2	2.691	3.170	6.254	1.206	1.695	4.017	1.907	Serine carboxypeptidase, putative	GI-H
MELO3C032312.2	1.786	2.533	4.043	1.117	1.048	1.209	1.263	serine carboxypeptidase-like 7 isoform X1	GI-H
MELO3C016813.2	12.196	15.741	39.328	12.174	12.635	31.531	11.109	Interferon-related developmental regulator family protein	GI-H
MELO3C016796.2	1.073	0.768	3.137	0.582	1.395	0.376	1.531	Cyclin A, putative	GI-H
MELO3C016790.2	1.233	2.286	7.420	0.865	2.089	0.203	1.454	MRG family protein, putative isoform 1	GI-H
MELO3C016758.2	0.774	1.217	5.679	0.612	2.074	1.210	1.516	Trimethylguanosine synthase	GI-H
MELO3C016756.2	3.814	4.545	10.549	3.250	3.634	6.406	4.630	THO complex subunit 5B	GI-H

Gene ID	FPKM							Gene Description	Specific in episode
	FS	GI-M	GM-M	AN-M	GI-H	GM-H	AN-H		
MELO3C016749.2	1.082	1.142	2.456	0.760	1.191	3.098	0.748	Protein WEAK CHLOROPLAST MOVEMENT UNDER BLUE LIGHT 1	GI-H
MELO3C016733.2	0.326	0.738	3.136	1.304	0.428	0.472	0.352	Estradiol 17-beta-dehydrogenase 1	GI-H
MELO3C016718.2	1.317	1.843	6.556	1.644	2.872	1.763	2.593	beta-glucosidase 18-like	GI-H
MELO3C016701.2	1.024	1.803	4.832	0.971	2.002	2.314	1.508	Ubiquitin conjugating enzyme, putative	GI-H
MELO3C016696.2	7.097	7.806	15.099	9.123	6.751	21.564	5.996	PROTON PUMP INTERACTOR 1 family protein	GI-H
MELO3C016676.2	1.379	1.248	7.307	1.993	0.916	2.864	1.658	Peptide methionine sulfoxide reductase	GI-H
MELO3C016668.2	0.603	0.654	1.369	0.575	0.548	1.219	0.474	COP9 signalosome complex subunit 5b	GI-H
MELO3C016667.2	0.630	2.507	2.776	1.243	1.187	0.667	0.908	Random slug protein 5	GI-H
MELO3C032464.2	1.292	1.865	4.248	1.253	1.611	1.074	1.593	Unknown protein	GI-H
MELO3C032351.2	1.957	1.979	5.446	1.158	1.791	1.510	1.971	zinc finger BED domain-containing protein RICESLEEPER 1-like	GI-H
MELO3C025646.2	0.733	1.051	5.268	0.799	2.352	0.703	1.758	DNA polymerase delta subunit 3	GI-H
MELO3C025643.2	1.600	1.724	8.151	1.146	2.166	0.280	2.180	Methylthioribose-1-phosphate isomerase	GI-H
MELO3C025632.2	3.206	3.357	6.535	1.664	2.717	2.818	2.654	Acyl-CoA N-acyltransferase with RING/FYVE/PHD-type zinc finger protein, putative isoform 1	GI-H
MELO3C025631.2	0.335	0.395	3.880	0.492	0.461	0.384	0.183	BnaC04g41920D protein	GI-H
MELO3C025620.2	2.049	3.646	7.412	1.750	2.926	0.838	2.636	RNA exonuclease 4	GI-H
MELO3C025617.2	0.622	0.849	4.368	0.329	1.014	NA	0.801	E3 SUMO-protein ligase MMS21	GI-H
MELO3C025606.2	1.628	3.008	6.598	1.784	1.732	2.013	2.002	Unknown protein	GI-H
MELO3C025602.2	0.425	0.432	3.689	0.341	0.367	0.311	0.349	germinal center kinase 1 isoform X1	GI-H
MELO3C025598.2	0.590	1.435	3.521	1.238	0.913	0.570	0.651	Protein phosphatase 2c	GI-H
MELO3C025593.2	0.510	0.602	1.946	0.209	0.695	0.232	0.734	Pentatricopeptide repeat-containing family protein	GI-H
MELO3C026357.2	0.267	0.644	1.570	0.836	0.290	1.967	0.327	Callose synthase-like protein	GI-H
MELO3C026360.2	3.290	5.258	15.057	7.427	5.909	14.157	4.081	Proteasome subunit alpha type	GI-H
MELO3C026396.2	0.864	1.201	3.557	0.627	1.097	0.763	1.431	carnosine N-methyltransferase-like isoform X2	GI-H
MELO3C026399.2	3.267	3.550	12.257	5.194	4.299	4.634	3.366	Heat shock 70 kDa protein, putative	GI-H
MELO3C026400.2	1.012	1.944	5.470	1.225	2.180	1.798	0.883	OTU-like cysteine protease family protein	GI-H
MELO3C026403.2	0.820	0.754	23.608	1.574	1.187	0.478	1.745	60S ribosomal protein L27	GI-H
MELO3C032529.2	1.355	2.155	9.888	1.073	2.156	0.367	1.914	protein FAR1-RELATED SEQUENCE 2 isoform X3	GI-H
MELO3C026973.2	3.643	5.934	19.973	5.653	6.549	2.478	4.921	60S ribosomal protein L18a	GI-H
MELO3C010540.2	0.815	NA	4.186	3.286	0.255	1.357	0.747	At4g19430	GI-H
MELO3C010535.2	0.599	0.844	2.035	0.598	1.005	0.990	0.706	Coiled-coil domain-containing 73	GI-H

Gene ID	FPKM							Gene Description	Specific in episode
	FS	GI-M	GM-M	AN-M	GI-H	GM-H	AN-H		
MELO3C010521.2	0.548	0.420	2.128	0.626	0.545	0.363	0.407	Zinc finger CCCH domain-containing protein 65	GI-H
MELO3C010520.2	2.620	8.051	35.469	0.141	13.187	0.987	0.239	Chalcone synthase	GI-H
MELO3C010518.2	0.287	0.329	1.191	0.126	0.241	0.224	0.139	Pentatricopeptide repeat-containing protein, putative	GI-H
MELO3C010516.2	0.613	1.166	2.321	0.607	0.706	1.460	0.982	Glutathione S-transferase family protein	GI-H
MELO3C010515.2	0.880	0.718	10.659	2.720	0.387	1.765	0.432	MADS-box transcription factor	GI-H
MELO3C010481.2	0.740	0.799	3.772	0.395	0.845	0.617	0.732	Sister chromatid cohesion PDS5-B-B-like protein	GI-H
MELO3C010476.2	0.525	0.828	3.212	1.069	0.811	0.774	0.407	acetyltransferase isoform X1	GI-H
MELO3C010475.2	0.886	1.857	4.474	1.776	2.056	1.031	1.532	UDP-glucuronosyltransferase 2B17	GI-H
MELO3C010472.2	0.657	1.085	4.138	0.482	0.668	0.689	0.677	p-loop nucleoside triphosphate hydrolase superfamily protein, putative	GI-H
MELO3C010456.2	3.738	4.582	8.841	1.726	3.820	1.445	3.787	Phosphatidylinositol-4-phosphate 5-kinase, putative	GI-H
MELO3C010434.2	0.518	1.356	6.163	1.031	0.932	1.393	0.778	Hexosyltransferase	GI-H
MELO3C010421.2	0.256	0.185	2.458	0.547	0.365	0.111	0.218	Random slug protein 5	GI-H
MELO3C032655.2	0.355	0.355	1.456	0.590	0.622	0.313	0.350	BEACH domain-containing protein	GI-H
MELO3C010379.2	0.950	0.936	4.596	0.631	1.474	0.374	1.253	Stress response protein	GI-H
MELO3C010371.2	0.367	0.351	2.092	0.745	0.310	3.557	0.231	transcription factor TCP17-like isoform X1	GI-H
MELO3C032683.2	0.535	0.340	1.519	0.316	0.630	0.187	0.603	aldo-keto reductase family 4 member C9-like	GI-H
MELO3C032454.2	0.349	0.373	4.464	0.655	0.863	0.354	0.685	PHD finger protein ALFIN-LIKE 3-like isoform X1	GI-H
MELO3C026307.2	0.604	1.057	2.179	2.510	0.150	5.008	0.167	protein COBRA-like	GI-H
MELO3C026300.2	1.406	1.860	12.939	2.064	1.984	3.713	1.380	MADS-box transcription factor	GI-H
MELO3C026299.2	0.533	0.451	1.874	0.336	0.176	NA	0.189	MADS-box transcription factor	GI-H
MELO3C018912.2	0.213	0.325	2.172	0.163	0.186	0.302	0.246	Wpp domain-interacting tail-anchored protein 2	GI-H
MELO3C018919.2	2.057	2.368	10.483	3.242	3.185	1.149	1.927	Hexosyltransferase	GI-H
MELO3C018930.2	0.233	0.172	1.421	0.291	0.282	1.792	0.163	zinc finger protein CONSTANS-LIKE 2	GI-H
MELO3C018937.2	0.379	0.481	2.301	NA	0.497	0.314	0.463	RHOMBOID-like protein 9, chloroplastic isoform X1	GI-H
MELO3C018942.2	0.515	0.492	3.883	0.516	0.930	0.094	1.030	Zinc-finger domain of monoamine-oxidase A repressor R1 protein	GI-H
MELO3C018948.2	2.342	3.660	7.713	8.627	3.079	5.043	3.205	Alpha-1,4 glucan phosphorylase	GI-H
MELO3C018954.2	1.342	4.122	6.702	2.159	1.973	0.928	2.031	50S ribosomal protein L29, chloroplastic	GI-H
MELO3C018956.2	0.543	1.003	4.808	0.814	0.917	1.638	0.991	Ubiquitin carboxyl-terminal hydrolase, putative	GI-H
MELO3C018993.2	0.721	0.883	2.271	0.545	1.058	0.683	0.627	dCTP pyrophosphatase 1-like	GI-H
MELO3C019002.2	0.765	1.987	11.458	0.860	3.415	0.621	2.083	Annexin	GI-H

Gene ID	FPKM							Gene Description	Specific in episode
	FS	GI-M	GM-M	AN-M	GI-H	GM-H	AN-H		
MELO3C019019.2	1.051	1.436	11.988	0.961	1.353	1.683	1.466	Protein LNK1	GI-H
MELO3C019020.2	0.350	0.373	1.715	0.143	0.243	0.234	0.259	pentatricopeptide repeat-containing protein At4g33990	GI-H
MELO3C019021.2	0.514	1.027	3.282	0.731	1.014	1.232	0.551	Endoplasmic reticulum-Golgi intermediate compartment 3	GI-H
MELO3C019036.2	0.744	0.935	2.600	0.712	0.786	0.653	0.750	Alpha-amylase, putative	GI-H
MELO3C024866.2	1.459	2.775	7.997	1.878	2.054	4.049	2.168	Calcium-binding endonuclease/exonuclease/phosphatase family	GI-H
MELO3C024862.2	1.310	1.390	2.901	0.873	1.253	2.349	0.990	Plant peroxidase	GI-H
MELO3C024845.2	0.842	0.862	2.887	0.450	0.978	0.365	0.906	DNA mismatch repair protein MutS2-like	GI-H
MELO3C024839.2	1.620	2.494	10.806	0.971	2.322	0.881	1.990	transcription factor bHLH143-like isoform X1	GI-H
MELO3C016073.2	0.480	0.846	3.003	0.545	0.646	1.101	0.734	Tudor/PWWP/MBT superfamily protein	GI-H
MELO3C016082.2	21.290	28.866	54.507	29.144	25.397	19.775	21.333	Ubiquitin	GI-H
MELO3C016083.2	47.959	68.938	212.627	76.211	71.964	24.365	58.635	Ubiquitin	GI-H
MELO3C016085.2	1.969	3.015	4.818	2.202	2.062	8.284	2.381	E3 ubiquitin-protein ligase Arkadia	GI-H
MELO3C016086.2	0.313	0.544	1.024	0.268	0.480	0.247	0.452	F12M16.11	GI-H
MELO3C016094.2	0.651	0.358	3.313	0.426	0.617	0.337	0.384	Transcription factor GTE4	GI-H
MELO3C016108.2	1.807	1.403	6.151	1.623	1.656	2.151	1.963	Receptor-like protein kinase	GI-H
MELO3C016123.2	1.998	2.948	11.595	2.413	4.907	1.082	4.231	NHL domain-containing protein	GI-H
MELO3C016132.2	16.032	18.880	35.549	23.037	11.217	25.552	12.230	Ubiquitin-conjugating enzyme E2	GI-H
MELO3C016135.2	25.726	69.411	53.024	6.209	10.331	13.378	12.348	Serine/threonine-protein kinase	GI-H
MELO3C016152.2	10.323	8.725	32.058	NA	1.211	0.454	1.568	Gibberellin 2-oxidase	GI-H
MELO3C016154.2	1.672	1.327	4.455	1.172	1.294	3.392	1.375	protein SIP5	GI-H
MELO3C032563.2	3.061	7.054	18.033	2.196	2.242	0.908	1.439	Glutathione S-transferase	GI-H
MELO3C016167.2	0.327	1.872	5.483	1.715	2.207	3.134	1.181	Glutathione S-transferase	GI-H
MELO3C016173.2	3.035	0.938	9.305	0.503	4.213	NA	3.440	kinesin-like calmodulin-binding protein	GI-H
MELO3C016178.2	3.667	4.780	14.625	3.459	5.436	2.271	5.520	DnaJ subfamily C member 14	GI-H
MELO3C016179.2	0.393	0.658	3.817	0.327	0.847	0.377	0.191	Receptor-like kinase	GI-H
MELO3C016183.2	0.625	1.057	3.767	1.123	0.882	1.602	0.677	Pleckstrin homology domain-containing family protein	GI-H
MELO3C032805.2	2.222	1.904	8.681	3.553	3.719	0.724	3.122	Unknown protein	GI-H
MELO3C016203.2	0.456	1.043	17.783	2.092	1.485	7.143	1.376	Myosin-related family protein	GI-H
MELO3C016211.2	2.368	1.561	6.117	1.111	2.622	1.437	2.300	Lipase, GDSL	GI-H
MELO3C016215.2	0.273	0.508	3.873	0.480	0.371	0.323	0.472	Transposase	GI-H

Gene ID	FPKM							Gene Description	Specific in episode
	FS	GI-M	GM-M	AN-M	GI-H	GM-H	AN-H		
MELO3C016234.2	0.786	1.334	13.553	NA	0.823	0.918	0.537	FAR1	GI-H
MELO3C032585.2	0.463	0.627	6.827	0.607	0.561	0.883	0.508	protein FAR-RED ELONGATED HYPOCOTYL 3	GI-H
MELO3C016242.2	0.237	0.244	1.894	0.235	0.141	0.238	0.135	Pentatricopeptide repeat-containing family protein	GI-H
MELO3C016273.2	8.626	17.956	37.464	12.922	6.207	30.342	4.296	BnaC09g01330D protein	GI-H
MELO3C016286.2	0.774	0.465	3.455	0.499	0.331	0.385	0.273	At1g64385	GI-H
MELO3C016306.2	0.547	0.508	13.505	0.119	1.610	NA	1.096	high mobility group B protein 6	GI-H
MELO3C016308.2	0.232	0.759	4.800	0.557	0.421	0.943	0.416	myosin-11-like	GI-H
MELO3C016314.2	0.978	1.063	3.751	NA	1.130	0.862	1.553	Aldose 1-epimerase, putative	GI-H
MELO3C016319.2	0.413	0.335	1.494	0.338	0.228	0.510	0.485	Ribonucleases P/MRP protein subunit POP1	GI-H
MELO3C016321.2	1.399	1.048	4.439	0.762	1.257	0.396	1.037	multiple RNA-binding domain-containing protein 1	GI-H
MELO3C016335.2	2.837	1.896	6.079	1.776	1.653	1.970	2.166	F22C12.19	GI-H
MELO3C016344.2	2.111	2.489	7.557	3.691	2.783	6.195	2.059	Transcription initiation factor IIE subunit beta	GI-H
MELO3C016354.2	2.036	2.534	6.154	1.464	2.218	0.787	1.274	Short-chain dehydrogenase TIC 32, chloroplastic	GI-H
MELO3C016363.2	0.218	0.391	1.405	0.287	0.321	0.063	0.231	AT-rich interactive domain-containing protein 2	GI-H
MELO3C016372.2	1.803	1.672	4.148	2.259	1.111	1.349	1.669	Protein chaperone-like protein of por1, chloroplastic	GI-H
MELO3C016393.2	0.719	0.749	12.203	NA	0.914	0.896	0.869	Polyamine-modulated factor 1-binding protein	GI-H
MELO3C016397.2	1.166	1.548	3.474	0.570	0.928	0.400	1.274	GTP-binding protein era, putative	GI-H
MELO3C016404.2	0.443	0.650	3.035	0.778	0.641	0.586	0.791	50S ribosomal protein L2	GI-H
MELO3C016411.2	0.727	1.068	2.280	0.749	0.767	0.931	0.880	Protein MICRORCHIDIA 4	GI-H
MELO3C017537.2	6.044	5.826	14.618	5.659	4.811	25.983	4.699	zinc finger CCCH domain-containing protein 44-like isoform X2	GI-H
MELO3C017544.2	1.647	2.475	7.158	1.418	2.698	1.280	2.615	DNA-directed RNA polymerase III subunit RPC5	GI-H
MELO3C017551.2	2.411	3.060	5.837	1.371	2.696	1.555	2.800	Pentatricopeptide repeat-containing protein	GI-H
MELO3C017558.2	0.854	0.880	10.491	0.454	1.477	0.386	1.728	ATP-dependent DNA helicase DDM1	GI-H
MELO3C017567.2	3.136	2.699	14.892	1.753	2.942	0.828	2.284	ATP/GTP binding protein-like	GI-H
MELO3C017576.2	1.529	0.953	3.346	0.655	1.064	0.820	0.875	Ribosomal protein L7Ae/L30e/S12e/Gadd45	GI-H
MELO3C017577.2	7.054	5.633	19.240	3.684	4.971	4.911	4.957	apoptotic chromatin condensation inducer in the nucleus	GI-H
MELO3C017579.2	0.568	0.544	3.775	0.654	1.745	NA	0.661	DNA-directed RNA polymerases II and V subunit 8A-like	GI-H
MELO3C017613.2	0.840	0.950	5.682	0.547	1.560	1.138	1.000	Pentatricopeptide repeat-containing family protein	GI-H
MELO3C017624.2	1.804	1.652	6.911	1.595	1.655	0.455	2.138	Superoxide dismutase	GI-H
MELO3C017654.2	1.499	2.544	3.416	1.902	1.298	4.853	0.732	Small G family protein/RhoGAP family protein	GI-H
MELO3C017663.2	0.291	0.357	2.827	NA	0.623	0.146	0.484	Structural maintenance of chromosomes family protein	GI-H

Gene ID	FPKM							Gene Description	Specific in episode
	FS	GI-M	GM-M	AN-M	GI-H	GM-H	AN-H		
MELO3C017665.2	1.233	1.415	4.122	1.862	1.347	0.577	1.693	Ubiquitin-protein ligase, putative	GI-H
MELO3C017674.2	30.576	43.389	168.763	12.251	47.933	1.690	46.866	Lipid transfer protein	GI-H
MELO3C017675.2	0.339	0.577	1.997	0.212	0.524	0.465	0.287	prolyl 4-hydroxylase 1	GI-H
MELO3C017678.2	0.721	0.661	2.475	0.441	0.967	0.569	0.628	ATP-dependent DNA helicase 2 subunit KU70	GI-H
MELO3C017682.2	0.868	3.778	3.066	7.441	1.046	13.919	1.209	extensin-2	GI-H
MELO3C017686.2	0.793	1.126	2.749	0.778	1.051	0.989	0.540	Pentatricopeptide repeat (PPR) superfamily protein	GI-H
MELO3C017700.2	0.210	0.310	2.631	0.482	0.330	1.450	0.206	disease resistance protein RGA2-like	GI-H
MELO3C017704.2	0.370	0.167	2.467	0.264	0.591	0.462	0.079	Protein ROOT PRIMORDIUM DEFECTIVE 1	GI-H
MELO3C017713.2	0.225	0.171	4.873	NA	0.334	NA	0.235	At3g59430	GI-H
MELO3C017715.2	0.679	1.270	2.671	1.031	0.643	1.277	0.517	UPF0454 protein C12orf49	GI-H
MELO3C017730.2	0.508	0.669	2.150	0.321	0.309	0.414	0.403	Pentatricopeptide repeat-containing family protein	GI-H
MELO3C017744.2	0.952	0.729	8.075	0.871	0.980	0.224	0.569	protein MAK16 homolog	GI-H
MELO3C017754.2	5.529	17.420	24.387	7.008	4.156	6.893	4.426	NAC domain-containing protein	GI-H
MELO3C017769.2	0.568	0.854	2.787	0.291	0.985	NA	0.591	Elongation factor 4	GI-H
MELO3C017775.2	1.801	2.941	5.679	1.199	2.395	0.739	2.388	DNA-directed RNA polymerases II and V subunit 8A-like	GI-H
MELO3C017781.2	1.209	1.977	3.224	1.845	1.576	2.605	1.556	NADH:ubiquinone oxidoreductase intermediate-associated protein 30	GI-H
MELO3C017782.2	0.349	0.351	1.453	0.144	0.283	0.077	0.416	snRNA-activating protein complex subunit, putative	GI-H
MELO3C017784.2	0.840	0.699	3.238	NA	0.864	0.597	0.466	Pentatricopeptide repeat-containing family protein	GI-H
MELO3C017796.2	0.236	0.386	8.820	0.230	1.008	NA	0.937	ATP-dependent RNA helicase	GI-H
MELO3C017798.2	1.858	2.036	7.172	2.157	2.375	4.682	2.942	Hydroxyacylglutathione hydrolase	GI-H
MELO3C017799.2	1.527	1.943	4.917	1.650	1.933	5.206	1.436	F2P16.20 protein, putative isoform 1	GI-H
MELO3C017804.2	0.764	0.583	2.642	0.408	0.445	0.497	0.395	LIM domain-containing protein A, putative isoform 1	GI-H
MELO3C017814.2	0.946	1.404	4.977	0.928	1.229	1.003	1.139	Ubiquitin thioesterase	GI-H
MELO3C017819.2	4.866	3.206	10.382	2.608	4.760	2.320	3.604	random slug protein 5	GI-H
MELO3C017821.2	0.818	0.801	2.753	0.782	0.984	0.733	0.991	DCD (Development and Cell Death) domain protein	GI-H
MELO3C017835.2	0.767	0.745	2.868	0.737	0.949	0.313	1.080	Mitochondrial glycoprotein family protein	GI-H
MELO3C017836.2	0.582	0.175	3.396	0.281	0.325	NA	0.516	UPF0326 protein	GI-H
MELO3C017841.2	1.675	2.374	12.079	2.038	2.239	3.261	2.390	Signal recognition particle 54 kDa protein	GI-H
MELO3C017849.2	0.413	0.769	3.179	0.803	0.617	0.698	0.543	At1g15760	GI-H
MELO3C017880.2	0.360	0.625	4.137	0.596	0.569	0.496	0.401	Glucan endo-1,3-beta-glucosidase	GI-H

Gene ID	FPKM							Gene Description	Specific in episode
	FS	GI-M	GM-M	AN-M	GI-H	GM-H	AN-H		
MELO3C017889.2	1.283	3.242	6.088	2.868	1.759	2.331	1.599	Embryo-specific protein 3	GI-H
MELO3C017894.2	0.466	0.659	2.400	0.351	0.699	0.253	0.586	Pentatricopeptide repeat-containing family protein	GI-H
MELO3C017902.2	1.268	1.446	6.416	1.651	1.480	1.272	0.961	monothiol glutaredoxin-S15, mitochondrial	GI-H
MELO3C017913.2	1.210	1.899	4.642	1.252	1.141	1.138	1.141	Histone-lysine N-methyltransferase	GI-H
MELO3C017936.2	4.775	4.003	20.905	2.322	9.511	1.104	8.033	kinesin-4	GI-H
MELO3C017939.2	32.439	37.779	168.980	37.972	37.169	14.020	31.960	Ribosomal protein L19	GI-H
MELO3C017941.2	2.584	2.466	5.953	2.245	1.935	2.722	2.418	Rela-spot homolog family protein	GI-H
MELO3C017960.2	0.399	1.976	4.239	1.693	1.391	1.762	0.805	NADH dehydrogenase [ubiquinone] iron-sulfur protein 5-B	GI-H
MELO3C017975.2	0.688	1.421	2.323	0.122	0.637	NA	1.072	Regulator of chromosome condensation (RCC1) family with FYVE zinc finger domain-containing protein	GI-H
MELO3C017986.2	0.259	0.133	3.899	NA	0.390	NA	0.798	Serine-threonine protein kinase plant-type	GI-H
MELO3C017988.2	1.813	2.419	9.823	1.490	1.614	1.982	1.493	Leucine-rich repeat family protein	GI-H
MELO3C017993.2	13.729	17.584	77.120	20.252	15.510	7.509	10.854	40S ribosomal protein S29	GI-H
MELO3C018004.2	7.193	7.126	30.214	8.263	7.650	0.424	4.382	Trypsin inhibitor 5	GI-H
MELO3C018007.2	3.279	4.877	26.396	6.882	4.067	NA	3.277	Glycyl-tRNA synthetase beta subunit	GI-H
MELO3C018008.2	4.651	8.393	47.647	8.723	8.995	3.555	6.376	Glycyl-tRNA synthetase beta subunit	GI-H
MELO3C018010.2	0.884	1.081	4.461	0.532	0.770	0.479	0.669	At3g19650	GI-H
MELO3C018014.2	1.709	2.698	4.111	1.998	1.140	3.932	1.846	telomere repeat-binding factor 1 isoform X2	GI-H
MELO3C018022.2	0.794	0.654	1.785	0.894	0.546	0.544	0.530	Pre-mRNA-processing protein 40A	GI-H
MELO3C018028.2	2.315	1.033	10.595	0.300	4.848	NA	4.213	histone acetyltransferase KAT6B-like	GI-H
MELO3C018032.2	0.728	0.578	6.447	0.764	1.006	0.973	0.703	Pinin-like protein	GI-H
MELO3C018043.2	22.530	27.023	49.377	16.325	24.610	24.366	19.283	Transcription factor GTE7	GI-H
MELO3C032639.2	0.724	0.797	2.576	0.404	0.724	0.625	0.417	Unknown protein	GI-H
MELO3C018056.2	1.297	1.285	7.779	2.936	1.855	0.190	1.480	Carbonic anhydrase	GI-H
MELO3C007004.2	0.882	0.872	4.235	0.916	1.063	0.741	1.156	protein FAR1-RELATED SEQUENCE 4 isoform X1	GI-H
MELO3C007007.2	0.951	0.971	4.005	1.043	1.132	0.818	1.636	protein prune homolog isoform X2	GI-H
MELO3C007029.2	1.142	2.016	3.399	0.486	0.780	1.702	0.687	glycine-rich RNA-binding protein 4, mitochondrial	GI-H
MELO3C007034.2	1.395	0.824	4.022	0.863	1.625	0.412	1.956	GTP-binding protein ERG	GI-H
MELO3C007035.2	0.420	0.566	3.144	NA	0.784	0.331	0.645	haloacid dehalogenase-like hydrolase domain-containing protein At3g48420	GI-H
MELO3C007042.2	7.944	14.871	28.112	21.604	12.876	10.318	12.012	coatomer subunit epsilon-1-like	GI-H
MELO3C007044.2	2.107	1.504	12.598	0.375	2.600	NA	2.938	Protein CHUP1, chloroplastic	GI-H

Gene ID	FPKM							Gene Description	Specific in episode
	FS	GI-M	GM-M	AN-M	GI-H	GM-H	AN-H		
MELO3C007047.2	0.178	0.337	3.492	0.312	0.321	0.657	0.379	Protein kinase	GI-H
MELO3C007049.2	1.136	1.473	2.595	1.459	1.066	1.584	0.799	Alpha N-terminal protein methyltransferase 1	GI-H
MELO3C007056.2	0.420	0.504	1.471	0.738	0.349	0.381	0.253	reticuline oxidase-like protein	GI-H
MELO3C007066.2	0.540	0.750	4.187	0.320	1.041	0.314	0.919	origin of replication complex subunit 1B-like	GI-H
MELO3C007071.2	1.161	0.905	4.442	0.358	0.805	1.367	0.751	WPP domain associated protein	GI-H
MELO3C007075.2	0.484	0.547	9.146	0.758	0.884	1.151	0.254	ADP-ribosylation factor GTPase-activating protein AGD12	GI-H
MELO3C007077.2	0.327	0.329	1.672	0.269	0.400	0.383	0.212	Nuclear transcription factor Y subunit A-7	GI-H
MELO3C007081.2	1.283	2.086	7.843	1.692	2.553	1.633	1.918	SIT4 phosphatase-associated family protein	GI-H
MELO3C007092.2	1.747	2.830	8.007	1.793	3.601	0.970	2.009	geranylgeranyl transferase type-2 subunit alpha	GI-H
MELO3C007093.2	1.253	1.073	4.947	1.189	1.712	0.121	1.973	protein BREAST CANCER SUSCEPTIBILITY 1 homolog	GI-H
MELO3C007106.2	2.684	3.402	14.588	0.921	4.001	NA	4.191	Remorin family protein	GI-H
MELO3C007107.2	0.264	0.885	2.599	NA	0.419	NA	0.749	Pentatricopeptide repeat-containing protein	GI-H
MELO3C007110.2	0.875	1.324	4.309	1.047	0.905	1.307	0.880	Non-specific serine/threonine protein kinase	GI-H
MELO3C007120.2	0.394	0.694	7.044	0.638	0.922	0.074	1.015	50S ribosomal protein L14	GI-H
MELO3C007141.2	58.476	92.988	167.233	83.224	80.065	18.824	68.068	60S ribosomal protein L18a	GI-H
MELO3C007151.2	6.561	14.682	25.487	10.176	10.398	34.413	7.363	transcription factor bHLH144	GI-H
MELO3C007159.2	3.398	4.382	19.041	4.743	2.791	4.226	3.918	Programmed cell death protein 5	GI-H
MELO3C007161.2	2.251	4.765	12.172	4.722	4.813	2.779	3.266	coatomer subunit zeta-1-like	GI-H
MELO3C007164.2	0.860	1.771	10.732	1.493	1.175	1.437	0.938	CTD small phosphatase-like protein 2	GI-H
MELO3C007165.2	3.317	3.037	6.797	1.571	2.673	2.889	3.014	protein MEI2-like 5	GI-H
MELO3C007172.2	1.227	1.089	10.629	2.146	1.582	1.461	1.408	Serine/threonine-protein kinase PLK4	GI-H
MELO3C007177.2	2.945	3.223	9.107	2.103	3.758	6.055	3.007	Transcription factor GTE12	GI-H
MELO3C007181.2	0.847	3.070	13.494	2.259	2.800	1.350	2.101	MADS box transcription factor AGAMOUS	GI-H
MELO3C007190.2	3.774	4.560	10.391	2.436	3.068	4.108	4.062	Splicing factor 3B, subunit 5	GI-H
MELO3C007192.2	1.133	3.931	5.892	1.816	2.696	2.011	1.541	heat stress transcription factor A-4c-like	GI-H
MELO3C007194.2	4.808	2.831	10.354	2.434	4.281	0.412	4.186	DNA polymerase III gamma-tau subunit	GI-H
MELO3C007205.2	0.268	0.801	1.268	0.729	0.329	0.916	0.430	RING/FYVE/PHD zinc finger protein	GI-H
MELO3C007235.2	0.406	0.613	3.029	0.637	0.724	0.800	0.538	Protein IQ-DOMAIN 1	GI-H
MELO3C007237.2	2.219	1.524	7.168	2.407	2.747	0.774	3.408	Apoptosis inhibitor 5-like protein API5	GI-H
MELO3C007246.2	6.186	9.650	36.966	10.753	7.404	12.824	6.652	28 kDa heat/acid-stable phosphoprotein-like protein	GI-H
MELO3C007250.2	0.463	0.480	1.630	0.301	0.587	0.390	0.267	Optic atrophy 3 protein (OPA3)	GI-H

Gene ID	FPKM							Gene Description	Specific in episode
	FS	GI-M	GM-M	AN-M	GI-H	GM-H	AN-H		
MELO3C007261.2	1.507	0.762	5.984	1.516	0.583	1.231	0.553	dnaJ protein homolog 1-like	GI-H
MELO3C007263.2	1.554	1.229	12.124	3.855	2.011	2.036	1.091	Protein E6	GI-H
MELO3C007268.2	0.597	0.676	2.323	0.468	0.638	0.248	0.630	protein FRIGIDA-ESSENTIAL 1 isoform X1	GI-H
MELO3C007288.2	0.390	0.291	3.679	0.308	0.410	NA	0.433	SMR domain-containing protein	GI-H
MELO3C007290.2	0.427	0.878	3.072	0.824	1.337	0.301	0.904	RNA polymerase II transcription factor B subunit 2	GI-H
MELO3C007295.2	0.369	0.322	2.086	NA	0.231	0.331	0.152	Tetratricopeptide repeat-like superfamily protein, putative isoform 1	GI-H
MELO3C007305.2	2.600	4.554	8.994	1.941	3.807	6.768	3.176	SNF2 domain-containing protein / helicase domain-containing protein / zinc finger protein-like protein	GI-H
MELO3C007313.2	2.035	2.272	7.474	1.699	2.505	1.372	2.029	pre-mRNA-splicing factor SYF1	GI-H
MELO3C007332.2	1.723	2.552	6.532	1.366	1.918	1.046	2.028	lysine-specific demethylase JMJ25	GI-H
MELO3C007341.2	0.360	0.744	1.472	0.503	0.323	0.769	0.699	transcription factor bHLH112-like isoform X1	GI-H
MELO3C007342.2	4.260	2.736	16.766	4.193	5.771	1.069	5.281	Pentatricopeptide repeat-containing family protein	GI-H
MELO3C007346.2	0.329	0.295	2.929	0.260	0.484	NA	0.431	Pentatricopeptide repeat-containing family protein	GI-H
MELO3C007347.2	1.947	3.014	6.789	2.383	2.527	4.217	2.480	Vacuolar protein sorting-associated protein 27	GI-H
MELO3C007349.2	0.248	0.316	1.956	0.175	0.449	0.137	0.254	Zinc finger, C2H2	GI-H
MELO3C007367.2	1.147	1.464	4.832	0.631	1.023	1.803	0.681	Receptor-like kinase	GI-H
MELO3C007380.2	1.271	0.770	5.991	0.513	1.089	NA	0.739	Adenosine deaminase-like protein	GI-H
MELO3C007388.2	1.402	2.135	8.304	1.426	2.635	1.376	2.438	calcium-dependent protein kinase-like	GI-H
MELO3C007392.2	0.556	0.874	4.952	1.159	2.070	0.409	1.392	Hexosyltransferase	GI-H
MELO3C007399.2	0.585	1.752	4.411	0.370	2.034	0.324	1.587	Rhamnogalacturonate lyase	GI-H
MELO3C007408.2	4.683	14.348	11.312	69.862	3.542	27.461	3.887	Aspartic proteinase	GI-H
MELO3C007425.2	1.199	0.638	14.200	3.852	0.585	22.647	0.711	1-aminocyclopropane-1-carboxylate oxidase 1	GI-H
MELO3C007431.2	3.004	4.654	7.000	3.474	2.450	5.321	1.623	alkaline ceramidase 3	GI-H
MELO3C007432.2	1.333	2.196	3.841	1.895	1.399	6.329	1.398	Phosphatidate cytidylyltransferase	GI-H
MELO3C007464.2	0.377	0.522	1.797	NA	0.678	0.328	0.350	tRNA(His) guanylyltransferase 2-like	GI-H
MELO3C007465.2	0.505	0.647	2.738	0.442	1.220	0.241	0.810	Protein SLOW GREEN 1, chloroplastic	GI-H
MELO3C007468.2	3.486	6.333	27.670	10.737	4.104	6.594	3.602	Male gametophyte defective 1	GI-H
MELO3C007469.2	0.528	1.321	3.025	1.422	1.282	3.004	0.471	iron-sulfur assembly protein IscA, chloroplastic	GI-H
MELO3C007480.2	0.646	0.800	5.837	2.133	1.960	5.415	1.107	Cytochrome P450 family protein	GI-H
MELO3C007482.2	1.147	2.010	4.998	1.617	2.268	109.149	1.090	Cytochrome P450 family protein	GI-H
MELO3C007497.2	0.656	0.969	3.384	0.333	1.140	0.101	0.670	Transcription termination factor family protein	GI-H

Gene ID	FPKM							Gene Description	Specific in episode
	FS	GI-M	GM-M	AN-M	GI-H	GM-H	AN-H		
MELO3C007499.2	0.751	1.765	12.084	1.618	2.257	0.406	1.116	protein DA1-related 1-like	GI-H
MELO3C007516.2	1.243	1.730	4.968	1.139	1.302	1.085	1.488	tRNA modification GTPase MnmE	GI-H
MELO3C007526.2	0.697	0.788	2.476	0.597	0.611	0.186	0.736	Cyclin-H1-1	GI-H
MELO3C007534.2	0.488	1.336	6.169	0.690	1.483	1.211	0.744	autophagy-related protein 101	GI-H
MELO3C007549.2	0.206	0.722	10.820	4.025	1.042	1.331	1.221	Estradiol 17-beta-dehydrogenase 1	GI-H
MELO3C007550.2	1.979	1.948	5.121	1.690	1.782	3.685	2.138	Transmembrane protein 131-like	GI-H
MELO3C007572.2	3.017	2.765	13.704	4.709	5.661	12.140	6.549	AP2-like ethylene-responsive transcription factor TOE3	GI-H
MELO3C007580.2	0.785	1.039	5.269	0.922	1.085	0.473	0.573	phosphatidylinositol 3,4,5-trisphosphate 3-phosphatase and protein-tyrosine-phosphatase PTEN2A-like	GI-H
MELO3C007587.2	0.602	0.871	2.177	0.717	0.700	0.753	0.832	Mortality factor 4-like protein 1	GI-H
MELO3C007593.2	1.445	1.630	6.004	0.989	1.392	1.973	1.361	F-box-like/WD repeat-containing protein TBL1XR1	GI-H
MELO3C007598.2	0.734	0.812	2.205	0.510	0.666	1.072	0.457	RING-type E3 ubiquitin transferase	GI-H
MELO3C007603.2	1.660	2.037	4.417	1.359	1.294	2.109	1.211	Suppressor of RPS4-RLD 1	GI-H
MELO3C007617.2	0.380	0.555	11.061	NA	0.499	0.312	0.151	At5g67390	GI-H
MELO3C007629.2	0.959	0.457	3.414	0.170	0.876	NA	1.384	Condensin-2 complex subunit G2, putative	GI-H
MELO3C007631.2	1.543	2.083	6.195	0.896	1.910	1.352	1.691	Peptidyl-prolyl cis-trans isomerase-like protein	GI-H
MELO3C007640.2	0.918	1.132	2.699	0.783	1.161	0.549	0.757	telomere repeat-binding factor 2 isoform X1	GI-H
MELO3C007644.2	1.469	1.761	6.703	0.337	1.832	0.777	1.482	At5g67620	GI-H
MELO3C007661.2	1.498	2.977	8.099	3.868	2.869	1.749	2.059	Transmembrane protein, putative	GI-H
MELO3C007665.2	3.451	3.599	7.699	1.769	2.837	3.090	3.182	Nuclear matrix constituent protein 1	GI-H
MELO3C007683.2	2.218	1.432	4.618	1.628	1.928	3.122	2.070	Kinesin-like protein	GI-H
MELO3C007686.2	1.827	2.883	8.847	3.070	2.015	4.531	2.972	SPT2 chromatin protein	GI-H
MELO3C007688.2	0.302	0.328	1.655	0.323	0.209	0.184	0.290	tRNA-dihydrouridine synthase	GI-H
MELO3C007694.2	0.830	0.473	21.810	0.397	2.724	NA	2.225	transcription factor bHLH93 isoform X2	GI-H
MELO3C007713.2	0.745	1.225	5.854	1.412	1.673	3.845	0.808	DNA ligase	GI-H
MELO3C007714.2	0.394	0.288	5.065	0.282	0.394	0.138	0.683	Basic helix-loop-helix (BHLH) DNA-binding superfamily protein	GI-H
MELO3C007755.2	0.488	1.865	2.275	0.951	0.647	2.840	0.114	Ras-related protein	GI-H
MELO3C007766.2	2.869	3.161	7.448	1.707	2.697	1.504	2.488	Protein sco1	GI-H
MELO3C007772.2	0.752	1.230	4.105	1.167	1.411	0.917	1.658	2,3-bisphosphoglycerate-dependent phosphoglycerate mutase	GI-H
MELO3C007782.2	1.890	1.944	6.973	2.810	2.221	2.109	1.780	Formation of crista junctions protein 1	GI-H
MELO3C007784.2	1.748	3.122	10.080	NA	1.267	1.181	2.728	Unknown protein	GI-H

Gene ID	FPKM							Gene Description	Specific in episode
	FS	GI-M	GM-M	AN-M	GI-H	GM-H	AN-H		
MELO3C007791.2	1.053	1.508	4.827	0.758	1.514	0.665	0.975	Pentatricopeptide repeat-containing family protein	GI-H
MELO3C007833.2	1.695	1.370	5.572	0.833	1.770	0.548	1.214	ATPase family AAA domain-containing protein 3	GI-H
MELO3C007840.2	1.200	3.223	6.042	NA	2.405	0.425	1.237	At2g18210	GI-H
MELO3C007841.2	1.948	3.384	6.836	1.850	1.681	4.167	1.438	Bacteriophage N4 adsorption B	GI-H
MELO3C007889.2	1.907	4.111	17.564	NA	3.288	3.945	3.301	histone H1	GI-H
MELO3C007904.2	4.655	7.335	18.040	2.480	5.333	1.882	2.325	calcium-dependent protein kinase 28-like	GI-H
MELO3C007921.2	0.474	0.984	3.012	0.645	0.741	2.020	0.902	At4g35980	GI-H
MELO3C007922.2	3.452	3.280	27.296	1.263	9.813	NA	8.071	Protein POLLENLESS 3-LIKE 2	GI-H
MELO3C007925.2	1.905	1.880	5.258	0.990	2.071	2.579	1.356	cell cycle checkpoint protein RAD17	GI-H
MELO3C007978.2	0.312	0.682	1.783	0.496	0.337	0.552	0.424	serine/threonine-protein kinase/endoribonuclease IRE1a-like isoform X3	GI-H
MELO3C007988.2	1.109	1.384	7.870	0.471	2.214	0.354	2.422	Myb family transcription factor family protein	GI-H
MELO3C007991.2	2.869	4.532	6.366	2.157	2.419	4.207	1.924	Hexosyltransferase	GI-H
MELO3C007996.2	2.704	5.443	25.234	3.541	6.756	0.977	5.848	Methyltransferase small domain protein, putative (DUF2431)	GI-H
MELO3C007997.2	1.589	2.132	8.670	1.396	2.087	0.290	2.234	DNA-directed RNA polymerase II subunit 4	GI-H
MELO3C007998.2	0.774	1.199	5.934	1.340	1.103	1.352	0.892	Calcineurin B-like protein	GI-H
MELO3C007999.2	0.675	1.103	7.893	0.468	1.786	1.079	2.559	BTB/POZ domain-containing protein At5g47800	GI-H
MELO3C008005.2	1.091	2.648	8.547	3.085	3.625	4.773	1.118	Sec14p-like phosphatidylinositol transfer family protein	GI-H
MELO3C008015.2	0.547	0.387	1.611	NA	0.757	0.260	0.667	pentatricopeptide repeat-containing protein At4g13650	GI-H
MELO3C008024.2	6.097	10.337	18.419	10.322	8.971	30.471	9.060	Transcription elongation factor (TFIIS) family protein	GI-H
MELO3C008025.2	0.196	0.971	7.797	0.533	0.660	1.948	1.008	WAT1-related protein	GI-H
MELO3C008038.2	0.239	0.248	2.641	NA	0.490	0.060	0.293	Protein TONSOKU	GI-H
MELO3C008046.2	1.430	1.522	5.193	1.034	1.251	0.966	1.342	zinc finger CCCH domain-containing protein 38 isoform X1	GI-H
MELO3C008053.2	0.338	0.901	4.054	0.463	0.533	0.530	1.337	O-fucosyltransferase family protein	GI-H
MELO3C008059.2	0.318	0.434	1.768	0.214	0.473	0.218	0.595	NADH dehydrogenase-like complex O	GI-H
MELO3C008064.2	1.138	1.535	3.094	0.938	1.347	2.313	0.950	Histone deacetylase, putative	GI-H
MELO3C024603.2	12.513	14.285	32.706	20.777	15.618	22.328	15.977	DUF248-1	GI-H
MELO3C024599.2	0.637	0.421	5.350	0.315	1.104	0.177	1.271	protein CHROMATIN REMODELING 24	GI-H
MELO3C024592.2	0.603	0.542	3.321	1.017	0.940	0.241	0.322	alpha-ketoglutarate-dependent dioxygenase alkB homolog 6	GI-H
MELO3C024585.2	0.712	0.902	1.853	0.801	0.425	0.515	0.418	exocyst complex component EXO70A1	GI-H
MELO3C024578.2	0.092	0.128	1.965	0.008	0.157	0.101	0.177	transcription factor E2FA-like isoform X2	GI-H

Gene ID	FPKM							Gene Description	Specific in episode
	FS	GI-M	GM-M	AN-M	GI-H	GM-H	AN-H		
MELO3C024558.2	0.990	1.588	9.751	1.634	1.994	1.189	1.245	sorting nexin 1	GI-H
MELO3C024552.2	3.148	2.913	47.983	3.625	7.622	1.469	4.595	Small nuclear ribonucleoprotein G, putative	GI-H
MELO3C024537.2	0.294	0.441	1.488	0.165	0.157	0.280	0.240	transcription initiation factor TFIID subunit 12	GI-H
MELO3C024525.2	5.333	4.116	10.673	2.536	4.389	1.623	4.370	serine/threonine-protein kinase D6PK	GI-H
MELO3C024524.2	1.949	2.237	6.255	1.689	1.827	2.452	2.547	protein BUD31 homolog 2	GI-H
MELO3C024516.2	1.312	1.203	4.657	0.930	1.356	0.632	1.249	protein ROS1	GI-H
MELO3C019311.2	1.127	4.510	12.550	7.591	5.060	NA	5.851	glucomannan 4-beta-mannosyltransferase 9-like	GI-H
MELO3C032937.2	0.742	1.096	5.875	2.471	2.491	NA	2.425	glucomannan 4-beta-mannosyltransferase 9-like	GI-H
MELO3C024499.2	0.261	0.774	1.632	1.641	0.493	NA	0.669	Threonine dehydratase	GI-H
MELO3C024490.2	10.212	10.744	66.742	7.480	20.077	0.992	21.362	Histone H2B	GI-H
MELO3C024481.2	1.186	2.085	3.251	1.767	1.425	2.415	0.985	Dual specificity phosphatase Cdc25, putative	GI-H
MELO3C024474.2	4.762	6.585	25.659	6.758	7.712	2.895	5.653	zinc finger protein 593	GI-H
MELO3C024470.2	0.469	0.380	1.780	0.371	0.841	0.106	0.645	E3 ubiquitin-protein ligase RFWD3	GI-H
MELO3C024466.2	2.087	2.699	14.607	1.417	2.240	NA	2.221	heavy metal-associated isoprenylated plant protein 3-like isoform X2	GI-H
MELO3C024460.2	0.683	1.510	3.473	0.922	1.622	1.906	0.737	RNA polymerase sigma factor	GI-H
MELO3C020742.2	0.876	0.414	3.657	0.198	0.987	0.054	0.966	kinesin-4 isoform X2	GI-H
MELO3C019097.2	0.998	1.977	11.625	1.313	1.414	1.951	1.381	Arginine/serine-rich coiled-coil 2	GI-H
MELO3C032976.2	0.422	0.304	1.162	0.582	0.257	0.964	0.415	Unknown protein	GI-H
MELO3C019112.2	7.351	8.062	16.052	7.160	6.895	1.825	6.468	10 kDa chaperonin-like	GI-H
MELO3C019115.2	3.294	3.679	35.412	1.261	10.162	NA	7.912	Mitotic cyclin	GI-H
MELO3C032982.2	0.102	NA	1.754	NA	0.090	0.095	0.156	dual specificity protein kinase shkC-like isoform X1	GI-H
MELO3C019126.2	1.137	5.925	8.331	2.689	1.575	8.975	2.504	cysteine proteinase inhibitor A	GI-H
MELO3C019181.2	0.199	0.419	1.406	0.300	0.195	0.333	0.129	Pentatricopeptide repeat-containing protein	GI-H
MELO3C019188.2	0.195	NA	2.672	0.923	0.664	1.534	0.381	Glycosyltransferase, putative	GI-H
MELO3C023156.2	0.168	0.130	2.073	0.084	0.149	NA	0.158	Unknown protein	GI-H
MELO3C023146.2	1.162	1.882	4.795	1.524	1.592	2.778	1.612	villin-2-like	GI-H
MELO3C023120.2	1.389	1.915	3.210	0.920	1.211	2.117	1.145	zinc finger AN1 and C2H2 domain-containing stress-associated protein 11-like	GI-H
MELO3C009082.2	0.842	1.014	2.470	1.025	1.147	0.810	0.929	protein cornichon homolog 1 isoform X1	GI-H
MELO3C009063.2	0.462	0.727	1.993	0.445	0.408	0.517	0.548	serine/threonine-protein kinase Nek6 isoform X1	GI-H
MELO3C009057.2	0.775	0.980	2.153	1.039	0.969	1.452	0.984	ATP-binding cassette sub-family G member 1	GI-H

Gene ID	FPKM							Gene Description	Specific in episode
	FS	GI-M	GM-M	AN-M	GI-H	GM-H	AN-H		
MELO3C009036.2	1.364	3.715	13.831	1.983	3.672	1.182	3.138	40S ribosomal protein S25, putative	GI-H
MELO3C009032.2	1.519	1.723	11.619	1.482	2.424	0.357	1.456	fructokinase-like 2, chloroplastic	GI-H
MELO3C009019.2	0.522	1.248	6.520	0.916	1.491	0.628	0.911	Rhodanese-like domain-containing family protein	GI-H
MELO3C009001.2	0.255	0.335	1.402	NA	0.274	0.055	0.349	Pentatricopeptide repeat-containing protein family	GI-H
MELO3C008988.2	0.759	8.890	16.910	0.208	5.033	0.369	0.118	Fatty acyl-CoA reductase	GI-H
MELO3C008981.2	0.378	0.467	4.038	0.605	0.389	0.400	0.634	Ribosomal protein L37, mitochondrial	GI-H
MELO3C008977.2	1.416	1.350	3.524	0.886	1.662	0.727	1.745	Kinesin-like protein	GI-H
MELO3C008970.2	0.427	0.305	1.682	0.406	0.319	0.419	0.341	vacuolar protein sorting-associated protein 20 homolog 2-like	GI-H
MELO3C008959.2	0.532	0.625	2.217	0.720	0.811	1.107	0.453	1-acyl-sn-glycerol-3-phosphate acyltransferase	GI-H
MELO3C033079.2	0.462	0.610	1.928	0.288	0.698	0.176	0.774	Pentatricopeptide repeat-containing protein	GI-H
MELO3C033084.2	0.853	0.823	1.893	0.618	0.696	0.782	0.933	Unknown protein	GI-H
MELO3C008937.2	0.349	0.455	1.276	0.265	0.370	0.586	0.318	RNA polymerase II transcriptional coactivator	GI-H
MELO3C033370.2	0.408	0.327	2.226	0.128	0.462	NA	0.430	Protein DETOXIFICATION	GI-H
MELO3C008909.2	0.851	1.787	8.332	0.760	1.917	0.908	1.442	transcription termination factor MTEF18, mitochondrial-like	GI-H
MELO3C033382.2	0.321	0.314	1.360	0.315	0.375	0.109	0.303	Peroxisomal multifunctional enzyme type 2	GI-H
MELO3C008903.2	0.482	0.762	2.374	0.785	0.524	0.947	0.596	CDK5RAP3-like protein	GI-H
MELO3C008899.2	2.210	2.070	5.526	1.705	2.725	1.265	2.481	Kinase family protein	GI-H
MELO3C008887.2	0.677	1.203	10.490	0.857	1.192	0.631	1.198	Serine/threonine-protein kinase	GI-H
MELO3C008876.2	0.814	0.957	2.028	0.880	0.989	0.736	1.001	mediator of RNA polymerase II transcription subunit 11	GI-H
MELO3C008875.2	3.483	4.017	11.126	5.828	3.992	13.152	4.382	Ulp1 protease family C-terminal catalytic domain containing protein expressed	GI-H
MELO3C008864.2	1.586	1.510	8.222	1.339	3.001	0.694	2.444	replication factor C subunit 3	GI-H
MELO3C008863.2	0.526	0.275	2.314	0.212	0.429	0.518	0.169	Microfibrillar-associated protein-like protein	GI-H
MELO3C008835.2	0.369	0.315	2.434	0.315	0.881	0.094	0.626	ATP-dependent DNA helicase RecQ-like	GI-H
MELO3C008833.2	0.458	0.580	2.730	0.246	1.200	0.100	1.166	ATP-dependent DNA helicase RecQ-like	GI-H
MELO3C008825.2	0.501	0.504	1.694	0.231	0.844	0.121	0.794	ATP-dependent DNA helicase RecQ-like	GI-H
MELO3C008822.2	2.120	3.217	8.565	2.009	3.038	3.118	3.329	protein RRP6-like 2	GI-H
MELO3C008815.2	2.040	1.679	6.111	1.019	2.292	0.667	1.405	nucleolar MIF4G domain-containing protein 1	GI-H
MELO3C008806.2	2.544	4.014	22.311	4.057	2.168	4.045	1.694	Echinoderm microtubule-associated protein-like 6	GI-H
MELO3C008802.2	2.118	6.067	8.657	6.231	3.498	3.139	3.918	Glutamate dehydrogenase	GI-H
MELO3C008794.2	0.976	1.009	4.985	NA	1.254	NA	2.026	syntaxin-related protein KNOLLE	GI-H

Gene ID	FPKM							Gene Description	Specific in episode
	FS	GI-M	GM-M	AN-M	GI-H	GM-H	AN-H		
MELO3C025283.2	1.168	1.179	6.030	1.332	1.280	1.459	1.432	Hexosyltransferase	GI-H
MELO3C025223.2	2.417	3.589	9.677	2.738	4.109	3.450	3.200	protein FATTY ACID EXPORT 3, chloroplastic	GI-H
MELO3C026055.2	0.549	0.799	5.978	NA	0.800	0.765	0.665	Non-specific serine/threonine protein kinase	GI-H
MELO3C026053.2	0.830	1.189	9.186	0.818	1.745	1.477	0.727	WEB family protein At2g38370	GI-H
MELO3C026046.2	0.259	0.469	1.889	0.792	0.369	NA	0.911	alpha-ketoglutarate-dependent dioxygenase AlkB-like	GI-H
MELO3C026013.2	0.171	0.082	1.678	NA	0.223	NA	0.400	Plectin-like protein	GI-H
MELO3C026009.2	0.610	1.148	4.394	1.834	1.146	1.522	1.348	BAG family molecular chaperone regulator 4	GI-H
MELO3C026805.2	0.404	0.582	2.183	0.330	0.360	0.396	0.298	Coiled-coil protein (DUF572)	GI-H
MELO3C026804.2	3.943	5.772	13.542	2.373	6.226	3.249	5.345	histone-lysine N-methyltransferase, H3 lysine-9 specific SUVH1-like	GI-H
MELO3C026324.2	1.299	1.320	4.053	0.926	1.389	0.688	1.204	DnaJ	GI-H
MELO3C033461.2	0.311	0.352	1.637	0.890	0.439	0.463	0.450	Ribosomal protein	GI-H
MELO3C026338.2	0.177	0.213	2.623	0.216	0.549	0.175	0.216	Threonine-tRNA ligase 2	GI-H
MELO3C026340.2	0.430	0.371	2.701	0.182	0.579	NA	0.346	SAP-like protein BP-73	GI-H
MELO3C014208.2	0.261	0.163	1.532	0.037	0.286	0.157	0.277	PAX-interacting protein 1	GI-H
MELO3C014217.2	0.518	0.721	3.222	0.280	0.464	0.594	0.701	Telomere repeat-binding protein 4	GI-H
MELO3C003069.2	0.985	1.395	2.761	0.898	1.169	1.617	0.891	Pentatricopeptide repeat-containing family protein	GI-H
MELO3C003080.2	0.148	0.149	1.533	0.191	0.258	0.056	0.165	Dirigent protein 17	GI-H
MELO3C003096.2	1.650	1.528	4.540	NA	0.883	1.977	0.765	Phosphatidylinositol N-acetyglucosaminlytransferase subunit P-related	GI-H
MELO3C003101.2	0.757	0.734	18.605	0.163	1.687	NA	1.618	kinesin-like protein KIN-5B	GI-H
MELO3C003110.2	3.437	2.651	8.421	2.256	2.757	0.587	1.941	Actin-depolymerizing factor family protein	GI-H
MELO3C003114.2	13.028	29.367	55.740	31.439	21.738	46.760	22.880	Mitochondrial ATP synthase 6 kDa subunit	GI-H
MELO3C003132.2	7.455	58.275	76.108	49.739	19.013	72.958	18.539	Metallothionein-like protein 4A	GI-H
MELO3C003139.2	5.778	6.485	12.435	4.897	5.798	21.049	5.535	ATP-dependent zinc metalloprotease FtsH	GI-H
MELO3C003207.2	1.849	1.899	4.554	1.324	1.419	0.874	1.938	proteasome assembly chaperone 4	GI-H
MELO3C003210.2	0.477	1.112	3.900	0.531	0.707	1.541	0.258	phosphatidylinositol:ceramide inositolphosphotransferase 1	GI-H
MELO3C003222.2	7.969	7.346	20.342	5.889	9.355	8.594	9.508	mRNA-capping enzyme	GI-H
MELO3C003246.2	0.984	0.601	2.760	0.247	0.642	0.277	0.488	RNA exonuclease 4-like	GI-H
MELO3C003249.2	0.385	0.505	1.799	0.306	0.338	0.520	0.351	target of Myb protein 1 isoform X1	GI-H
MELO3C003263.2	1.423	1.537	5.189	1.819	1.754	1.707	1.114	Golgi SNAP receptor complex member 1	GI-H
MELO3C033508.2	0.748	0.747	2.140	0.572	0.655	0.703	0.869	Pentatricopeptide repeat-containing protein	GI-H

Gene ID	FPKM							Gene Description	Specific in episode
	FS	GI-M	GM-M	AN-M	GI-H	GM-H	AN-H		
MELO3C003274.2	0.678	1.149	6.731	1.316	2.092	1.165	1.294	BnaA02g25840D protein	GI-H
MELO3C003287.2	0.603	0.588	4.152	0.206	0.391	NA	0.379	DYAD protein	GI-H
MELO3C003298.2	3.110	4.249	9.962	1.318	2.365	5.214	2.035	high mobility group B protein 1	GI-H
MELO3C033520.2	1.323	1.673	7.643	2.722	1.902	3.063	2.049	Ubiquitin carboxyl-terminal hydrolase	GI-H
MELO3C022216.2	0.589	0.582	1.276	0.235	0.540	0.401	0.584	fanconi-associated nuclease 1 homolog isoform X1	GI-H
MELO3C022214.2	0.433	0.725	1.745	0.506	0.597	0.491	0.730	O-fucosyltransferase family protein	GI-H
MELO3C022211.2	0.439	1.494	1.930	1.305	0.537	1.775	0.681	Polyneuridine-aldehyde esterase, putative	GI-H
MELO3C033525.2	0.420	0.645	5.075	0.737	0.855	1.332	0.900	Pentatricopeptide repeat-containing protein	GI-H
MELO3C022204.2	0.775	1.022	4.214	0.690	0.822	0.943	0.770	SART-1 family protein DOT2 isoform X2	GI-H
MELO3C022175.2	1.963	4.289	7.306	1.659	3.525	3.403	3.244	succinate dehydrogenase assembly factor 1, mitochondrial	GI-H
MELO3C022168.2	0.263	0.342	1.506	0.194	0.254	0.396	0.379	Pentatricopeptide repeat-containing protein	GI-H
MELO3C022161.2	0.461	1.006	3.522	0.480	0.975	0.362	0.894	pentatricopeptide repeat-containing protein At3g13880	GI-H
MELO3C022154.2	0.242	0.288	3.319	0.363	0.334	0.271	0.144	TMV resistance protein N-like	GI-H
MELO3C022152.2	0.232	0.212	1.968	0.108	0.299	NA	0.365	TMV resistance protein N-like	GI-H
MELO3C022145.2	0.759	0.986	3.809	0.569	0.731	0.519	0.847	TMV resistance protein N-like	GI-H
MELO3C022144.2	0.292	0.324	3.330	0.230	0.223	0.195	0.233	TMV resistance protein N-like	GI-H
MELO3C022143.2	0.554	0.984	2.117	0.734	1.006	0.207	0.747	MRGH12	GI-H
MELO3C022137.2	0.376	0.703	2.297	0.690	0.743	0.964	0.738	Membrane protein-like	GI-H
MELO3C022134.2	2.512	2.103	7.987	1.664	2.682	1.858	3.343	ribosome biogenesis protein BOP1 homolog	GI-H
MELO3C022115.2	0.956	1.112	3.584	1.650	1.107	2.897	1.495	Clp protease regulatory subunit clpx3, mitochondrial	GI-H
MELO3C022108.2	1.522	1.404	11.594	0.546	4.962	NA	4.107	protein NETWORKED 4A	GI-H
MELO3C022103.2	0.913	1.499	2.592	0.710	1.069	1.426	0.904	WD-repeat protein, putative	GI-H
MELO3C022094.2	5.832	5.533	26.074	4.325	9.797	0.829	7.778	T-complex protein 1 subunit zeta 1	GI-H
MELO3C022077.2	0.337	0.497	2.483	0.564	0.062	0.333	0.824	ATP-dependent zinc metalloprotease FtsH	GI-H
MELO3C022072.2	0.165	0.148	1.734	0.111	0.186	NA	0.227	Trichome birefringence-like 19	GI-H
MELO3C022066.2	0.659	1.053	3.068	0.373	0.905	0.501	1.047	Zinc finger CCHC domain-containing protein 8	GI-H
MELO3C022065.2	3.057	3.834	13.169	2.683	3.777	3.786	4.605	Nuclear matrix constituent protein 1	GI-H
MELO3C022059.2	0.471	0.414	2.601	0.396	0.606	0.769	0.913	Pentatricopeptide repeat-containing protein	GI-H
MELO3C022054.2	2.077	2.584	5.597	1.660	2.417	1.356	2.229	calcium/calmodulin-regulated receptor-like kinase 2	GI-H
MELO3C022045.2	0.434	1.428	5.161	10.826	0.903	6.744	0.787	Cytochrome P450	GI-H
MELO3C022042.2	2.745	4.339	9.599	1.562	4.574	1.926	3.558	DUF2039 family protein	GI-H

Gene ID	FPKM							Gene Description	Specific in episode
	FS	GI-M	GM-M	AN-M	GI-H	GM-H	AN-H		
MELO3C022040.2	0.292	0.605	2.266	NA	0.453	0.502	0.729	receptor-like protein kinase FERONIA	GI-H
MELO3C022038.2	10.435	11.369	28.729	8.766	13.731	5.128	11.827	eukaryotic translation initiation factor 3 subunit M	GI-H
MELO3C022036.2	2.549	1.845	6.999	1.452	1.793	1.505	1.743	protein LONGIFOLIA 2	GI-H
MELO3C022035.2	2.088	4.092	10.705	2.055	3.714	2.114	4.031	transcription initiation factor TFIID subunit 8	GI-H
MELO3C022032.2	4.485	4.951	16.191	3.284	3.802	3.031	4.627	Dentin sialophosphoprotein-like protein	GI-H
MELO3C033586.2	0.322	0.353	1.337	0.238	0.364	0.092	0.455	Unknown protein	GI-H
MELO3C022026.2	0.334	0.176	6.690	NA	0.208	NA	0.587	TPX2 protein family	GI-H
MELO3C022023.2	0.240	0.530	3.906	0.799	0.766	0.930	0.372	Membrane-anchored ubiquitin-fold protein	GI-H
MELO3C022022.2	1.011	2.220	3.751	1.970	1.055	4.141	0.884	Plant calmodulin-binding protein-related, putative isoform 1	GI-H
MELO3C022017.2	2.410	2.337	6.270	1.278	2.745	0.553	2.714	Ribosomal protein L18/L5	GI-H
MELO3C022012.2	0.333	0.362	3.108	NA	0.473	0.538	0.607	Protein ENHANCED DISEASE RESISTANCE 2-like	GI-H
MELO3C022009.2	1.109	3.853	11.855	4.740	3.180	5.682	1.994	Vesicle transport v-SNARE family protein	GI-H
MELO3C022003.2	0.995	1.878	5.561	2.432	1.448	NA	0.650	MYB-related transcription factor	GI-H
MELO3C021990.2	2.692	3.005	7.482	2.585	2.179	4.719	2.778	Defective in cullin neddylation protein	GI-H
MELO3C021984.2	2.886	2.268	8.367	1.775	3.339	0.929	3.109	NAP1-related protein 2-like	GI-H
MELO3C021479.2	0.970	2.531	2.575	2.440	0.985	0.966	1.094	AAA-ATPase ASD, mitochondrial-like	GI-H
MELO3C021486.2	0.674	0.590	1.506	0.502	0.587	0.409	0.577	BnaC06g35670D protein	GI-H
MELO3C021520.2	0.224	0.389	1.216	0.617	0.363	3.722	0.247	WAT1-related protein	GI-H
MELO3C033554.2	0.289	0.434	1.787	0.556	0.307	1.353	0.312	BnaA02g25840D protein	GI-H
MELO3C021549.2	2.614	4.531	11.848	3.208	3.876	4.593	4.572	serine/threonine-protein kinase fray2 isoform X2	GI-H
MELO3C021552.2	2.435	5.480	9.932	2.428	4.501	1.288	3.525	universal stress protein A-like protein	GI-H
MELO3C021554.2	1.874	3.491	5.146	2.506	2.514	2.452	1.900	Monodehydroascorbate reductase family protein	GI-H
MELO3C021570.2	1.803	1.292	8.764	NA	0.756	NA	1.463	BnaCnng71050D protein	GI-H
MELO3C021588.2	0.365	0.245	1.774	NA	0.273	NA	0.094	CRS1/YhbY (CRM) domain protein	GI-H
MELO3C021598.2	4.637	5.209	28.650	3.138	4.785	4.325	5.162	pre-rRNA-processing protein ESF2	GI-H
MELO3C021608.2	0.382	0.548	5.182	0.077	0.776	0.438	0.765	zinc finger protein CONSTANS-LIKE 6	GI-H
MELO3C021616.2	0.687	0.623	7.829	NA	1.391	0.787	0.627	DVL11	GI-H
MELO3C021622.2	0.346	0.960	1.774	1.391	0.884	0.779	0.252	Elicitor-responsive protein 1	GI-H
MELO3C021633.2	1.014	13.213	2.106	29.035	0.257	88.444	0.920	Actin family protein	GI-H
MELO3C021655.2	2.531	3.220	16.977	2.294	4.511	0.402	3.868	Transmembrane protein, putative	GI-H
MELO3C021657.2	0.725	1.345	7.712	0.897	0.974	0.897	0.978	Serine/threonine-protein phosphatase	GI-H

Gene ID	FPKM							Gene Description	Specific in episode
	FS	GI-M	GM-M	AN-M	GI-H	GM-H	AN-H		
MELO3C025449.2	2.929	3.608	7.238	2.025	3.500	3.034	2.616	Zinc finger C3H1 domain-containing protein, putative	GI-H
MELO3C025470.2	0.758	1.065	1.691	0.719	0.621	0.391	0.560	ribulose-1,5 bisphosphate carboxylase/oxygenase large subunit N-methyltransferase, chloroplastic isoform X2	GI-H
MELO3C025474.2	5.144	6.330	79.511	9.105	6.100	1.695	6.610	60S ribosomal protein L23a	GI-H
MELO3C025479.2	0.508	1.140	3.846	0.614	1.126	2.298	1.009	Phosphatidylinositol N-acetyglucosaminlytransferase subunit P-like protein	GI-H
MELO3C025488.2	1.579	1.489	7.479	0.768	1.479	0.951	1.125	transcription initiation factor TFIID subunit 1-like	GI-H
MELO3C025491.2	1.167	1.326	5.250	0.942	1.294	3.082	1.315	transcription initiation factor TFIID subunit 1-like	GI-H
MELO3C025492.2	0.392	0.865	4.923	0.645	0.515	0.906	0.830	transcription initiation factor TFIID subunit 1-like	GI-H
MELO3C025507.2	0.752	0.792	3.015	0.552	0.922	0.346	0.756	Nucleolar complex protein 2 homolog	GI-H
MELO3C002874.2	4.446	20.317	21.242	15.129	4.093	7.444	4.802	26 kDa phloem lectin	GI-H
MELO3C002879.2	0.833	1.167	4.901	1.847	1.198	0.716	0.929	Transmembrane 70, mitochondrial	GI-H
MELO3C002885.2	0.925	0.776	5.609	0.514	1.035	0.642	1.028	transcription factor GTE1	GI-H
MELO3C002931.2	0.551	1.215	3.398	1.022	0.723	2.015	0.694	Paired amphipathic helix SIN3-like protein	GI-H
MELO3C002943.2	0.415	0.931	2.588	1.345	0.756	0.827	0.834	Terpene cyclase/mutase family member	GI-H
MELO3C002951.2	0.626	0.854	1.796	0.631	0.420	1.178	0.619	Peptidyl-prolyl cis-trans isomerase	GI-H
MELO3C002960.2	1.147	0.963	2.390	0.626	1.053	0.267	0.884	anaphase-promoting complex subunit 2	GI-H
MELO3C002974.2	0.492	0.906	2.909	0.855	0.841	0.179	0.533	cell division topological specificity factor homolog, chloroplastic-like	GI-H
MELO3C002986.2	0.174	0.104	1.341	0.215	0.181	0.128	0.256	Sporulation protein RMD1	GI-H
MELO3C002995.2	1.258	2.120	6.798	2.585	2.229	3.834	2.516	Chaperone protein dnaJ 16	GI-H
MELO3C003005.2	1.201	1.378	2.734	0.701	1.229	0.414	0.961	Pentatricopeptide repeat-containing family protein	GI-H
MELO3C033863.2	0.111	0.703	1.814	0.521	0.779	0.073	0.242	Kelch-like protein	GI-H
MELO3C033861.2	1.301	2.389	6.523	1.977	2.837	0.331	2.345	Lipopolysaccharide core biosynthesis mannosyltransferase lpsB	GI-H
MELO3C033871.2	0.153	0.217	1.325	NA	0.597	NA	0.434	60S ribosomal protein L17, putative	GI-H
MELO3C003044.2	1.095	1.236	6.898	0.504	2.666	0.380	2.277	C2H2-like zinc finger protein	GI-H
MELO3C003047.2	1.476	1.072	6.682	1.379	2.128	0.684	1.524	serine/threonine-protein kinase MPS1 isoform X1	GI-H
MELO3C003048.2	0.872	1.211	5.170	0.383	0.902	0.197	1.033	Histone-lysine N-methyltransferase ASHR1-like protein	GI-H
MELO3C003061.2	1.897	1.652	15.469	3.419	1.732	2.708	2.104	Ribosomal protein S13	GI-H
MELO3C003066.2	0.718	4.227	6.453	2.165	1.041	1.401	1.166	17 kDa phloem lectin	GI-H
MELO3C022775.2	0.315	0.412	1.361	0.429	0.365	0.416	0.473	Small nuclear ribonucleoprotein-associated B	GI-H
MELO3C022799.2	0.995	1.209	2.877	0.559	1.095	2.890	0.888	sulfate transporter 1.3-like	GI-H

Gene ID	FPKM							Gene Description	Specific in episode
	FS	GI-M	GM-M	AN-M	GI-H	GM-H	AN-H		
MELO3C022803.2	0.161	0.222	1.010	0.230	0.213	0.264	0.163	At1g22140/F2E2_13	GI-H
MELO3C022813.2	0.294	0.482	2.310	0.545	0.413	0.521	0.411	THO complex subunit 1	GI-H
MELO3C022815.2	15.922	19.403	58.502	23.015	26.882	6.006	23.314	60S ribosomal protein L30-like	GI-H
MELO3C022819.2	1.329	1.206	5.671	1.152	1.820	0.360	1.400	Myosin heavy chain-like protein, putative	GI-H
MELO3C033898.2	0.195	0.117	1.011	0.307	0.325	0.504	0.136	zinc finger BED domain-containing protein RICESLEEPER 2-like	GI-H
MELO3C022860.2	0.285	0.292	1.947	0.401	0.506	NA	0.222	K(+) efflux antiporter 4 isoform X2	GI-H
MELO3C022863.2	0.685	0.610	1.839	0.625	0.712	0.551	0.677	DNA repair protein RAD4 isoform X1	GI-H
MELO3C022865.2	0.453	1.161	2.685	1.217	1.182	1.209	0.728	ADP-ribosylation factor, putative	GI-H
MELO3C025120.2	0.935	12.440	23.226	7.434	5.350	NA	2.505	rRNA N-glycosidase	GI-H
MELO3C025118.2	0.177	0.164	1.233	0.412	0.315	NA	0.155	Nascent polypeptide associated complex alpha	GI-H
MELO3C025101.2	0.961	12.161	17.675	48.782	3.560	95.106	3.456	Sucrose synthase	GI-H
MELO3C025091.2	0.803	1.038	3.595	0.074	0.805	NA	0.881	protein REVEILLE 1-like isoform X1	GI-H
MELO3C025085.2	0.677	0.765	1.786	0.145	0.601	0.799	0.197	Class I heat shock protein	GI-H
MELO3C025065.2	1.923	1.308	4.840	1.556	1.813	0.956	1.837	Protein TIC 40, chloroplastic	GI-H
MELO3C025063.2	0.383	0.606	1.717	0.255	0.625	0.424	0.291	N-alpha-acetyltransferase MAK3	GI-H
MELO3C025053.2	0.514	0.603	4.623	0.377	0.521	0.633	0.641	lysine-specific demethylase JMJ25	GI-H
MELO3C033732.2	10.977	10.955	52.193	17.405	10.076	8.599	9.992	50S ribosomal protein L2, chloroplastic	GI-H
MELO3C005080.2	1.916	1.842	4.158	1.097	2.066	0.777	1.886	helicase-like transcription factor CHR28	GI-H
MELO3C005085.2	0.328	0.467	1.638	0.197	0.272	0.112	0.207	Retrovirus-related Pol polyprotein from transposon TNT 1-94	GI-H
MELO3C005095.2	0.267	0.424	3.479	0.497	1.192	0.357	0.933	proline-rich receptor-like protein kinase PERK3	GI-H
MELO3C005100.2	0.311	0.286	2.719	0.195	0.201	0.435	0.285	Nuclear factor related to kappa-B-binding protein	GI-H
MELO3C033745.2	1.463	2.615	5.103	1.590	1.933	1.026	1.585	Maternal effect embryo arrest 22	GI-H
MELO3C033939.2	0.799	1.188	2.611	1.088	0.499	0.536	1.188	Unknown protein	GI-H
MELO3C005121.2	0.222	0.415	1.210	0.252	0.377	1.163	0.321	ADP-ribosylation factor GTPase-activating protein AGD12-like	GI-H
MELO3C005131.2	3.776	5.482	11.921	2.777	4.034	7.029	4.750	Plant calmodulin-binding-like protein	GI-H
MELO3C005136.2	1.454	2.955	6.015	2.710	2.130	9.342	2.695	Protein EARLY RESPONSIVE TO DEHYDRATION 15	GI-H
MELO3C005139.2	0.171	0.422	1.471	0.440	0.144	0.321	0.172	bifunctional endo-1,4-beta-xylanase XylA-like	GI-H
MELO3C005140.2	0.775	0.898	3.392	0.503	1.061	1.038	1.056	Pentatricopeptide repeat-containing family protein	GI-H
MELO3C005176.2	0.659	1.079	5.588	0.834	1.351	1.171	1.214	WPP domain-interacting tail-anchored protein 2	GI-H
MELO3C033963.2	2.057	2.523	9.087	2.322	1.790	1.039	2.265	Unknown protein	GI-H
MELO3C005180.2	0.172	0.306	1.586	0.291	0.337	0.152	0.216	Pentatricopeptide repeat-containing protein	GI-H

Gene ID	FPKM							Gene Description	Specific in episode
	FS	GI-M	GM-M	AN-M	GI-H	GM-H	AN-H		
MELO3C005183.2	2.007	2.352	8.737	2.212	2.817	3.257	3.845	Hepatocellular carcinoma-associated antigen 59 family protein, expressed	GI-H
MELO3C005192.2	1.283	2.073	5.186	1.395	2.073	0.730	1.911	Ribosomal protein L7Ae/L30e/S12e/Gadd45 family protein, putative	GI-H
MELO3C005194.2	0.219	0.312	1.763	0.283	0.303	0.503	0.344	NEFA-interacting nuclear protein	GI-H
MELO3C005198.2	2.096	1.863	4.213	1.295	1.891	2.150	1.176	Meiosis arrest female protein 1	GI-H
MELO3C005207.2	1.446	1.584	5.638	0.803	1.972	0.997	1.393	Homeodomain transcription factor superfamily protein, putative	GI-H
MELO3C005209.2	1.214	3.402	4.085	0.641	1.634	0.504	1.227	Phytosulfokine receptor, putative	GI-H
MELO3C033967.2	0.380	0.522	2.848	NA	0.694	NA	0.680	Pentatricopeptide repeat-containing protein, putative	GI-H
MELO3C005241.2	4.817	5.040	15.436	5.599	5.218	11.382	6.870	65-kDa microtubule-associated protein 6	GI-H
MELO3C005244.2	1.640	1.426	4.881	1.378	2.379	0.418	1.382	ABC transporter F family member 3	GI-H
MELO3C005275.2	5.997	9.637	38.359	4.352	4.959	2.502	2.950	nudix hydrolase 18, mitochondrial	GI-H
MELO3C005277.2	0.754	1.055	3.486	0.811	1.097	0.866	0.685	Ubiquitin-conjugating enzyme E2 variant 1B	GI-H
MELO3C033979.2	0.379	0.332	1.251	0.195	0.429	0.397	0.608	Unknown protein	GI-H
MELO3C005285.2	1.119	1.360	2.596	1.236	1.043	1.315	1.251	Mannosyltransferase	GI-H
MELO3C005296.2	0.564	0.991	3.906	0.873	1.119	0.549	0.854	Protein BCCIP homolog	GI-H
MELO3C005316.2	1.664	0.944	7.443	NA	1.140	NA	0.924	Heavy-metal-associated domain-containing family protein	GI-H
MELO3C005336.2	0.734	0.296	2.509	NA	0.303	NA	0.328	Two-component response regulator	GI-H
MELO3C005338.2	0.880	1.162	2.544	0.824	0.777	2.389	1.174	Alpha/beta fold hydrolase	GI-H
MELO3C033986.2	1.506	1.205	3.980	0.936	1.022	1.092	0.942	Mitochondrial import inner membrane translocase subunit TIM44-2	GI-H
MELO3C005366.2	3.311	4.957	14.917	3.382	5.892	1.446	5.249	Valine--tRNA ligase	GI-H
MELO3C005368.2	0.302	0.726	3.568	1.396	0.481	1.275	0.718	BAG family molecular chaperone regulator 3	GI-H
MELO3C005389.2	0.675	1.265	6.774	2.758	1.325	3.134	1.255	NADH dehydrogenase [ubiquinone] 1 alpha subcomplex subunit 12	GI-H
MELO3C005392.2	0.219	0.092	2.846	0.311	0.199	NA	0.467	molybdenum cofactor sulfurase	GI-H
MELO3C005417.2	1.365	1.164	4.124	0.706	1.034	NA	0.469	rRNA-processing protein EBP2, putative	GI-H
MELO3C005420.2	0.730	0.231	10.933	NA	0.736	NA	1.085	65-kDa microtubule-associated protein 3-like	GI-H
MELO3C005422.2	0.954	1.800	3.702	1.276	1.062	1.352	1.119	ADP-ribosylation factor-like	GI-H
MELO3C005430.2	3.143	2.372	29.802	1.508	5.489	NA	4.355	Zinc finger, CCHC-type	GI-H
MELO3C005447.2	1.966	2.194	5.131	2.456	2.020	5.632	1.453	SWR1 complex subunit 2	GI-H
MELO3C005461.2	0.176	0.226	2.575	0.260	0.152	0.199	0.227	Kokopelli	GI-H

Gene ID	FPKM							Gene Description	Specific in episode
	FS	GI-M	GM-M	AN-M	GI-H	GM-H	AN-H		
MELO3C005464.2	3.230	6.874	18.958	2.491	7.620	NA	5.849	Oleosin-like protein	GI-H
MELO3C005472.2	5.176	6.522	37.422	8.794	11.878	1.288	8.015	60S ribosomal protein L36	GI-H
MELO3C005477.2	0.856	0.938	3.483	0.587	1.572	0.373	1.434	Sn1-specific diacylglycerol lipase alpha	GI-H
MELO3C005479.2	0.788	0.725	11.054	NA	1.243	NA	1.131	Kinesin-like protein	GI-H
MELO3C005490.2	0.253	0.424	1.205	NA	0.448	NA	0.135	U-box domain-containing protein 35-like isoform X2	GI-H
MELO3C005497.2	0.318	0.501	1.872	NA	0.467	0.664	0.321	DNA repair protein REV1	GI-H
MELO3C005504.2	0.359	0.703	2.170	NA	0.559	0.336	0.329	Disease resistance family protein	GI-H
MELO3C005536.2	4.839	3.831	12.654	6.130	6.263	3.794	3.873	vesicle-associated protein 1-2	GI-H
MELO3C005557.2	0.580	0.822	4.630	NA	0.930	0.258	0.798	DNA-binding WRKY	GI-H
MELO3C005578.2	0.122	0.531	1.417	0.719	0.381	24.754	0.223	Amine oxidase	GI-H
MELO3C005589.2	2.661	5.234	9.824	3.225	3.275	2.922	3.394	ATP-dependent Clp protease proteolytic subunit	GI-H
MELO3C005602.2	1.778	3.020	14.079	5.459	2.521	0.569	3.064	Cytochrome P450, putative	GI-H
MELO3C005619.2	2.530	2.320	6.788	1.396	3.161	1.717	2.353	protein FAM63A isoform X1	GI-H
MELO3C005626.2	1.239	1.585	5.550	0.534	0.720	0.714	0.748	Nuclear pore complex protein Nup50	GI-H
MELO3C005646.2	0.526	1.103	2.776	1.382	0.379	0.589	0.905	Elicitor-responsive protein 3	GI-H
MELO3C005647.2	1.042	1.152	9.166	1.615	1.363	2.279	0.884	Methyl-cpg-binding domain-containing protein 13	GI-H
MELO3C005651.2	0.958	1.674	7.388	0.970	1.889	1.274	1.460	30S ribosomal S1	GI-H
MELO3C005653.2	1.447	2.600	18.986	1.097	1.654	NA	1.961	Remorin	GI-H
MELO3C005655.2	2.872	3.759	14.962	3.255	5.670	0.829	4.732	Myosin heavy chain-related protein	GI-H
MELO3C005675.2	0.234	0.535	3.109	NA	0.749	0.185	0.260	Bromo-adjacent-like (BAH) domain protein	GI-H
MELO3C005676.2	1.943	1.950	5.955	0.748	2.546	0.256	1.992	Bromo-adjacent domain-containing protein	GI-H
MELO3C005677.2	0.523	1.216	2.723	0.550	0.674	0.782	0.964	Mitochondrial carrier protein, putative	GI-H
MELO3C005678.2	6.904	4.786	18.486	3.621	7.055	2.910	8.567	kinesin-4 isoform X1	GI-H
MELO3C005679.2	4.951	7.209	21.324	8.480	8.427	6.651	8.084	Tyrosine--tRNA ligase	GI-H
MELO3C005681.2	0.362	0.814	4.612	0.466	1.198	1.554	0.914	Phosphatidylinositol N-acetylglucosaminlytransferase subunit P-like protein	GI-H
MELO3C005692.2	0.809	0.884	2.559	0.970	0.302	2.721	0.753	E3 ubiquitin-protein ligase At3g02290-like	GI-H
MELO3C005703.2	3.597	9.211	9.990	3.886	4.799	6.064	3.602	Beta-carotene 3-hydroxylase	GI-H
MELO3C005705.2	2.329	4.311	10.515	3.068	2.958	1.660	4.311	Mitogen-activated protein kinase	GI-H
MELO3C005715.2	2.887	3.200	8.367	2.715	3.001	2.267	2.745	PIN2/TERF1-interacting telomerase inhibitor 1	GI-H
MELO3C005731.2	1.620	1.718	7.056	1.077	2.145	0.634	1.144	Intracellular protein transport protein USO1-like protein	GI-H

Gene ID	FPKM							Gene Description	Specific in episode
	FS	GI-M	GM-M	AN-M	GI-H	GM-H	AN-H		
MELO3C005749.2	1.345	1.658	4.544	1.710	1.814	1.828	2.168	long chain acyl-CoA synthetase 4-like	GI-H
MELO3C033830.2	0.217	0.042	1.969	0.180	0.239	0.237	0.052	Glycosyl transferase, family 31	GI-H
MELO3C005774.2	0.247	0.397	1.858	NA	0.428	0.246	0.455	dof zinc finger protein DOF4.6-like	GI-H
MELO3C005775.2	1.181	2.222	4.838	0.831	1.401	1.652	1.225	BTB/POZ domain protein	GI-H
MELO3C005777.2	3.087	4.945	11.472	5.156	4.357	11.758	5.365	serine/threonine-protein kinase WNK8-like isoform X1	GI-H
MELO3C005789.2	0.260	0.408	1.712	0.384	0.580	0.408	0.647	Protein BRANCHLESS TRICHOME	GI-H
MELO3C005802.2	0.707	2.523	4.576	3.079	2.092	0.813	2.131	Mitochondrial import inner membrane translocase subunit Tim13	GI-H
MELO3C005815.2	0.680	0.767	1.732	2.786	0.493	1.468	0.843	Trihelix transcription factor GT-2	GI-H
MELO3C005828.2	0.463	0.978	3.617	0.521	1.437	0.867	0.929	SKP1-like protein 21	GI-H
MELO3C005875.2	0.217	0.430	3.017	0.299	0.454	0.740	0.333	Curved DNA-binding protein	GI-H
MELO3C005876.2	1.426	2.849	7.416	1.609	1.959	5.245	1.820	cyclic nucleotide-gated ion channel 1	GI-H
MELO3C005880.2	0.967	1.546	4.838	2.392	0.814	2.312	1.704	At5g24165	GI-H
MELO3C005883.2	1.202	1.520	10.553	0.510	1.566	NA	1.593	Zinc finger protein CONSTANS	GI-H
MELO3C005887.2	2.055	2.107	7.514	1.511	3.007	2.820	1.469	Defective in meristem silencing 3	GI-H
MELO3C005891.2	0.153	0.524	2.269	0.237	0.178	0.465	0.301	Transmembrane protein, putative	GI-H
MELO3C005907.2	3.659	8.727	12.727	6.717	3.815	3.984	4.254	cytochrome b5	GI-H
MELO3C005912.2	7.699	8.956	31.814	10.140	10.358	5.616	8.170	Ubiquitin	GI-H
MELO3C005924.2	0.201	0.255	1.852	0.385	0.448	0.476	0.337	ABC transporter A family member 1	GI-H
MELO3C012493.2	0.347	0.863	3.463	1.377	0.417	2.285	0.545	Choline/ethanolamine kinase	GI-H
MELO3C012475.2	0.994	1.026	2.927	0.552	0.481	0.934	0.857	phosphatidylinositol 4-phosphate 5-kinase 6-like	GI-H
MELO3C012466.2	0.207	NA	1.921	NA	0.406	NA	0.265	Cytochrome C oxidase assembly factor	GI-H
MELO3C012461.2	2.597	4.123	13.178	3.338	2.182	5.959	2.386	Protein LNK2	GI-H
MELO3C012441.2	8.176	8.351	17.270	4.386	6.593	9.993	7.100	Nuclear receptor corepressor 1	GI-H
MELO3C012434.2	0.290	0.477	1.191	0.312	0.094	0.491	0.230	LOW QUALITY PROTEIN: aminodeoxychorismate synthase, chloroplastic	GI-H
MELO3C012426.2	2.585	1.942	7.448	2.058	3.256	1.343	2.309	GATA transcription factor	GI-H
MELO3C012413.2	0.925	2.562	6.188	1.849	2.354	2.296	2.084	OTU-like cysteine protease domain containing protein	GI-H
MELO3C012399.2	0.956	0.958	3.409	0.484	1.596	0.184	1.046	Translation initiation factor IF-2, putative isoform 2	GI-H
MELO3C012376.2	3.372	3.950	8.645	3.878	3.470	6.138	2.755	LOW QUALITY PROTEIN: 3-phosphoinositide-dependent protein kinase 1	GI-H
MELO3C012368.2	2.149	3.137	10.709	4.302	1.727	6.025	1.918	transcriptional activator DEMETER isoform X1	GI-H

Gene ID	FPKM							Gene Description	Specific in episode
	FS	GI-M	GM-M	AN-M	GI-H	GM-H	AN-H		
MELO3C012350.2	1.031	1.712	3.201	1.677	1.219	2.781	1.277	iron-sulfur assembly protein IscA-like 1, mitochondrial	GI-H
MELO3C012349.2	0.566	0.733	8.779	0.361	1.679	NA	0.736	replication protein A 14 kDa subunit B-like	GI-H
MELO3C012338.2	2.378	3.220	8.444	3.009	3.963	7.590	3.259	exocyst complex component EXO70A1	GI-H
MELO3C012334.2	0.696	1.012	1.920	0.894	0.799	2.052	0.675	Apoptosis-inducing factor-like protein A	GI-H
MELO3C012333.2	0.835	2.213	13.212	2.612	2.109	3.141	1.452	charged multivesicular body protein 5	GI-H
MELO3C012332.2	0.437	0.364	3.199	0.422	0.498	0.152	0.690	Basic-leucine zipper transcription factor family protein	GI-H
MELO3C012326.2	4.074	4.582	11.170	5.003	4.652	7.274	3.831	Calcium-dependent protein kinase, putative	GI-H
MELO3C012309.2	0.142	0.335	1.557	0.391	0.335	0.166	0.186	DNA repair REX1-B protein	GI-H
MELO3C012297.2	0.778	1.085	6.564	0.657	0.899	0.948	0.386	Repressor of RNA polymerase III transcription	GI-H
MELO3C012294.2	1.002	1.841	10.607	1.387	1.911	1.390	0.780	sarcoplasmic reticulum histidine-rich calcium-binding protein	GI-H
MELO3C012244.2	0.466	0.299	2.549	NA	0.870	0.339	0.882	DNA binding protein	GI-H
MELO3C012242.2	0.855	0.769	5.921	1.497	2.055	NA	1.633	ethylene-responsive transcription factor ERF118	GI-H
MELO3C012215.2	1.200	1.168	6.812	0.426	1.153	0.718	1.073	NAC domain-containing protein	GI-H
MELO3C012211.2	0.238	1.072	6.192	0.518	0.858	1.072	0.444	Coiled-coil domain-containing protein 130	GI-H
MELO3C012209.2	1.697	2.114	3.931	2.393	1.473	92.767	1.447	Protein CHUP1, chloroplastic	GI-H
MELO3C012196.2	2.172	2.665	7.608	1.929	3.182	3.987	2.754	Phospholipid-transporting ATPase	GI-H
MELO3C012184.2	0.567	0.705	3.080	0.518	0.676	1.191	0.573	Uridine kinase	GI-H
MELO3C012180.2	0.449	1.036	2.152	0.495	0.454	0.527	0.664	ruBisCO large subunit-binding protein subunit beta, chloroplastic-like	GI-H
MELO3C012175.2	0.804	1.454	2.558	0.421	0.633	0.957	0.587	Thioredoxin-like protein aaed1, chloroplastic	GI-H
MELO3C012169.2	0.873	1.213	3.739	0.787	0.184	0.731	0.657	BSD domain-containing protein	GI-H
MELO3C012167.2	0.417	1.208	2.244	1.687	0.626	0.627	0.659	Formate--tetrahydrofolate ligase	GI-H
MELO3C034075.2	2.094	3.549	48.409	NA	4.587	NA	2.841	protein CRABS CLAW	GI-H
MELO3C012153.2	1.031	1.006	3.689	1.081	0.798	0.561	1.768	Pentatricopeptide repeat-containing family protein	GI-H
MELO3C012146.2	1.693	3.628	15.492	2.416	3.024	2.012	3.687	14-3-3 protein, putative	GI-H
MELO3C012133.2	1.383	1.691	8.114	1.449	2.716	0.595	1.890	At2g03350	GI-H
MELO3C012127.2	1.996	1.806	6.322	1.218	2.178	0.895	1.920	protein argonaute 7	GI-H
MELO3C012117.2	1.995	4.125	12.508	4.219	3.930	2.238	3.354	RanBP2-type zinc finger protein	GI-H
MELO3C012115.2	0.183	0.326	1.940	0.248	0.577	0.530	0.628	Histone-lysine N-methyltransferase SMYD3	GI-H
MELO3C012112.2	0.654	1.092	4.053	3.130	1.721	2.186	0.435	cAMP-regulated phosphoprotein-like protein	GI-H
MELO3C012105.2	0.587	0.333	2.394	NA	0.368	NA	0.143	Myb transcription factor	GI-H

Gene ID	FPKM							Gene Description	Specific in episode
	FS	GI-M	GM-M	AN-M	GI-H	GM-H	AN-H		
MELO3C012102.2	0.420	0.695	1.624	0.256	0.390	0.268	0.172	50S ribosomal protein L18	GI-H
MELO3C012100.2	0.350	0.883	6.214	9.784	0.520	2.355	0.323	ribonuclease 3-like	GI-H
MELO3C012082.2	0.443	0.984	4.808	1.029	0.548	1.675	0.681	histone-lysine N-methyltransferase SETD1A	GI-H
MELO3C012079.2	0.998	1.069	11.755	1.208	2.005	0.803	1.198	Homeobox-leucine zipper family protein	GI-H
MELO3C012077.2	0.549	0.634	3.726	0.179	1.195	0.127	0.697	Myb family transcription factor family protein	GI-H
MELO3C012073.2	1.391	2.994	4.609	1.992	1.696	3.950	1.838	SNF1-related kinase regulatory subunit gamma 1	GI-H
MELO3C012036.2	5.728	7.864	22.928	5.781	5.411	11.899	6.745	Paired amphipathic helix SIN3-like protein	GI-H
MELO3C012033.2	1.866	3.720	18.575	4.371	1.801	1.678	2.238	Neurofilament heavy protein	GI-H
MELO3C012018.2	0.520	0.365	1.684	0.759	0.524	0.508	0.819	protein ALTERED XYLOGLUCAN 4	GI-H
MELO3C012014.2	1.489	1.283	5.842	1.850	1.789	1.395	0.693	Serine/threonine-protein kinase WNK-related	GI-H
MELO3C012004.2	0.458	1.029	2.260	0.443	0.423	12.190	0.457	Xyloglucan endotransglucosylase/hydrolase	GI-H
MELO3C012003.2	0.788	3.444	3.005	NA	0.840	5.513	0.312	RING-type E3 ubiquitin transferase	GI-H
MELO3C012000.2	0.220	0.621	3.946	0.324	0.610	0.450	0.242	ubiquitin-like-specific protease 1D isoform X1	GI-H
MELO3C011999.2	2.463	2.549	6.657	2.316	2.157	1.716	1.891	pre-mRNA-processing factor 17 isoform X1	GI-H
MELO3C011997.2	0.707	3.268	2.703	2.617	1.333	3.935	0.920	Carbonic anhydrase	GI-H
MELO3C011969.2	0.815	1.734	7.746	1.494	0.857	0.459	1.328	Pentatricopeptide repeat-containing family protein	GI-H
MELO3C011941.2	0.474	0.782	3.469	2.693	0.744	2.980	0.349	Lysine ketoglutarate reductase trans-splicing protein	GI-H
MELO3C011924.2	1.347	1.072	4.197	0.826	0.957	0.650	0.924	protein GLE1	GI-H
MELO3C011920.2	10.797	6.517	116.780	12.509	6.137	5.108	4.493	50S ribosomal protein L14, chloroplastic	GI-H
MELO3C011907.2	0.748	1.077	4.452	NA	1.999	0.812	1.292	RING finger protein	GI-H
MELO3C011906.2	5.121	9.776	34.815	3.930	14.336	2.483	12.054	LRR receptor-like kinase	GI-H
MELO3C011904.2	0.964	1.172	9.129	1.347	1.838	1.279	1.043	Leucine-rich repeat-containing protein DDB_G0290503, putative isoform 1	GI-H
MELO3C011898.2	3.230	3.657	16.202	3.552	5.164	4.673	5.288	signal recognition particle subunit SRP72	GI-H
MELO3C011894.2	1.065	0.975	3.945	1.502	1.226	0.374	1.363	ABC transporter C family member 3	GI-H
MELO3C011882.2	0.908	1.244	3.700	0.765	0.807	0.830	0.924	RING-type E3 ubiquitin transferase	GI-H
MELO3C011854.2	1.088	3.451	5.069	3.320	1.089	2.639	1.242	Cytochrome oxidase assembly 3, mitochondrial	GI-H
MELO3C011853.2	0.782	1.126	1.897	1.953	0.794	2.564	0.941	(+)-neomenthol dehydrogenase	GI-H
MELO3C011839.2	0.687	1.370	4.560	1.524	0.876	0.343	1.242	Basic-leucine zipper (BZIP) transcription factor family protein	GI-H
MELO3C011833.2	0.407	0.974	7.749	0.684	0.778	0.133	0.638	transcription termination factor MTERF5, chloroplastic-like	GI-H
MELO3C011829.2	0.090	0.116	1.278	0.094	0.149	NA	0.194	transcription termination factor MTERF5, chloroplastic-like	GI-H

Gene ID	FPKM							Gene Description	Specific in episode
	FS	GI-M	GM-M	AN-M	GI-H	GM-H	AN-H		
MELO3C011822.2	0.854	0.995	2.788	0.609	0.943	0.858	1.348	Ion channel dmi1	GI-H
MELO3C011821.2	0.445	0.460	3.080	0.331	0.518	0.441	0.358	Bromodomain-containing protein, putative	GI-H
MELO3C011818.2	2.809	5.960	8.428	1.556	2.800	2.645	2.112	C2 domain-containing family protein	GI-H
MELO3C011817.2	0.307	0.768	2.305	0.507	0.548	0.249	0.382	peptide chain release factor 1	GI-H
MELO3C011814.2	0.499	0.921	2.241	NA	0.576	0.548	0.461	Receptor-like protein kinase, putative	GI-H
MELO3C011785.2	0.490	0.551	2.327	0.293	0.793	0.163	0.720	transcription termination factor MTERF5, chloroplastic-like	GI-H
MELO3C011797.2	1.285	1.070	8.509	1.256	0.797	1.557	1.733	dr1-associated corepressor	GI-H
MELO3C011793.2	0.748	0.365	6.147	NA	0.712	0.559	0.544	transcription termination factor MTERF9, chloroplastic-like	GI-H
MELO3C011783.2	0.232	0.416	1.073	NA	0.203	0.259	0.225	serine/threonine-protein kinase CDG1 isoform X1	GI-H
MELO3C011781.2	0.470	0.734	2.898	0.429	0.319	0.320	0.520	SAC3 family protein C	GI-H
MELO3C011759.2	0.251	0.232	1.447	0.198	0.170	0.243	0.082	transcription factor DIVARICATA	GI-H
MELO3C011755.2	2.610	2.235	8.543	2.154	3.614	4.454	2.821	Myosin-related family protein	GI-H
MELO3C011753.2	0.452	0.413	7.319	0.410	1.836	NA	0.599	transcription termination factor MTERF9, chloroplastic-like	GI-H
MELO3C011737.2	0.373	0.546	2.032	NA	0.439	0.363	0.254	cytokinin dehydrogenase 6	GI-H
MELO3C011721.2	0.408	0.427	5.313	0.380	0.217	0.443	0.309	zinc finger CCCH domain-containing protein 11	GI-H
MELO3C011714.2	0.527	1.697	2.621	1.361	1.093	0.496	0.949	Glutathione S-transferase	GI-H
MELO3C023808.2	2.492	3.319	7.017	2.156	2.675	4.632	3.209	SAC3/GANP/Nin1/mts3/eIF-3 p25 family isoform 1	GI-H
MELO3C023813.2	0.930	1.410	5.592	0.951	1.475	0.759	1.554	At5g13560	GI-H
MELO3C023816.2	0.597	0.270	3.673	0.439	1.833	NA	0.793	3-ketoacyl-CoA synthase	GI-H
MELO3C023829.2	1.545	2.615	3.938	2.082	1.896	2.046	1.962	Beta-hexosaminidase	GI-H
MELO3C023835.2	11.284	16.228	109.919	21.707	26.966	4.044	15.642	60S ribosomal protein L23a	GI-H
MELO3C023862.2	3.496	4.918	17.920	4.039	5.447	2.192	4.169	Factor of DNA methylation 1	GI-H
MELO3C023869.2	3.353	5.704	12.950	4.369	3.127	10.615	3.243	telomere repeat-binding protein 4 isoform X2	GI-H
MELO3C034277.2	0.643	0.748	2.698	0.487	0.876	0.164	0.776	Beta-glucosidase	GI-H
MELO3C026567.2	0.407	0.555	2.200	0.539	0.589	0.383	0.442	Pentatricopeptide repeat-containing protein At4g18975, chloroplastic	GI-H
MELO3C025140.2	1.864	2.494	30.861	3.916	5.196	0.160	2.390	60S ribosomal protein L35a	GI-H
MELO3C025148.2	1.707	2.161	13.790	1.868	4.834	1.227	2.562	Adenylosuccinate lyase	GI-H
MELO3C025151.2	1.321	1.169	5.412	NA	1.843	0.481	2.511	transcription factor DIVARICATA	GI-H
MELO3C025156.2	0.685	2.141	14.928	3.278	2.563	0.888	1.963	Microsomal signal peptidase 25 kDa subunit family protein	GI-H
MELO3C034330.2	0.525	0.528	2.536	1.174	0.394	0.528	1.139	COP9 signalosome complex subunit 5b-like	GI-H

Gene ID	FPKM							Gene Description	Specific in episode
	FS	GI-M	GM-M	AN-M	GI-H	GM-H	AN-H		
MELO3C025192.2	0.616	0.889	3.196	NA	0.866	0.155	0.795	Peptidyl-prolyl cis-trans isomerase	GI-H
MELO3C025194.2	4.005	5.203	14.907	4.053	7.092	1.454	4.711	Phosphoinositide phospholipase C	GI-H
MELO3C026789.2	0.442	0.463	1.571	NA	0.366	0.387	0.374	N-(5-phosphoribosyl)anthranilate isomerase	GI-H
MELO3C026797.2	0.412	0.404	4.077	NA	0.080	NA	0.321	Pentatricopeptide repeat-containing protein At5g38730	GI-H
MELO3C020033.2	0.849	1.257	5.226	0.780	1.422	NA	1.033	Ribosomal RNA-processing protein 7	GI-H
MELO3C020045.2	0.402	0.486	1.640	0.200	0.294	0.338	0.262	Pentatricopeptide repeat-containing protein	GI-H
MELO3C020055.2	5.691	8.381	14.571	2.131	5.407	2.890	6.390	histidine kinase 4-like	GI-H
MELO3C020077.2	0.484	0.657	1.841	0.535	0.834	0.412	0.552	folate synthesis bifunctional protein, mitochondrial-like	GI-H
MELO3C034164.2	0.304	0.445	3.997	0.181	1.146	0.129	0.378	Origin recognition complex subunit 4	GI-H
MELO3C020095.2	0.866	1.343	3.018	1.095	0.861	2.197	0.742	Neuronal PAS domain protein	GI-H
MELO3C020097.2	1.776	1.959	41.148	4.757	1.809	1.496	2.777	Ribosomal protein S7	GI-H
MELO3C020109.2	2.857	2.640	14.777	1.226	2.069	1.580	2.846	Nop53 protein	GI-H
MELO3C020110.2	0.756	1.011	2.904	1.692	0.851	0.667	0.855	Nucleic acid-binding, OB-fold	GI-H
MELO3C020114.2	0.237	0.361	1.911	1.299	0.645	0.882	0.492	phosphoglucan phosphatase LSF2, chloroplastic isoform X3	GI-H
MELO3C034379.2	0.144	0.239	1.261	NA	0.232	0.057	0.084	BEACH domain-containing protein C2	GI-H
MELO3C020148.2	0.824	0.389	6.941	0.501	1.373	0.458	0.570	Cation-transporting ATPase	GI-H
MELO3C020154.2	1.219	0.946	14.569	2.164	1.346	0.823	1.715	aminoacyl tRNA synthase complex-interacting multifunctional protein 1 isoform X1	GI-H
MELO3C034188.2	0.481	0.703	1.829	0.391	0.778	0.464	0.684	Rhodanese-related sulfurtransferase	GI-H
MELO3C020181.2	3.185	2.863	13.018	3.033	3.862	1.426	3.721	COP1-interacting-like protein, putative	GI-H
MELO3C026815.2	5.176	7.035	27.204	1.758	10.123	0.983	9.630	RNA-dependent RNA polymerase	GI-H
MELO3C022540.2	1.060	1.851	3.140	1.696	1.412	2.464	1.391	NADH dehydrogenase [ubiquinone] 1 beta subcomplex subunit 7	GI-H
MELO3C022554.2	0.343	0.402	1.323	0.329	0.584	0.055	0.368	THUMP domain-containing protein 1	GI-H
MELO3C022586.2	1.022	0.953	9.294	1.333	0.989	0.465	1.187	Sm-like protein lsm36b	GI-H
MELO3C034414.2	0.373	0.203	3.637	NA	0.125	0.287	0.224	Aspartic proteinase	GI-H
MELO3C018402.2	0.289	0.539	2.464	0.410	0.739	0.279	0.465	p-loop nucleoside triphosphate hydrolase superfamily protein	GI-H
MELO3C018397.2	0.105	0.373	1.771	0.158	0.398	0.184	0.315	p-loop nucleoside triphosphate hydrolase superfamily protein	GI-H
MELO3C018395.2	3.740	4.173	10.400	2.079	5.039	1.061	3.851	SNF2 domain-containing protein CLASSY 1-like	GI-H
MELO3C018386.2	0.561	0.607	1.810	0.562	0.876	0.269	0.813	Cytosolic endo-beta-N-acetylglucosaminidase	GI-H
MELO3C018364.2	2.112	4.590	7.301	3.193	1.911	3.917	1.358	Differentiation-associated protein 1, putative isoform 1	GI-H
MELO3C034445.2	0.432	0.527	1.605	0.240	0.252	0.247	0.271	Transposon protein, putative, CACTA, En/Spm sub-class	GI-H

Gene ID	FPKM							Gene Description	Specific in episode
	FS	GI-M	GM-M	AN-M	GI-H	GM-H	AN-H		
MELO3C018356.2	1.074	3.214	9.270	1.323	0.859	0.691	0.905	Protein WVD2-like 7	GI-H
MELO3C018345.2	0.495	0.582	1.770	0.476	0.440	1.120	0.383	Uridine-cytidine kinase C	GI-H
MELO3C018327.2	0.404	0.946	7.164	0.759	1.267	0.662	0.849	annexin D5-like	GI-H
MELO3C018308.2	0.181	0.405	1.986	0.673	0.548	0.630	0.551	Serine hydroxymethyltransferase	GI-H
MELO3C018283.2	0.310	0.395	1.071	0.307	0.318	0.248	0.383	FAD/NAD(P)-binding oxidoreductase family protein	GI-H
MELO3C018272.2	2.896	2.759	19.119	1.852	3.922	0.292	4.426	Ribosome production factor 2 like	GI-H
MELO3C018265.2	0.286	0.270	1.684	0.224	0.221	0.274	0.277	ADP-ribosylation factor GTPase-activating protein	GI-H
MELO3C018262.2	0.251	0.344	1.311	0.283	0.153	0.450	0.266	Nascent polypeptide-associated complex subunit beta	GI-H
MELO3C016630.2	6.195	10.890	22.528	29.147	6.630	26.715	6.470	cytochrome c	GI-H
MELO3C026421.2	0.157	0.249	1.720	0.293	0.293	0.101	0.369	30S ribosomal protein S17-like	GI-H
MELO3C026432.2	0.120	0.949	10.006	3.793	0.636	4.692	0.366	Caleosin-related family protein	GI-H
MELO3C022885.2	0.697	0.764	7.111	0.721	0.884	0.691	1.152	methyl-CpG-binding domain protein 4-like protein	GI-H
MELO3C022897.2	1.024	0.700	3.801	0.292	1.236	0.171	1.036	Kinesin-like protein	GI-H
MELO3C022910.2	0.625	0.264	1.770	0.442	0.642	0.139	0.504	Structural maintenance of chromosomes protein	GI-H
MELO3C022920.2	1.096	1.035	2.444	0.714	1.061	0.405	1.170	squamous cell carcinoma antigen recognized by T-cells 3 isoform X1	GI-H
MELO3C022930.2	0.331	0.208	1.183	0.167	0.399	0.101	0.241	mitotic spindle checkpoint protein MAD1	GI-H
MELO3C022945.2	0.397	0.854	2.266	0.409	0.721	NA	0.401	mediator-associated protein 1-like	GI-H
MELO3C022946.2	0.299	0.765	1.992	0.655	0.759	0.925	0.495	Serine/threonine-protein phosphatase 7 long form-like protein	GI-H
MELO3C022956.2	1.059	1.006	2.232	0.643	0.673	0.430	0.788	tRNA-specific adenosine deaminase-like protein 3	GI-H
MELO3C026446.2	1.397	1.355	3.273	0.618	1.482	0.552	1.315	Pentatricopeptide repeat-containing protein family	GI-H
MELO3C026448.2	1.591	1.255	8.400	NA	2.494	0.205	2.491	Cytosine-specific methyltransferase	GI-H
MELO3C026449.2	1.681	3.200	26.036	3.500	4.437	1.826	3.676	Serine--tRNA ligase	GI-H
MELO3C026691.2	0.929	1.418	2.243	1.023	0.954	3.574	0.912	Paramyosin	GI-H
MELO3C026683.2	0.299	0.453	1.433	0.364	0.610	0.522	0.332	Transcription factor-related family protein	GI-H
MELO3C023189.2	0.432	0.340	2.835	0.245	0.279	0.262	0.285	Cyclin family protein	GI-H
MELO3C023191.2	0.761	0.760	2.004	0.832	0.334	0.434	0.566	DNA-binding protein BIN4 isoform X2	GI-H
MELO3C023202.2	0.705	0.932	3.388	0.458	0.902	0.470	0.773	DDT domain-containing protein DDB_G0282237 isoform X1	GI-H
MELO3C023205.2	1.139	1.078	2.516	0.884	0.979	0.985	0.977	peptidyl-prolyl cis-trans isomerase G isoform X2	GI-H
MELO3C023221.2	0.551	0.738	2.093	0.418	0.692	0.751	0.393	DNA cross-link repair family protein	GI-H
MELO3C023228.2	0.528	1.539	2.908	0.357	1.030	0.480	1.024	U2 small nuclear ribonucleoprotein auxiliary factor-like protein	GI-H

Gene ID	FPKM							Gene Description	Specific in episode
	FS	GI-M	GM-M	AN-M	GI-H	GM-H	AN-H		
MELO3C023265.2	0.323	0.504	4.690	0.740	0.917	1.407	0.283	Unknown protein	GI-H
MELO3C023266.2	0.227	1.262	1.747	1.027	0.595	3.592	0.625	Trihelix transcription factor GT-2	GI-H
MELO3C023267.2	0.521	0.416	1.621	0.910	0.637	0.738	0.475	DnaJ-like protein	GI-H
MELO3C023279.2	2.420	3.352	7.010	4.581	2.753	5.915	3.464	Golgin candidate 5	GI-H
MELO3C023293.2	0.409	0.191	1.308	0.180	0.299	0.379	0.257	Dipeptide transport ATP-binding protein dppF	GI-H
MELO3C023301.2	1.458	1.384	4.689	1.555	1.606	0.600	1.629	glutamine--tRNA ligase-like	GI-H
MELO3C023319.2	1.601	2.147	6.605	2.304	2.363	2.030	2.212	Chaperone protein dnaJ 10	GI-H
MELO3C023325.2	1.404	2.027	5.169	1.332	1.700	2.048	1.585	SUMO-activating enzyme subunit 2	GI-H
MELO3C023332.2	0.927	1.061	5.873	NA	1.308	0.255	1.530	protein CHROMATIN REMODELING 35-like	GI-H
MELO3C023334.2	0.476	1.375	5.006	0.738	0.874	1.325	0.761	zinc finger CCHC domain-containing protein 10	GI-H
MELO3C023351.2	0.287	0.144	3.347	0.343	0.408	0.173	0.220	Altered inheritance of mitochondria protein	GI-H
MELO3C023357.2	2.039	5.425	14.145	7.137	4.285	7.339	4.402	NADH dehydrogenase [ubiquinone] 1 beta subcomplex subunit 9	GI-H
MELO3C020972.2	5.374	6.798	47.744	6.717	8.581	5.320	6.570	26S proteasome non-ATPase regulatory subunit 12	GI-H
MELO3C020959.2	0.447	0.748	2.593	0.854	0.800	0.637	0.779	Developmentally regulated GTP-binding protein, putative	GI-H
MELO3C020947.2	0.353	0.259	1.502	NA	0.191	0.140	0.422	Pentatricopeptide repeat-containing family protein	GI-H
MELO3C020943.2	0.401	0.744	2.025	0.666	0.493	0.695	0.613	Upstream activation factor subunit spp27	GI-H
MELO3C020940.2	0.252	0.408	1.388	0.211	0.528	0.342	0.429	Pentatricopeptide repeat-containing family protein	GI-H
MELO3C020934.2	0.573	2.644	15.024	1.937	1.818	NA	1.807	Patatin	GI-H
MELO3C020926.2	0.851	0.452	4.024	NA	1.197	NA	1.668	Zinc-finger domain of monoamine-oxidase A repressor R1, putative	GI-H
MELO3C020924.2	0.915	0.892	2.720	1.355	1.240	1.093	1.297	zinc finger protein 830 isoform X1	GI-H
MELO3C020899.2	2.907	5.885	9.984	4.644	3.732	8.016	3.705	RCC_reductase domain-containing protein/EF_hand_5 domain-containing protein	GI-H
MELO3C020867.2	0.139	0.117	1.787	0.231	0.138	0.106	0.092	FRIGIDA-like protein	GI-H
MELO3C020864.2	0.282	0.547	2.611	0.732	0.866	2.097	1.110	homeobox protein 6	GI-H
MELO3C020854.2	1.053	0.908	4.525	0.719	1.137	0.518	1.208	Histone-lysine N-methyltransferase	GI-H
MELO3C020850.2	0.944	1.079	10.400	1.003	1.456	0.135	0.987	Filament-like plant protein 7	GI-H
MELO3C020849.2	1.319	1.408	6.607	0.563	1.743	0.157	1.348	U3 small nucleolar RNA-associated protein 18 homolog	GI-H
MELO3C020845.2	0.066	0.253	1.729	0.210	0.145	0.118	0.145	Glycosyltransferase	GI-H
MELO3C020830.2	1.088	0.513	5.478	0.162	1.658	NA	1.085	BZIP transcription factor, putative (DUF630 and DUF632)	GI-H
MELO3C020828.2	0.407	0.574	3.789	1.098	0.957	0.674	0.545	Translation initiation factor if-3, putative	GI-H
MELO3C020824.2	1.483	2.379	13.103	1.949	2.783	3.243	1.633	Pre-mRNA-splicing factor syf2	GI-H

Gene ID	FPKM							Gene Description	Specific in episode
	FS	GI-M	GM-M	AN-M	GI-H	GM-H	AN-H		
MELO3C020815.2	0.740	0.439	2.104	0.623	0.564	0.390	0.666	protein MCM10 homolog isoform X1	GI-H
MELO3C020804.2	0.492	3.108	7.614	NA	2.317	0.159	1.074	endo-1,4-beta-xylanase B	GI-H
MELO3C020778.2	0.766	0.812	3.835	0.517	1.030	0.173	0.806	Urb2/Npa2 family protein	GI-H
MELO3C021947.2	0.468	0.359	1.055	0.217	0.414	0.103	0.500	Pentatricopeptide repeat-containing protein	GI-H
MELO3C021939.2	1.170	2.636	6.424	0.563	1.084	1.819	0.751	Extra-large guanine nucleotide binding protein, putative, expressed	GI-H
MELO3C021934.2	2.931	6.323	20.147	3.205	0.532	5.529	0.495	Unknown protein	GI-H
MELO3C021930.2	1.830	3.442	8.237	1.777	2.630	1.183	2.324	B3 domain-containing transcription factor VRN1-like	GI-H
MELO3C021929.2	0.598	0.794	4.528	0.707	0.813	0.844	0.796	B3 domain-containing transcription factor VRN1-like	GI-H
MELO3C021920.2	1.809	0.931	12.007	0.501	3.705	0.068	3.187	phragmoplast orienting kinesin 2	GI-H
MELO3C021912.2	0.758	0.475	2.537	0.227	0.324	NA	0.462	Peptidyl-prolyl cis-trans isomerase	GI-H
MELO3C021898.2	1.397	2.365	9.978	2.052	1.963	3.472	1.756	RING/FYVE/PHD zinc finger protein, putative	GI-H
MELO3C021891.2	0.653	0.946	1.861	0.761	0.820	1.382	0.565	dehydrodolichyl diphosphate syntase complex subunit NUS1 isoform X1	GI-H
MELO3C021887.2	0.912	0.878	3.043	0.598	0.933	1.154	0.878	THO complex subunit 7A-like	GI-H
MELO3C021886.2	7.215	12.990	42.294	3.522	13.591	3.405	12.265	S-norcoclaurine synthase 1-like	GI-H
MELO3C021884.2	1.560	2.885	8.214	NA	2.227	NA	2.509	S-norcoclaurine synthase 1-like	GI-H
MELO3C034624.2	0.457	0.507	2.729	0.340	0.284	0.403	0.799	Pentatricopeptide repeat-containing family protein	GI-H
MELO3C021858.2	2.358	2.107	22.364	2.813	3.622	1.379	3.924	WEB family protein At3g02930, chloroplastic	GI-H
MELO3C021851.2	0.265	2.397	4.023	1.484	1.353	NA	0.853	cytochrome P450 89A2-like	GI-H
MELO3C021823.2	1.244	0.947	3.548	1.990	0.474	12.406	0.765	Pleiotropic drug resistance ABC transporter	GI-H
MELO3C024728.2	1.597	1.017	5.707	1.174	2.644	0.658	2.273	ATPase family AAA domain-containing protein 3	GI-H
MELO3C024733.2	0.931	1.052	2.542	0.286	0.806	0.443	0.398	Pentatricopeptide repeat-containing protein	GI-H
MELO3C024738.2	0.223	NA	3.828	0.496	0.116	0.343	0.067	lactation elevated protein 1	GI-H
MELO3C024743.2	0.267	0.161	5.067	NA	0.471	0.160	0.173	disease resistance protein RGA2-like	GI-H
MELO3C024747.2	0.669	0.630	4.489	0.627	0.687	0.173	0.674	30S ribosomal protein S5, putative	GI-H
MELO3C024749.2	1.482	1.597	5.530	0.753	2.000	1.384	1.411	Pentatricopeptide repeat-containing family protein	GI-H
MELO3C024750.2	1.676	2.843	18.519	1.488	4.875	0.583	2.779	binding partner of ACD11 1-like	GI-H
MELO3C024760.2	2.560	3.544	17.013	1.945	2.973	3.168	2.320	RNA polymerase sigma factor sigA	GI-H
MELO3C024775.2	1.803	1.530	5.387	1.088	1.496	1.366	1.638	Bud13	GI-H
MELO3C024783.2	1.454	1.972	6.441	1.002	2.985	1.902	2.000	1-aminocyclopropane-1-carboxylate oxidase	GI-H
MELO3C024792.2	0.214	0.521	10.543	0.485	1.333	NA	0.688	RP/EB family microtubule-associated protein	GI-H

Gene ID	FPKM							Gene Description	Specific in episode
	FS	GI-M	GM-M	AN-M	GI-H	GM-H	AN-H		
MELO3C019213.2	13.978	14.132	36.263	14.424	13.600	14.285	12.641	mitochondrial import receptor subunit TOM7-1-like	GI-H
MELO3C019256.2	2.475	3.018	6.112	1.068	2.446	10.638	2.629	Glutamate receptor	GI-H
MELO3C019257.2	0.294	0.213	2.777	NA	0.552	NA	0.416	CDT1-like protein a, chloroplastic	GI-H
MELO3C019269.2	1.229	1.636	4.422	0.842	2.108	1.120	1.723	NEDD8-activating enzyme E1 regulatory subunit	GI-H
MELO3C019270.2	0.123	0.563	2.591	0.604	0.999	0.424	0.651	Hexosyltransferase	GI-H
MELO3C019281.2	0.613	0.433	3.439	NA	0.708	0.265	0.852	DNA repair protein RAD16	GI-H
MELO3C019290.2	0.264	1.184	1.463	9.616	0.461	31.288	0.418	mitogen-activated protein kinase kinase kinase 2-like	GI-H
MELO3C019300.2	0.700	0.446	6.457	NA	1.318	NA	1.030	Condensin complex subunit 2	GI-H
MELO3C034973.2	0.548	0.403	9.361	NA	0.674	0.441	0.964	RING-H2 finger protein ATL29-like	GI-H
MELO3C034980.2	0.468	0.610	1.272	0.650	0.176	0.385	0.376	Unknown protein	GI-H
MELO3C025991.2	0.487	1.177	2.890	1.569	0.649	1.784	1.066	heterogeneous nuclear ribonucleoprotein 1	GI-H
MELO3C025982.2	5.098	6.162	13.417	3.073	6.219	3.588	5.422	histidine kinase 2	GI-H
MELO3C025972.2	0.977	2.518	5.987	1.585	1.917	3.528	2.272	vacuolar protein-sorting-associated protein 11 homolog	GI-H
MELO3C025970.2	0.358	0.483	2.259	0.375	0.346	0.468	0.255	Pentatricopeptide repeat-containing protein	GI-H
MELO3C025953.2	1.278	1.115	3.709	0.918	0.617	1.383	0.814	Protein TIC 40, chloroplastic	GI-H
MELO3C025943.2	2.987	7.722	18.481	5.071	7.124	13.216	5.478	RPM1-interacting protein 4	GI-H
MELO3C035005.2	1.458	1.180	4.956	1.392	2.401	0.546	2.156	Polynucleotidyl transferase, ribonuclease H-like superfamily protein	GI-H
MELO3C013466.2	2.774	1.197	10.602	0.774	4.352	NA	3.737	Pesticidal crystal cry8Ba protein	GI-H
MELO3C013479.2	4.082	4.469	10.269	2.677	3.029	5.977	4.249	protein FAR1-RELATED SEQUENCE 9	GI-H
MELO3C013519.2	0.362	0.802	1.956	0.681	0.508	0.570	0.131	O-fucosyltransferase family protein	GI-H
MELO3C013535.2	0.507	NA	9.902	NA	0.408	NA	1.147	At4g28230	GI-H
MELO3C013558.2	0.166	0.591	1.941	0.760	0.231	2.558	0.290	Indole-3-acetic acid-amido synthetase GH3.3	GI-H
MELO3C034779.2	0.493	0.223	1.706	0.324	0.436	0.235	0.322	Transposon protein, putative, CACTA, En/Spm sub-class	GI-H
MELO3C013580.2	4.484	8.114	44.224	6.027	8.178	6.184	6.576	multiprotein-bridging factor 1b	GI-H
MELO3C013581.2	9.515	12.078	32.928	11.570	11.992	10.249	10.616	shaggy-related protein kinase eta	GI-H
MELO3C034788.2	0.370	0.323	2.741	NA	0.521	0.316	0.375	RING zinc finger protein	GI-H
MELO3C035057.2	1.288	2.439	6.351	1.751	1.792	0.905	1.692	Beta-glucosidase	GI-H
MELO3C013605.2	1.076	1.233	3.029	0.593	0.696	0.637	1.417	Clathrin light chain	GI-H
MELO3C013608.2	0.099	0.260	1.843	NA	0.359	0.703	0.273	tetraspanin-19	GI-H
MELO3C013609.2	0.897	1.364	5.384	1.192	1.124	NA	0.858	Acyl-CoA N-acyltransferase	GI-H

Gene ID	FPKM							Gene Description	Specific in episode
	FS	GI-M	GM-M	AN-M	GI-H	GM-H	AN-H		
MELO3C013680.2	1.142	1.234	8.298	0.693	1.251	0.766	1.346	DNA-binding protein, putative	GI-H
MELO3C013684.2	60.553	75.635	223.873	56.636	107.941	81.158	96.329	histone H2B	GI-H
MELO3C013687.2	4.873	2.764	17.624	3.487	5.974	NA	5.439	Histone H4	GI-H
MELO3C034814.2	8.603	9.144	45.612	13.204	9.580	8.610	12.206	Histone H4	GI-H
MELO3C013692.2	0.917	1.703	4.128	1.084	0.883	NA	0.625	Transmembrane protein, putative	GI-H
MELO3C013693.2	0.189	0.748	2.049	NA	0.469	NA	0.304	dof zinc finger protein DOF3.6	GI-H
MELO3C035087.2	0.592	0.574	4.328	NA	0.851	0.585	0.832	S-acyltransferase	GI-H
MELO3C025918.2	0.548	0.433	2.789	0.365	0.459	1.088	0.793	Phosphatidylinositol N-acetyglucosaminlytransferase subunit P-related	GI-H
MELO3C025905.2	0.779	0.774	2.504	0.282	0.599	0.237	0.548	telomere repeat-binding protein 5-like	GI-H
MELO3C025899.2	1.961	2.546	5.418	2.266	2.550	5.013	2.475	ATP-dependent zinc metalloprotease FTSH protein	GI-H
MELO3C025898.2	1.045	1.937	2.808	0.654	0.921	0.535	0.837	Protein DETOXIFICATION	GI-H
MELO3C025893.2	0.180	0.235	3.287	0.641	0.458	NA	0.192	chaperone protein DnaJ	GI-H
MELO3C035097.2	0.369	0.659	1.649	NA	0.588	NA	0.248	Pentatricopeptide repeat-containing protein	GI-H
MELO3C025886.2	0.823	1.369	6.355	1.283	1.877	1.471	1.427	AT5g59210/mnc17_100	GI-H
MELO3C025881.2	2.829	3.736	7.854	3.233	3.839	2.269	3.312	28S ribosomal S34 protein	GI-H
MELO3C025857.2	0.223	0.506	2.378	0.462	0.274	0.147	0.225	villin-1	GI-H
MELO3C025848.2	7.667	10.391	24.974	26.863	11.452	8.582	10.743	Peptidyl-prolyl cis-trans isomerase	GI-H
MELO3C035107.2	0.477	0.301	1.736	0.108	0.136	0.048	0.118	Zinc finger, RING-type	GI-H
MELO3C026841.2	0.215	0.676	1.030	0.878	0.415	2.147	0.400	UDP-glucose 4-epimerase family protein	GI-H
MELO3C026873.2	0.804	3.196	2.612	1.979	0.854	10.547	0.589	Non-specific serine/threonine protein kinase	GI-H
MELO3C026875.2	2.276	4.162	7.692	0.957	2.207	6.965	1.914	Kinase, putative	GI-H
MELO3C026878.2	1.322	1.566	6.349	0.572	1.597	0.830	1.905	Telomeric repeat binding protein, putative	GI-H
MELO3C019761.2	0.189	0.230	4.253	0.508	0.277	0.410	0.092	At2g31130/T16B12.6	GI-H
MELO3C019760.2	0.239	0.710	5.596	0.803	0.836	0.439	0.481	Adaptin ear-binding coat-associated protein 1	GI-H
MELO3C019743.2	1.957	2.117	7.925	1.685	2.328	1.962	2.813	DNA ligase 1	GI-H
MELO3C019735.2	0.759	5.946	28.114	10.558	0.843	20.417	0.933	1-aminocyclopropane-1-carboxylate oxidase	GI-H
MELO3C019734.2	0.717	0.718	3.998	0.807	1.106	0.645	1.294	Tyrosine-protein phosphatase	GI-H
MELO3C035120.2	1.593	1.702	7.665	1.445	2.579	2.360	2.009	E3 ubiquitin-protein ligase COP1	GI-H
MELO3C019724.2	0.476	0.699	1.828	0.983	0.381	0.501	0.538	Triacylglycerol lipase 2, putative	GI-H
MELO3C019717.2	0.936	2.630	7.451	1.180	1.240	4.879	1.453	Ubiquitin and WLM domain-containing metalloprotease	GI-H

Gene ID	FPKM							Gene Description	Specific in episode
	FS	GI-M	GM-M	AN-M	GI-H	GM-H	AN-H		
MELO3C019712.2	0.392	0.713	3.792	0.589	0.291	1.102	0.516	transcription elongation factor B polypeptide 3 isoform X1	GI-H
MELO3C019702.2	0.834	0.870	2.654	0.577	0.869	0.786	0.997	Serine/threonine-protein phosphatase	GI-H
MELO3C019696.2	0.273	0.976	2.458	1.642	0.878	2.432	0.600	HVA22-like protein	GI-H
MELO3C019688.2	0.510	0.318	2.459	0.407	0.198	0.192	0.253	Peroxisomal and mitochondrial division factor 2	GI-H
MELO3C019687.2	7.901	11.966	26.854	11.853	11.338	14.604	8.596	Mitogen-activated protein kinase	GI-H
MELO3C019678.2	2.249	2.290	7.148	1.656	2.506	1.288	2.824	Receptor-like protein kinase	GI-H
MELO3C019676.2	0.940	0.978	2.745	1.207	1.149	0.829	1.093	Tobamovirus multiplication protein 1	GI-H
MELO3C019673.2	0.405	0.661	2.762	NA	0.233	0.076	0.394	BOI-related E3 ubiquitin-protein ligase 1-like	GI-H
MELO3C019653.2	0.836	0.325	5.042	NA	1.336	0.383	1.070	PH-response transcription factor pacC/RIM101 isoform 2	GI-H
MELO3C019652.2	0.531	0.651	2.268	0.299	0.932	0.364	0.801	Cyclin-T1-like protein	GI-H
MELO3C019642.2	2.050	4.709	22.890	4.218	5.609	4.218	4.640	Polyubiquitin	GI-H
MELO3C019630.2	1.172	0.814	4.108	0.912	1.105	0.762	1.218	ribosomal RNA large subunit methyltransferase I	GI-H
MELO3C019629.2	2.478	4.159	16.992	3.747	3.579	6.830	2.025	ALA-interacting subunit 3-like	GI-H
MELO3C019611.2	2.373	4.465	24.075	6.157	3.726	6.889	1.996	DNA-3-methyladenine glycosylase, putative	GI-H
MELO3C019605.2	0.503	0.493	1.739	0.252	0.759	0.355	0.376	Pentatricopeptide repeat-containing family protein	GI-H
MELO3C025691.2	0.614	0.585	1.485	0.210	0.386	0.123	0.502	Pentatricopeptide repeat-containing protein, putative	GI-H
MELO3C025708.2	0.833	0.737	3.036	0.858	0.989	0.377	0.661	Ribonuclease P subunit p30	GI-H
MELO3C025712.2	0.225	1.353	2.570	0.337	0.275	0.167	0.389	Respiratory burst oxidase-like protein	GI-H
MELO3C025723.2	0.538	0.255	5.472	0.302	1.266	NA	0.807	Retrovirus-related Pol polyprotein from transposon TNT 1-94	GI-H
MELO3C025740.2	2.875	3.161	12.267	1.032	4.922	0.278	3.812	Interactor of constitutive active ROPs 3	GI-H
MELO3C025752.2	0.612	1.555	4.863	1.123	1.136	1.170	1.190	protein disulfide-isomerase 5-1	GI-H
MELO3C025768.2	1.560	1.982	4.117	3.337	1.363	5.733	1.345	Kelch repeat-containing F-box family protein	GI-H
MELO3C025779.2	0.639	0.982	2.334	2.610	0.697	1.815	0.651	Nucleotide/sugar transporter family protein	GI-H
MELO3C026904.2	1.180	2.088	4.220	0.243	1.940	0.624	1.575	protein N-lysine methyltransferase METTL21A	GI-H
MELO3C034922.2	87.046	78.019	336.675	93.304	65.086	65.147	86.937	Cytochrome f	GI-H
MELO3C001821.2	116.443	81.062	611.438	134.033	109.017	77.606	132.252	Cytochrome f	GI-H
MELO3C021444.2	2.180	5.899	17.679	7.947	3.338	9.740	3.432	Cytochrome c oxidase, subunit VIIa	GI-H
MELO3C021443.2	0.646	0.744	4.509	1.253	0.423	0.686	0.439	Acytochrome-C oxidase/electron carrier protein	GI-H
MELO3C021442.2	1.449	2.737	7.722	1.372	1.797	2.588	2.487	Histone deacetylase	GI-H
MELO3C021434.2	1.237	1.091	4.268	0.514	1.957	0.497	1.356	DNA polymerase epsilon catalytic subunit A	GI-H
MELO3C021425.2	0.919	0.721	3.349	0.739	1.548	0.343	1.009	Alpha/beta-Hydrolases superfamily protein	GI-H

Gene ID	FPKM							Gene Description	Specific in episode
	FS	GI-M	GM-M	AN-M	GI-H	GM-H	AN-H		
MELO3C021413.2	5.804	7.226	18.785	7.997	7.308	16.523	6.806	katanin p60 ATPase-containing subunit A1	GI-H
MELO3C021404.2	2.556	2.578	9.682	8.092	3.614	10.480	4.468	Heavy metal-associated isoprenylated plant protein 21	GI-H
MELO3C021389.2	0.406	0.757	7.400	0.823	0.812	1.403	0.482	At5g18940	GI-H
MELO3C021385.2	1.644	1.392	7.952	NA	1.546	1.663	1.959	Phosphatase 2C family protein	GI-H
MELO3C021380.2	1.098	1.006	5.109	1.307	1.979	1.531	1.350	Prolyl 4-hydroxylase alpha subunit, putative	GI-H
MELO3C021379.2	2.663	5.830	10.078	0.557	4.191	0.527	2.867	Histidine-containing phosphotransfer protein, putative	GI-H
MELO3C021370.2	0.654	0.329	2.544	NA	0.450	0.116	0.559	PAX-interacting protein 1	GI-H
MELO3C021353.2	3.886	5.032	23.807	2.714	5.722	3.445	7.493	Chromatin remodeling factor, putative	GI-H
MELO3C021344.2	1.709	1.736	4.629	0.664	1.377	1.892	1.525	Arginine-glutamic acid dipeptide repeats	GI-H
MELO3C021333.2	1.190	2.631	9.400	1.633	0.954	0.960	1.258	Vesicle-associated membrane protein	GI-H
MELO3C021329.2	0.559	0.549	3.608	0.218	0.414	0.444	0.487	ATP-binding cassette sub-family C member 11	GI-H
MELO3C021325.2	0.894	0.610	2.767	NA	1.282	1.764	1.065	Chaperone protein dnaJ, putative	GI-H
MELO3C021317.2	0.196	0.155	9.175	NA	0.717	0.278	0.544	Dynein light chain	GI-H
MELO3C021296.2	3.013	3.692	9.743	2.100	3.325	0.969	4.418	pumilio homolog 24	GI-H
MELO3C021285.2	0.804	0.678	3.215	0.320	1.444	NA	1.274	E3 SUMO-protein ligase NSE2	GI-H
MELO3C021283.2	0.770	1.365	3.393	1.251	0.973	1.887	0.890	AMSH-like ubiquitin thioesterase 1	GI-H
MELO3C021280.2	2.059	2.517	9.826	1.206	2.208	2.206	2.638	Calmodulin-binding transcription activator	GI-H
MELO3C021278.2	13.351	7.842	131.752	16.422	10.015	5.913	17.607	60S ribosomal protein L29	GI-H
MELO3C021277.2	3.347	4.798	53.608	11.724	6.131	3.479	9.919	60S ribosomal protein L29	GI-H
MELO3C021275.2	2.618	2.513	8.058	1.261	2.270	2.312	2.058	Binding protein	GI-H
MELO3C021264.2	0.221	2.784	5.370	4.595	2.448	5.825	2.401	Ubiquitin-conjugating enzyme, E2	GI-H
MELO3C021257.2	1.366	0.929	4.829	1.024	1.680	0.628	1.146	Pentatricopeptide repeat-containing family protein	GI-H
MELO3C021256.2	1.263	24.213	31.509	1.061	7.996	1.556	0.836	Desiccation-related protein PCC13-62	GI-H
MELO3C021255.2	0.613	0.897	3.187	0.611	1.439	0.700	0.692	30S ribosomal protein S6	GI-H
MELO3C021254.2	2.051	2.274	5.265	1.437	1.901	1.984	2.319	DnaJ homolog subfamily C member 14	GI-H
MELO3C021251.2	0.386	0.829	3.944	0.356	1.004	0.900	0.662	nuclear export mediator factor Nemf	GI-H
MELO3C021248.2	1.458	1.566	7.375	1.224	1.566	0.627	0.967	At5g30145	GI-H
MELO3C021243.2	0.976	1.558	4.602	0.996	1.949	0.401	1.517	DNA (Cytosine-5)-methyltransferase DRM2	GI-H
MELO3C021242.2	6.369	6.525	13.302	4.481	4.795	16.473	3.872	YTH domain family protein 2	GI-H
MELO3C021238.2	0.752	0.223	5.269	0.098	0.967	NA	0.887	phragmoplast orienting kinesin-1 isoform X1	GI-H
MELO3C021232.2	0.453	0.546	5.619	0.826	0.599	0.583	0.665	Novel plant snare, putative	GI-H

Gene ID	FPKM							Gene Description	Specific in episode
	FS	GI-M	GM-M	AN-M	GI-H	GM-H	AN-H		
MELO3C021231.2	0.329	0.367	1.529	0.645	0.116	0.389	0.173	Non-specific serine/threonine protein kinase	GI-H
MELO3C021217.2	0.688	1.037	3.787	0.953	0.671	0.580	0.923	MACPF domain-containing CAD1-like protein	GI-H
MELO3C021204.2	0.219	0.274	1.967	NA	0.329	0.046	0.383	Origin recognition complex subunit 5	GI-H
MELO3C021203.2	1.285	1.410	5.161	0.868	1.684	1.293	1.334	ATP-dependent RNA helicase SUPV3L1, mitochondrial	GI-H
MELO3C021200.2	0.247	0.245	1.537	0.128	0.206	0.058	0.194	AT-rich interactive domain protein	GI-H
MELO3C021188.2	1.546	1.510	5.564	1.450	1.150	2.554	1.811	Receptor-like protein kinase HERK 1	GI-H
MELO3C021187.2	0.492	0.975	3.195	0.894	1.098	0.761	0.550	Mitogen-activated protein kinase	GI-H
MELO3C021186.2	10.606	19.397	30.178	9.568	10.987	17.378	8.855	Protein BPS1, chloroplastic-like protein	GI-H
MELO3C021183.2	0.340	NA	6.691	NA	0.264	1.466	0.245	FK506-binding protein 4-like	GI-H
MELO3C021177.2	0.475	0.599	2.432	0.518	0.985	0.841	0.689	Golgin candidate 3	GI-H
MELO3C021153.2	0.891	0.476	4.942	0.412	1.305	0.079	0.919	Arginine--tRNA ligase	GI-H
MELO3C021149.2	2.889	4.670	17.002	4.212	4.154	3.255	3.223	vesicle-associated protein 1-3	GI-H
MELO3C021148.2	2.170	4.721	7.144	1.906	1.944	3.245	2.811	Autophagy-related protein	GI-H
MELO3C022263.2	0.551	0.958	9.083	1.995	1.120	1.655	0.829	Fiber protein fb11	GI-H
MELO3C022268.2	0.300	0.142	2.688	0.541	0.614	NA	0.654	Hydroxyproline-rich glycoprotein family protein	GI-H
MELO3C022270.2	2.239	2.138	7.806	1.496	2.810	0.642	2.317	Nucleolar protein 6	GI-H
MELO3C022272.2	1.126	1.245	3.257	0.570	1.325	0.583	0.821	Vesicle transport protein SEC20	GI-H
MELO3C022274.2	1.704	1.474	5.050	1.609	2.029	1.214	1.701	COP9 signalosome complex subunit 7	GI-H
MELO3C022277.2	1.122	1.725	4.825	1.439	1.465	1.204	1.581	Leucine carboxyl methyltransferase, putative	GI-H
MELO3C022302.2	2.361	1.999	7.842	1.008	1.599	6.982	1.922	protein LHY-like isoform X1	GI-H
MELO3C022308.2	1.569	1.855	3.759	1.423	1.675	4.752	1.276	Beclin 1 protein	GI-H
MELO3C022309.2	1.635	2.726	6.873	5.496	2.062	7.014	2.152	Ectonucleotide pyrophosphatase/phosphodiesterase family member 3	GI-H
MELO3C022310.2	7.496	11.456	15.683	4.413	5.289	20.429	5.666	Histidine kinase	GI-H
MELO3C022316.2	0.826	3.724	12.006	4.948	2.207	8.857	2.913	MADS-box transcription factor	GI-H
MELO3C022321.2	2.561	3.678	6.414	3.555	1.687	9.848	1.326	sphingosine-1-phosphate lyase	GI-H
MELO3C022342.2	4.191	9.110	12.003	7.553	2.349	19.653	4.631	NAC domain-containing protein 83	GI-H
MELO3C035240.2	0.324	0.334	1.562	0.241	0.384	NA	0.363	DNA-directed RNA polymerase subunit beta	GI-H
MELO3C022370.2	0.999	1.045	11.667	0.838	0.983	0.233	1.731	U2 small nuclear ribonucleoprotein a	GI-H
MELO3C022384.2	0.489	0.621	3.200	NA	0.568	NA	0.736	Protein JASON	GI-H
MELO3C022387.2	1.736	0.726	5.855	0.655	2.078	0.169	2.068	Histone-lysine N-methyltransferase	GI-H

Gene ID	FPKM							Gene Description	Specific in episode
	FS	GI-M	GM-M	AN-M	GI-H	GM-H	AN-H		
MELO3C022396.2	0.497	1.244	5.460	0.622	1.596	1.138	1.646	nitric oxide synthase-interacting protein-like	GI-H
MELO3C022403.2	2.540	2.579	6.888	2.237	3.103	3.287	2.951	Myosin	GI-H
MELO3C022408.2	1.008	1.442	4.242	1.509	1.410	4.980	0.818	UV-stimulated scaffold protein A homolog	GI-H
MELO3C022427.2	0.527	0.992	2.638	1.349	0.757	0.473	1.046	Trigger factor	GI-H
MELO3C022429.2	3.020	8.515	8.077	0.616	2.038	4.733	0.819	Amaranthin-like lectin	GI-H
MELO3C022430.2	2.340	7.974	8.508	1.155	2.785	2.993	2.310	Amaranthin-like lectin	GI-H
MELO3C022433.2	1.888	2.957	16.970	3.286	1.394	NA	2.611	Dirigent protein	GI-H
MELO3C022441.2	2.133	1.441	15.214	4.918	2.559	0.594	3.480	60S ribosomal protein L38	GI-H
MELO3C022483.2	0.704	0.609	3.890	0.356	0.724	0.109	0.553	RNA polymerase sigma factor	GI-H
MELO3C022484.2	1.035	1.562	5.731	1.224	1.021	0.376	0.911	chromo domain-containing protein LHP1-like	GI-H
MELO3C022500.2	0.844	1.215	2.245	1.207	1.100	0.741	1.108	Vacuolar protein sorting 26	GI-H
MELO3C022501.2	0.274	0.418	3.195	NA	0.407	0.186	0.293	SNARE-interacting protein KEULE	GI-H
MELO3C022502.2	3.462	2.932	9.601	1.025	3.467	0.446	2.296	Peptidylprolyl isomerase	GI-H
MELO3C022508.2	0.837	1.749	4.125	0.558	1.307	0.896	0.854	Coiled-coil protein	GI-H
MELO3C022510.2	0.260	0.205	1.834	0.205	0.298	0.094	0.465	Iron import ATP-binding/permease IrtA	GI-H
MELO3C022516.2	2.046	3.421	10.763	3.061	1.825	1.854	0.850	MADS-box transcription factor	GI-H
MELO3C022524.2	0.432	1.084	2.457	1.134	0.933	0.576	0.738	SPX domain-containing membrane protein	GI-H
MELO3C022534.2	0.225	0.655	1.652	0.520	0.575	0.364	0.426	Transmembrane protein, putative	GI-H
MELO3C020474.2	0.806	0.898	2.281	0.705	0.821	0.874	0.757	BSD transcription factor	GI-H
MELO3C020478.2	0.531	0.503	1.198	0.301	0.337	0.328	0.395	LRR receptor-like kinase	GI-H
MELO3C020490.2	3.667	3.424	15.251	2.798	2.962	0.842	3.211	U2 small nuclear ribonucleoprotein B'' 2-like	GI-H
MELO3C020494.2	1.559	1.540	3.741	1.132	1.675	1.576	1.824	E3 ubiquitin-protein ligase listerin	GI-H
MELO3C020498.2	0.299	0.213	1.006	0.211	0.299	0.195	0.221	tRNA pseudouridine synthase	GI-H
MELO3C020503.2	0.451	1.183	3.894	1.140	1.350	2.065	1.579	Structure-specific endonuclease subunit SLX1 homolog	GI-H
MELO3C020530.2	2.323	4.239	12.896	3.567	4.191	1.123	3.497	Protein CHUP1, chloroplastic	GI-H
MELO3C020534.2	0.752	1.286	6.623	1.480	0.874	1.229	1.556	Ran-binding protein 1	GI-H
MELO3C020546.2	0.310	2.116	2.753	1.575	1.162	2.187	0.822	Phosphoinositide phospholipase C	GI-H
MELO3C035260.2	128.422	116.711	462.071	151.353	145.805	94.899	159.589	Ribosomal protein L5	GI-H
MELO3C020570.2	1.394	5.780	14.041	3.168	3.008	2.680	2.408	Maternal effect embryo arrest protein	GI-H
MELO3C020577.2	0.718	1.714	2.558	0.961	0.763	1.145	0.959	Homeobox prospero	GI-H

Gene ID	FPKM							Gene Description	Specific in episode
	FS	GI-M	GM-M	AN-M	GI-H	GM-H	AN-H		
MELO3C020586.2	0.569	0.629	11.451	1.663	0.718	0.413	0.904	Mitochondrial import inner membrane translocase subunit Tim10	GI-H
MELO3C035274.2	3.565	4.531	27.628	2.297	3.067	10.204	1.350	17.5 kDa class I heat shock protein	GI-H
MELO3C020599.2	2.622	4.499	13.729	5.958	5.041	3.146	5.563	transcription elongation factor B polypeptide 1	GI-H
MELO3C020637.2	3.960	5.797	11.046	3.387	4.386	4.299	4.018	Outer envelope pore protein 16, chloroplastic	GI-H
MELO3C020662.2	0.657	0.585	1.683	0.482	0.826	0.479	0.622	nodulin homeobox isoform X1	GI-H
MELO3C020668.2	1.369	0.935	10.447	1.247	2.704	0.111	2.508	3-ketoacyl-CoA synthase	GI-H
MELO3C020670.2	0.900	0.949	3.150	2.462	0.514	0.836	0.405	3-ketoacyl-CoA synthase	GI-H
MELO3C020686.2	0.313	0.242	1.561	NA	0.208	0.738	0.217	iron-sulfur assembly protein IscA-like 2, mitochondrial	GI-H
MELO3C020688.2	1.770	1.562	10.569	1.297	2.627	0.304	2.051	Kinesin-related protein	GI-H
MELO3C020704.2	12.928	21.589	76.569	20.776	16.862	7.266	15.740	40S ribosomal protein S15A	GI-H
MELO3C026848.2	2.086	1.224	5.999	1.568	1.830	0.793	2.499	Mitogen-activated protein kinase	GI-H
MELO3C026849.2	0.323	0.434	1.798	0.438	0.583	0.182	0.338	Hydrolase family protein / HAD-superfamily protein	GI-H
MELO3C005046.2	0.527	0.855	4.147	0.293	0.806	0.927	1.296	protein indeterminate-domain 2	GI-H
MELO3C005030.2	0.787	0.781	1.925	0.736	0.869	0.706	0.882	RNA ligase, putative	GI-H
MELO3C005015.2	0.981	1.052	3.187	0.861	1.050	1.073	1.225	Ubiquitin carboxyl-terminal hydrolase, putative	GI-H
MELO3C005006.2	0.401	0.529	1.525	0.437	0.505	0.423	0.328	Photosystem II reaction center PsbP family protein	GI-H
MELO3C005002.2	1.699	1.406	5.844	0.500	0.785	NA	0.879	Interactor of constitutive active ROPs-like protein	GI-H
MELO3C004999.2	3.518	2.597	9.539	2.565	3.783	4.502	2.574	nicotinamide adenine dinucleotide transporter 1, chloroplastic-like	GI-H
MELO3C004995.2	1.425	1.670	5.083	1.522	2.078	0.587	1.861	50S ribosomal protein L9, chloroplastic	GI-H
MELO3C004994.2	0.324	0.377	2.844	0.291	0.685	NA	0.199	Actin protein 2/3 complex subunit-like protein	GI-H
MELO3C035453.2	0.914	0.846	4.008	0.569	1.822	0.354	1.047	Pentatricopeptide repeat-containing family protein	GI-H
MELO3C004967.2	1.149	1.325	4.532	1.054	1.825	0.540	1.962	ABC transporter G family member 3	GI-H
MELO3C004946.2	2.251	6.272	8.381	11.795	3.242	5.932	2.705	serine carboxypeptidase-like	GI-H
MELO3C004945.2	1.148	1.715	4.373	2.477	1.780	2.800	1.415	vacuolar protein sorting-associated protein 24 homolog 1	GI-H
MELO3C004936.2	0.941	0.714	4.374	1.220	1.002	1.494	1.124	P-loop NTPase domain-containing protein LPA1 homolog 1	GI-H
MELO3C004927.2	0.931	0.896	4.015	NA	1.767	0.399	1.794	homeobox protein BEL1 homolog isoform X1	GI-H
MELO3C004917.2	1.468	2.999	5.705	2.184	1.959	2.448	1.714	sulfite oxidase-like	GI-H
MELO3C004905.2	0.226	0.331	2.318	0.237	0.345	0.219	0.287	helicase protein MOM1	GI-H
MELO3C004898.2	0.352	0.977	1.708	0.720	0.502	0.471	0.510	Glycosyl transferase, family 2	GI-H
MELO3C004892.2	1.344	3.360	7.307	1.162	0.784	2.791	0.636	Unknown protein	GI-H

Gene ID	FPKM							Gene Description	Specific in episode
	FS	GI-M	GM-M	AN-M	GI-H	GM-H	AN-H		
MELO3C035524.2	0.204	0.566	2.423	0.602	0.534	0.286	0.438	Dynamin	GI-H
MELO3C035546.2	0.285	0.855	3.122	NA	0.475	NA	0.359	pheophorbide a oxygenase, chloroplastic	GI-H
MELO3C004853.2	22.219	11.698	218.485	20.343	11.686	6.652	9.700	30S ribosomal protein S3, chloroplastic	GI-H
MELO3C004833.2	1.113	0.680	2.727	0.652	0.597	0.710	1.078	crooked neck-like protein 1	GI-H
MELO3C004816.2	0.503	0.507	2.560	NA	1.060	0.149	0.670	Pentatricopeptide repeat-containing protein	GI-H
MELO3C004786.2	0.750	0.566	3.512	NA	0.420	NA	1.307	Wall-associated receptor kinase-like 20	GI-H
MELO3C004759.2	0.512	1.367	7.327	0.513	1.413	NA	1.200	protein BUD31 homolog 2	GI-H
MELO3C004729.2	1.233	1.592	4.506	1.179	1.460	1.710	1.254	Plant/F27B13-30 protein	GI-H
MELO3C004713.2	7.813	14.247	54.949	12.709	22.070	3.119	15.369	Ribosomal protein	GI-H
MELO3C035414.2	0.760	0.434	4.316	1.523	1.136	0.171	0.904	RING/FYVE/PHD zinc finger superfamily protein	GI-H
MELO3C004699.2	0.995	1.130	3.384	2.136	1.140	1.116	1.113	Protein ROOT PRIMORDIUM DEFECTIVE 1	GI-H
MELO3C004698.2	5.910	6.424	20.389	9.508	6.197	7.185	6.239	F1F0-ATPase inhibitor protein	GI-H
MELO3C035594.2	0.160	0.313	1.070	NA	0.197	0.408	0.237	lactation elevated protein 1	GI-H
MELO3C004669.2	1.081	1.265	4.172	0.529	1.151	0.416	1.302	Pentatricopeptide repeat-containing protein	GI-H
MELO3C025590.2	0.445	0.781	3.087	0.815	0.597	1.265	0.499	NF-kappa-B inhibitor-like protein 2 isoform 2	GI-H
MELO3C025588.2	0.732	0.699	7.475	0.805	1.220	0.223	0.647	Protein phosphatase 1 regulatory subunit 7	GI-H
MELO3C025579.2	0.434	0.225	2.476	NA	0.181	0.157	0.285	Unknown protein	GI-H
MELO3C025562.2	2.468	3.310	13.443	1.678	5.146	3.279	2.923	U4/U6.U5 tri-snRNP-associated protein 2-like	GI-H
MELO3C025555.2	1.071	1.232	6.597	2.267	0.824	1.272	1.406	Mo25 family protein	GI-H
MELO3C025549.2	1.183	1.794	4.357	2.272	2.071	2.215	1.947	vesicle-fusing ATPase	GI-H
MELO3C025545.2	1.029	0.980	3.300	0.855	1.170	1.367	0.907	Pentatricopeptide repeat-containing protein	GI-H
MELO3C025541.2	0.600	2.304	4.255	4.484	1.147	1.632	0.685	Thymidine kinase	GI-H
MELO3C026103.2	2.295	2.820	83.253	5.582	2.295	NA	1.384	DUF538 family protein	GI-H
MELO3C026097.2	0.601	0.628	4.719	0.929	1.162	0.268	0.592	Hexosyltransferase	GI-H
MELO3C035628.2	0.304	0.346	1.761	0.218	0.401	NA	0.356	Unknown protein	GI-H
MELO3C026075.2	1.742	1.836	6.942	2.293	3.222	0.332	3.215	Pentatricopeptide repeat-containing protein, chloroplastic	GI-H
MELO3C026068.2	0.501	0.537	5.504	1.309	0.817	0.409	1.036	Transcriptional adapter ADA2b-like protein	GI-H
MELO3C026067.2	0.352	0.338	2.819	0.253	0.314	NA	0.311	tRNA (Guanine(9)-N1)-methyltransferase-like	GI-H
MELO3C021698.2	1.932	1.726	6.698	1.899	2.942	1.979	2.078	Mitochondrial pyruvate carrier	GI-H
MELO3C021699.2	0.706	0.453	1.578	0.551	0.588	0.496	0.519	Golgin family A protein	GI-H
MELO3C035649.2	0.087	0.236	1.743	0.195	0.298	0.163	0.327	K(+) efflux antiporter	GI-H

Gene ID	FPKM							Gene Description	Specific in episode
	FS	GI-M	GM-M	AN-M	GI-H	GM-H	AN-H		
MELO3C021715.2	1.322	1.254	3.954	1.377	1.015	1.410	1.677	E3 ubiquitin-protein ligase COP1-like	GI-H
MELO3C021729.2	0.802	1.474	3.762	1.315	1.217	2.441	1.151	Malonyl-CoA decarboxylase, mitochondrial	GI-H
MELO3C021732.2	0.943	0.866	2.802	0.680	0.583	0.518	1.098	TATA box-binding protein associated factor RNA polymerase I subunit C	GI-H
MELO3C021742.2	2.461	9.543	10.387	7.425	4.881	3.745	3.610	Glycine cleavage system H, mitochondrial	GI-H
MELO3C021744.2	0.216	0.221	1.153	NA	0.155	NA	0.292	Pentatricopeptide repeat-containing protein	GI-H
MELO3C021747.2	0.151	0.543	1.617	0.306	0.492	0.106	0.385	DCD (Development and Cell Death) domain protein	GI-H
MELO3C021757.2	3.523	10.549	10.209	5.439	4.792	9.784	3.809	Glutathione S-transferase family protein	GI-H
MELO3C021758.2	0.298	0.320	2.792	NA	0.445	1.369	0.450	DNA-directed RNA polymerases II and IV subunit 5A	GI-H
MELO3C021769.2	0.731	0.850	4.203	0.562	1.637	2.811	0.886	Ribonuclease H2, subunit C	GI-H
MELO3C021777.2	3.656	4.957	9.480	4.518	4.319	4.603	3.764	Vesicle-associated membrane protein, putative	GI-H
MELO3C021786.2	1.047	0.835	2.213	0.555	0.826	0.766	0.864	Cyclin-T1-like protein	GI-H
MELO3C021793.2	0.481	0.917	4.293	0.994	2.060	0.301	1.934	RING-type E3 ubiquitin transferase	GI-H
MELO3C021803.2	6.791	16.607	27.282	11.874	12.141	17.366	7.326	ras-related protein RABF2b	GI-H
MELO3C021804.2	0.511	0.405	1.402	0.139	0.312	0.138	0.235	snRNA-activating protein complex subunit, putative	GI-H
MELO3C021807.2	0.352	0.576	1.724	0.308	0.779	0.610	0.520	Nucleolin	GI-H
MELO3C021810.2	2.542	3.989	8.390	4.974	3.276	6.196	3.703	gamma-soluble NSF attachment protein	GI-H
MELO3C021813.2	0.774	0.833	1.850	0.651	0.437	0.740	0.532	UDP-glucose 4-epimerase, putative	GI-H
MELO3C021816.2	0.412	0.770	3.240	0.426	0.476	0.338	0.409	Pentatricopeptide repeat-containing protein chloroplastic	GI-H
MELO3C002863.2	2.492	2.035	63.057	0.670	7.099	NA	5.103	patellin-4	GI-H
MELO3C002837.2	0.749	1.045	2.900	0.458	0.849	0.730	1.083	Maternal effect embryo arrest 22	GI-H
MELO3C002831.2	0.312	0.207	2.024	NA	0.365	0.705	0.269	Pentatricopeptide repeat-containing protein	GI-H
MELO3C002823.2	0.280	0.058	2.353	0.111	0.463	NA	0.105	Protein SULFUR DEFICIENCY-INDUCED 1	GI-H
MELO3C002819.2	0.606	0.813	2.154	0.320	0.717	0.493	0.587	eukaryotic translation initiation factor 3 subunit A-like	GI-H
MELO3C002816.2	2.244	3.228	10.517	3.023	3.948	1.066	3.235	obg-like ATPase 1	GI-H
MELO3C002808.2	0.786	0.993	5.709	1.448	0.899	0.274	0.884	ADP-ribosylation factor GTPase-activating protein AGD12-like	GI-H
MELO3C002800.2	1.600	2.133	12.235	1.766	2.746	0.350	2.533	50S ribosomal protein L33-like	GI-H
MELO3C002796.2	2.762	2.726	8.918	1.603	3.310	0.779	4.405	BZIP domain class transcription factor (DUF630 and DUF632)	GI-H
MELO3C002789.2	0.489	0.563	2.270	0.117	0.323	0.215	0.184	BnaCnng71930D protein	GI-H
MELO3C002786.2	0.466	0.426	1.104	0.394	0.527	0.784	0.319	Ubiquitin fusion degradation 1	GI-H
MELO3C002766.2	0.387	0.915	4.781	0.821	0.740	0.797	0.631	Non-specific serine/threonine protein kinase	GI-H

Gene ID	FPKM							Gene Description	Specific in episode
	FS	GI-M	GM-M	AN-M	GI-H	GM-H	AN-H		
MELO3C002757.2	0.810	0.963	2.879	1.160	0.714	1.780	0.983	signal recognition particle 14 kDa protein	GI-H
MELO3C002738.2	1.105	1.413	7.952	1.840	1.762	1.349	1.628	cullin-4	GI-H
MELO3C002736.2	1.236	1.198	7.218	2.498	1.819	0.569	2.042	Calcyclin-binding protein	GI-H
MELO3C002733.2	1.104	1.211	3.593	1.109	1.070	1.899	1.172	Intron maturase, type II family protein	GI-H
MELO3C002729.2	0.520	0.460	1.563	0.391	0.374	1.270	0.671	Purine permease	GI-H
MELO3C002719.2	2.919	1.881	20.437	3.915	3.902	0.384	2.307	60S ribosomal protein L18a	GI-H
MELO3C002717.2	0.372	0.576	2.899	0.962	0.553	0.421	0.516	prefoldin subunit 6	GI-H
MELO3C002712.2	2.043	4.603	11.473	4.273	4.618	2.418	3.337	coatomer subunit zeta-1-like	GI-H
MELO3C002711.2	0.365	0.744	1.461	0.578	0.663	0.439	0.378	Mg2+ transporter protein, CorA-like/Zinc transport protein ZntB	GI-H
MELO3C002700.2	2.012	3.206	11.085	1.599	2.763	NA	2.498	Serine/threonine-protein kinase PLK4	GI-H
MELO3C002697.2	1.188	0.793	6.865	0.977	2.061	NA	1.703	exonuclease 1	GI-H
MELO3C002694.2	1.136	1.014	2.407	0.636	1.075	0.958	0.801	Trehalose-6-phosphate synthase, putative	GI-H
MELO3C002692.2	1.262	2.077	9.715	1.455	1.193	0.803	1.643	GDSL esterase/lipase	GI-H
MELO3C002686.2	0.579	1.299	5.801	0.719	1.931	0.737	1.303	Receptor-like protein kinase	GI-H
MELO3C002679.2	0.569	0.876	3.715	NA	0.390	0.759	0.264	heat stress transcription factor A-4c	GI-H
MELO3C002663.2	1.658	1.826	8.696	1.423	1.713	1.154	1.392	SAP30-binding protein	GI-H
MELO3C002659.2	1.390	2.352	4.541	2.348	1.900	6.432	1.695	Receptor protein kinase, putative	GI-H
MELO3C002639.2	0.913	0.625	3.279	0.725	0.336	0.259	0.519	Pentatricopeptide repeat-containing protein	GI-H
MELO3C002617.2	0.289	0.733	3.909	0.738	1.172	1.189	0.455	dof zinc finger protein DOF1.4-like	GI-H
MELO3C002614.2	1.418	1.736	2.857	1.863	0.835	2.035	1.032	Nuclear control of ATPase protein 2	GI-H
MELO3C002612.2	1.563	4.017	9.780	1.552	2.641	2.328	2.344	Protein SMG7	GI-H
MELO3C002608.2	0.719	0.795	13.004	0.371	1.282	NA	0.357	transcription factor bHLH118-like	GI-H
MELO3C002597.2	0.434	0.409	2.950	NA	1.005	NA	0.667	pentatricopeptide repeat-containing protein At2g20710, mitochondrial-like	GI-H
MELO3C002596.2	0.759	0.770	5.962	0.672	1.323	0.601	1.030	pentatricopeptide repeat-containing protein At2g20710, mitochondrial-like	GI-H
MELO3C002572.2	0.965	3.965	3.363	0.409	1.134	NA	1.610	Carboxypeptidase	GI-H
MELO3C002570.2	1.012	2.061	12.887	1.457	1.667	1.360	1.515	LisH and RanBPM domains containing protein	GI-H
MELO3C002564.2	1.093	1.244	5.801	1.611	1.631	5.700	1.351	Lysine ketoglutarate reductase trans-splicing-like protein	GI-H
MELO3C002555.2	0.547	0.802	1.324	1.602	0.518	5.108	0.563	external alternative NAD(P)H-ubiquinone oxidoreductase B2, mitochondrial	GI-H
MELO3C002542.2	0.426	0.847	2.132	0.345	0.146	0.321	0.263	Serine/threonine-protein kinase	GI-H

Gene ID	FPKM							Gene Description	Specific in episode
	FS	GI-M	GM-M	AN-M	GI-H	GM-H	AN-H		
MELO3C002509.2	5.971	3.245	29.361	0.772	8.743	NA	7.060	Kinesin-like protein	GI-H
MELO3C002482.2	1.300	1.290	3.864	1.870	0.905	1.796	0.890	Chaperone protein dnaJ 50	GI-H
MELO3C002478.2	2.476	3.446	17.927	2.302	3.836	4.894	5.255	Crib domain-containing protein ric10	GI-H
MELO3C002469.2	1.060	1.567	5.495	NA	1.492	NA	2.466	BnaC01g13120D protein	GI-H
MELO3C002466.2	1.400	0.446	4.864	0.329	1.782	0.154	1.964	Abnormal spindle-like microcephaly-associated-like protein, putative	GI-H
MELO3C002454.2	1.347	2.325	9.561	NA	2.809	NA	2.559	Glycosyl transferase, family 31	GI-H
MELO3C002417.2	0.224	0.498	2.609	NA	0.330	0.158	0.420	Ankyrin repeat family protein	GI-H
MELO3C002416.2	0.355	0.824	2.639	0.608	0.502	0.262	0.373	Ankyrin repeat family protein	GI-H
MELO3C002412.2	0.584	0.508	2.006	0.470	0.320	0.438	0.390	Ankyrin repeat family protein	GI-H
MELO3C002407.2	4.506	5.610	13.833	5.198	5.357	7.772	4.617	PB1 domain-containing protein/Pkinase_Tyr domain-containing protein	GI-H
MELO3C035735.2	0.091	0.123	1.917	NA	0.218	0.174	0.133	protein FAR-RED IMPAIRED RESPONSE 1 isoform X1	GI-H
MELO3C002405.2	4.310	3.931	14.022	4.282	6.969	2.025	3.925	50S ribosomal protein L7/L12	GI-H
MELO3C002383.2	3.345	3.000	14.897	NA	3.156	5.263	2.212	transcription factor bHLH137	GI-H
MELO3C002357.2	0.805	0.547	3.020	0.408	0.995	0.483	0.557	UPF0235 protein At5g63440	GI-H
MELO3C002356.2	3.161	3.549	9.116	3.367	3.538	5.045	3.857	AAA-type ATPase family protein	GI-H
MELO3C002341.2	0.503	3.348	4.784	1.206	0.565	NA	0.217	At5g50335	GI-H
MELO3C002333.2	0.581	1.037	4.469	NA	0.801	0.204	0.568	30S ribosomal protein S21	GI-H
MELO3C002328.2	4.345	5.352	14.987	3.784	5.101	4.819	5.214	zinc finger CCCH domain-containing protein 43	GI-H
MELO3C002327.2	2.568	2.403	8.290	1.640	3.665	0.504	3.466	lysine-specific demethylase REF6	GI-H
MELO3C002326.2	0.853	1.651	5.031	0.685	1.577	0.208	1.391	DNA-(Apurinic or apyrimidinic site) lyase	GI-H
MELO3C002306.2	0.259	NA	1.935	0.498	0.416	0.190	0.194	Manganese-dependent ADP-ribose/CDP-alcohol diphosphatase	GI-H
MELO3C002302.2	0.224	0.604	2.145	0.461	0.472	0.706	0.414	Kinase family protein	GI-H
MELO3C002296.2	0.468	0.543	2.124	NA	0.462	0.211	0.376	C2 and GRAM domain-containing protein	GI-H
MELO3C002295.2	0.512	0.766	3.926	NA	0.749	NA	1.098	(3R)-hydroxymyristoyl-[acyl-carrier-protein] dehydratase	GI-H
MELO3C002285.2	0.626	0.550	4.635	0.500	1.995	0.604	1.139	Carboxyl-terminal peptidase, putative (DUF239)	GI-H
MELO3C002284.2	0.386	0.513	2.661	0.965	0.386	0.485	0.268	Plant UBX domain-containing protein 10	GI-H
MELO3C002277.2	0.686	0.889	2.902	0.442	1.209	0.269	1.261	Glutathione transport system permease protein gsiD	GI-H
MELO3C002248.2	1.450	2.378	5.900	4.186	1.697	1.744	1.110	Ubiquitin-conjugating enzyme, E2	GI-H
MELO3C002234.2	0.582	0.568	2.879	0.244	0.976	0.257	0.869	anaphase-promoting complex subunit 8	GI-H
MELO3C002230.2	1.404	1.256	5.908	1.006	0.929	1.066	0.864	At1g74860	GI-H

Gene ID	FPKM							Gene Description	Specific in episode
	FS	GI-M	GM-M	AN-M	GI-H	GM-H	AN-H		
MELO3C002229.2	1.515	1.953	4.774	1.069	2.020	0.727	1.999	Pentatricopeptide repeat-containing protein At1g74850, chloroplastic	GI-H
MELO3C002222.2	0.440	0.424	8.446	0.373	0.251	0.311	0.191	AAA-ATPase At3g28580-like	GI-H
MELO3C002214.2	0.425	0.786	1.871	NA	0.573	0.273	0.358	dnaJ homolog subfamily B member 13	GI-H
MELO3C002186.2	0.351	0.690	3.822	0.852	0.647	1.396	0.348	Testis-expressed sequence 2 protein	GI-H
MELO3C002178.2	0.432	0.384	7.752	NA	1.175	NA	0.626	CDT1-like protein a, chloroplastic	GI-H
MELO3C002172.2	0.537	1.173	4.664	0.318	0.674	1.005	0.423	DNA-directed RNA polymerases I, II, and III subunit RPABC4	GI-H
MELO3C002168.2	0.589	0.609	6.687	0.677	2.114	0.170	1.020	DNA-binding protein RHL1 isoform X2	GI-H
MELO3C002164.2	0.384	0.549	1.785	0.933	0.670	0.627	0.470	Peptidoglycan-binding LysM domain protein, putative	GI-H
MELO3C002153.2	0.205	0.255	3.131	0.236	0.264	0.364	0.164	transcription repressor KAN1-like isoform X1	GI-H
MELO3C035756.2	0.239	0.411	1.315	NA	0.515	0.080	0.599	CAI-1 autoinducer sensor kinase/phosphatase cqsS isoform 2	GI-H
MELO3C002124.2	0.400	0.735	5.371	1.034	0.688	1.585	0.658	Mitogen-activated protein kinase	GI-H
MELO3C002118.2	2.302	3.772	19.707	4.241	4.879	3.573	3.755	Elongation factor 1 alpha	GI-H
MELO3C002106.2	1.917	0.945	18.546	0.690	3.443	NA	3.361	Kinesin-like protein	GI-H
MELO3C002099.2	2.387	4.883	13.954	3.729	4.683	5.589	3.631	Calmodulin binding protein-like, putative	GI-H
MELO3C002093.2	1.981	2.567	7.003	1.628	1.820	3.730	1.397	AMSH-like ubiquitin thioesterase 1	GI-H
MELO3C002088.2	1.302	1.432	2.649	1.113	1.014	2.009	0.954	C2 domain-containing family protein	GI-H
MELO3C002086.2	5.469	8.371	29.168	9.424	4.785	21.156	6.761	NADH dehydrogenase [ubiquinone] 1 beta subcomplex subunit 10-B	GI-H
MELO3C002067.2	2.717	3.259	8.957	1.502	3.257	1.286	3.835	RNA helicase	GI-H
MELO3C002064.2	0.639	0.984	1.574	0.506	0.551	1.147	0.501	phospholipid-transporting ATPase 10-like	GI-H
MELO3C002057.2	2.377	2.972	7.482	3.241	2.921	1.497	2.107	transcription factor BIM1-like isoform X2	GI-H
MELO3C002054.2	2.483	2.264	6.209	0.962	1.684	1.147	2.259	Movement protein binding protein 2C	GI-H
MELO3C002050.2	1.762	2.725	16.797	NA	1.313	2.365	1.096	MADS box transcription factor	GI-H
MELO3C002049.2	0.349	0.807	1.619	0.451	0.118	1.709	0.109	MADS-box transcription factor	GI-H
MELO3C002041.2	0.917	1.140	2.428	0.512	1.131	1.261	0.905	RHOMBOID-like protein 12, mitochondrial	GI-H
MELO3C002040.2	1.253	1.296	5.179	1.077	1.505	0.401	0.896	Haloacid dehalogenase-like hydrolase (HAD) superfamily protein	GI-H
MELO3C002033.2	1.428	2.939	4.551	1.565	1.271	3.342	0.842	glycerophosphodiester phosphodiesterase GDPD6	GI-H
MELO3C002019.2	0.573	0.583	3.798	NA	0.818	0.584	0.601	protein LEO1 homolog	GI-H
MELO3C002018.2	3.784	4.399	12.429	2.248	3.945	4.284	3.867	Dentin sialophosphoprotein-like protein	GI-H
MELO3C002015.2	0.339	1.029	4.975	0.410	1.349	0.774	0.647	Myosin heavy chain-like protein	GI-H

Gene ID	FPKM							Gene Description	Specific in episode
	FS	GI-M	GM-M	AN-M	GI-H	GM-H	AN-H		
MELO3C002014.2	0.832	1.248	4.282	NA	0.851	1.082	0.483	SNAP25 homologous protein SNAP33	GI-H
MELO3C002008.2	0.719	1.257	2.107	1.278	0.190	2.091	0.625	Plant calmodulin-binding-like protein	GI-H
MELO3C002006.2	1.157	2.135	4.190	1.977	2.059	2.184	1.664	T-box transcription factor, putative (DUF863)	GI-H
MELO3C001973.2	3.097	3.089	45.812	2.267	4.035	0.593	5.151	NAP1-related protein 2	GI-H
MELO3C001970.2	0.583	0.325	3.525	0.271	0.344	0.600	0.206	RP/EB family microtubule-associated protein	GI-H
MELO3C001956.2	0.806	0.986	3.547	0.989	0.705	2.843	0.846	Sucrose synthase	GI-H
MELO3C001953.2	1.348	2.821	6.782	1.382	2.352	2.172	1.816	IBR domain-containing protein	GI-H
MELO3C001932.2	2.627	2.385	11.187	1.914	3.699	1.462	2.770	Kinase family protein	GI-H
MELO3C001924.2	0.203	0.346	1.776	0.211	0.656	0.172	0.220	N-alpha-acetyltransferase 40	GI-H
MELO3C001916.2	0.284	0.290	1.844	0.244	0.320	0.339	0.240	chaperone protein DnaJ isoform X2	GI-H
MELO3C009127.2	0.851	1.277	1.782	NA	0.135	0.260	0.166	WRKY family transcription factor family protein	GI-H
MELO3C023678.2	1.636	0.307	3.497	1.002	0.908	0.729	0.130	Extensin	GI-H
MELO3C026740.2	11.572	17.978	34.053	1.629	5.510	0.947	1.148	WRKY transcription factor	GI-H
MELO3C006037.2	2.275	4.396	6.959	0.602	1.097	0.299	0.361	WRKY transcription factor	GI-H
MELO3C011749.2	0.629	0.504	1.294	0.258	0.541	0.568	0.108	thioredoxin-like protein YLS8	GI-H
MELO3C027188.2	0.471	0.465	1.001	0.224	0.183	3.053	0.381	Rhamnogalacturonate lyase	GI-H
MELO3C028475.2	0.521	0.372	1.110	0.810	0.140	0.154	0.401	Tm-1 protein	GI-H
MELO3C023673.2	1.025	0.653	2.409	0.520	0.705	0.221	0.688	Ribonuclease P/MRP protein subunit POP5	GI-H
MELO3C024895.2	0.808	1.718	1.869	0.647	0.395	0.672	0.833	Cytokinin riboside 5'-monophosphate phosphoribohydrolase LOG	GI-H
MELO3C023449.2	1.049	0.663	3.323	NA	0.771	0.521	0.459	UPF0481 plant-like protein	GI-H
MELO3C015351.2	1.389	0.603	3.103	1.160	0.771	NA	0.941	Superoxide dismutase 2	GI-H
MELO3C015610.2	1.003	0.733	2.246	0.586	0.624	0.185	0.720	Mucin-like protein	GI-H
MELO3C015679.2	1.058	0.724	2.426	0.551	0.732	0.866	0.919	octanoyltransferase	GI-H
MELO3C015088.2	0.488	0.943	1.118	NA	0.157	NA	0.332	IQ domain-containing protein IQM2	GI-H
MELO3C017225.2	0.727	1.119	2.286	NA	0.366	0.489	0.558	Formate hydrogenlyase subunit 5	GI-H
MELO3C008192.2	1.089	0.413	2.317	0.222	0.140	NA	0.351	Transcription factor TFIIIC tau55-related protein	GI-H
MELO3C030303.2	0.610	0.664	1.574	NA	0.251	NA	0.252	Transmembrane protein, putative	GI-H
MELO3C030305.2	1.396	1.319	3.193	1.190	0.819	0.542	0.773	enhancer of rudimentary homolog	GI-H
MELO3C011003.2	0.310	NA	1.601	0.310	0.175	NA	0.335	transcription factor E2FC isoform X3	GI-H
MELO3C003418.2	3.342	4.300	7.062	1.890	1.707	10.653	1.379	PLATZ transcription factor family protein	GI-H

Gene ID	FPKM							Gene Description	Specific in episode
	FS	GI-M	GM-M	AN-M	GI-H	GM-H	AN-H		
MELO3C030414.2	0.780	0.359	1.622	1.506	0.519	0.300	0.723	Unknown protein	GI-H
MELO3C003678.2	1.150	2.009	3.304	NA	0.575	3.233	0.682	DNA-directed RNA polymerase subunit beta	GI-H
MELO3C030466.2	0.885	0.836	1.785	1.065	0.508	2.164	0.397	FAR1-related sequence 6 isoform 1	GI-H
MELO3C003800.2	0.587	0.784	1.407	0.537	0.315	0.989	0.321	AAA-ATPase At3g50940-like	GI-H
MELO3C009830.2	0.663	1.051	1.854	NA	0.359	1.665	0.618	FK506-binding-like protein	GI-H
MELO3C003998.2	4.043	3.167	9.727	0.826	3.321	NA	4.356	Proline--tRNA ligase	GI-H
MELO3C004188.2	0.570	0.454	1.449	0.278	0.287	0.527	0.388	Pentatricopeptide repeat-containing protein	GI-H
MELO3C004580.2	0.982	0.835	1.987	0.393	0.592	0.897	0.583	SNW/SKI-interacting protein-like	GI-H
MELO3C006010.2	2.079	1.725	5.139	0.780	1.720	0.929	0.786	Krueppel-like factor 17	GI-H
MELO3C006013.2	1.166	1.237	2.385	0.696	0.940	1.719	1.038	Transmembrane protein, putative	GI-H
MELO3C006159.2	0.492	0.304	1.461	0.260	0.262	NA	0.323	MADS-box protein	GI-H
MELO3C006182.2	0.400	0.324	1.214	0.083	0.230	NA	0.108	Pentatricopeptide repeat-containing protein At1g01970	GI-H
MELO3C014085.2	1.885	1.462	5.196	0.766	0.731	1.499	0.606	Transcription factor	GI-H
MELO3C016732.2	1.183	1.693	2.637	1.279	0.414	1.514	0.754	Calmodulin-binding protein	GI-H
MELO3C017570.2	1.485	0.975	4.135	NA	0.896	0.675	0.918	transcription termination factor MTERF5, chloroplastic	GI-H
MELO3C017574.2	0.478	0.889	1.706	0.449	0.183	NA	0.317	Beta-glucosidase, putative	GI-H
MELO3C017645.2	1.900	4.480	7.926	NA	1.095	NA	1.375	ethylene-responsive transcription factor ERF106	GI-H
MELO3C008840.2	0.481	0.299	1.251	NA	0.243	0.072	0.226	RVT_1 domain-containing protein/Intron_maturas2 domain-containing protein	GI-H
MELO3C033128.2	3.820	6.344	10.749	2.427	1.837	1.556	3.096	50S ribosomal protein L18	GI-H
MELO3C003186.2	1.205	5.057	2.993	1.924	0.839	2.293	0.564	Proteasome inhibitor-related	GI-H
MELO3C022227.2	1.176	1.283	2.636	0.568	0.820	0.463	0.656	ATP-dependent protease La (LON) domain protein	GI-H
MELO3C021586.2	0.619	0.619	1.297	1.307	0.301	2.174	0.488	serine/threonine-protein kinase HT1	GI-H
MELO3C022845.2	0.601	0.606	1.369	1.114	0.463	2.310	0.332	RING-type E3 ubiquitin transferase	GI-H
MELO3C005191.2	3.902	2.078	11.045	NA	1.797	4.187	2.187	Unknown protein	GI-H
MELO3C034036.2	0.717	0.429	2.100	NA	0.440	0.363	0.372	Unknown protein	GI-H
MELO3C011777.2	1.052	1.119	2.594	1.371	0.750	4.406	1.138	F-box/LRR-repeat protein At4g29420	GI-H
MELO3C025175.2	0.741	1.202	1.841	0.907	0.278	2.906	0.314	Protein yippee-like	GI-H
MELO3C034342.2	1.087	1.078	2.471	0.519	0.773	0.281	0.757	PHD finger alfin-like protein	GI-H
MELO3C020096.2	0.759	0.510	1.870	0.436	0.537	0.839	0.552	Neuronal PAS domain protein	GI-H
MELO3C020965.2	0.500	0.442	1.002	0.257	0.348	0.317	0.457	Violaxanthin de-epoxidase, chloroplastic	GI-H

Gene ID	FPKM							Gene Description	Specific in episode
	FS	GI-M	GM-M	AN-M	GI-H	GM-H	AN-H		
MELO3C024776.2	1.287	0.833	2.685	0.543	0.642	0.536	0.670	Zinc finger with UFM1-specific peptidase domain protein	GI-H
MELO3C035058.2	0.707	0.473	2.200	0.289	0.369	0.513	0.526	Plant/protein (DUF789)	GI-H
MELO3C021416.2	8.139	13.973	16.636	6.038	6.654	21.266	5.565	zinc finger A20 and AN1 domain-containing stress-associated protein 3	GI-H
MELO3C021765.2	2.862	1.674	6.778	2.199	1.573	0.404	2.148	oxysterol-binding protein-related protein 2B-like	GI-H
MELO3C002869.2	0.611	0.665	1.869	NA	0.323	0.342	0.504	transcription termination factor MTERF15, mitochondrial	GI-H
MELO3C002821.2	2.156	3.512	6.697	3.825	0.926	2.748	1.126	Unknown protein	GI-H
MELO3C002231.2	1.598	2.132	3.458	1.235	0.998	1.683	1.620	AT3g47831/T23J7	GI-H
MELO3C023478.2	3.505	3.654	7.946	1.288	2.951	0.148	2.210	nudix hydrolase 3-like	GI-H
MELO3C015513.2	1.779	1.155	3.756	0.330	1.817	NA	1.191	growth-regulating factor 4	GI-H
MELO3C017182.2	4.632	7.240	13.268	NA	6.034	1.343	2.237	Photosynthetic NDH subcomplex B 4	GI-H
MELO3C026232.2	0.828	0.390	1.999	NA	0.530	0.363	0.226	C2H2-like zinc finger protein	GI-H
MELO3C011379.2	1.397	1.648	3.080	1.413	1.280	1.663	0.847	Chaperone protein dnaJ	GI-H
MELO3C018199.2	1.140	1.198	2.885	0.526	1.002	NA	0.887	Pentatricopeptide repeat-containing protein-like protein	GI-H
MELO3C022678.2	14.898	25.058	41.893	10.894	11.154	2.879	2.987	Protein TIFY 10B	GI-H
MELO3C030909.2	0.358	0.253	1.586	NA	0.309	NA	0.155	methyltransferase-like protein 13	GI-H
MELO3C009961.2	1.222	3.222	2.511	1.887	1.116	3.699	0.867	At1g23710	GI-H
MELO3C009829.2	0.942	0.620	3.649	NA	1.192	NA	0.346	DNA-(apurinic or apyrimidinic site) lyase	GI-H
MELO3C009352.2	0.866	1.044	1.985	0.574	0.770	0.952	0.486	transcription initiation factor TFIID subunit 8-like	GI-H
MELO3C031411.2	0.348	0.207	1.853	NA	0.434	NA	0.217	PHD finger alfin-like protein	GI-H
MELO3C006491.2	0.937	1.022	3.513	NA	1.589	1.190	0.419	Calmodulin, putative	GI-H
MELO3C006578.2	1.520	3.934	3.111	2.902	1.257	5.239	0.888	AWPM-19-like membrane family protein	GI-H
MELO3C006800.2	0.915	0.671	7.967	NA	1.871	NA	0.073	Strictosidine synthase family protein	GI-H
MELO3C019461.2	1.602	1.687	3.555	NA	1.285	1.851	0.904	LOW QUALITY PROTEIN: probable receptor-like protein kinase At1g11050	GI-H
MELO3C016859.2	1.136	0.619	2.804	0.866	0.832	NA	0.587	DUF1764 domain-containing protein	GI-H
MELO3C032690.2	0.359	0.491	1.080	NA	0.446	0.300	0.170	AP-3 complex subunit sigma-like	GI-H
MELO3C016392.2	0.902	0.864	2.735	0.428	1.362	0.542	0.379	maf-like protein DDB_G0281937	GI-H
MELO3C017697.2	0.358	0.267	1.171	0.299	0.409	0.336	0.105	Kinase interacting family protein, putative	GI-H
MELO3C007533.2	0.509	1.701	1.182	3.401	0.524	1.647	0.382	Salicylic acid-binding protein 2	GI-H
MELO3C007855.2	0.238	0.258	1.381	NA	0.680	NA	0.115	Protein pleiotropic regulatory locus 1	GI-H
MELO3C003121.2	1.359	2.191	2.797	0.806	1.201	0.244	0.937	Unknown protein	GI-H

Gene ID	FPKM							Gene Description	Specific in episode
	FS	GI-M	GM-M	AN-M	GI-H	GM-H	AN-H		
MELO3C026419.2	0.683	NA	3.203	1.938	0.731	NA	0.340	C2 domain-containing protein	GI-H
MELO3C034289.2	1.826	NA	6.160	3.167	1.912	NA	0.613	40S ribosomal protein S13-like	GI-H
MELO3C023199.2	0.487	0.432	2.234	NA	0.453	0.398	0.199	Arginine/glutamate-rich 1 protein	GI-H
MELO3C020879.2	0.858	0.662	1.890	NA	0.617	NA	0.296	Globin	GI-H
MELO3C019223.2	0.549	0.338	1.199	0.346	0.546	0.297	0.282	BTB/POZ domain-containing protein At3g22104	GI-H
MELO3C002226.2	0.656	0.409	2.549	0.353	1.047	NA	0.492	polynucleotide 5'-hydroxyl-kinase NOL9	GI-H
MELO3C025311.2	2.853	11.008	8.888	2.781	34.648	1.630	0.936	omega-hydroxypalmitate O-feruloyl transferase	GM-H
MELO3C031734.2	3.527	3.526	1.667	4.330	9.706	0.776	4.470	Bidirectional sugar transporter SWEET	GM-H
MELO3C014872.2	0.463	11.550	0.945	4.307	2.214	10.370	1.102	Lipase	GM-H
MELO3C016616.2	2.561	49.345	1.978	25.013	6.008	1.372	2.757	Indole-3-acetic acid-amido synthetase GH3.3	GM-H
MELO3C017776.2	3.505	3.785	2.254	2.085	9.417	1.301	4.034	Peptidylprolyl isomerase	GM-H
MELO3C025486.2	3.581	55.656	2.518	78.809	8.950	12.583	2.947	ABC transporter G family member 6	GM-H
MELO3C005631.2	0.163	22.870	0.113	0.721	1.145	0.107	0.188	sugar carrier protein C-like	GM-H
MELO3C025894.2	4.118	9.016	3.404	4.554	8.969	8.219	4.450	Two-component response regulator	GM-H
MELO3C005052.2	0.343	0.628	0.752	2.882	1.639	5.979	0.405	Laccase	GM-H
MELO3C010969.2	4.851	5.560	3.257	6.935	10.222	0.803	4.981	transmembrane protein 136	GM-H
MELO3C016712.2	2.664	4.838	2.115	1.127	6.078	2.998	1.904	Stem-specific protein TSJT1	GM-H
MELO3C022107.2	0.332	4.864	0.225	1.540	1.011	0.454	0.324	Methylesterase 17	GM-H
MELO3C005224.2	2.113	2.029	0.992	0.608	5.616	44.322	1.191	Glutamate decarboxylase	GM-H
MELO3C005816.2	0.629	0.714	0.645	0.832	2.276	0.571	0.631	Receptor protein kinase, putative	GM-H
MELO3C020044.2	0.712	1.685	0.694	0.938	1.760	0.683	0.715	PHD finger protein MALE STERILITY 1	GM-H
MELO3C009495.2	0.651	0.411	0.066	6.644	0.204	3.778	1.631	CASP-like protein	AN-H
MELO3C017055.2	0.637	0.871	0.210	0.296	0.945	0.686	1.989	Phosphate transporter	AN-H
MELO3C007508.2	1.380	5.898	0.371	19.308	1.184	2.029	2.937	Cold acclimation protein	AN-H
MELO3C001303.2	0.525	0.223	0.838	NA	0.908	0.053	3.566	Subtilisin-like protease	AN-H
MELO3C010979.2	1.209	1.771	1.064	6.687	2.027	25.442	4.463	WAT1-related protein	AN-H
MELO3C031014.2	0.215	0.572	0.212	4.827	0.280	3.038	2.016	Protein nuclear fusion defective 4	AN-H
MELO3C005585.2	4.803	4.118	4.414	4.571	6.221	0.443	13.430	F5O11.10 isoform 1	AN-H
MELO3C029970.2	0.556	0.993	0.532	7.145	0.161	4.608	1.285	protein NRT1/ PTR FAMILY 3.1	AN-H
MELO3C010965.2	0.526	1.177	0.459	1.603	0.414	0.698	1.066	omega-hydroxypalmitate O-feruloyl transferase	AN-H
MELO3C012939.2	1.817	4.840	1.272	10.106	1.693	20.916	3.859	Caffeoylshikimate esterase	AN-H

Gene ID	FPKM							Gene Description	Specific in episode
	FS	GI-M	GM-M	AN-M	GI-H	GM-H	AN-H		
MELO3C006552.2	0.791	1.474	0.845	2.605	0.879	0.571	2.017	Glucose-1-phosphate adenylyltransferase	AN-H
MELO3C019481.2	0.453	1.008	0.355	2.322	0.305	2.021	1.081	E3 ubiquitin-protein ligase RING1-like	AN-H
MELO3C004789.2	1.672	1.086	1.107	1.271	1.734	0.783	4.013	DUF868 family protein, putative (DUF868)	AN-H
